# Supplementary material for: DDX5 inhibits inflammation by modulating m6A levels of TLR2/4 transcripts during bacterial infection
Source: EMBO Rep. 2024 Jan 5;25(2):19. doi: 10.1038/s44319-023-00047-9 (PMC10897170; doi:10.1038/s44319-023-00047-9)
Supplement: Supplementary file 1 — Appendix [file 44319_2023_47_MOESM1_ESM.pdf]

Appendix

Table of Contents Page

Appendix Figure S1 and Figure Legend……………………………………………………..2

Appendix Figure S2 and Figure Legend……………………………………………………..3

Appendix Figure S3 and Figure Legend……………………………………………………..4

Appendix Figure S4 and Figure Legend……………………………………………………..5

Appendix Figure S5 and Figure Legend……………………………………………………..6

Appendix Figure S6 and Figure Legend……………………………………………………..7

Appendix Figure S7 and Figure Legend……………………………………………………..8

Appendix Figure S8 and Figure Legend……………………………………………………..9

Appendix Figure S9 and Figure Legend……………………………………………………10

Appendix Figure S10 and Figure Legend…………………………………………………11

Appendix Figure S11 and Figure Legend………………………………………………....12

Appendix Figure S12 and Figure Legend…………………………………………………13

Appendix Figure S13 and Figure Legend…………………………………………………14

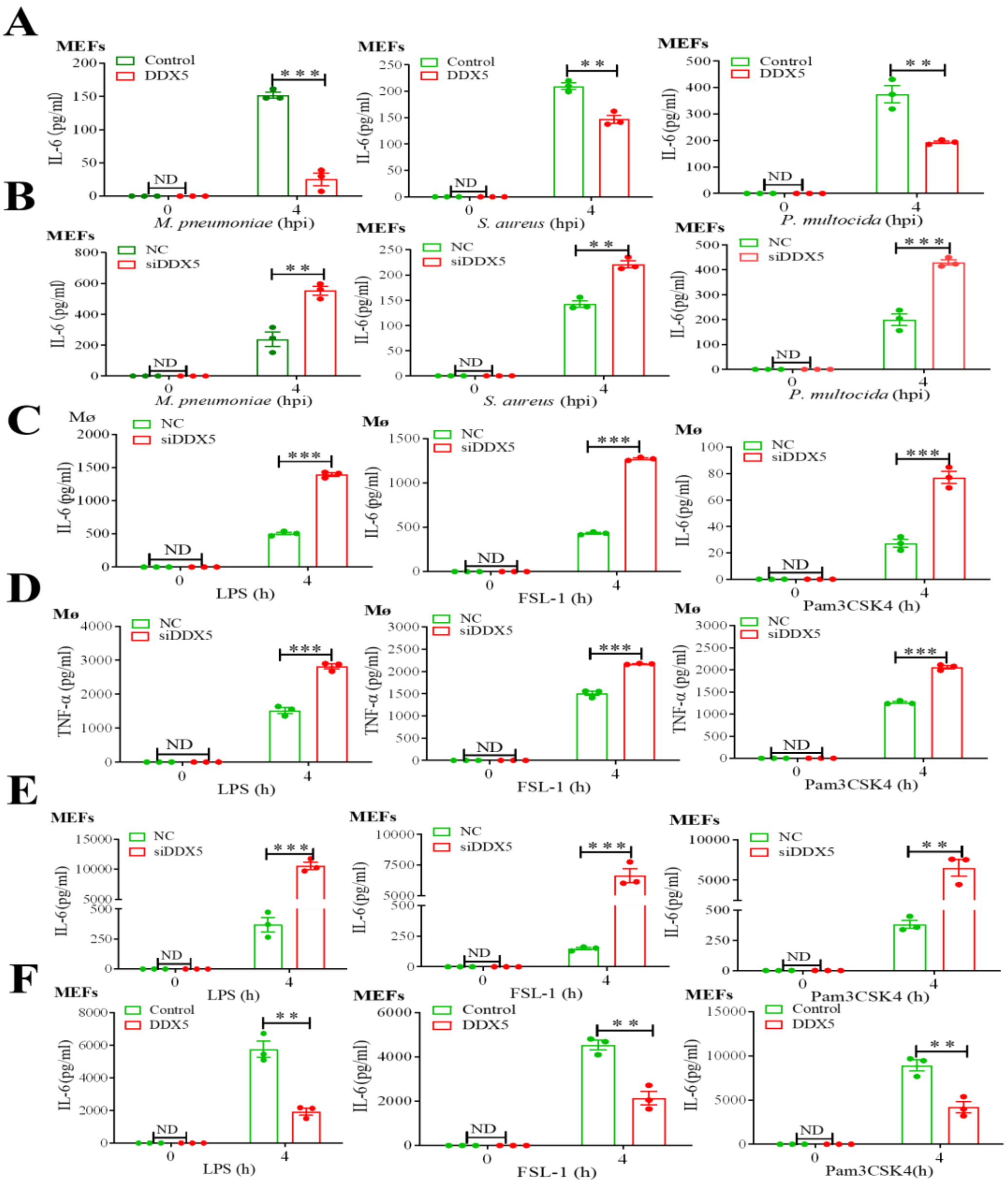

Appendix Figure S1. DDX5 suppresses the production of IL-6 and TNF-α triggered by pathogenic bacteria or TLR2/4 agonists, related to Figure 1.

A-B: IL-6 production in control and DDX5-overexpressing MEFs (A) or in NC and siDDX5 MEFs (B) infected with *P. multocida* or *S. aureus*, *M. pneumonia* for 4 h (n = 3).

C-D: IL-6 (C) or TNF-α (D) production in NC and siDDX5 Mø treated with LPS, FSL-1, and Pam3CSK4 for 4 h (n = 3).

E-F: IL-6 production in NC and siDDX5 MEFs (E) or in control and DDX5-overexpressing MEFs (F) treated with LPS, FSL-1, and Pam3CSK4 for 4 h (n = 3).

Data information: In (A-F), all data are represented as the mean ± SEM of three biologically independent samples. ND, not detected. "ns" indicates no significant difference, *p < 0.05, **p < 0.01, and ***p<0.001 (Student's *t*-test).

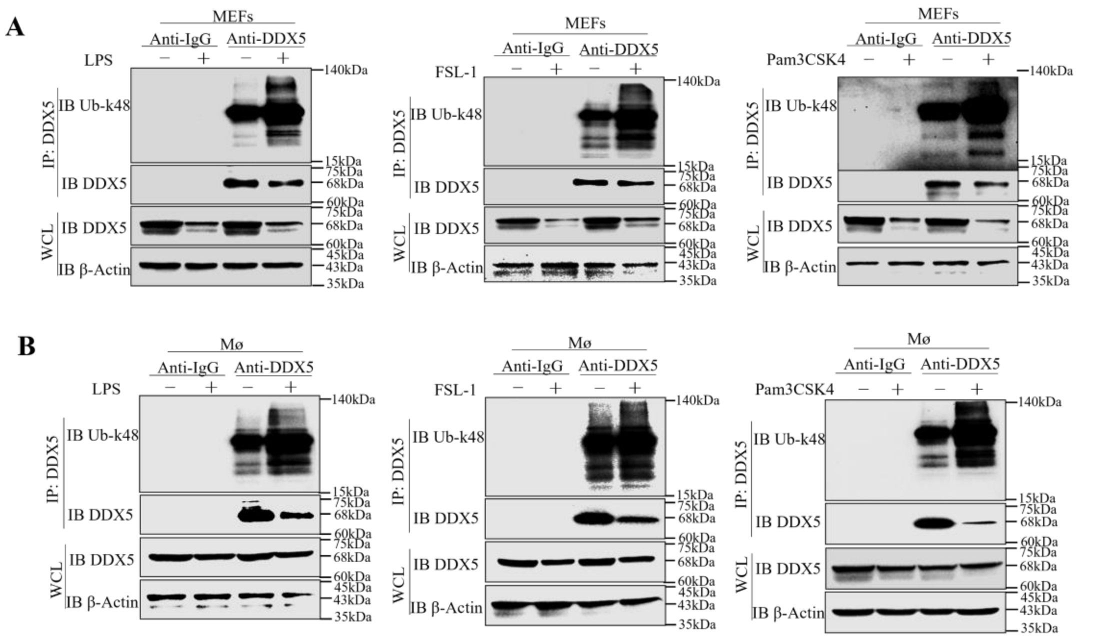

Appendix Figure S2. Bacterial infection led to DDX5 depression in K48-linked ubiquitin modification, related to Figure 2 and Figure EV1.

A: K48-linked ubiquitination of DDX5 in MEFs treated with LPS, FSL-1, and Pam3CSK4.

B: K48-linked ubiquitination of DDX5 in Mø treated with LPS, FSL-1, and Pam3CSK4.

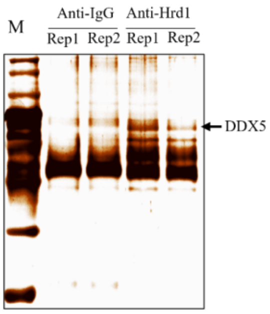

Accession	Protein name
Q61656	***Probable ATP-dependent RNA helicase DDX5***
Q501J6	Probable ATP-dependent RNA helicase DDX17
P63017	Heat shock cognate 71 kDa protein
P20029	Endoplasmic reticulum chaperone BiP
Q61188	Histone-lysine N-methyltransferase EZH2

Appendix Figure S3. Hrd1 pull-down assay in MEFs, related to Figure 3.

MEFs were seeded and cultured on 60-mm dishes for 24 h; then, the cells were lysed with NP40 lysis buffer containing protease inhibitor cocktail (Roche). Lysates were immunoprecipitated with a Rabbit anti-Hrd1 antibody or Rabbit IgG in conjunction with protein G/A-magnetic beads. Immunoprecipitated Hrd1 and its associated proteins from MEFs were then resolved on a PAGE gel for the indicated time. Different bands were analyzed by LC/MS. Arrow indicates the band of DDX5 protein detected by LC/MS.

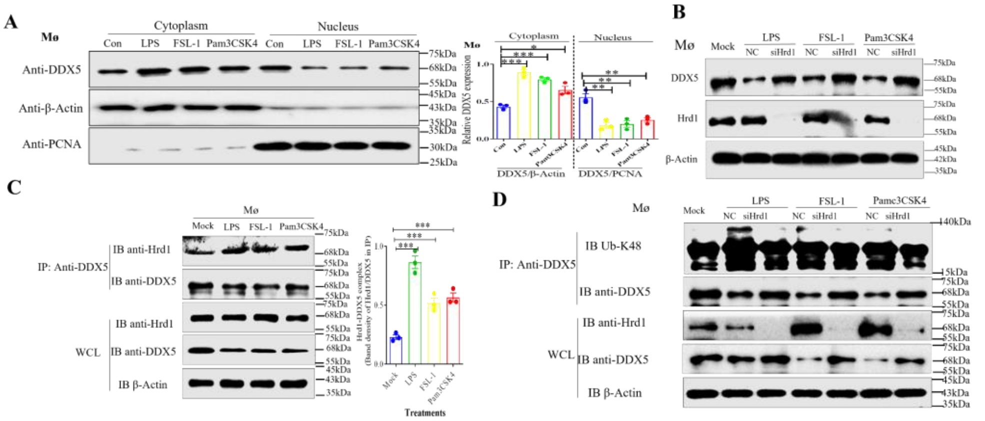

Appendix Figure S4. DDX5 is degraded in mouse macrophages by Hrd1 triggered by TLR2/4 agonists, related to Figure 3.

A: Redistribution of DDX5 after treatment of TLR2/TLR4 agonists. Mouse macrophages (Mø) were treated with LPS, FSL-1, and Pam3CSK4 respectively, harvested to extract the nuclear and cytoplasmic protein for western blot assay to detect the expression of DDX5. β-Actin acted as a cytoplasmic reference control, and PCNA was a nuclear reference control. The expression of cytoplasmic DDX5 was quantified by the relative intensity of DDX5/β-Actin, and the expression of nuclear DDX5 was quantified by the relative intensity of DDX5/PCNA . The band intensity was measured by Image J software.

B: DDX5 level in Hrd1 knockdown Mø after treatment with LPS, FSL-1, and Pam3CSK4; NC served as the siRNA negative control.

C: Interaction between Hrd1 and DDX5 in Mø after treatment with LPS, FSL-1, and Pam3CSK4 by Co-IP with an anti-DDX5 antibody. The interaction of Hrd1 and DDX5 was quantified by the band density of Hrd1/DDX5 in the IP system; the band density was measured by Image J software.

D: The K48-linked ubiquitin modification of DDX5 in Hrd1 knockdown Mø after treatment with LPS, FSL-1, and Pam3CSK4; NC served as the siRNA negative control.

Data information: In (A, C), all data are represented as the mean ± SEM of three biologically independent samples. “ns” indicates no significant difference, *p < 0.05, **p < 0.01 and ***p < 0.001 (Student’s t-test).

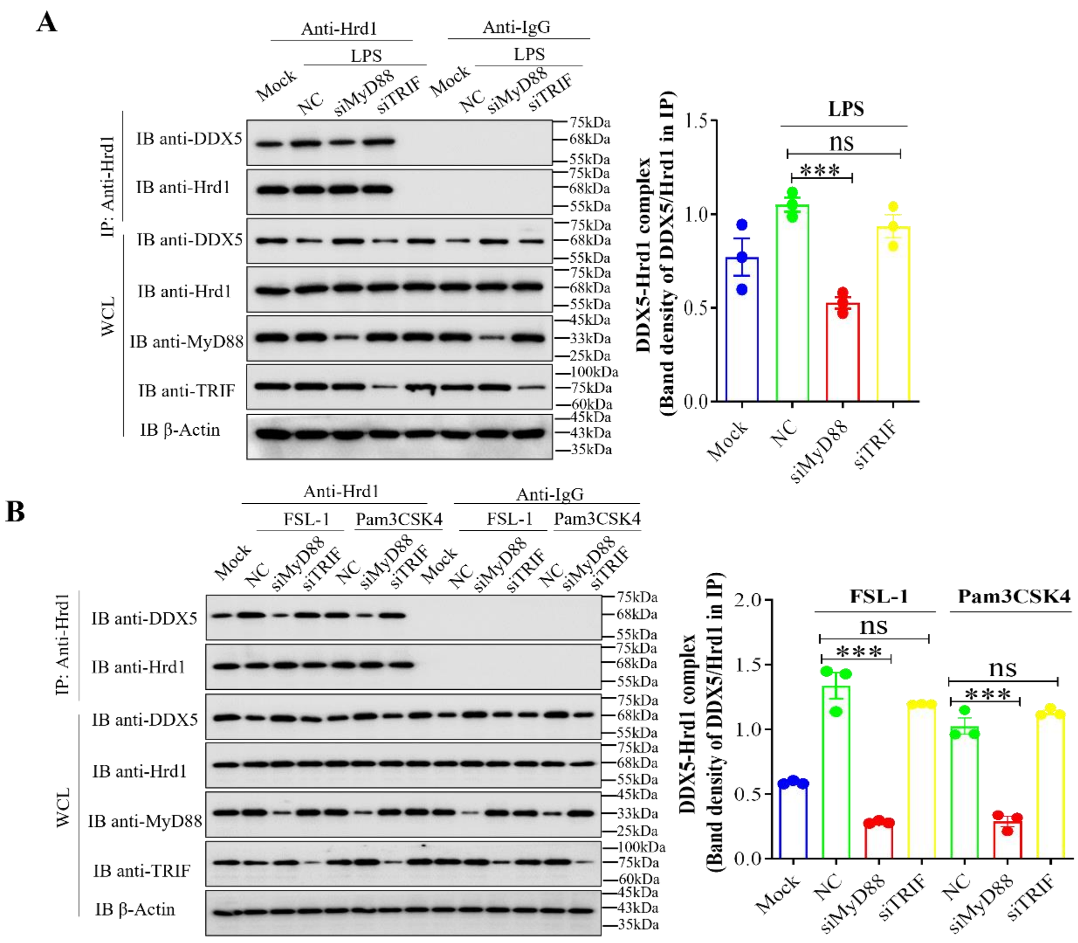

Appendix Figure S5. The recruitment of DDX5 to Hrd1 upon TLR stimulation was MyD88-dependent pathway, related to Figure 3.

MyD88 and TRIF was knocked down in MEFs followed by treatment with LPS (A), and FSL-1 or Pam3CSK4 (B). NC served as the siRNA negative control. The interaction of DDX5 and Hrd1 was examined by Co-IP, and quantified by the relative density of DDX5/Hrd1. The band density was measured by Image J software.

Data information: In (A-B), all data are represented as the mean ± SEM of three biologically independent samples. “ns” indicates no significant difference, *p < 0.05, **p < 0.01 and ***p < 0.001 (Student’s t-test).

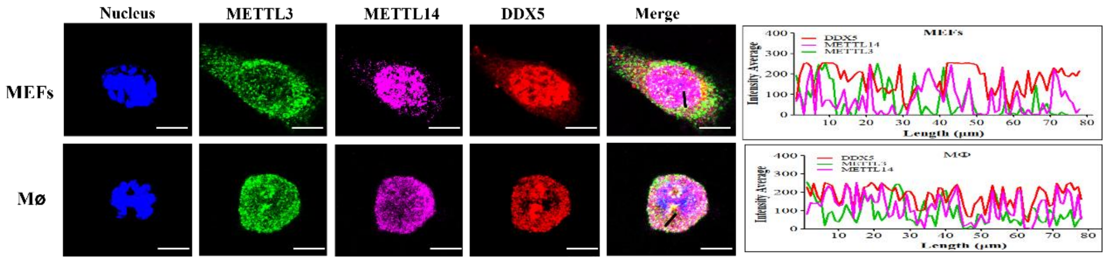

Appendix Figure S6. Co-localization of DDX5, METTL3, and METTL14 in MEFs or mouse macrophages, related to Figure 4.

The MEFs or mouse macrophages (Mø) were seeded on coverslips in 12-well plates and cultured for 18–24 h, then fixed with 4% paraformaldehyde and stained with anti-METTL3, anti-DDX5 and anti-METTL14 before confocal microscopy. Right panels show the pixel intensity of green (METTL3), purple (METTL14) and red (DDX5) from the black line in merge image. Scale bars: 7.5 μm.

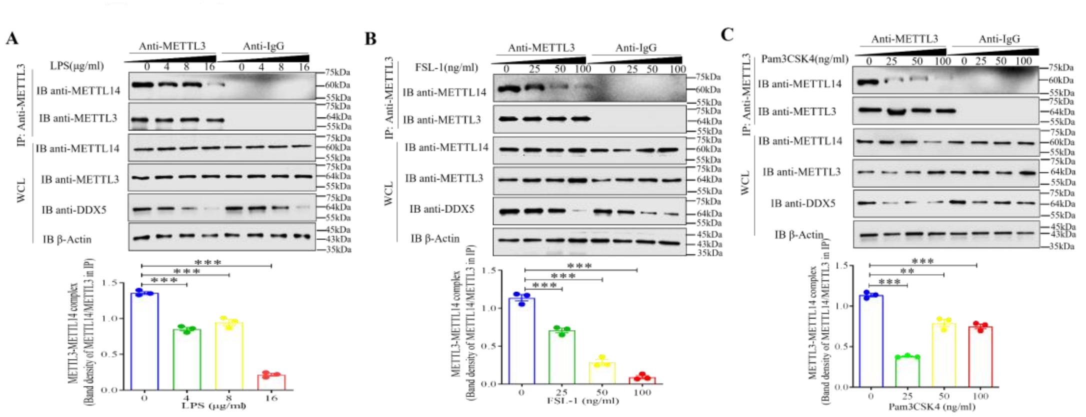

Appendix Figure S7. The interplay of METTL3 and METTL14 in MEFs after treatment with TLR2/TLR4 agonists, related to Figure EV2.

Interaction between METTL3 and METTL14 in MEFs after treatment with different concentrations of LPS (0, 4, 8, 16 μg/ml) (A), FSL-1(0, 25, 50, 100 ng/ml) (B), and Pam3CSK4 (0, 25, 50, 100 ng/ml) (C) by Co-IP with an anti-METTL3 antibody. The interaction of METTL3 and METTL14 was quantified by the band intensity of METTL14/METTL3 in the IP system; the band intensity was measured by Image J software.

Data information: In (A-C), all data are represented as the mean ± SEM of three biologically independent samples. **$p < 0.01$ and ***$p < 0.001$ (Student's t-test).

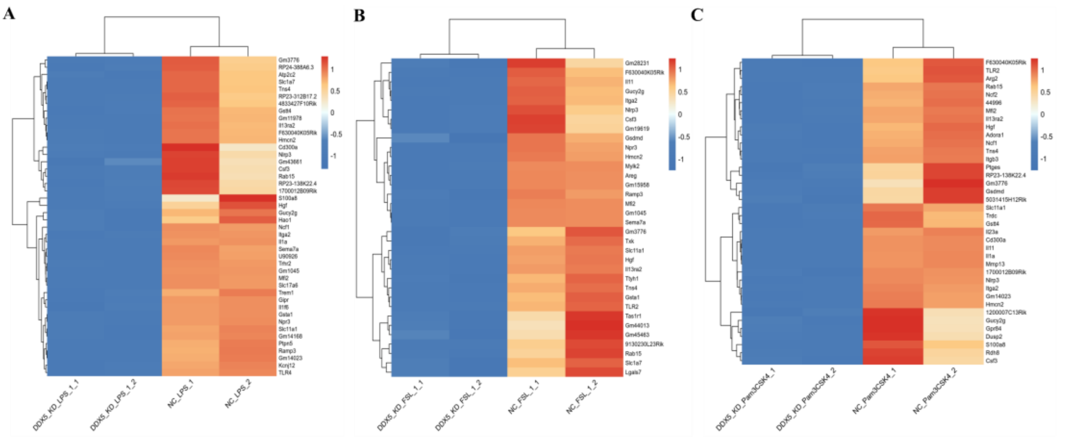

D

m6A Peak of TLR2/4 transcripts	Sequence
m6A *tlr4*	5'-CUACCAAUUUGACAUUCAGUGGACUUUUUCAUUCUCUGUCUUUCCAUAUU-3'
m6A *tlr4*-M	5'-CUACCAAUUUGACAUUCAGUGGBCUUUUUCAUUCUCUGUCUUUCCAUAUU-3'
m6A *tlr2-1*	5'-AUGACUAAGAAUGCAUCUGAUCAGGACAGGGGCAUGUUAUUAUGCAUGUC-3'
m6A *tlr2-1*-M	5'-AUGACUAAGAAUGCAUCUGAUCAGGBCAGGGGCAUGUUAUUAUGCAUGUC-3'
m6A *tlr2*-2	5'-UGGCACUGUGAGGACAGGGCCAGUGAUUUUAGGGUA-3'
m6A *tlr2*-2-M	5'-UGGCACUGUGAGGBCAGGGCCAGUGAUUUUAGGGUA-3'

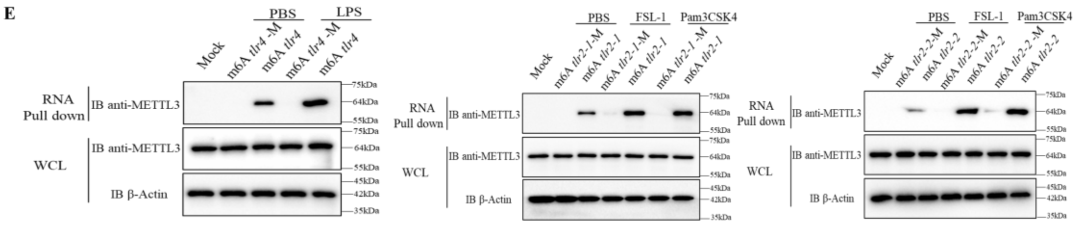

Appendix Figure S8. The analysis of m6A modification of TLR2/4 transcripts based on the MeRIP-seq, related to Figure EV3.

A-C: Heatmap of differential m6A modified transcripts of DDX5 knockdown (KD) MEFs after LPS (A), FSL-1 (B) and Pam3CSK4 (C) treatment. Differential m6A modified transcripts were generated from MeRIP-seq and RNA-seq data (logFC≥2.5).

D: The sequence of m6A peak of TLR2/4 transcripts and its mutants. The sequence of m6A peak of TLR2/4 transcripts were required based on IGV assays of MeRIP-Seq. The underline indicated the m6A motif "RRACH", the red A was the m6A site on TLR2/4, the red B (G/C/U) in mutant was replaced with red A of m6A site on TLR2/4. The black arrow indicated TLR2/4 transcripts.

E: RNA Pull down of m6A peak of TLR2/4 transcripts and its mutants with METTL3 in MEFs. The Biotin labeled m6A peak of TLR2/4 transcripts and its mutants were transfected into MEFs, then treated with TLR2/4 agonists, respectively. MEFs were lysed for RNA Pull down to detect the binding of m6A peak of TLR2/4 transcripts with mutants or METTL3. β-Actin was used as a reference control.

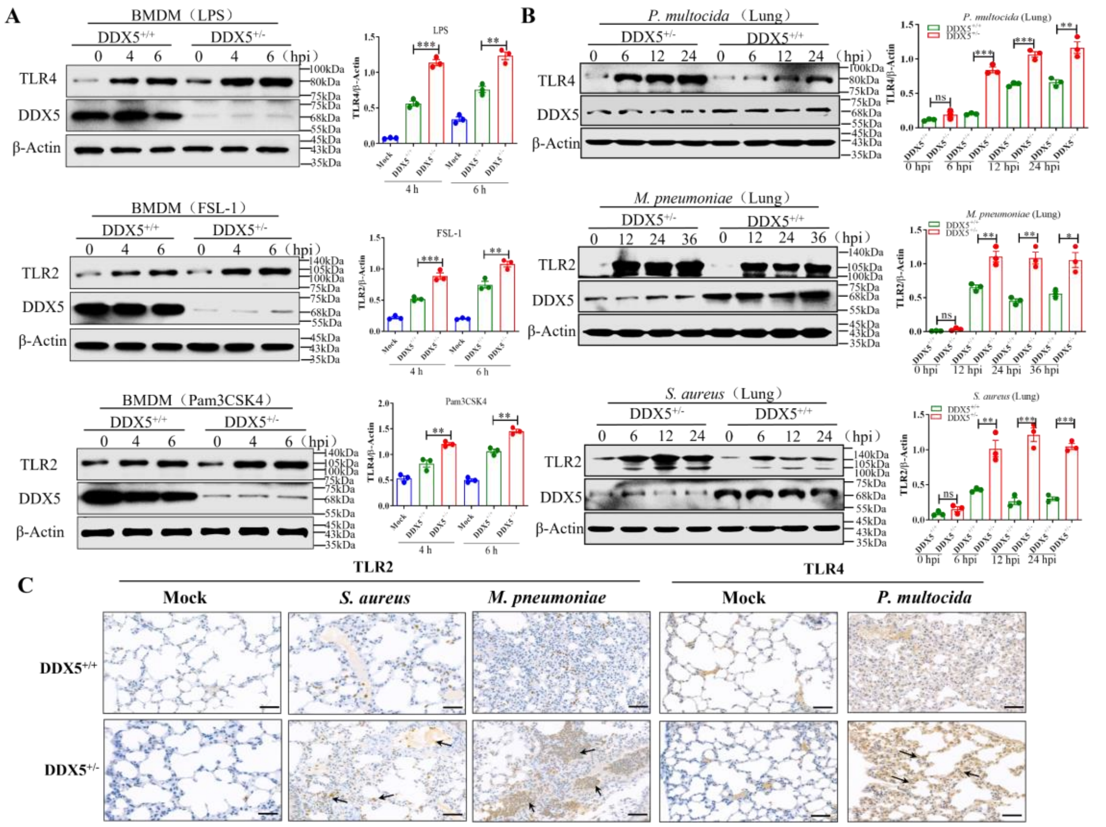

Appendix Figure S9. Expression of TLR4/TLR2 in primary BMDM or lungs of DDX5$^{+/+}$ or DDX5$^{+/-}$ mice, related to Figure 5.

A: Expression of TLR4, TLR2, DDX5, and β-Actin in primary DDX5$^{+/+}$ BMDM or DDX5$^{+/-}$ BMDM treated with LPS, FSL-1 or Pam3CSK4 for 4 or 6 h.

B: Expression of TLR4, TLR2, DDX5, and β-Actin in the lungs of DDX5$^{+/+}$ or DDX5$^{+/-}$ mice infected with *P. multocida* for 6, 12, and 24 h, *M. pneumoniae* for 12, 24, and 36 h, or *S. aureus* for 6, 12, and 24 h. The expression of TLR4 and TLR2 were quantified by the band intensity of DDX5/β-Actin in the western blot; the band intensity was measured by Image J software.

C: Immunohistochemistry analysis of TLR4 in the lungs of DDX5$^{+/+}$ or DDX5$^{+/-}$ mice infected with *P. multocida* for 12 h, and of TLR2 in the lungs of DDX5$^{+/+}$ or DDX5$^{+/-}$ mice infected with *S. aureus* for 12 h and *M. pneumoniae* for 24 h. Scale bars, 50 μm. The black arrows indicated strong positive signals.

Data information: In (A-B), all data are represented as the mean ± SEM of three biologically independent samples. "ns" indicates no significant difference, *p <0.05, **p < 0.01, and ***p < 0.001 (Student's *t*-test).

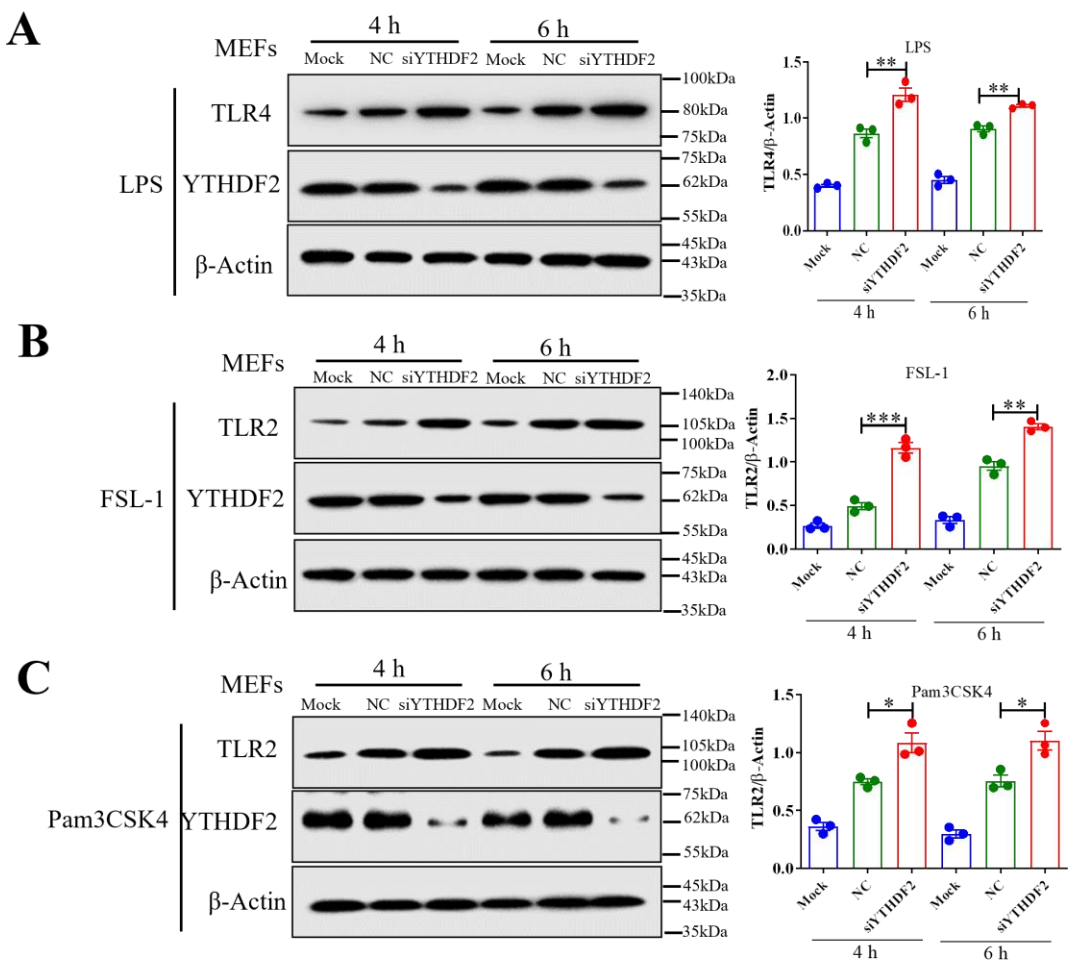

Appendix Figure S10. TLR2/TLR4 protein level in YTHDF2-deficient MEFs treated with TLR2/4 agonists, related to Figure 6.

A-C: TLR4 protein level in NC and siYTHDF2 MEFs treated with LPS for 4 or 6 h (A), or TLR2 protein level in NC and siYTHDF2 MEFs treated with FSL-1 (B) or Pam3CSK4 (C) for 4 or 6 h. The expression of TLR4 and TLR2 were quantified by the band intensity of DDX5/β-Actin in the western blot; the band intensity was measured by Image J software.

Data information: In (A-C), all data are represented as the mean ± SEM of three biologically independent samples. “ns” indicates no significant difference, *p <0.05, **p < 0.01, and ***p < 0.001 (Student’s *t*-test).

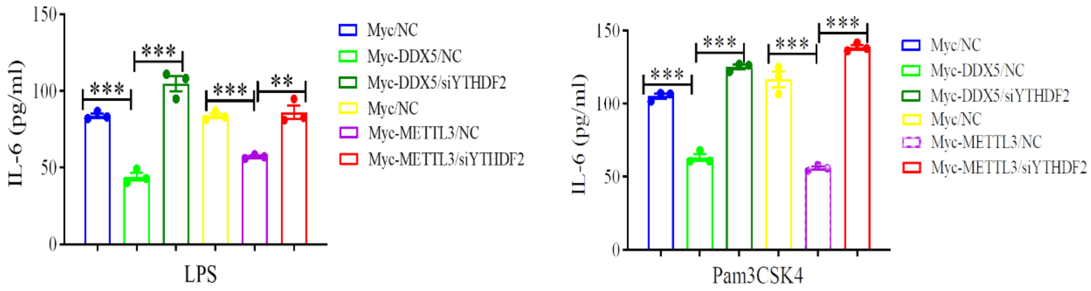

Appendix Figure S11. IL-6 production in Myc-DDX5/Myc-METTL3 overexpressed MEFs with knockdowned-YTHDF2 treated with LPS, and Pam3CSK4, related to Figure 6.

Data information: All data are represented as the mean ± SEM of three biologically independent samples. **$p < 0.01$ and ***$p < 0.001$ (Student's t-test).

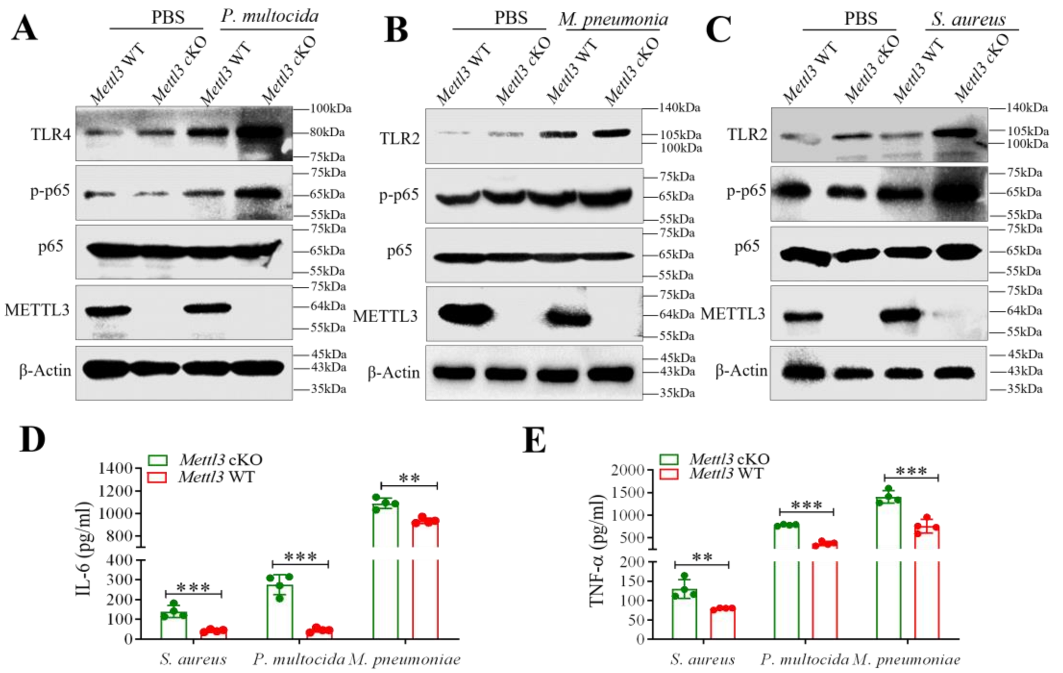

Appendix Figure S12. The expression of TLR4/2 and inflammatory cytokines in primary *Mettl3* WT or *Mettl3* cKO BMDM infected with pathogenic bacteria, related to Figure 7.

A-C: Expression of TLR4, p-p65, DDX5, and β-actin in primary *Mettl3* WT BMDM or *Mettl3* cKO BMDM infected with *P. multocida* for 12 h, *M.pneumoniae* for 24 h (B) or *S. aureus* for 12 h (C), respectively.

D, E: IL-6 (D) or TNF-α (E) production in primary *Mettl3* WT BMDM or *Mettl3* cKO BMDM infected with *P. multocida*, *S. aureus*, or *M. pneumonia*, respectively (n=4).

Data information: In (D-E), all data are represented as the mean ± SEM of three biologically independent samples. *p < 0.05, **p< 0.01, and ***p < 0.001 (Student's *t*-test).

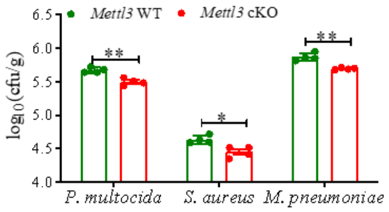

Appendix Figure S13. The bacteria loading of *P. multocida*, *S. aureus*, or *M. pneumoniae* was measured in the lungs of *Mettl3* WT or *Mettl3* cKO mice (n=4), related to Figure 7.

Data information: All data are represented as the mean ± SEM of three biologically independent samples. *p < 0.05, and **p< 0.01 (Student's *t*-test).
