# Supplementary figures and images for: DDX5 inhibits inflammation by modulating m6A levels of TLR2/4 transcripts during bacterial infection (part 1 of 2)
Source: EMBO Rep. 2024 Jan 5;25(2):19. doi: 10.1038/s44319-023-00047-9 (PMC10897170; doi:10.1038/s44319-023-00047-9)

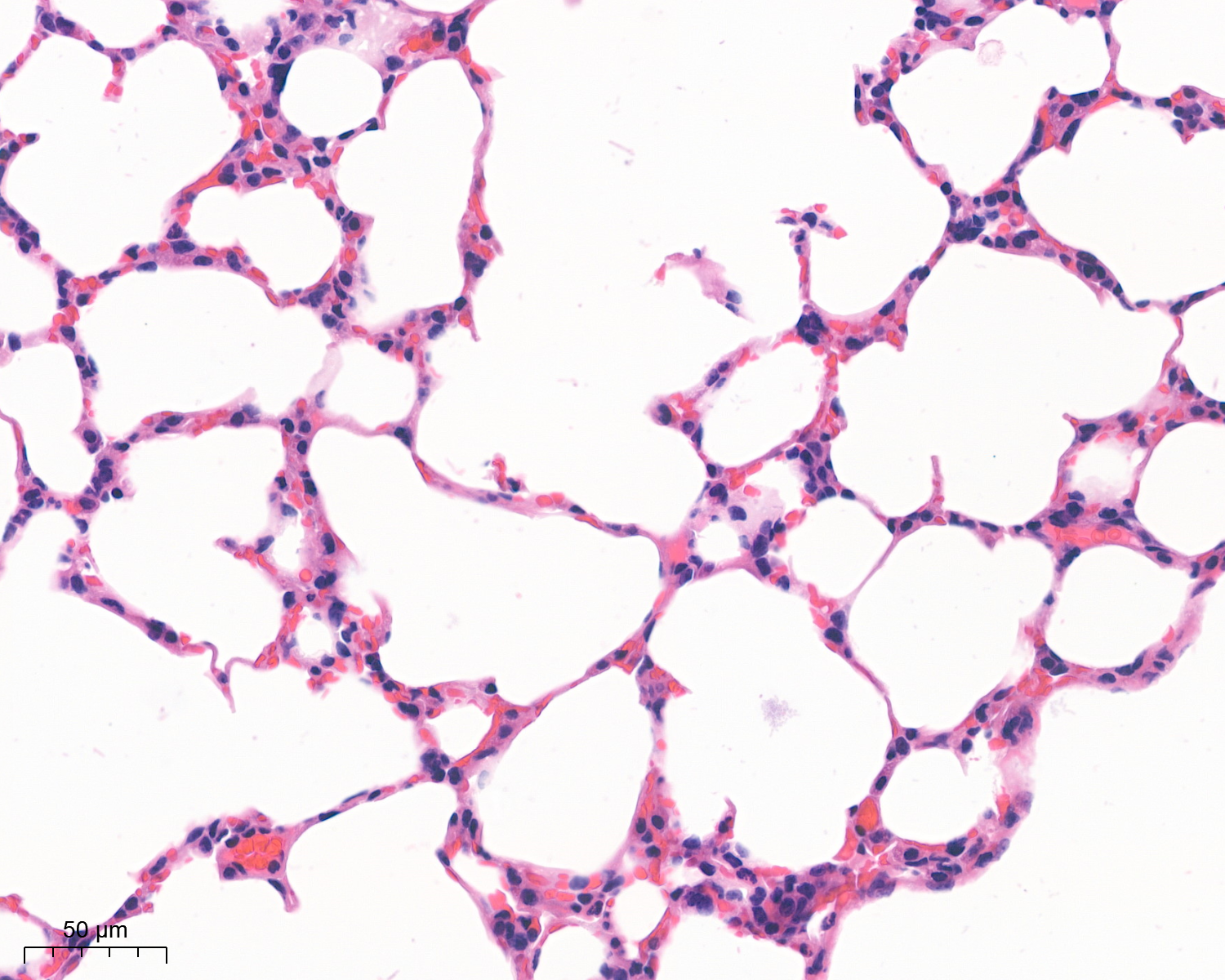

Supplement: Supplementary file 2 — Source Data Fig. 1 [file 44319_2023_47_MOESM2_ESM.zip › EMBOR-2023-57416V3-Figure_1_Source_Data-sd/Figure 1/E/1 DDX5 WT Mock.tif]

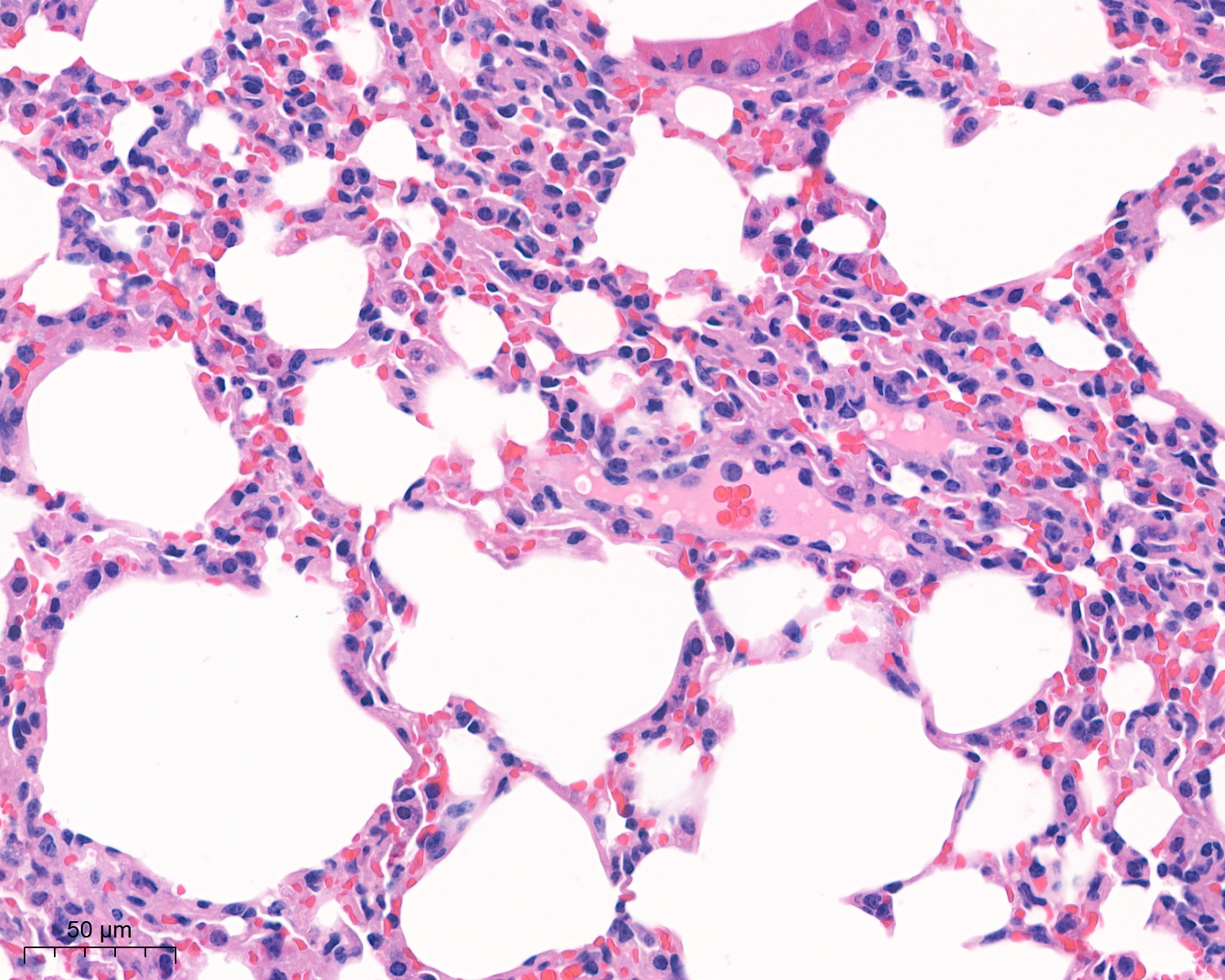

Supplement: Supplementary file 2 — Source Data Fig. 1 [file 44319_2023_47_MOESM2_ESM.zip › EMBOR-2023-57416V3-Figure_1_Source_Data-sd/Figure 1/E/2 DDX5 WT P. multocida.tif]

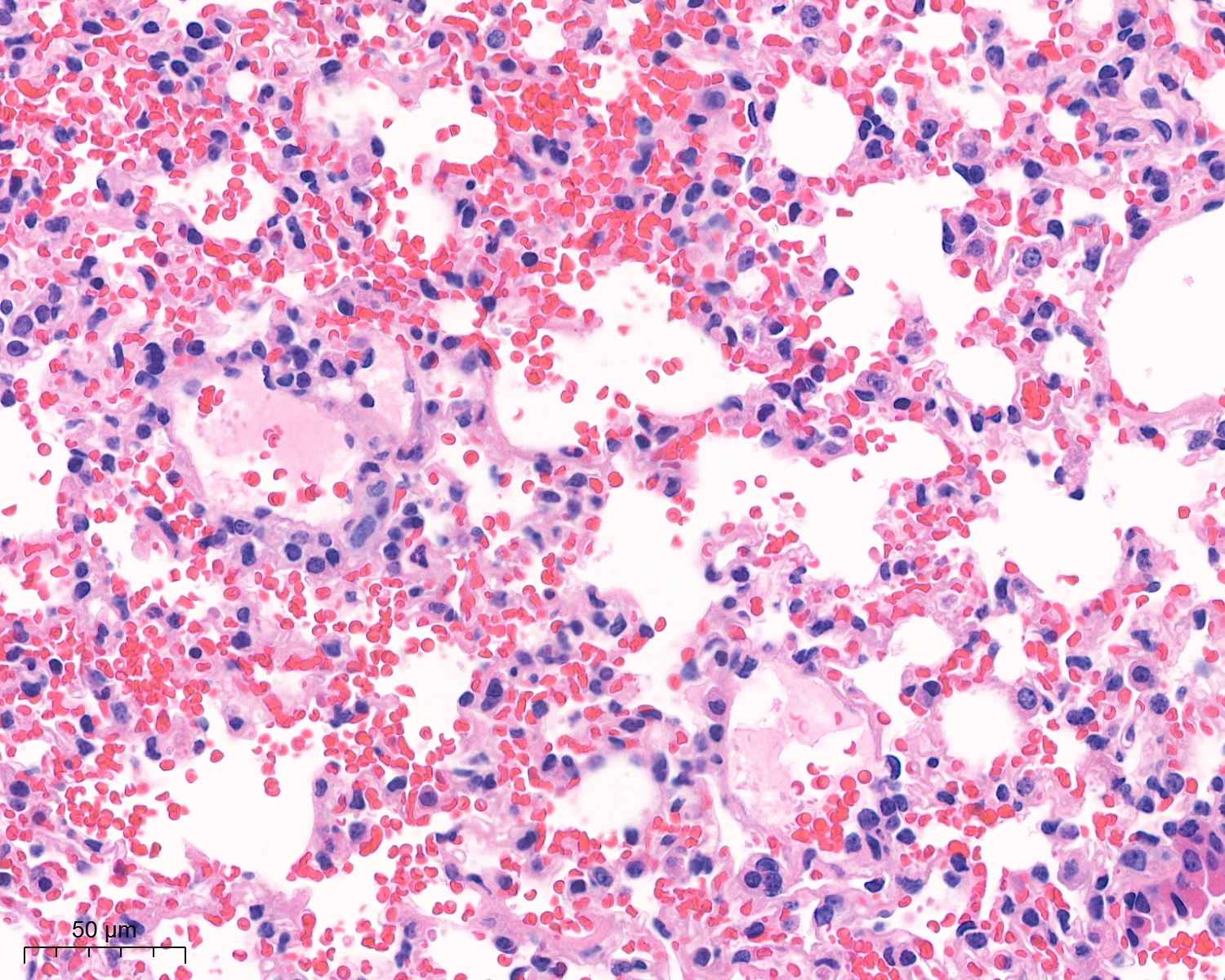

Supplement: Supplementary file 2 — Source Data Fig. 1 [file 44319_2023_47_MOESM2_ESM.zip › EMBOR-2023-57416V3-Figure_1_Source_Data-sd/Figure 1/E/3 DDX5 WT S. aureus.tif]

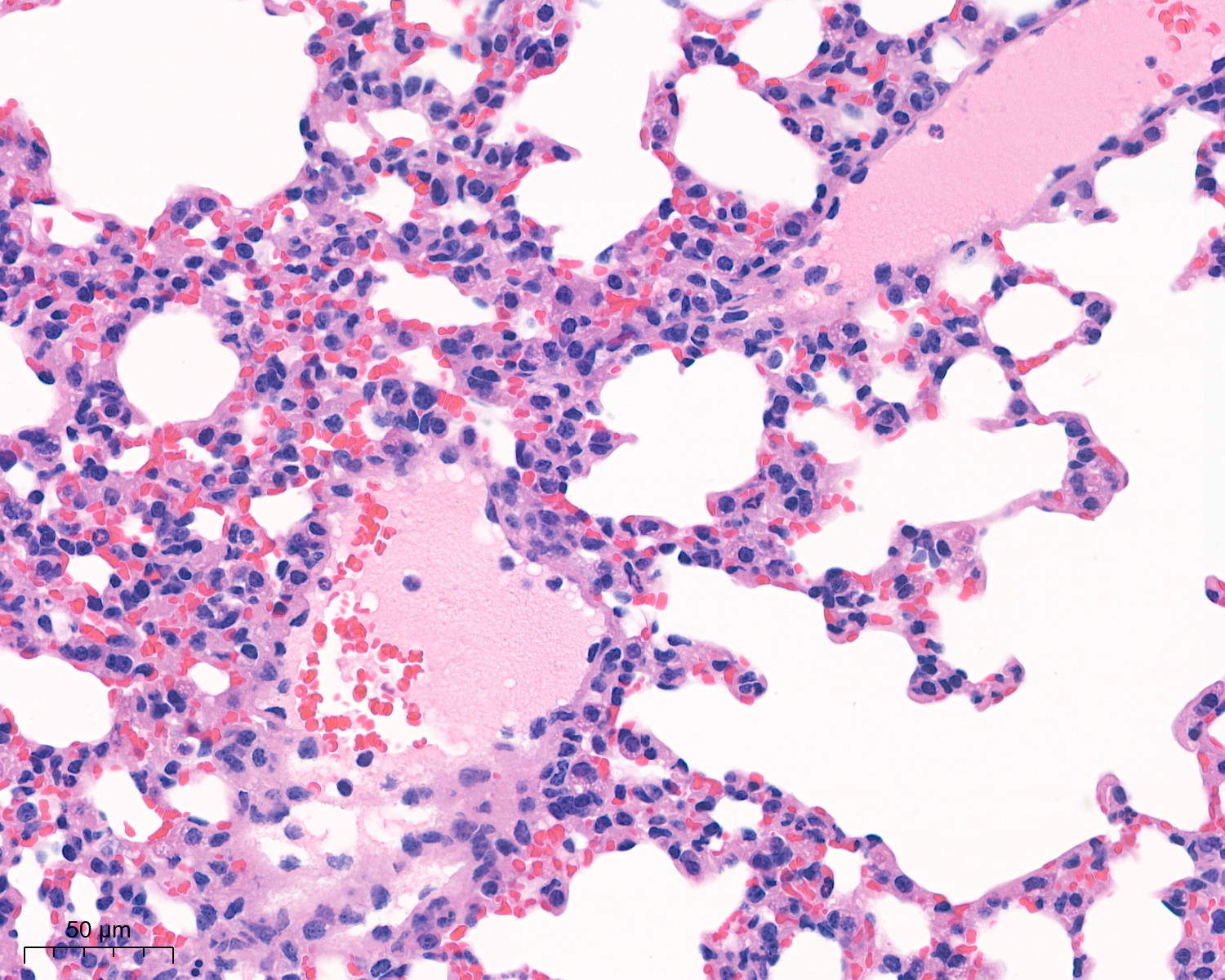

Supplement: Supplementary file 2 — Source Data Fig. 1 [file 44319_2023_47_MOESM2_ESM.zip › EMBOR-2023-57416V3-Figure_1_Source_Data-sd/Figure 1/E/4 DDX5 WT M. pneumoniae.tif]

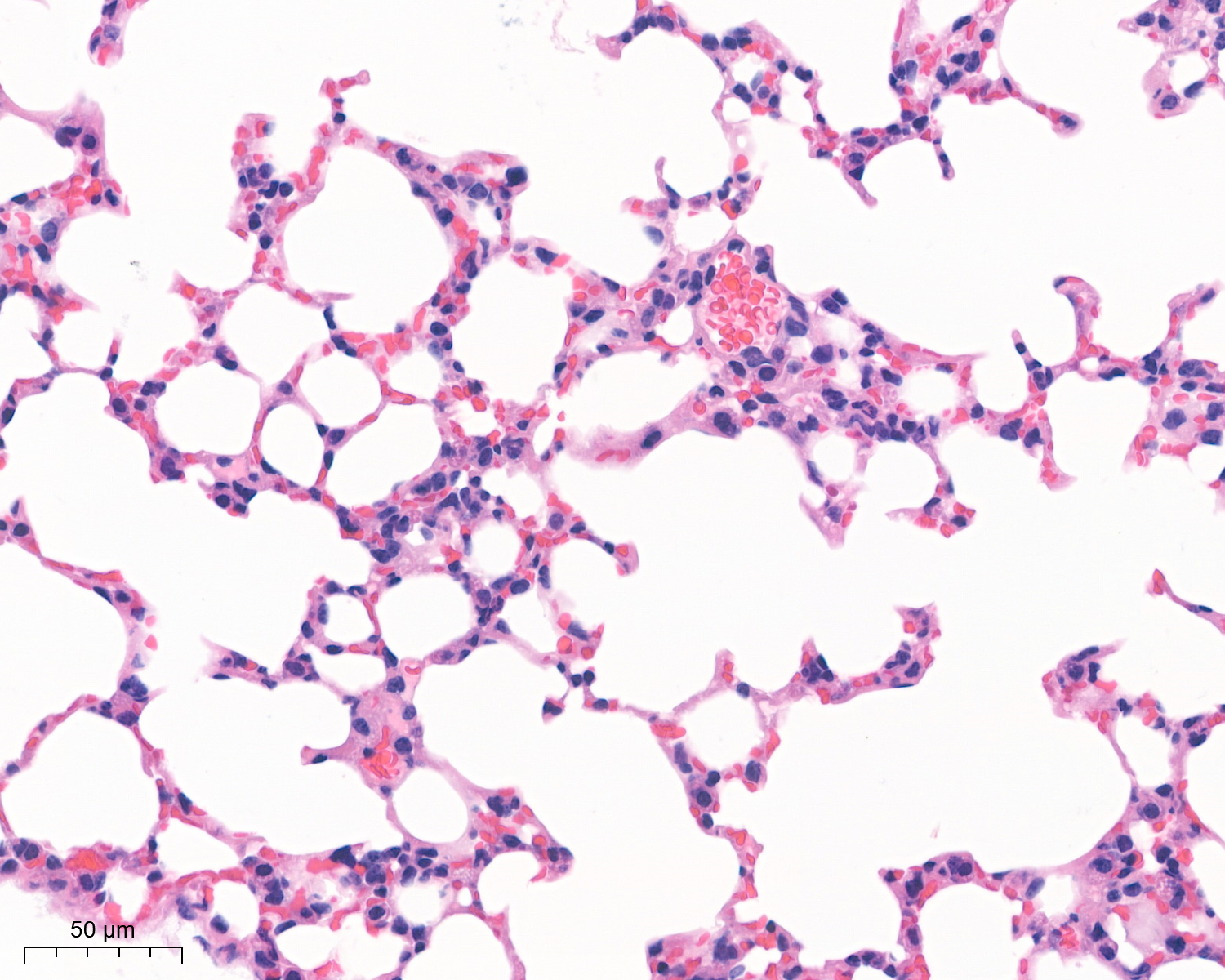

Supplement: Supplementary file 2 — Source Data Fig. 1 [file 44319_2023_47_MOESM2_ESM.zip › EMBOR-2023-57416V3-Figure_1_Source_Data-sd/Figure 1/E/5 DDX5 KO Mock.tif]

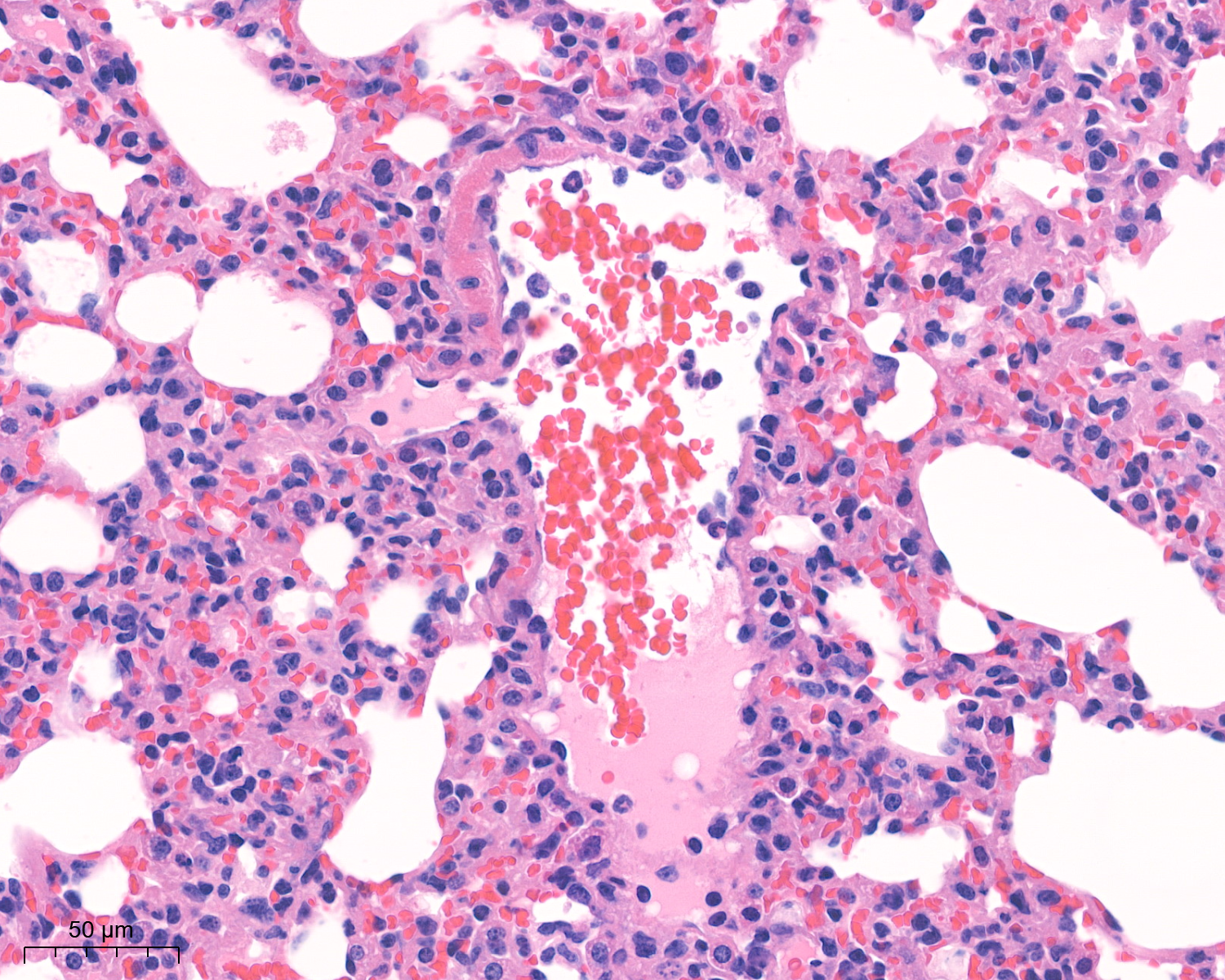

Supplement: Supplementary file 2 — Source Data Fig. 1 [file 44319_2023_47_MOESM2_ESM.zip › EMBOR-2023-57416V3-Figure_1_Source_Data-sd/Figure 1/E/6 DDX5 KO P. multocida.tif]

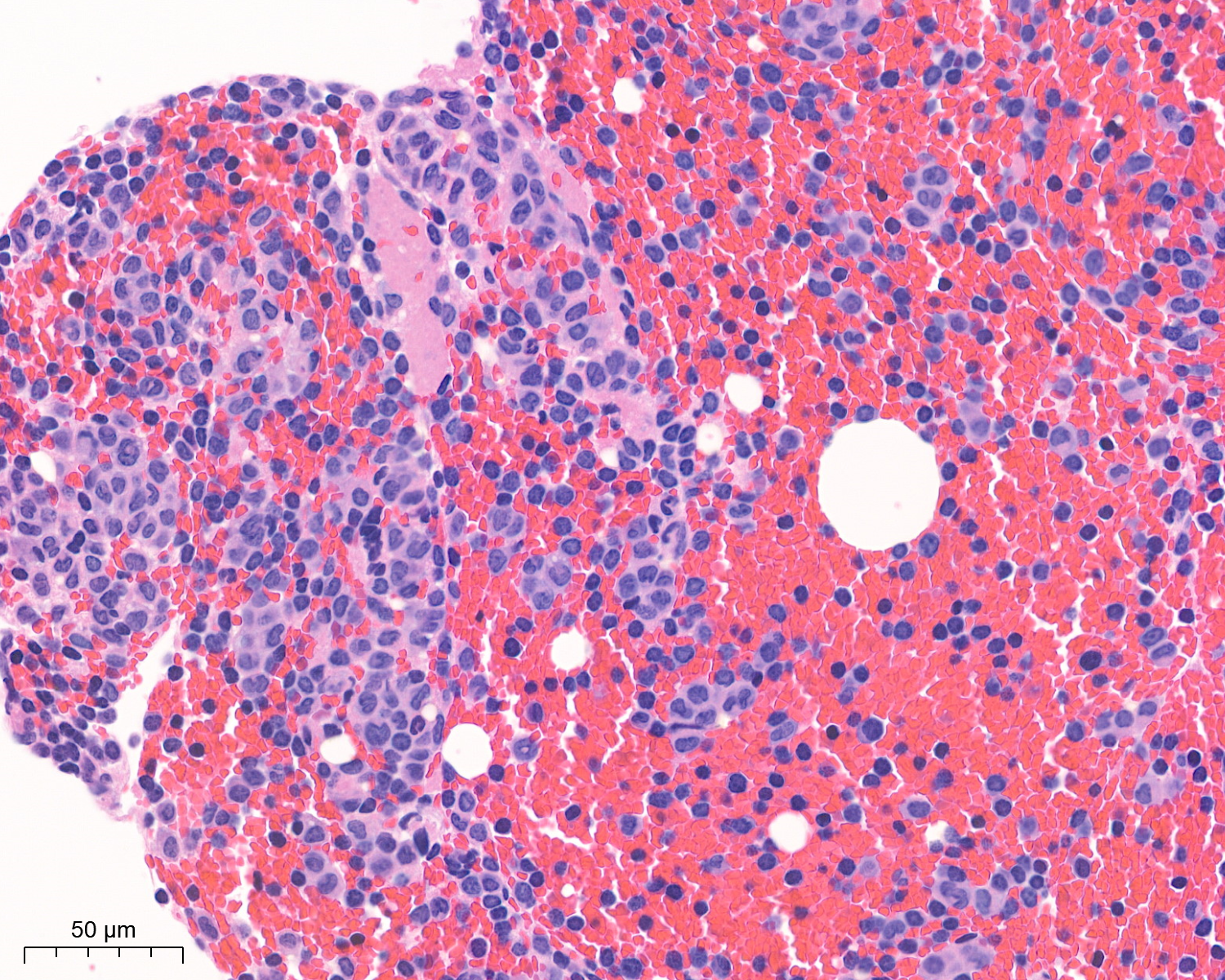

Supplement: Supplementary file 2 — Source Data Fig. 1 [file 44319_2023_47_MOESM2_ESM.zip › EMBOR-2023-57416V3-Figure_1_Source_Data-sd/Figure 1/E/7 DDX5 KO S. aureus.tif]

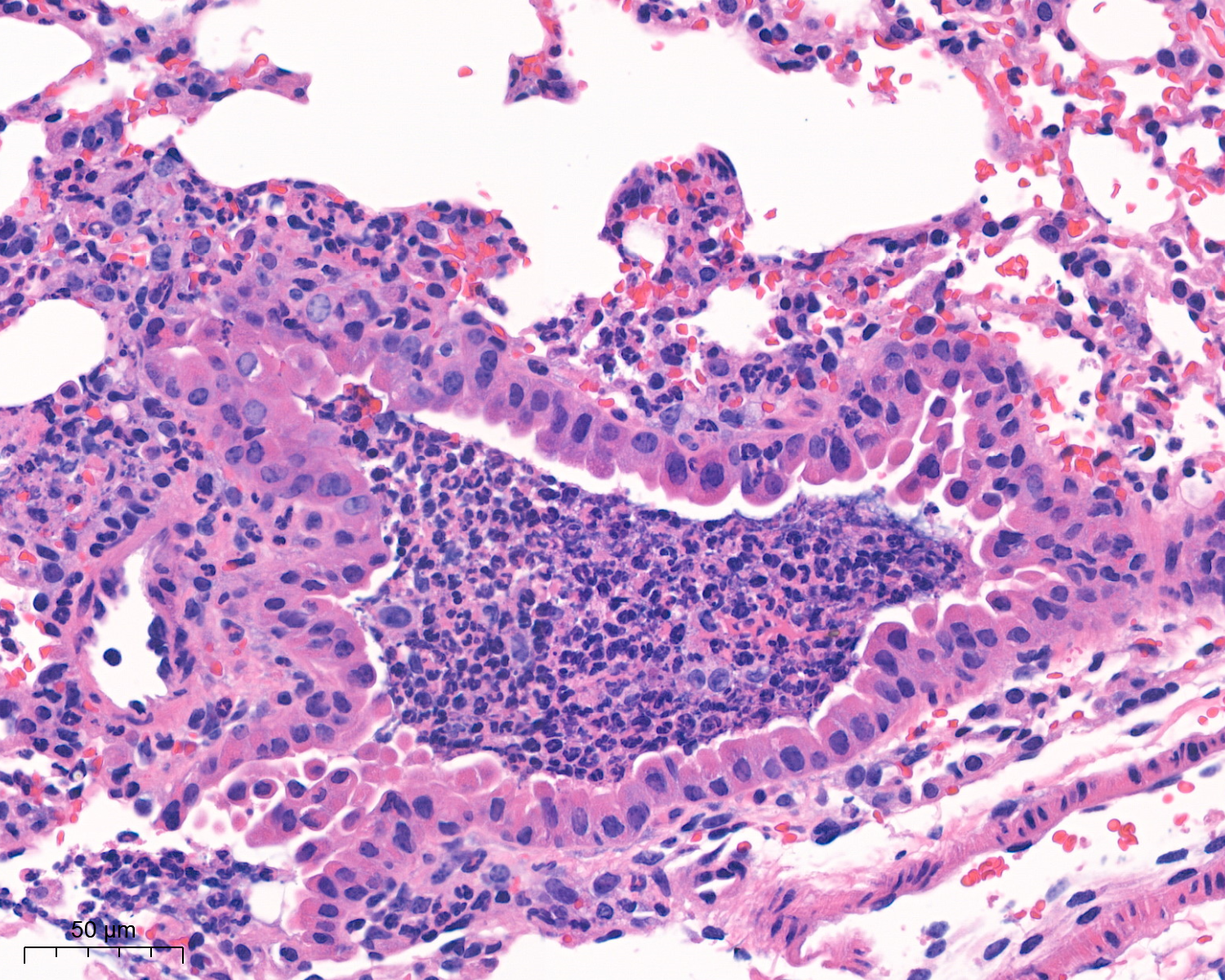

Supplement: Supplementary file 2 — Source Data Fig. 1 [file 44319_2023_47_MOESM2_ESM.zip › EMBOR-2023-57416V3-Figure_1_Source_Data-sd/Figure 1/E/8 DDX5 KO M. pneumoniae.tif]

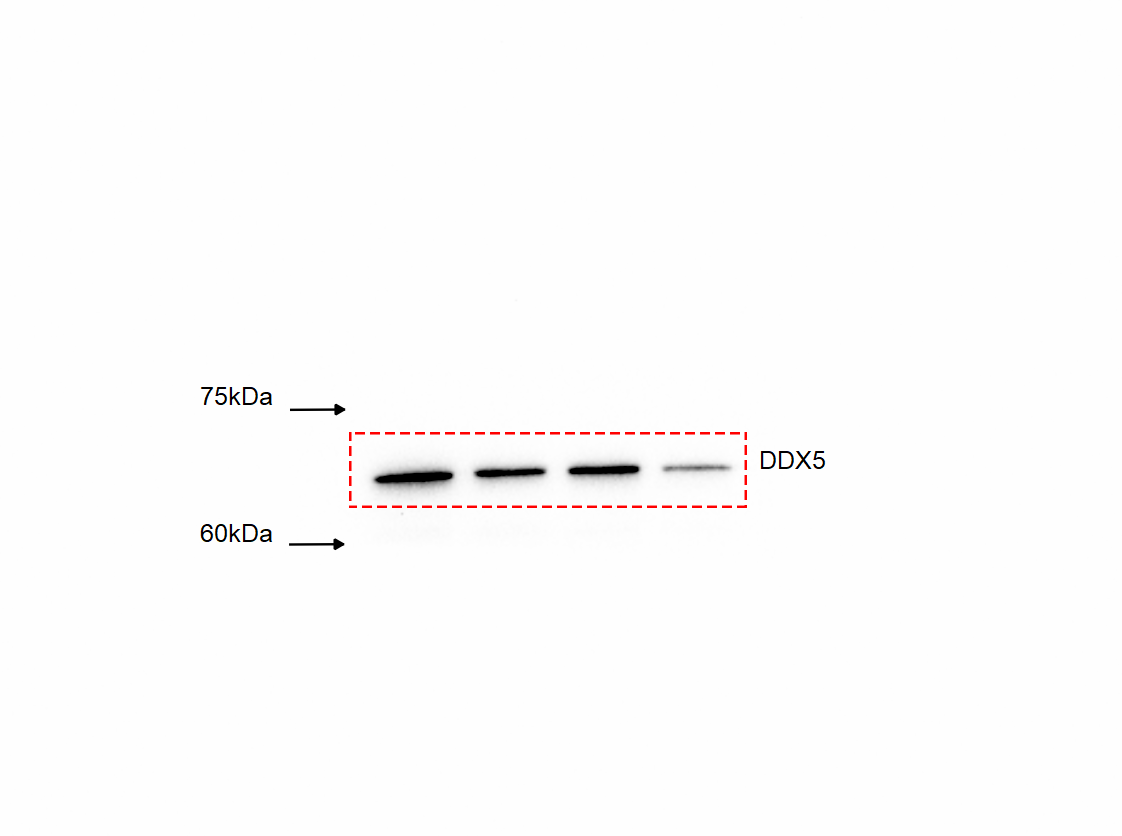

Supplement: Supplementary file 3 — Source Data Fig. 2 [file 44319_2023_47_MOESM3_ESM.zip › EMBOR-2023-57416V3-Figure_2_Source_Data-sd/Figure 2/A/MP-DDX5.tif]

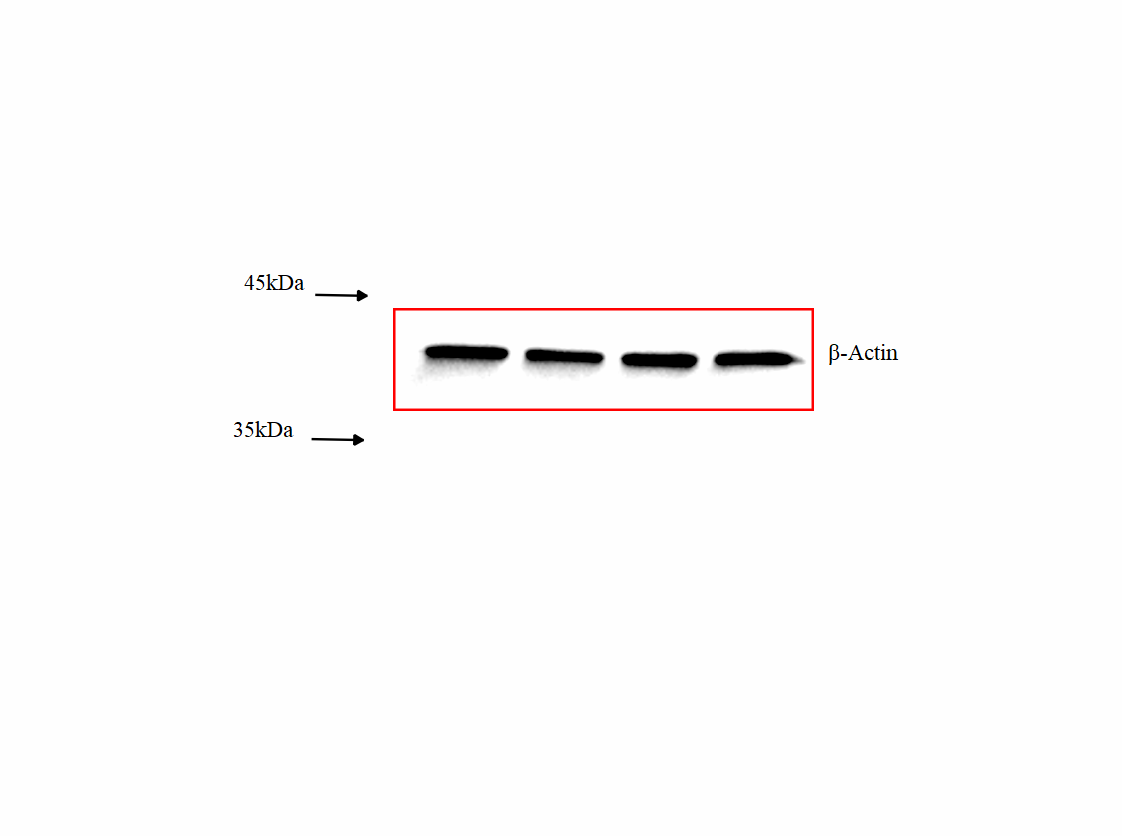

Supplement: Supplementary file 3 — Source Data Fig. 2 [file 44319_2023_47_MOESM3_ESM.zip › EMBOR-2023-57416V3-Figure_2_Source_Data-sd/Figure 2/A/MP-β-Actin.tif]

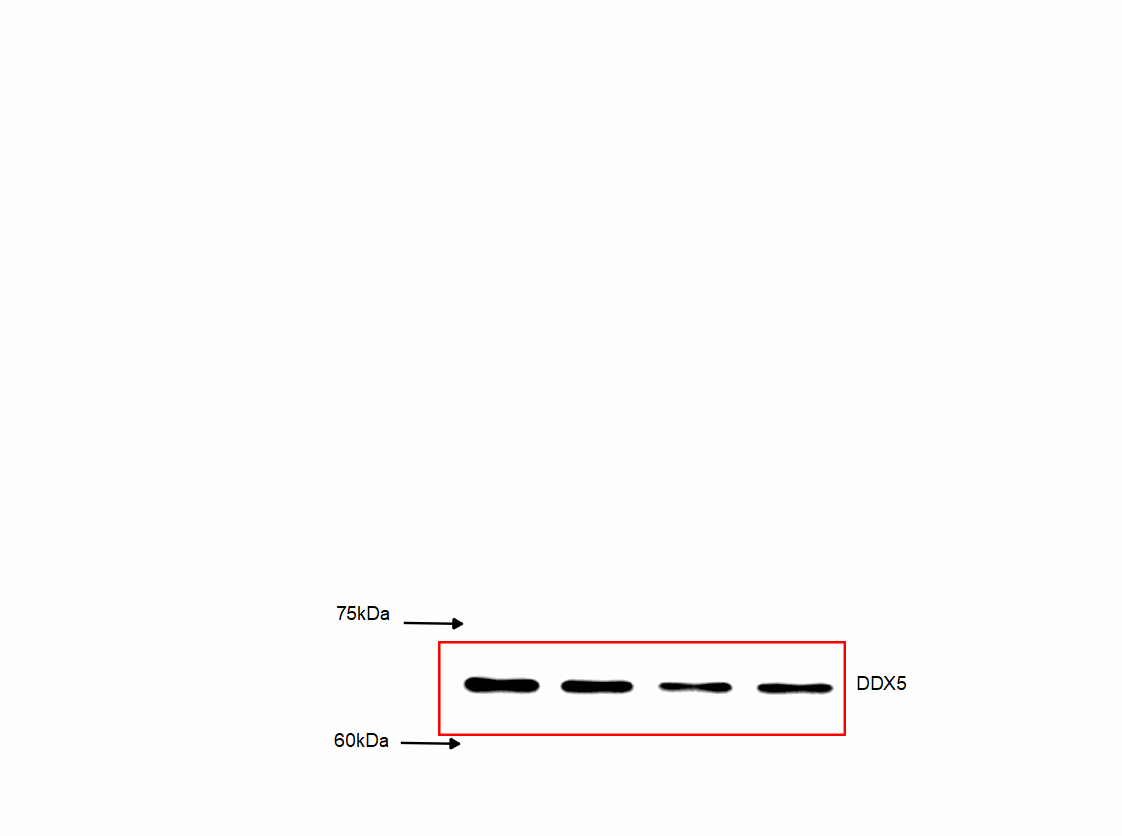

Supplement: Supplementary file 3 — Source Data Fig. 2 [file 44319_2023_47_MOESM3_ESM.zip › EMBOR-2023-57416V3-Figure_2_Source_Data-sd/Figure 2/A/PM-DDX5.tif]

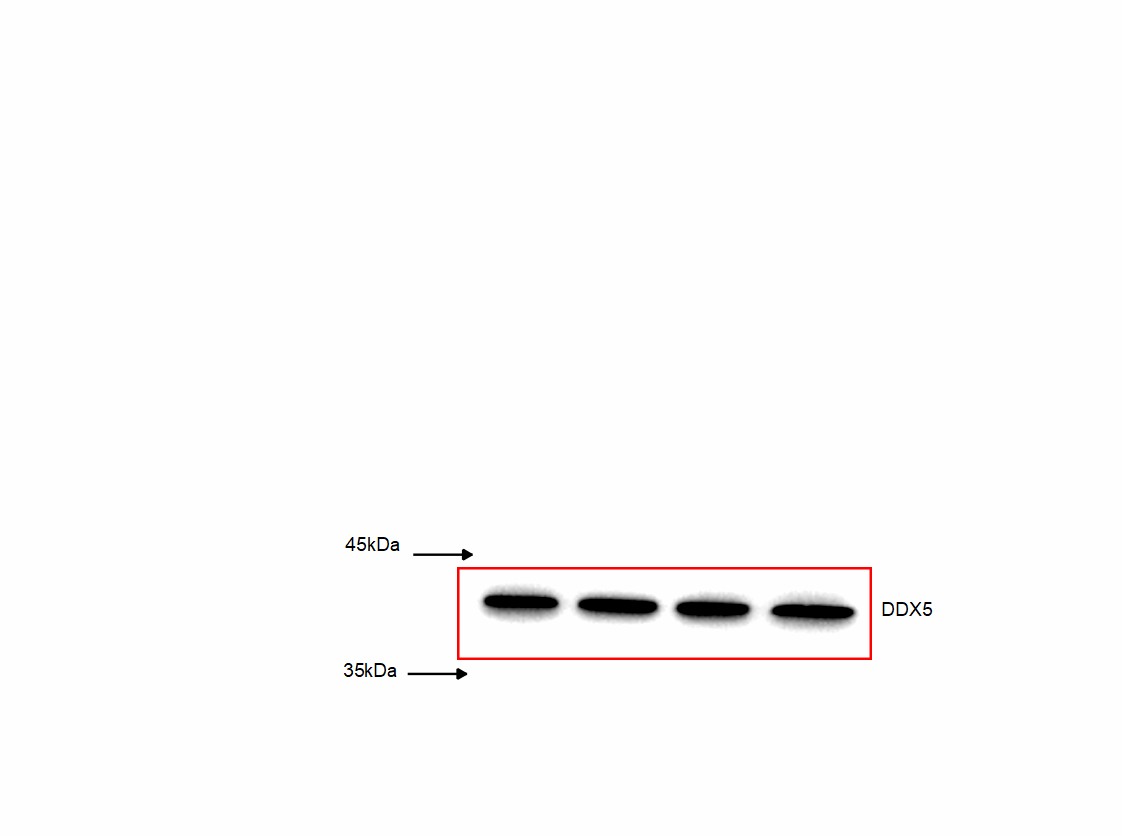

Supplement: Supplementary file 3 — Source Data Fig. 2 [file 44319_2023_47_MOESM3_ESM.zip › EMBOR-2023-57416V3-Figure_2_Source_Data-sd/Figure 2/A/PM-β-Actin.tif]

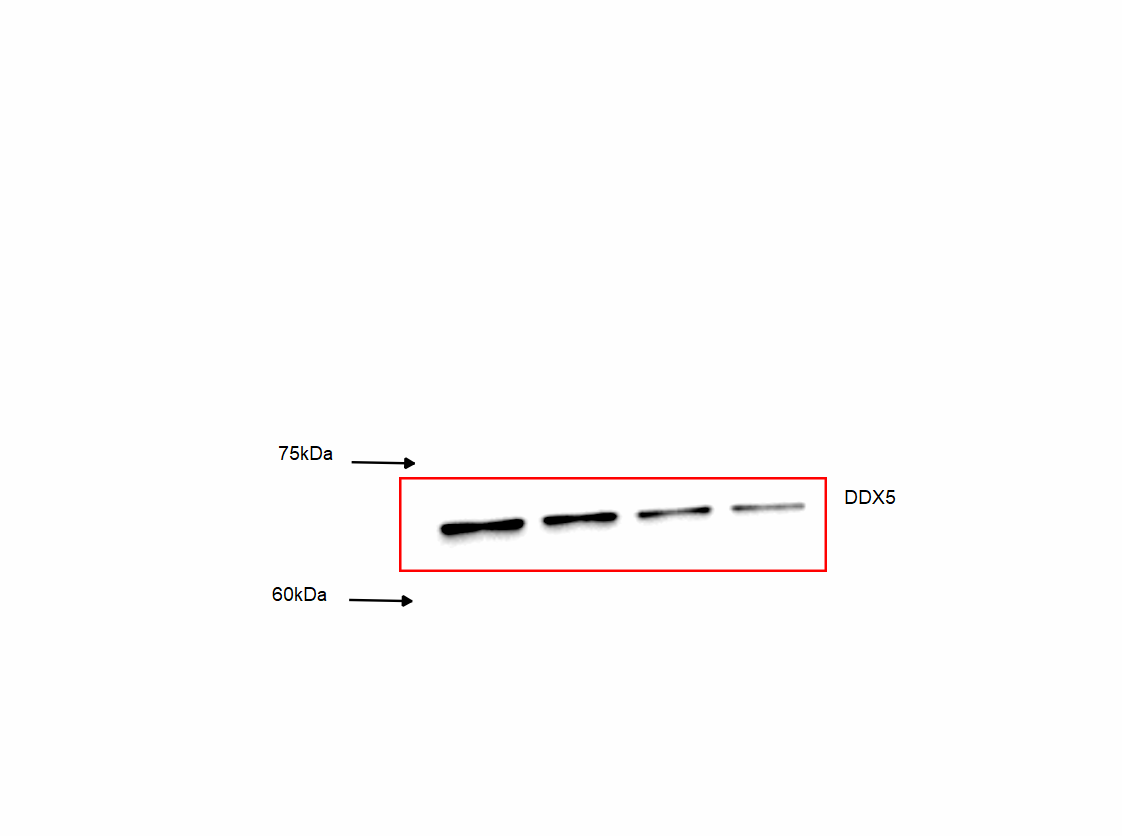

Supplement: Supplementary file 3 — Source Data Fig. 2 [file 44319_2023_47_MOESM3_ESM.zip › EMBOR-2023-57416V3-Figure_2_Source_Data-sd/Figure 2/A/SA-DDX5.tif]

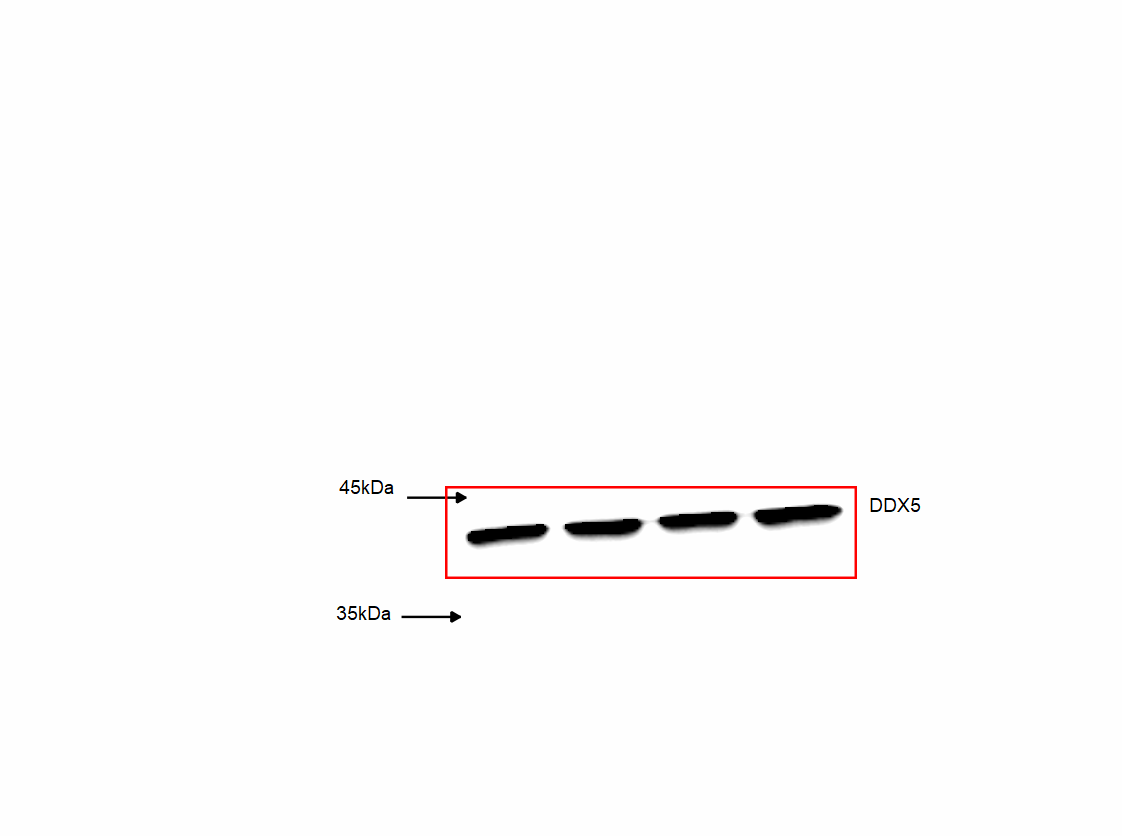

Supplement: Supplementary file 3 — Source Data Fig. 2 [file 44319_2023_47_MOESM3_ESM.zip › EMBOR-2023-57416V3-Figure_2_Source_Data-sd/Figure 2/A/SA-β-Actin.tif]

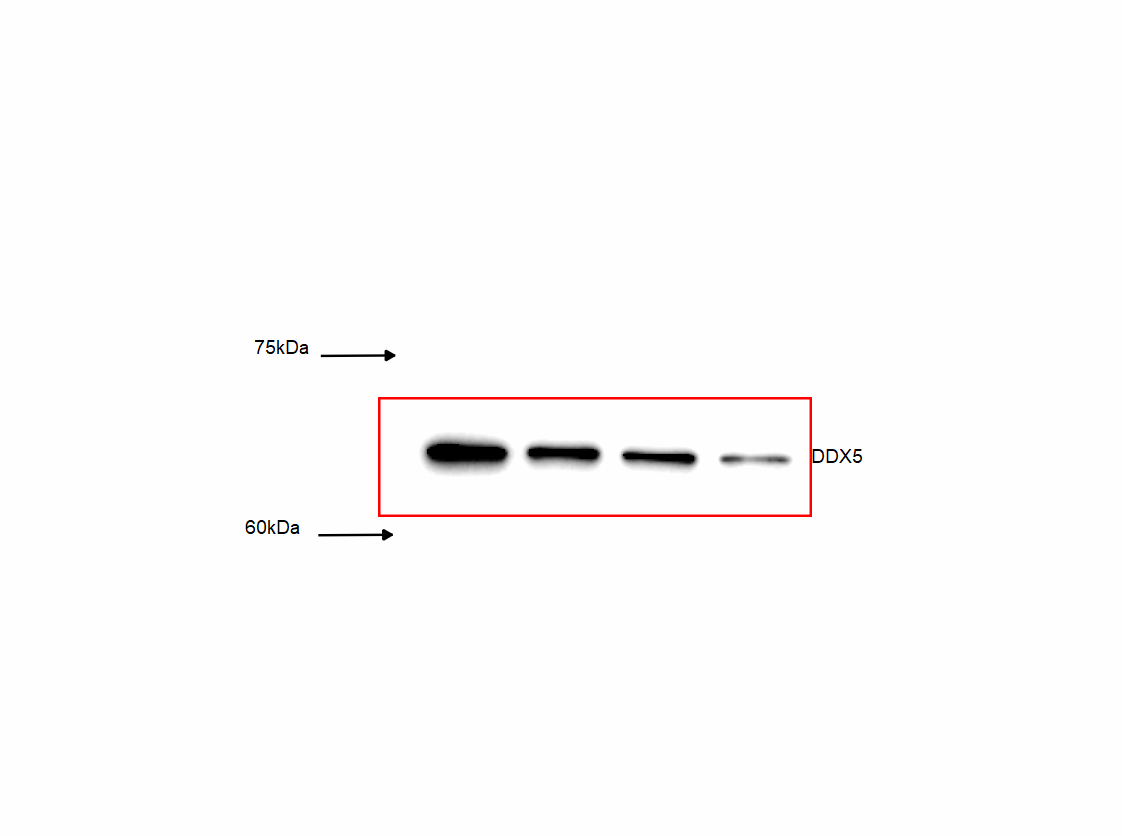

Supplement: Supplementary file 3 — Source Data Fig. 2 [file 44319_2023_47_MOESM3_ESM.zip › EMBOR-2023-57416V3-Figure_2_Source_Data-sd/Figure 2/B/FSL-1-DDX5.tif]

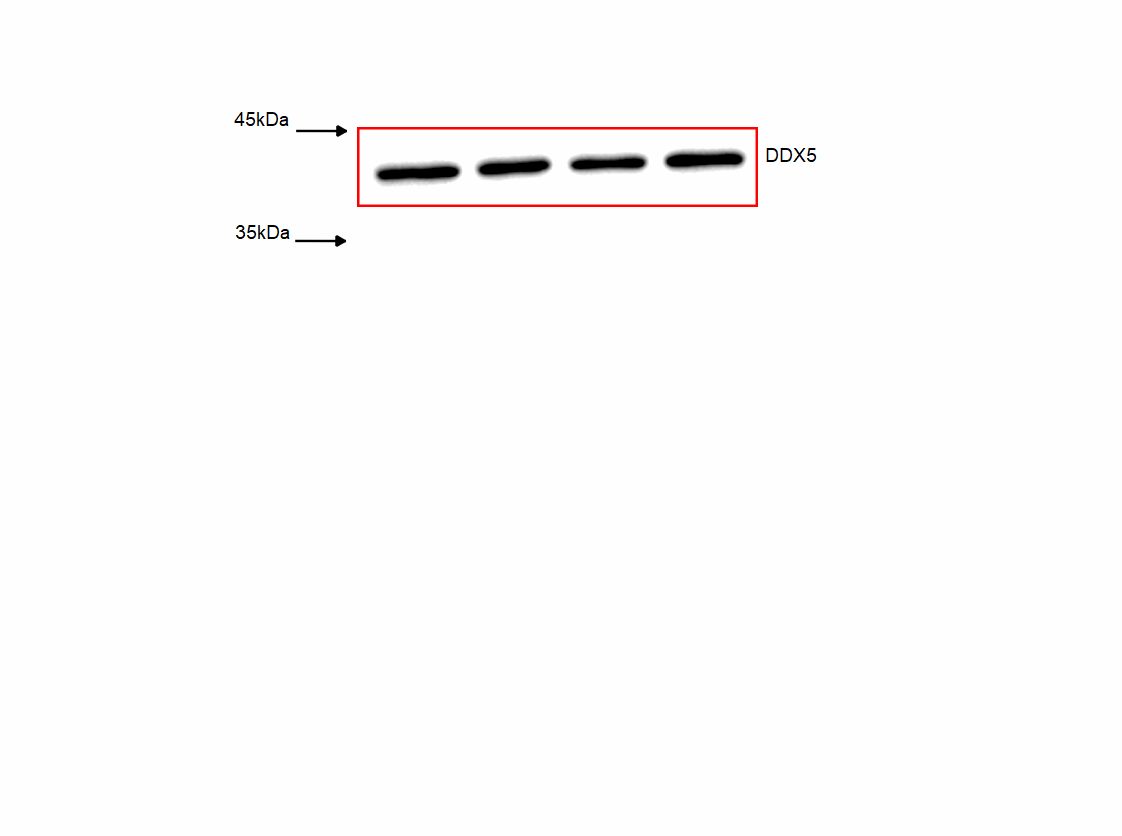

Supplement: Supplementary file 3 — Source Data Fig. 2 [file 44319_2023_47_MOESM3_ESM.zip › EMBOR-2023-57416V3-Figure_2_Source_Data-sd/Figure 2/B/FSL-1-β-Actin.tif]

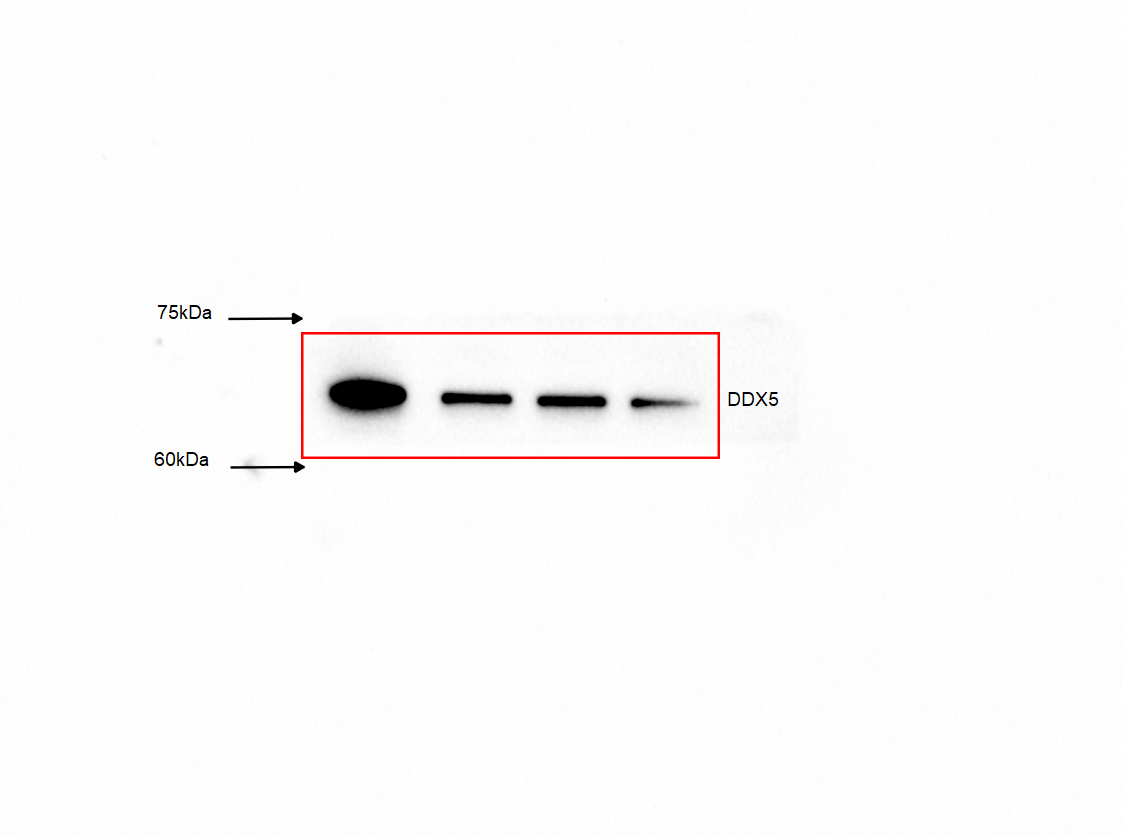

Supplement: Supplementary file 3 — Source Data Fig. 2 [file 44319_2023_47_MOESM3_ESM.zip › EMBOR-2023-57416V3-Figure_2_Source_Data-sd/Figure 2/B/LPS-DDX5.tif]

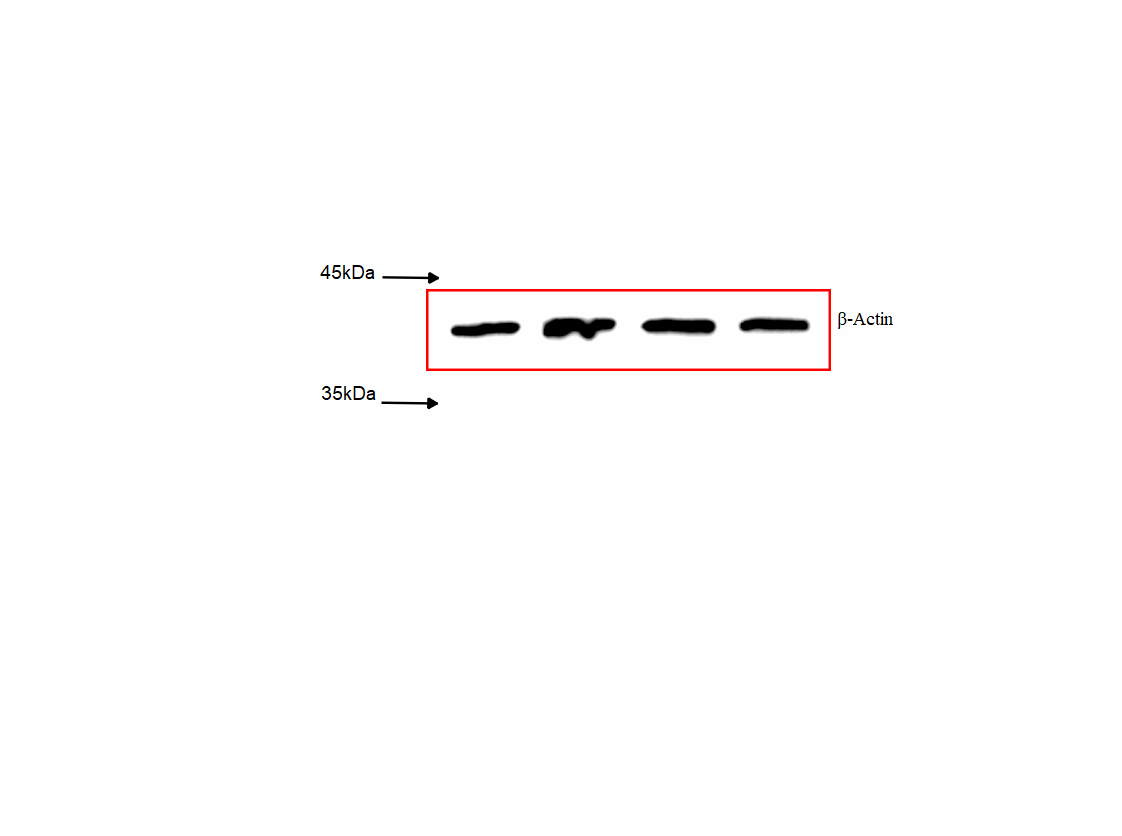

Supplement: Supplementary file 3 — Source Data Fig. 2 [file 44319_2023_47_MOESM3_ESM.zip › EMBOR-2023-57416V3-Figure_2_Source_Data-sd/Figure 2/B/LPS-β-Actin.tif]

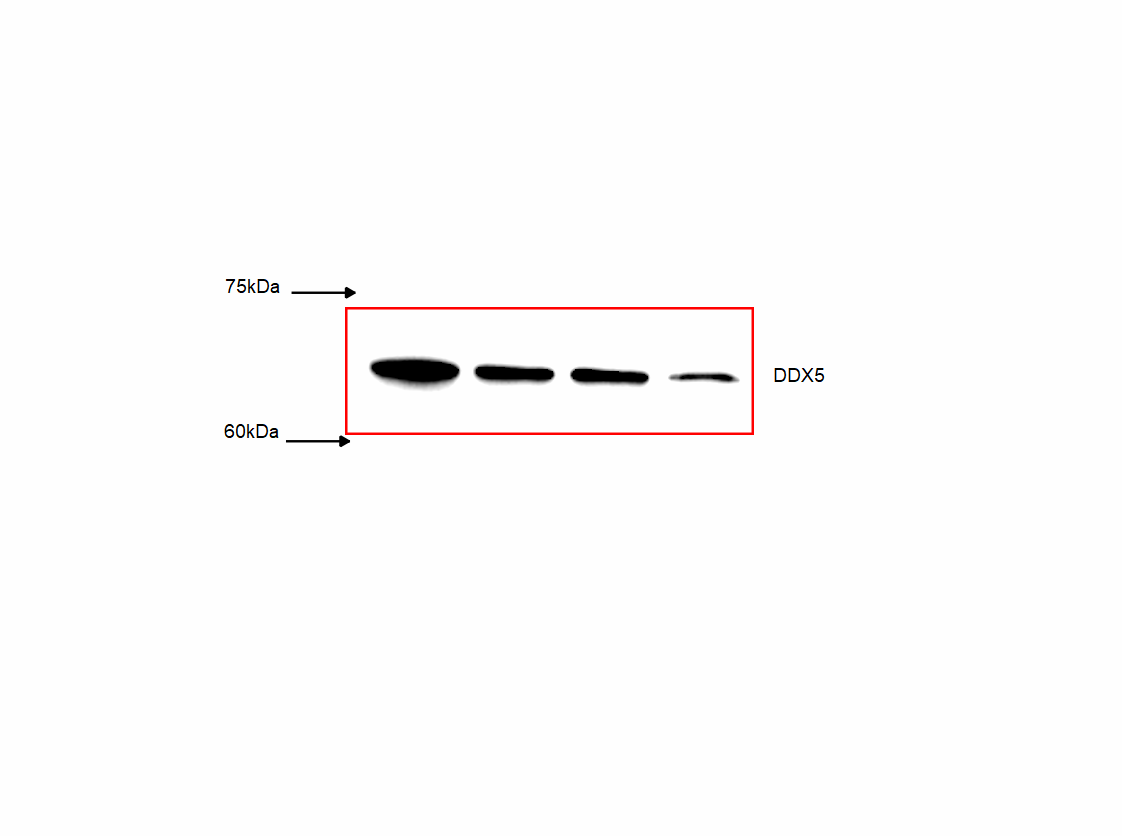

Supplement: Supplementary file 3 — Source Data Fig. 2 [file 44319_2023_47_MOESM3_ESM.zip › EMBOR-2023-57416V3-Figure_2_Source_Data-sd/Figure 2/B/Pam3CSK4-DDX5.tif]

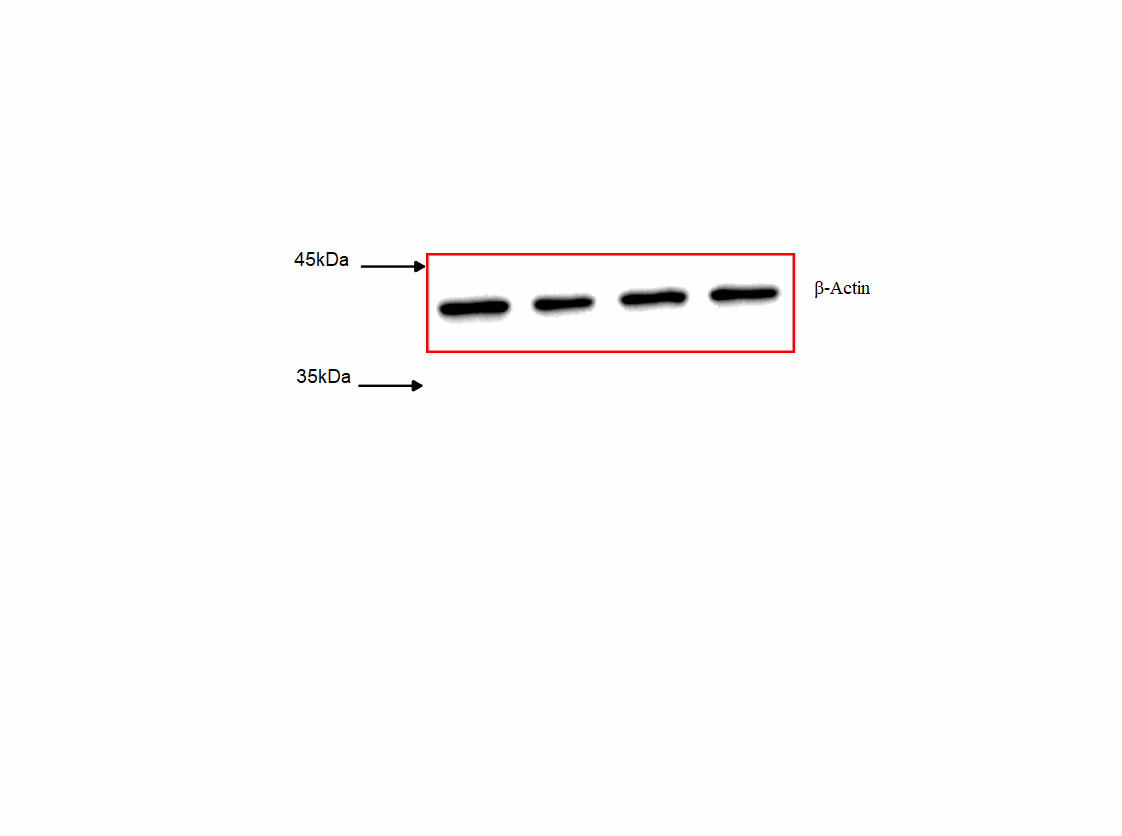

Supplement: Supplementary file 3 — Source Data Fig. 2 [file 44319_2023_47_MOESM3_ESM.zip › EMBOR-2023-57416V3-Figure_2_Source_Data-sd/Figure 2/B/Pam3CSK4-β-Actin.tif]

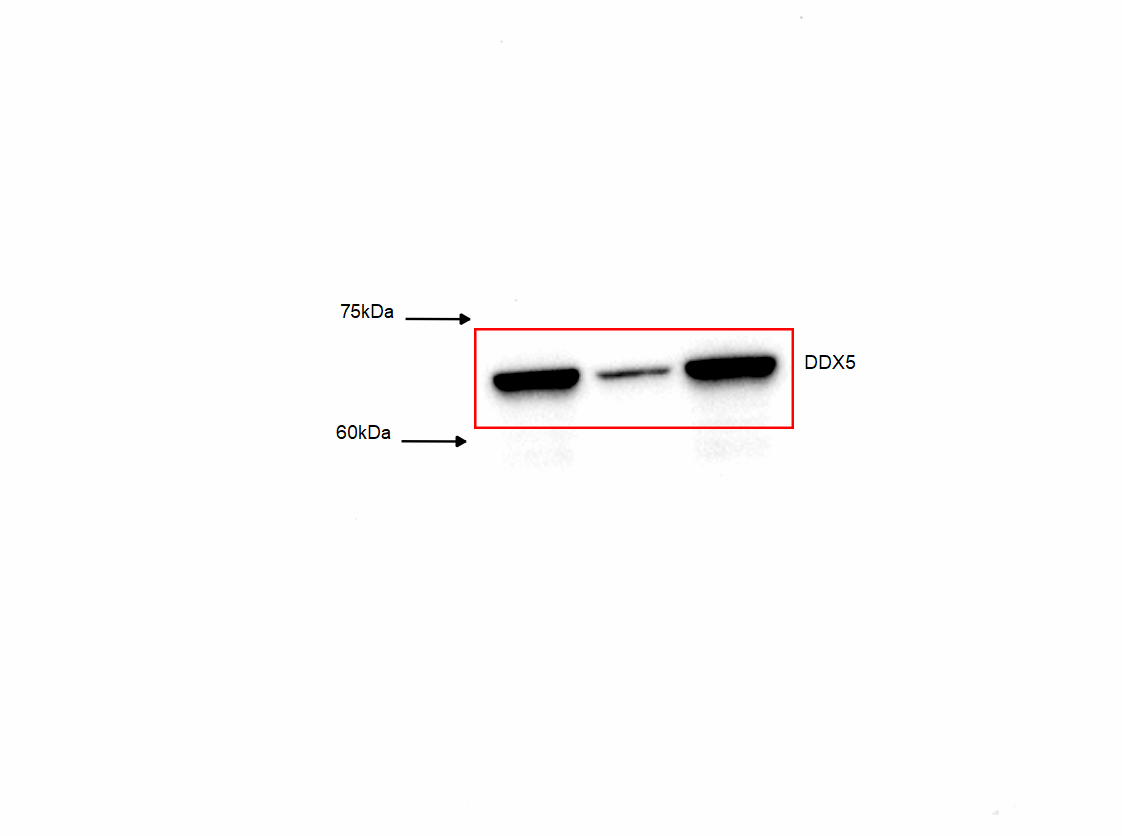

Supplement: Supplementary file 3 — Source Data Fig. 2 [file 44319_2023_47_MOESM3_ESM.zip › EMBOR-2023-57416V3-Figure_2_Source_Data-sd/Figure 2/C/FSL-1-DDX5.tif]

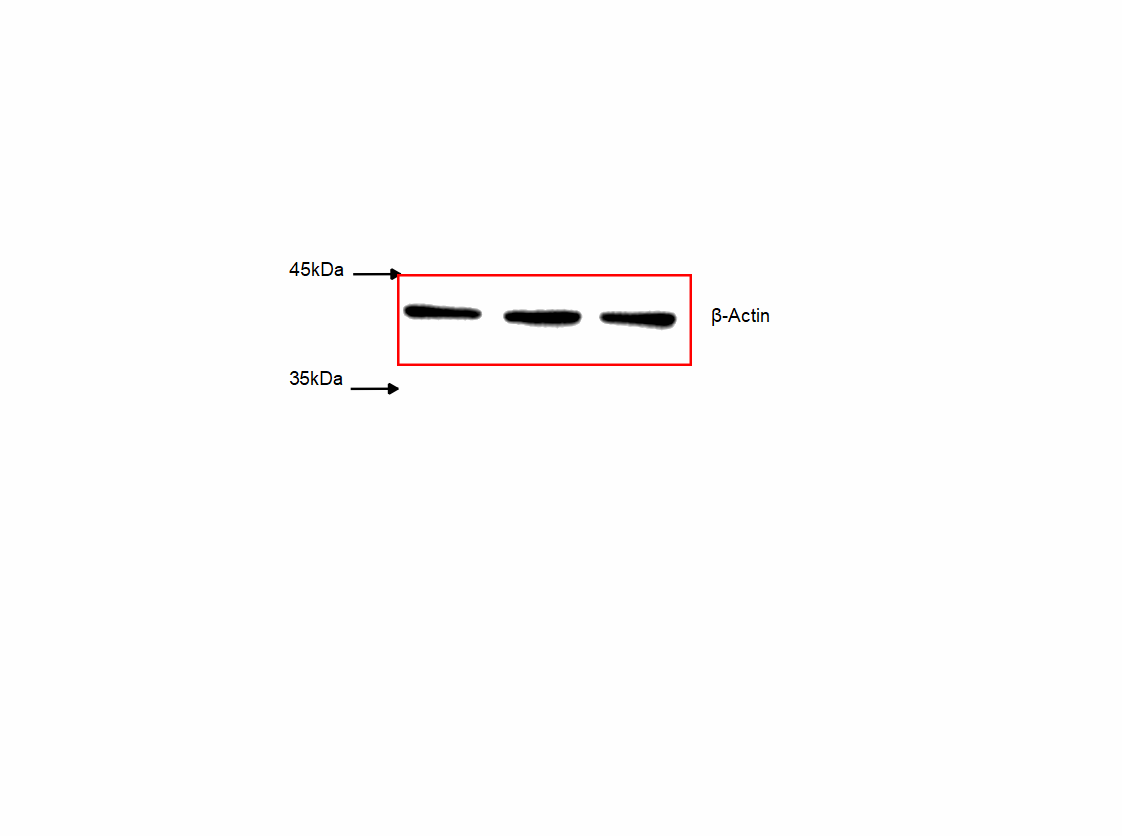

Supplement: Supplementary file 3 — Source Data Fig. 2 [file 44319_2023_47_MOESM3_ESM.zip › EMBOR-2023-57416V3-Figure_2_Source_Data-sd/Figure 2/C/FSL-1-β-Actin.tif]

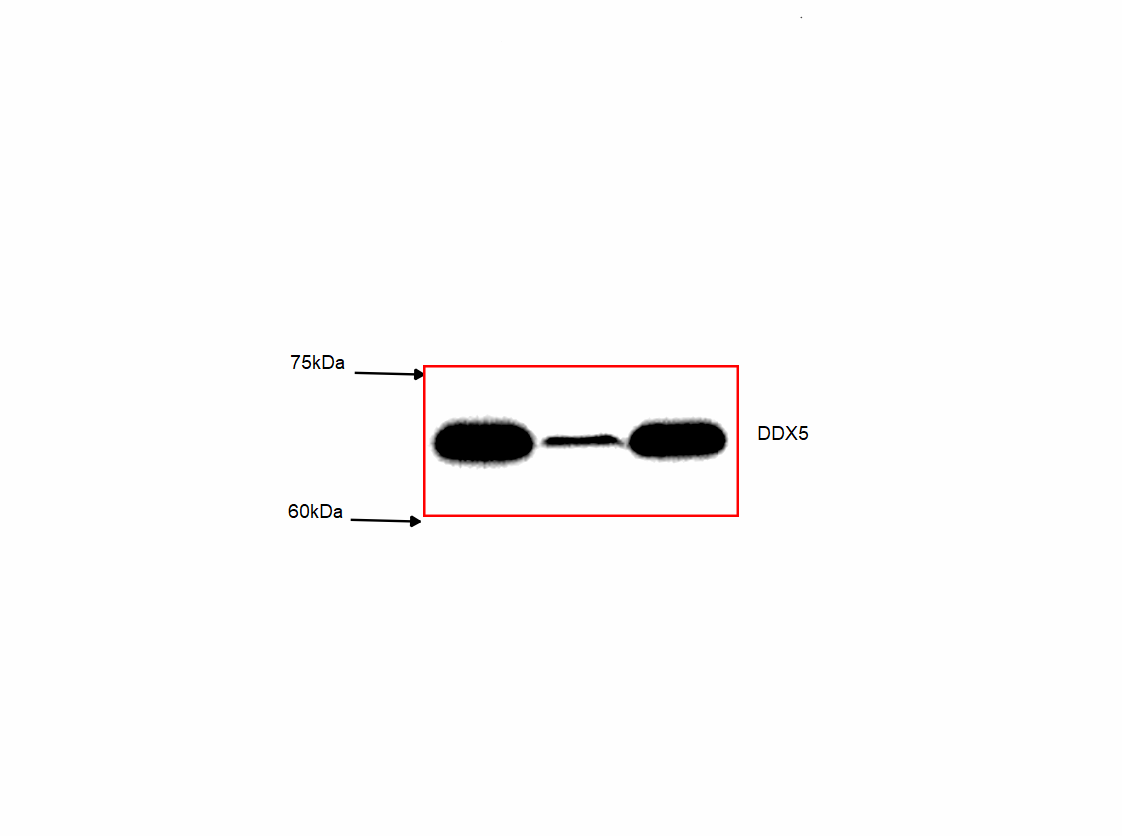

Supplement: Supplementary file 3 — Source Data Fig. 2 [file 44319_2023_47_MOESM3_ESM.zip › EMBOR-2023-57416V3-Figure_2_Source_Data-sd/Figure 2/C/LPS-DDX5.tif]

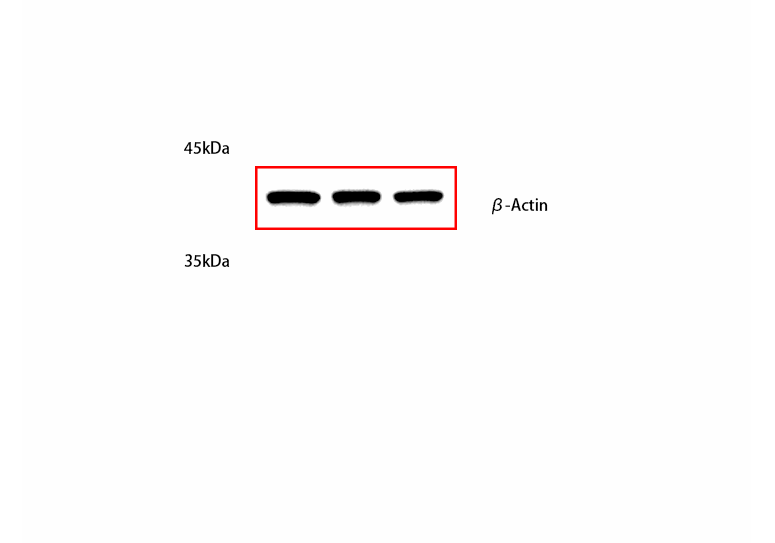

Supplement: Supplementary file 3 — Source Data Fig. 2 [file 44319_2023_47_MOESM3_ESM.zip › EMBOR-2023-57416V3-Figure_2_Source_Data-sd/Figure 2/C/LPS-β-Actin.tif]

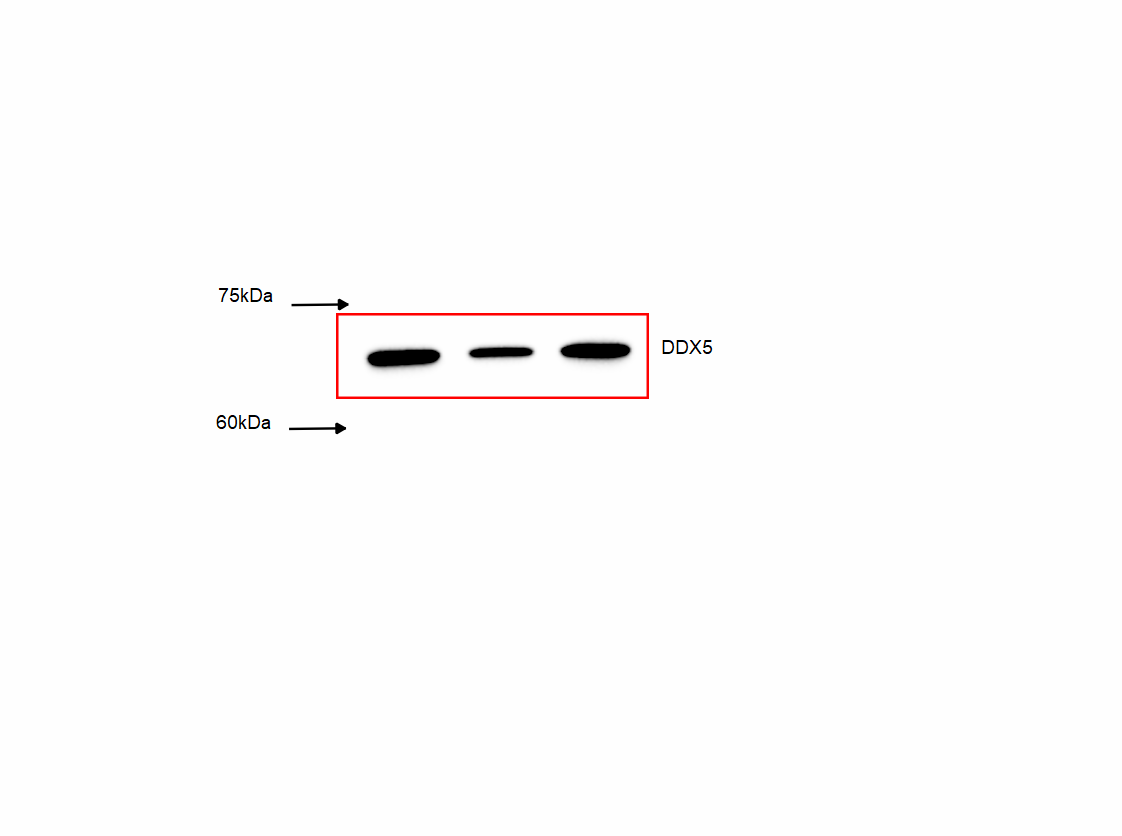

Supplement: Supplementary file 3 — Source Data Fig. 2 [file 44319_2023_47_MOESM3_ESM.zip › EMBOR-2023-57416V3-Figure_2_Source_Data-sd/Figure 2/C/Pam3CSK4-DDX5.tif]

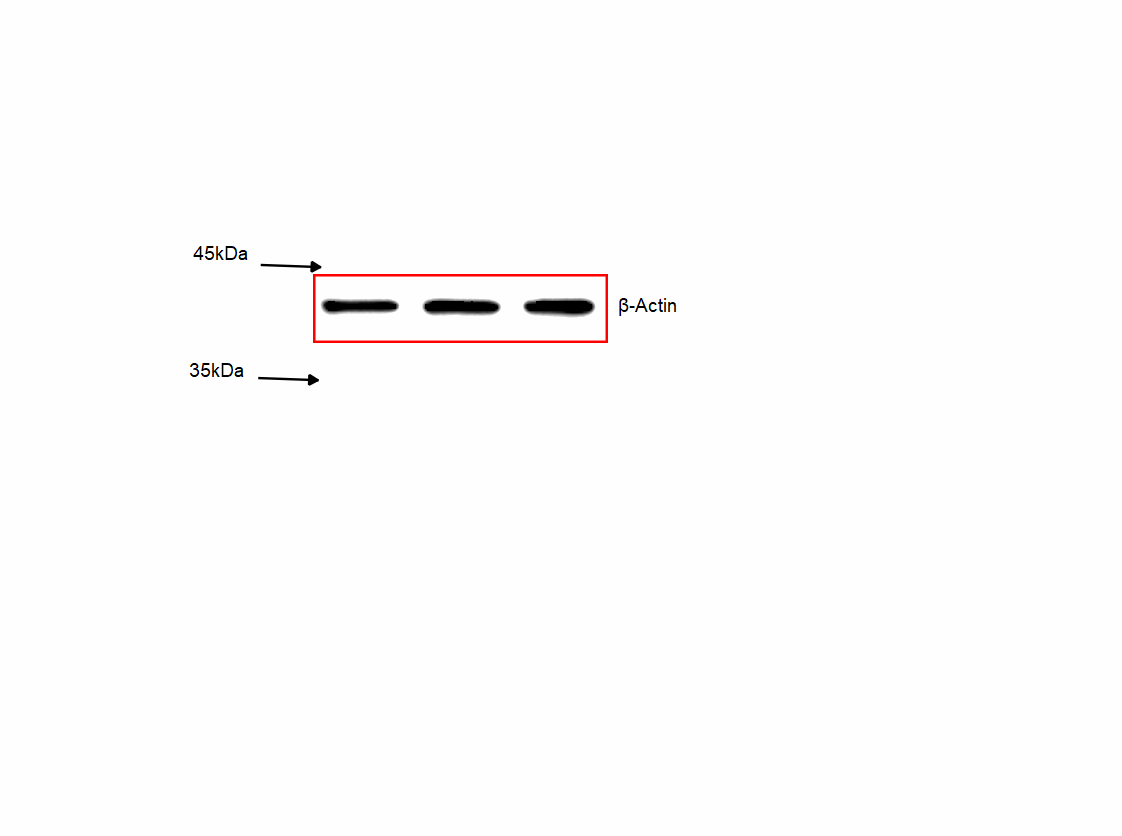

Supplement: Supplementary file 3 — Source Data Fig. 2 [file 44319_2023_47_MOESM3_ESM.zip › EMBOR-2023-57416V3-Figure_2_Source_Data-sd/Figure 2/C/Pam3CSK4-β-Actin.tif]

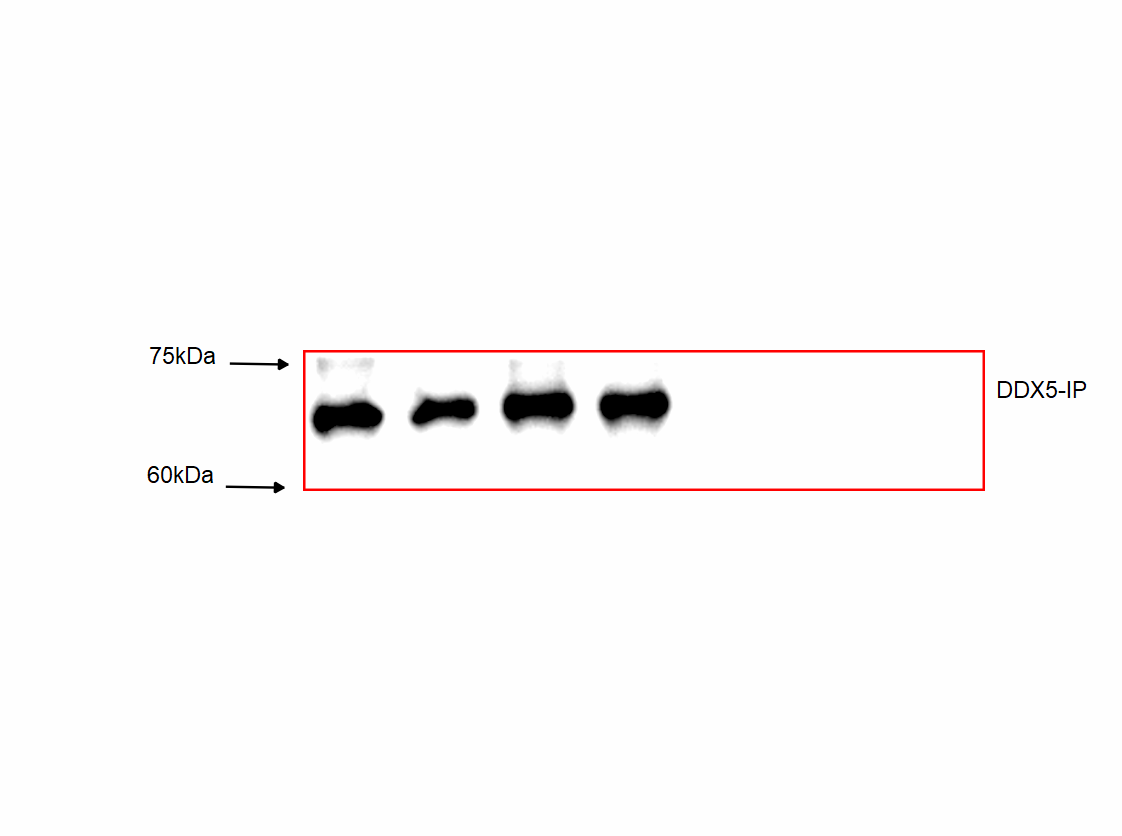

Supplement: Supplementary file 3 — Source Data Fig. 2 [file 44319_2023_47_MOESM3_ESM.zip › EMBOR-2023-57416V3-Figure_2_Source_Data-sd/Figure 2/D/IP-DDX5.tif]

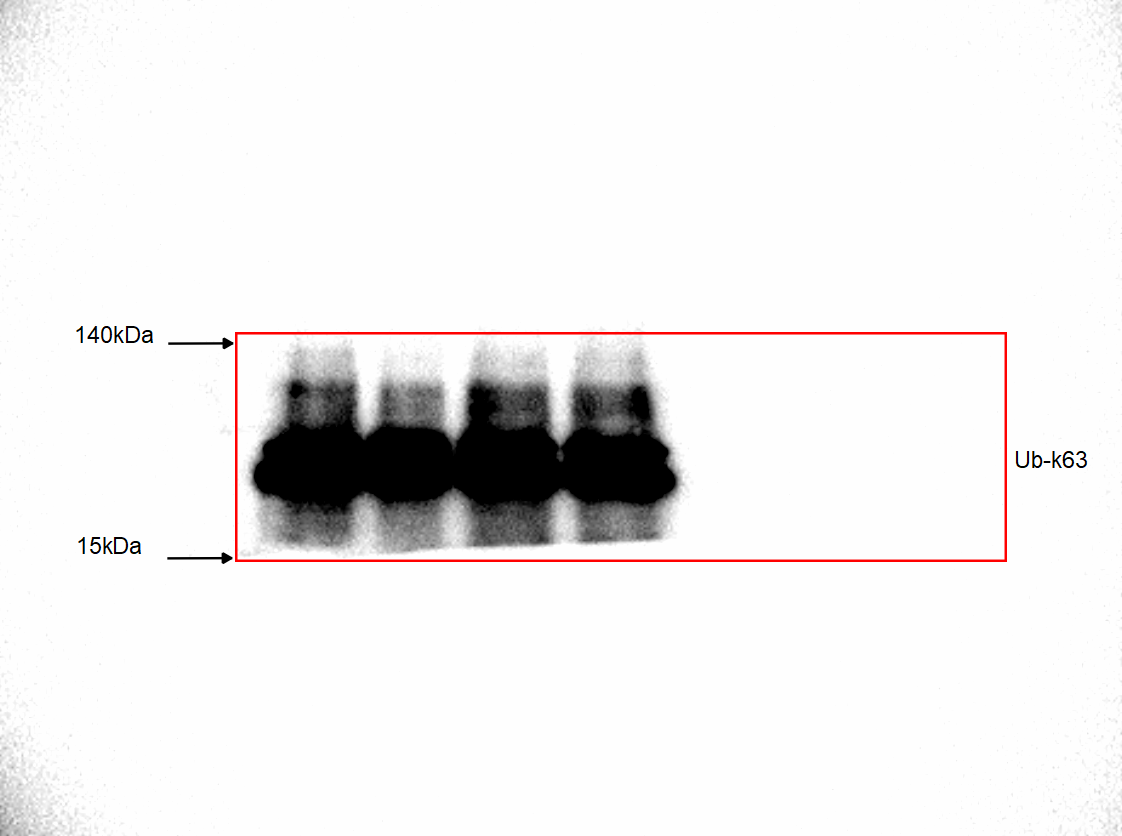

Supplement: Supplementary file 3 — Source Data Fig. 2 [file 44319_2023_47_MOESM3_ESM.zip › EMBOR-2023-57416V3-Figure_2_Source_Data-sd/Figure 2/D/IP-K63.tif]

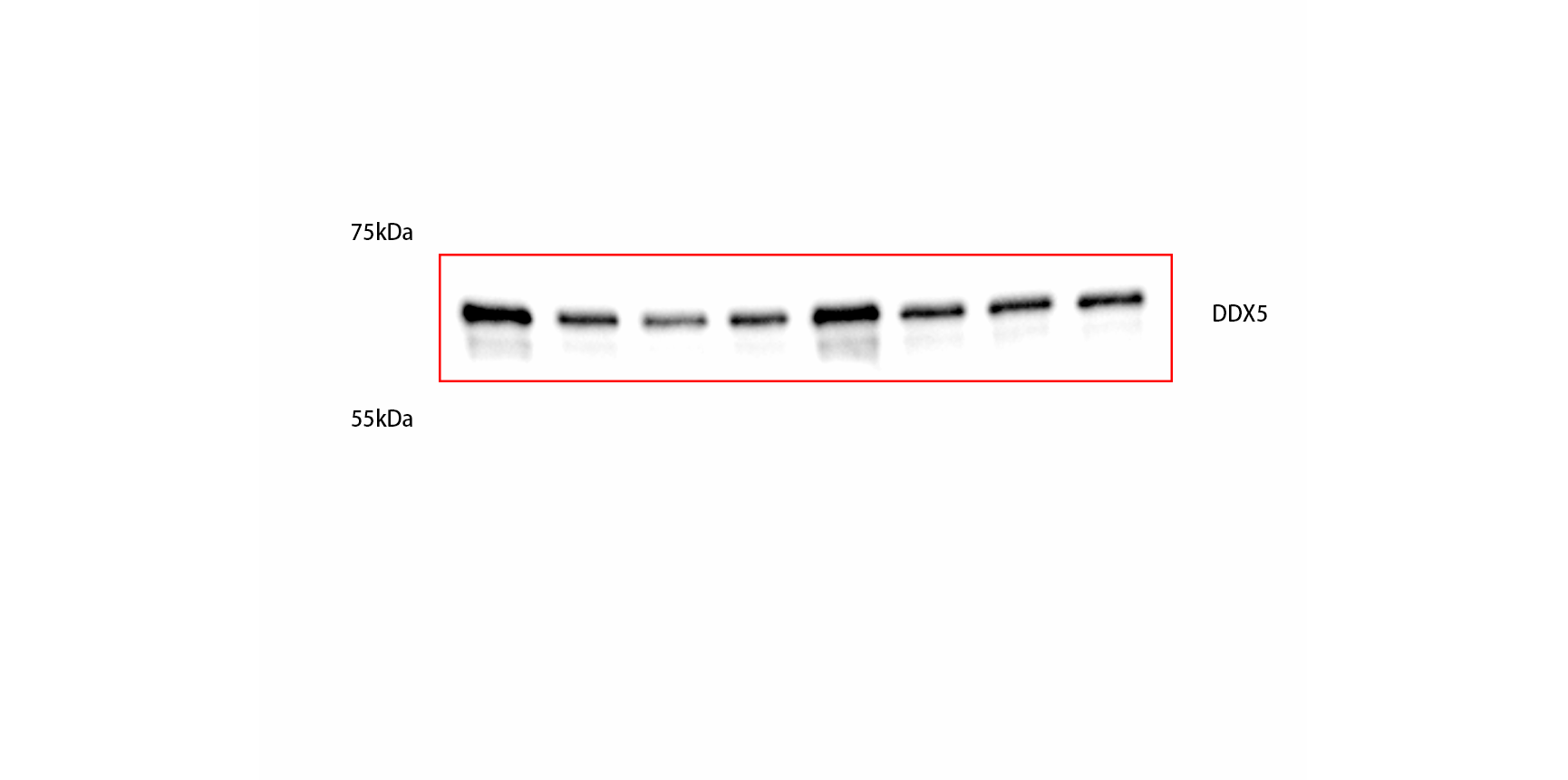

Supplement: Supplementary file 3 — Source Data Fig. 2 [file 44319_2023_47_MOESM3_ESM.zip › EMBOR-2023-57416V3-Figure_2_Source_Data-sd/Figure 2/D/WCL-DDX5.tif]

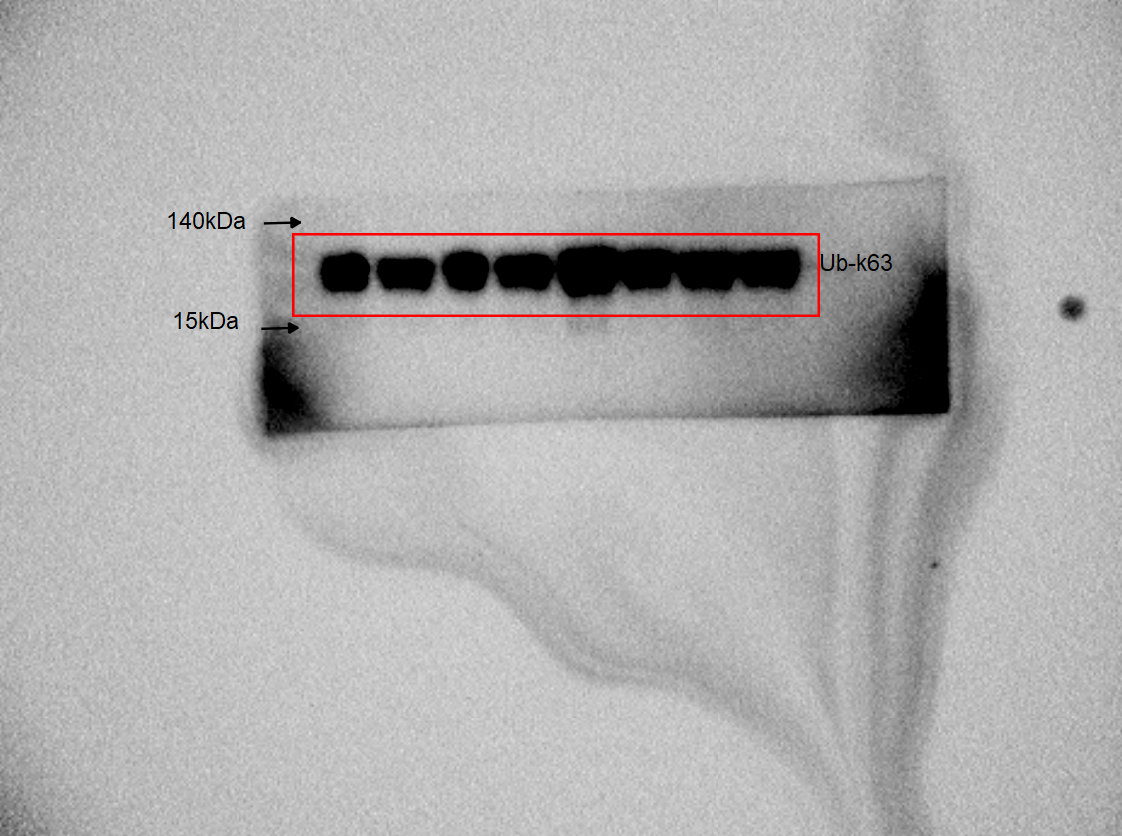

Supplement: Supplementary file 3 — Source Data Fig. 2 [file 44319_2023_47_MOESM3_ESM.zip › EMBOR-2023-57416V3-Figure_2_Source_Data-sd/Figure 2/D/WCL-Ub-k63.tif]

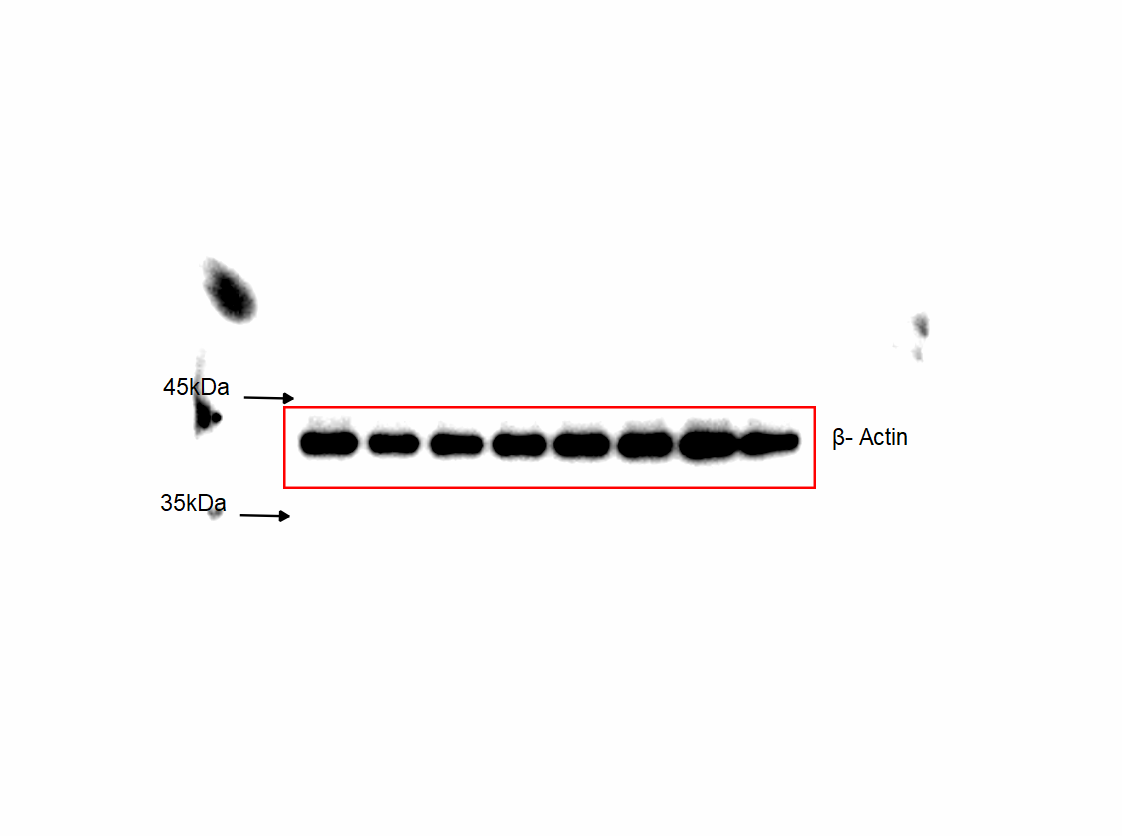

Supplement: Supplementary file 3 — Source Data Fig. 2 [file 44319_2023_47_MOESM3_ESM.zip › EMBOR-2023-57416V3-Figure_2_Source_Data-sd/Figure 2/D/WCL-β-Actin.tif]

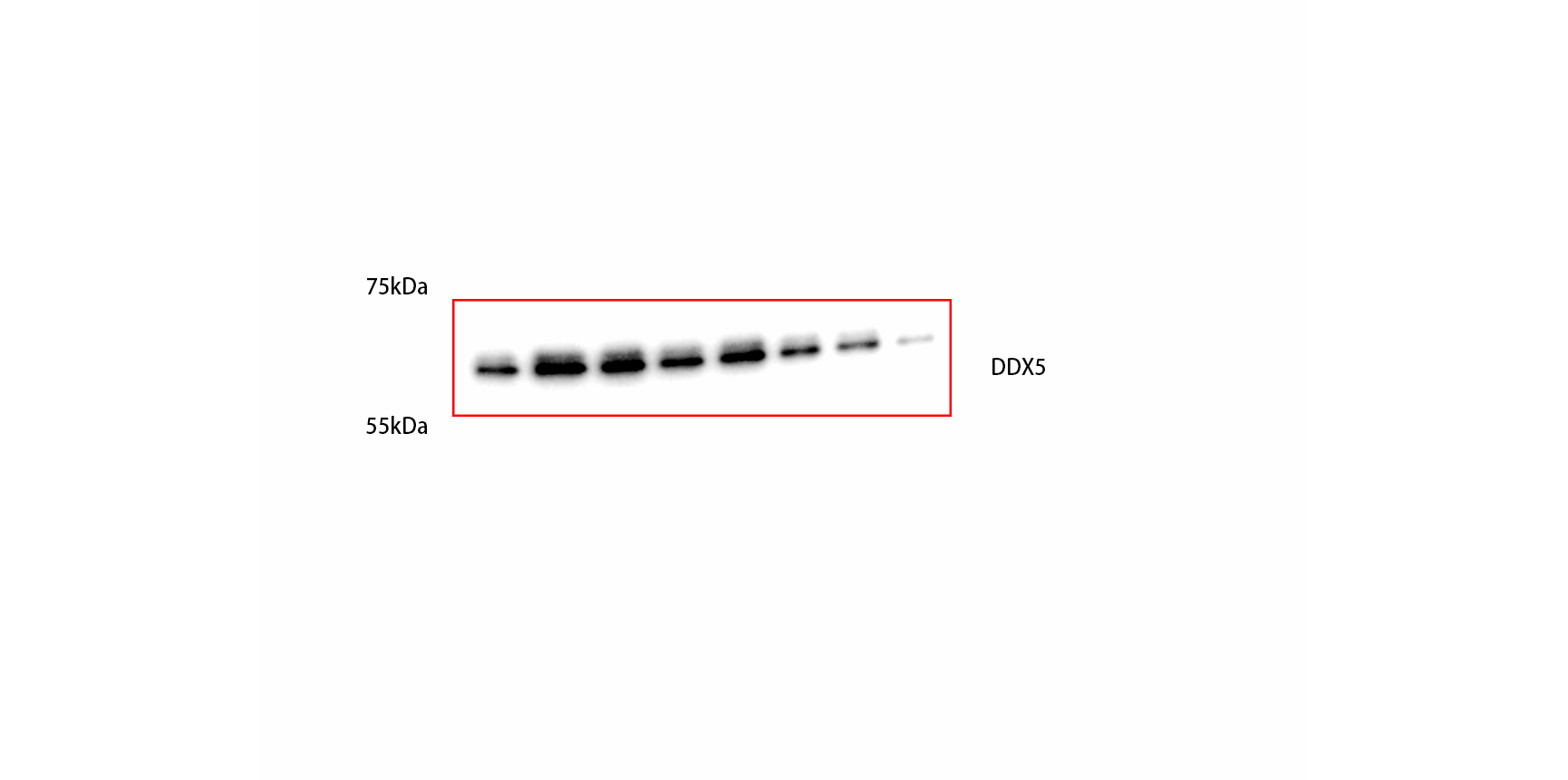

Supplement: Supplementary file 4 — Source Data Fig. 3 [file 44319_2023_47_MOESM4_ESM.zip › EMBOR-2023-57416V3-Figure_3_Source_Data-sd/Figure 3/A/DDX5.tif]

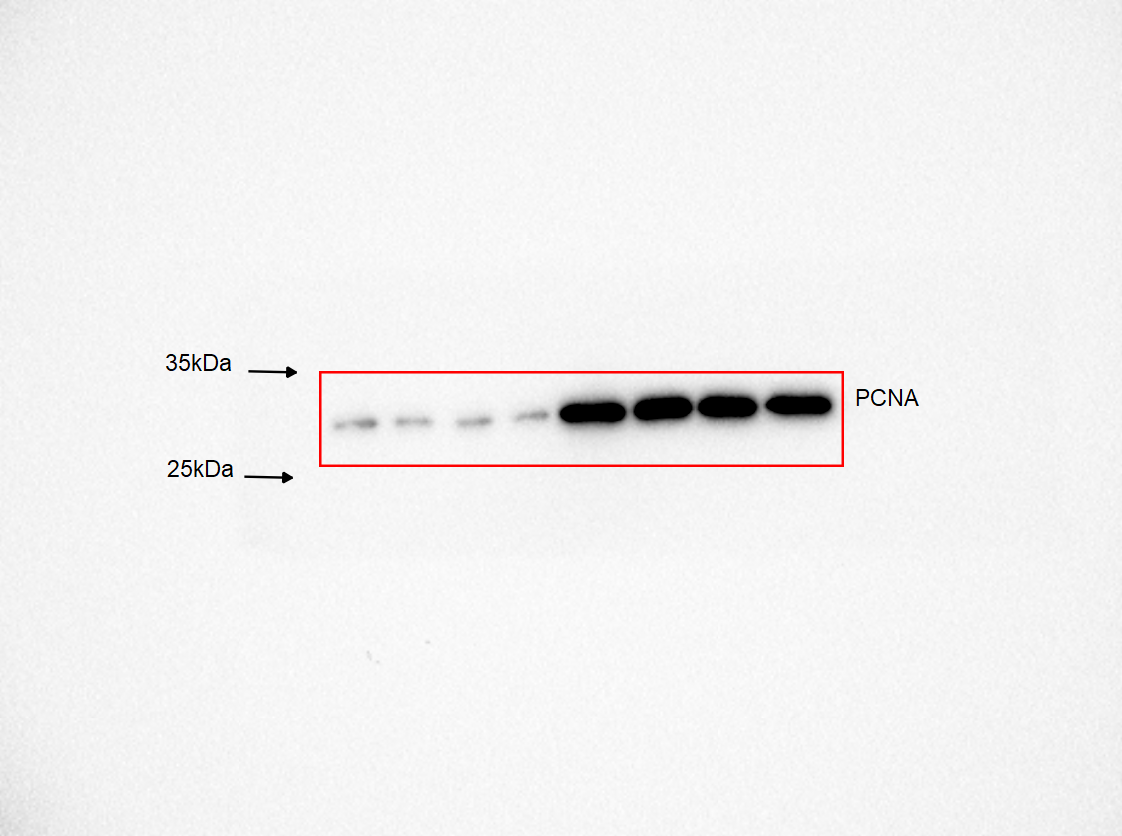

Supplement: Supplementary file 4 — Source Data Fig. 3 [file 44319_2023_47_MOESM4_ESM.zip › EMBOR-2023-57416V3-Figure_3_Source_Data-sd/Figure 3/A/PCNA.tif]

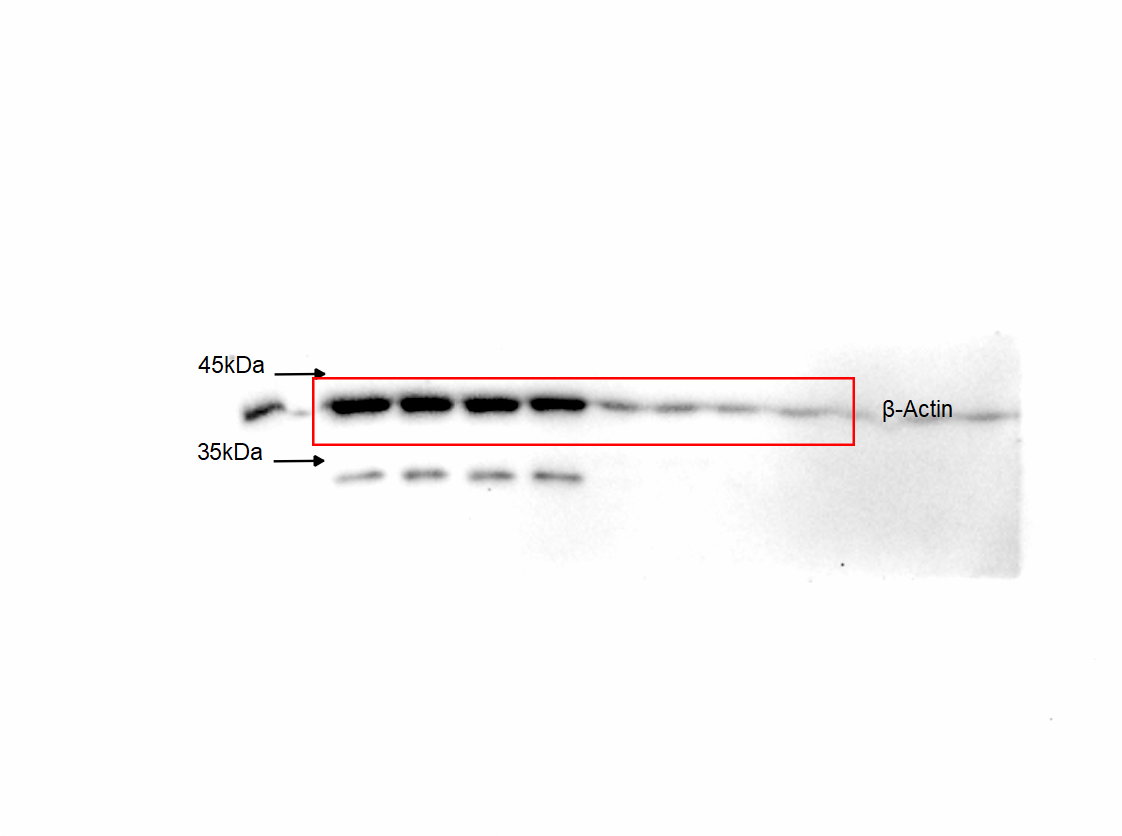

Supplement: Supplementary file 4 — Source Data Fig. 3 [file 44319_2023_47_MOESM4_ESM.zip › EMBOR-2023-57416V3-Figure_3_Source_Data-sd/Figure 3/A/β-Actin.tif]

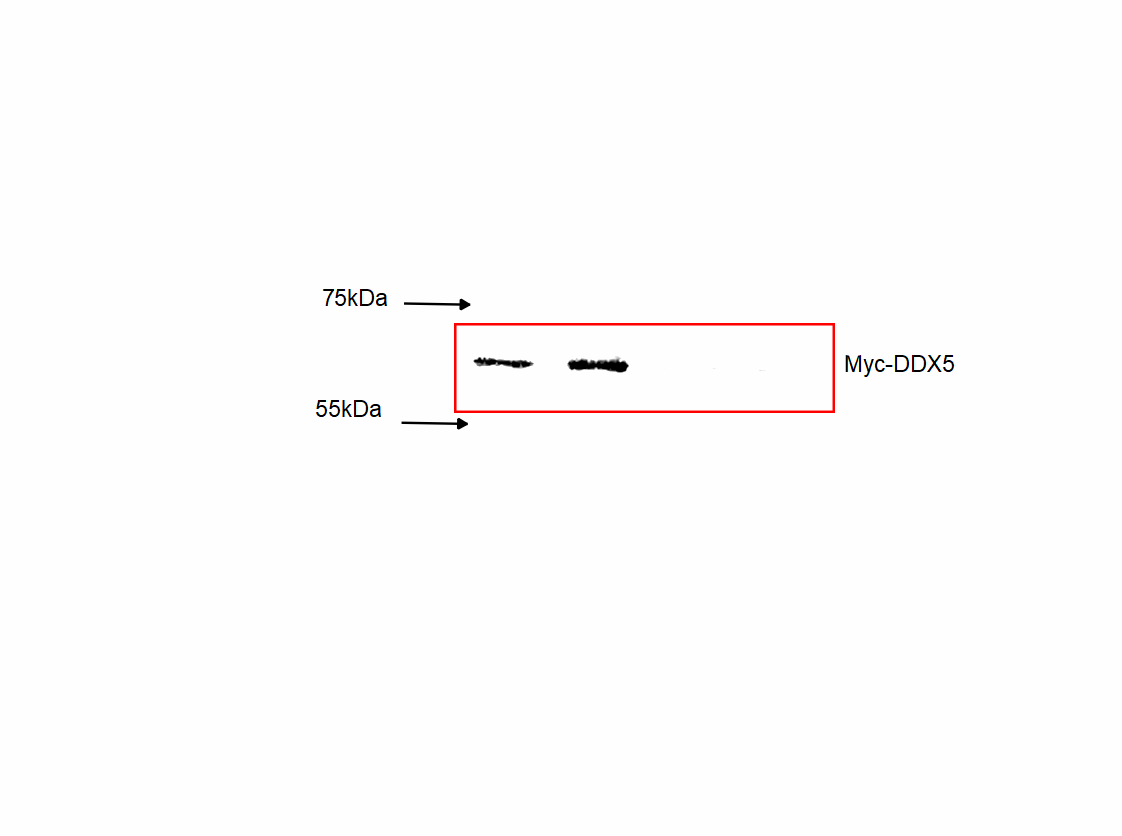

Supplement: Supplementary file 4 — Source Data Fig. 3 [file 44319_2023_47_MOESM4_ESM.zip › EMBOR-2023-57416V3-Figure_3_Source_Data-sd/Figure 3/B/IP-DDX5.tif]

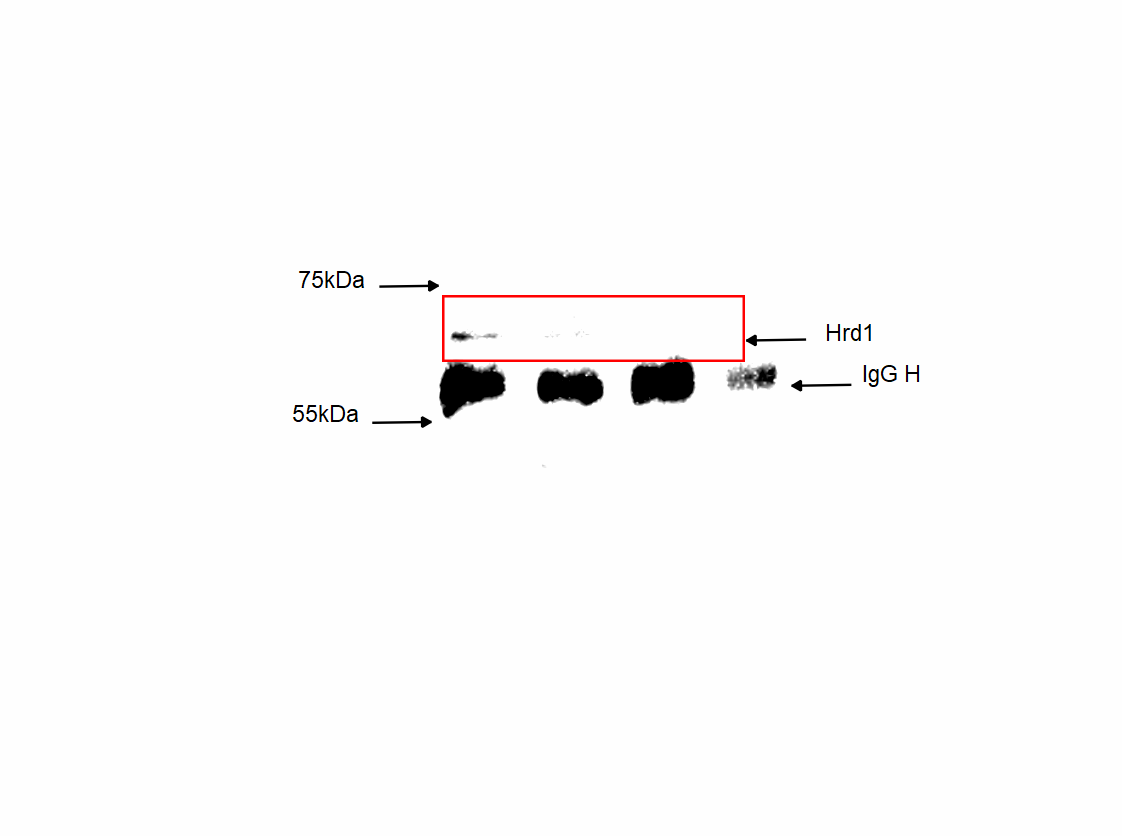

Supplement: Supplementary file 4 — Source Data Fig. 3 [file 44319_2023_47_MOESM4_ESM.zip › EMBOR-2023-57416V3-Figure_3_Source_Data-sd/Figure 3/B/IP-Hrd1.tif]

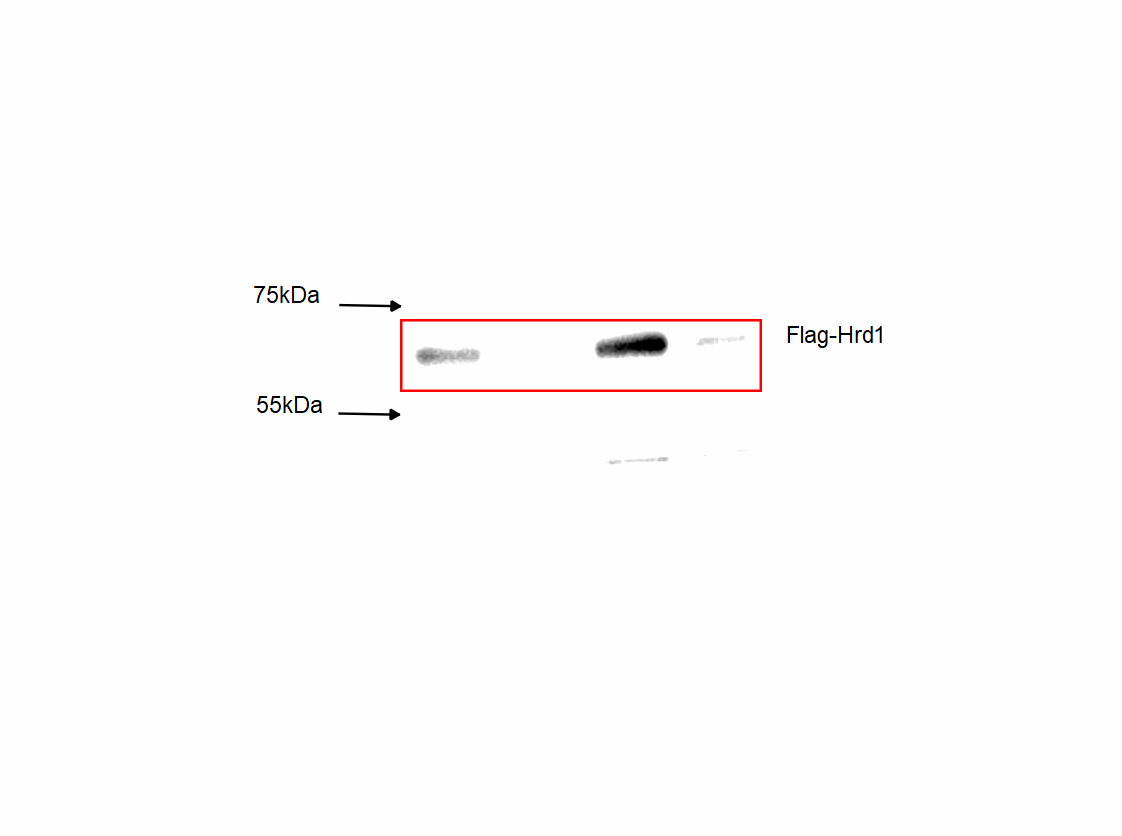

Supplement: Supplementary file 4 — Source Data Fig. 3 [file 44319_2023_47_MOESM4_ESM.zip › EMBOR-2023-57416V3-Figure_3_Source_Data-sd/Figure 3/B/WCL-Flag-Hrd1.tif]

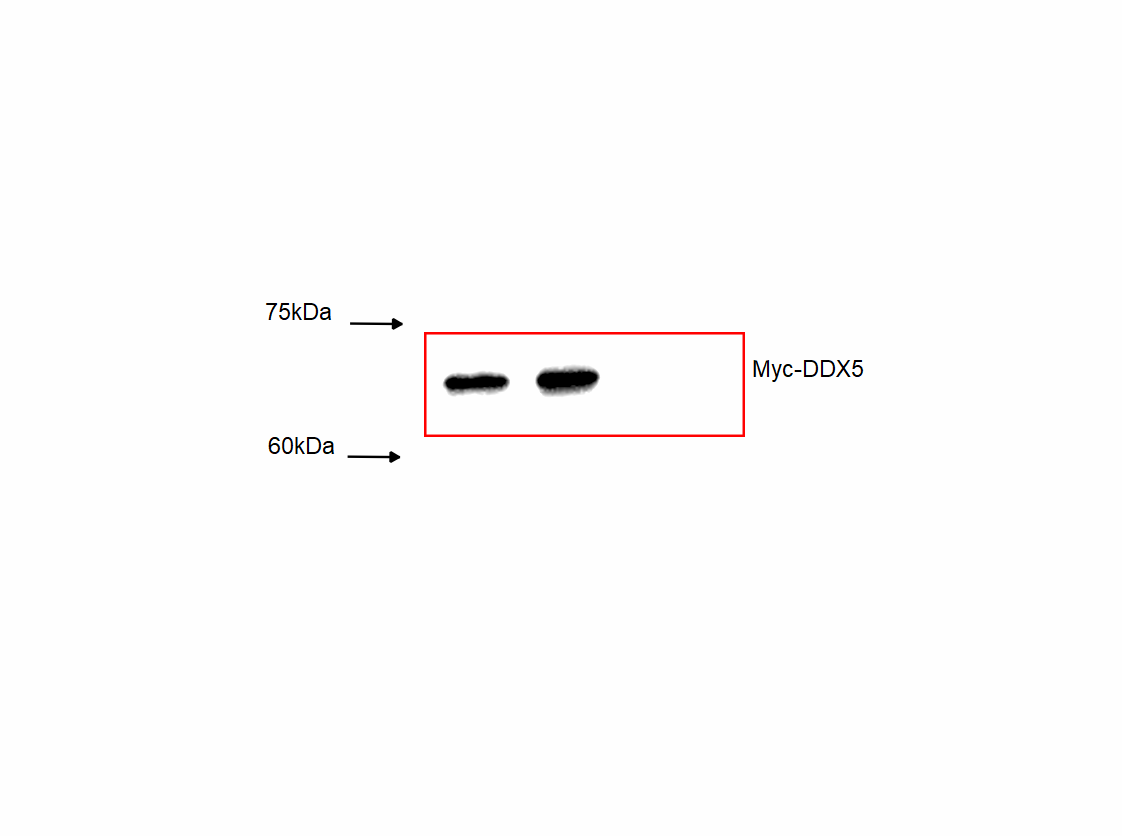

Supplement: Supplementary file 4 — Source Data Fig. 3 [file 44319_2023_47_MOESM4_ESM.zip › EMBOR-2023-57416V3-Figure_3_Source_Data-sd/Figure 3/B/WCL-Myc-DDX5.tif]

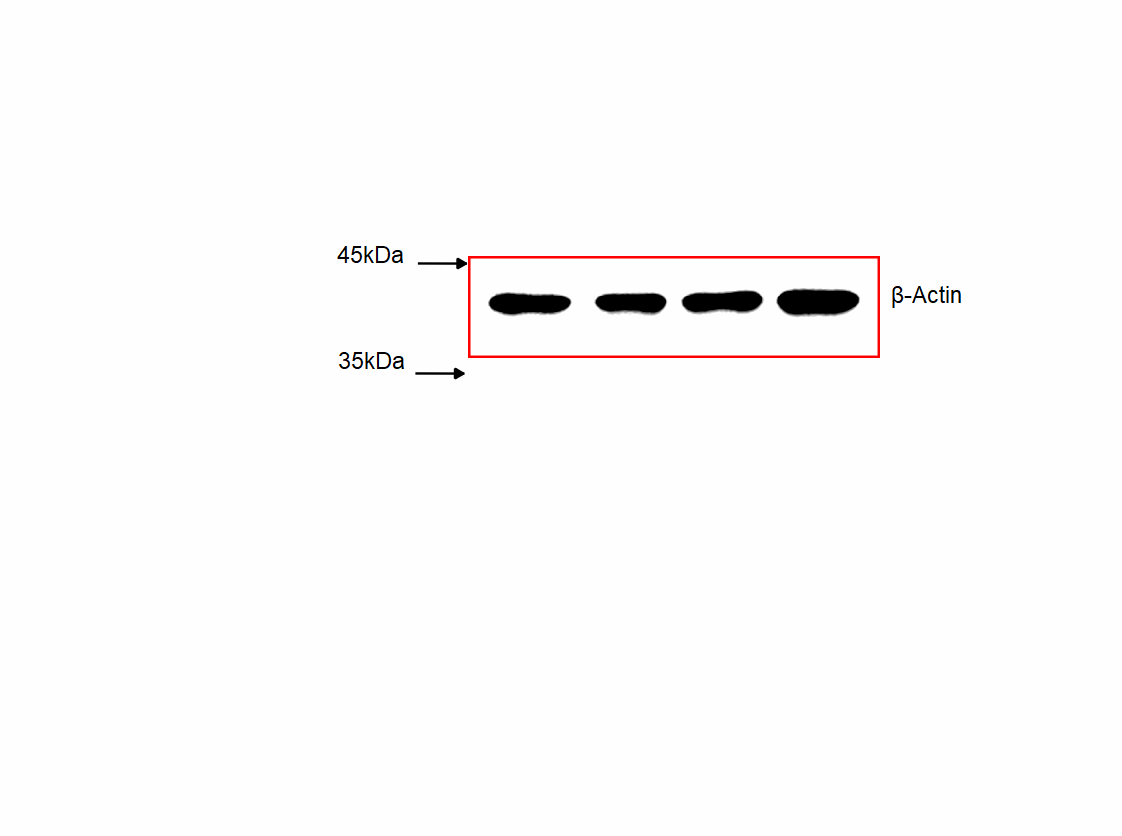

Supplement: Supplementary file 4 — Source Data Fig. 3 [file 44319_2023_47_MOESM4_ESM.zip › EMBOR-2023-57416V3-Figure_3_Source_Data-sd/Figure 3/B/WCL-β-Actin.tif]

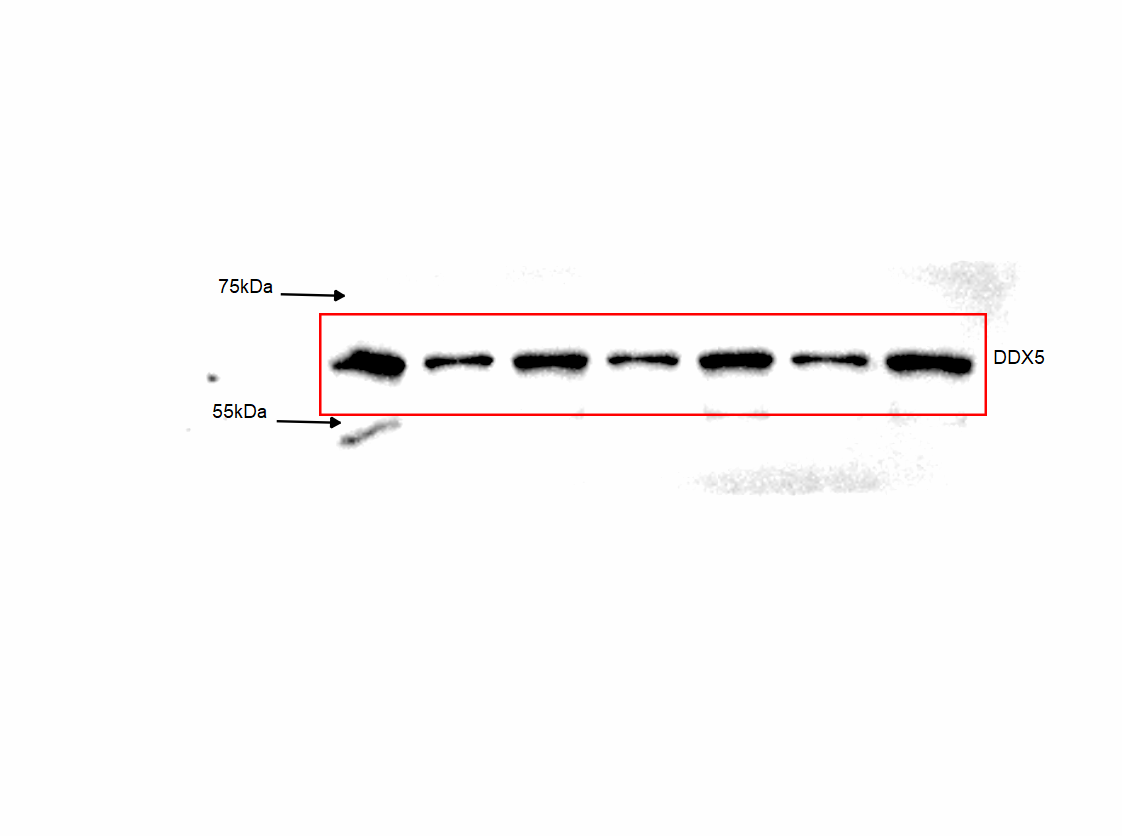

Supplement: Supplementary file 4 — Source Data Fig. 3 [file 44319_2023_47_MOESM4_ESM.zip › EMBOR-2023-57416V3-Figure_3_Source_Data-sd/Figure 3/C/DDX5.tif]

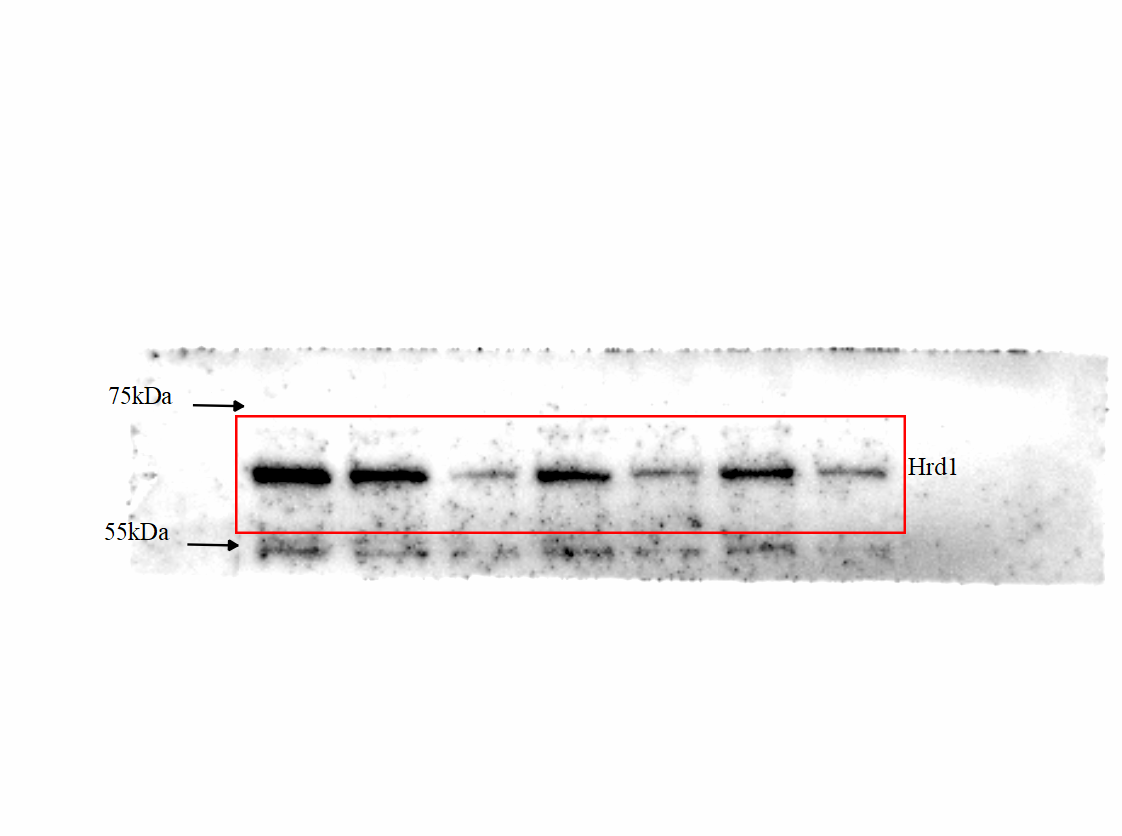

Supplement: Supplementary file 4 — Source Data Fig. 3 [file 44319_2023_47_MOESM4_ESM.zip › EMBOR-2023-57416V3-Figure_3_Source_Data-sd/Figure 3/C/HRD1.tif]

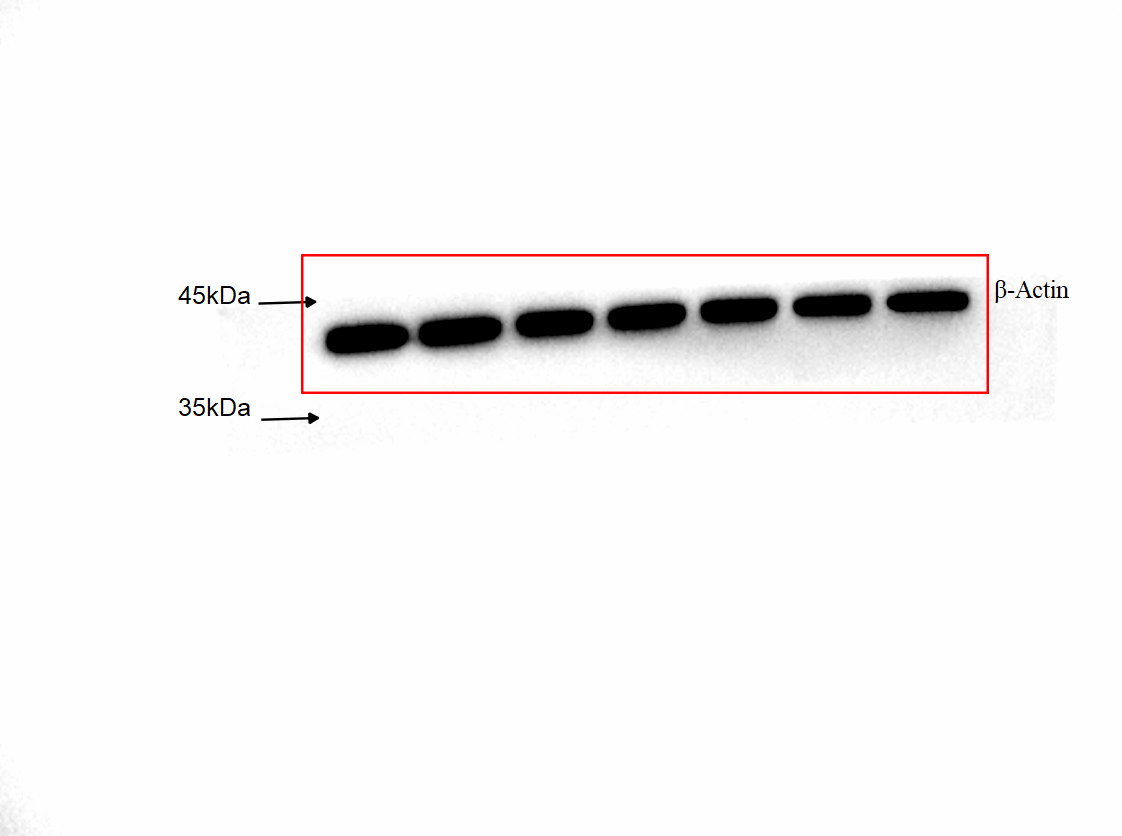

Supplement: Supplementary file 4 — Source Data Fig. 3 [file 44319_2023_47_MOESM4_ESM.zip › EMBOR-2023-57416V3-Figure_3_Source_Data-sd/Figure 3/C/β-Actin.tif]

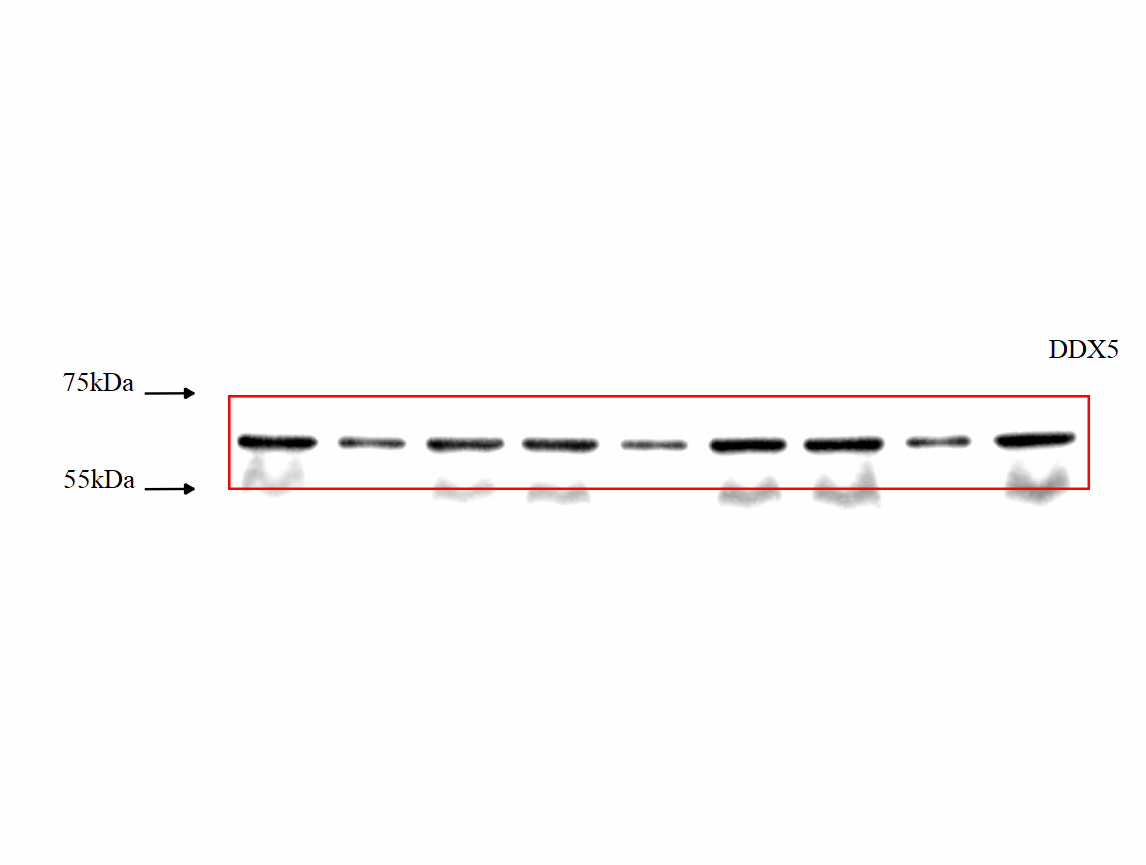

Supplement: Supplementary file 4 — Source Data Fig. 3 [file 44319_2023_47_MOESM4_ESM.zip › EMBOR-2023-57416V3-Figure_3_Source_Data-sd/Figure 3/D/DDX5.tif]

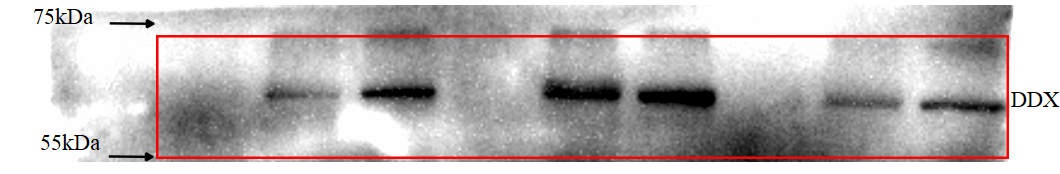

Supplement: Supplementary file 4 — Source Data Fig. 3 [file 44319_2023_47_MOESM4_ESM.zip › EMBOR-2023-57416V3-Figure_3_Source_Data-sd/Figure 3/D/Hrd1.tif]

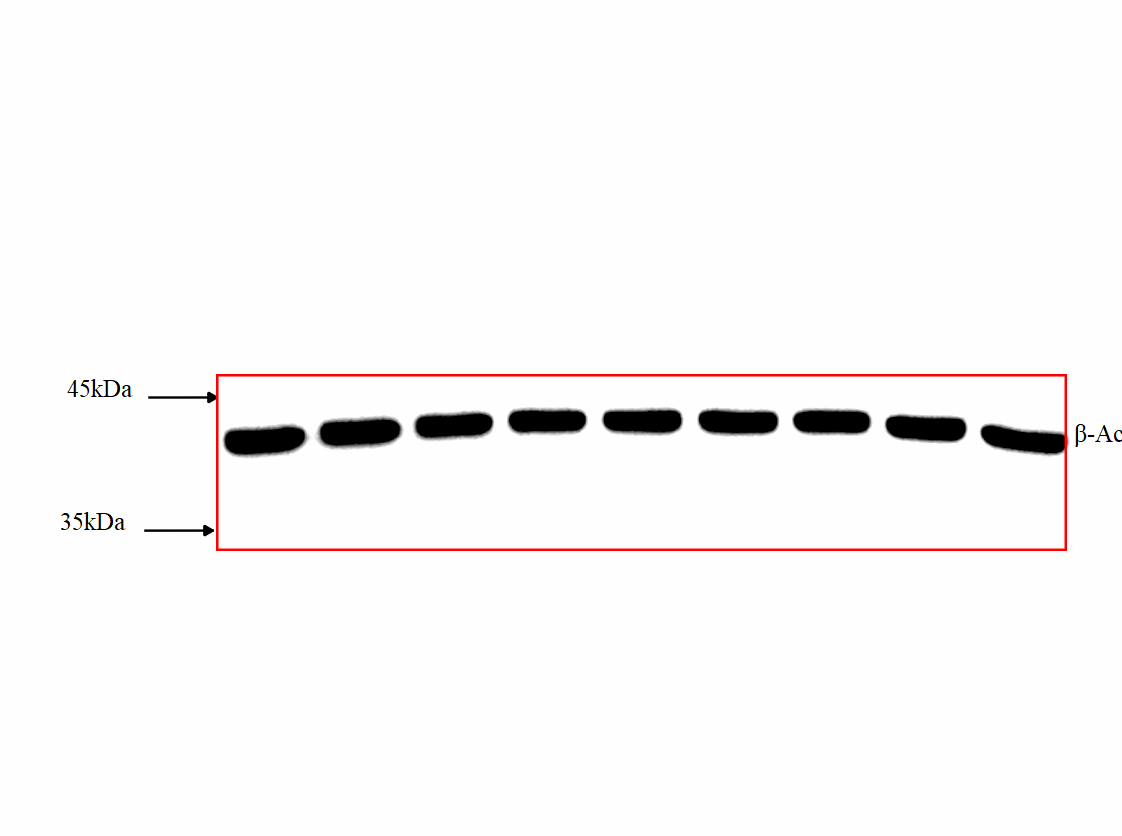

Supplement: Supplementary file 4 — Source Data Fig. 3 [file 44319_2023_47_MOESM4_ESM.zip › EMBOR-2023-57416V3-Figure_3_Source_Data-sd/Figure 3/D/β-Actin.tif]

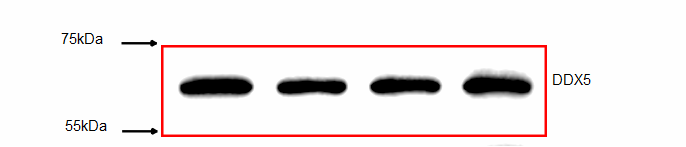

Supplement: Supplementary file 4 — Source Data Fig. 3 [file 44319_2023_47_MOESM4_ESM.zip › EMBOR-2023-57416V3-Figure_3_Source_Data-sd/Figure 3/E/IP-DDX5.tif]

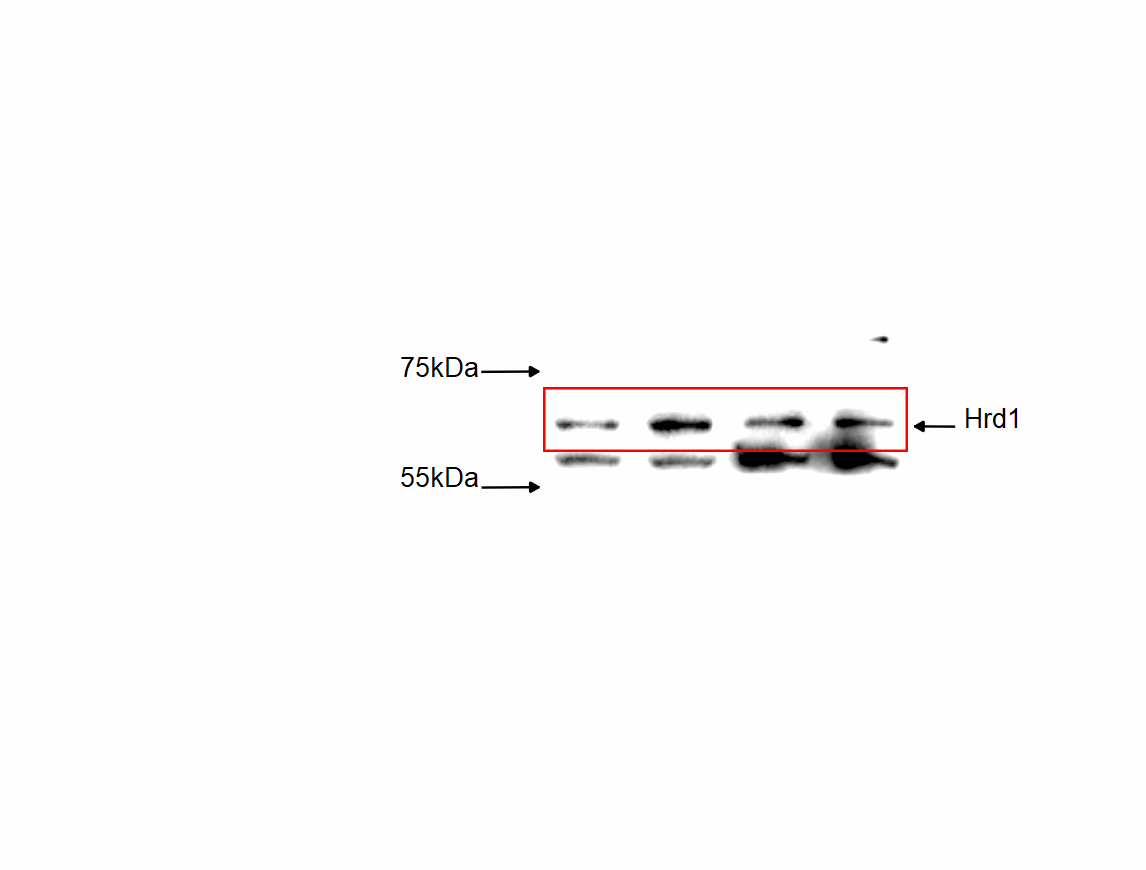

Supplement: Supplementary file 4 — Source Data Fig. 3 [file 44319_2023_47_MOESM4_ESM.zip › EMBOR-2023-57416V3-Figure_3_Source_Data-sd/Figure 3/E/IP-Hrd1.tif]

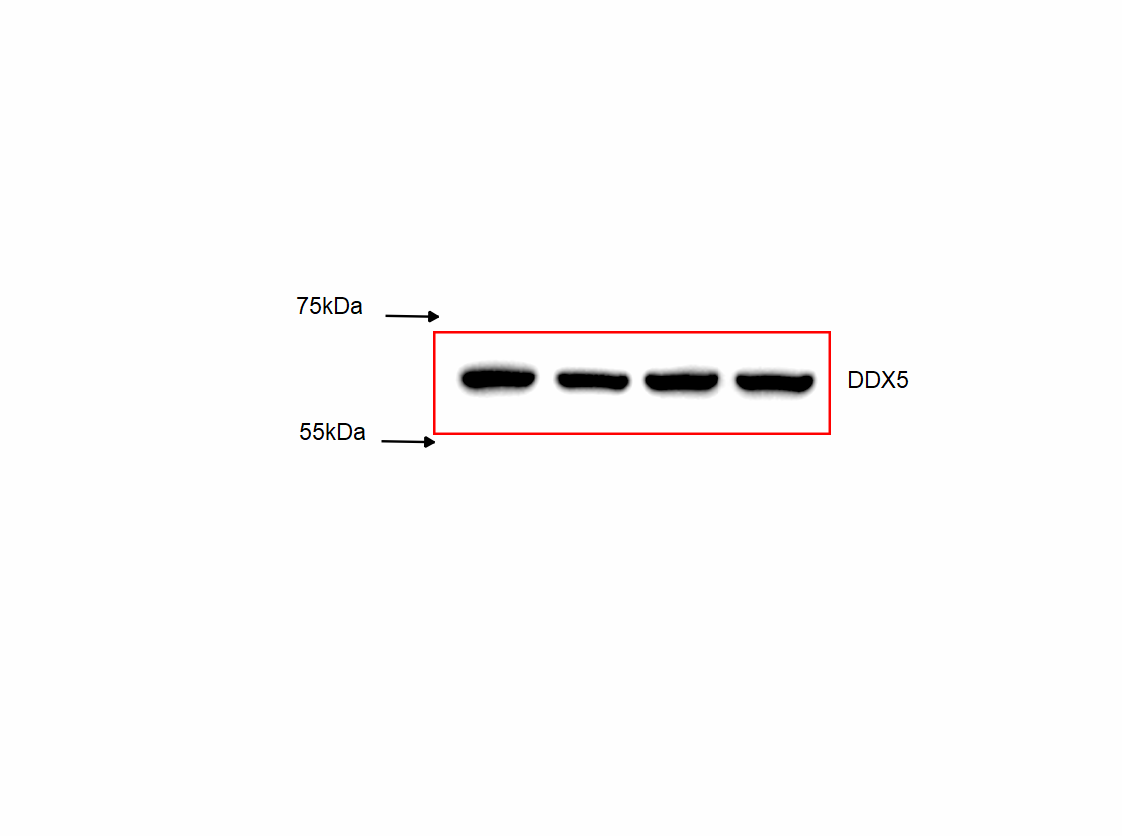

Supplement: Supplementary file 4 — Source Data Fig. 3 [file 44319_2023_47_MOESM4_ESM.zip › EMBOR-2023-57416V3-Figure_3_Source_Data-sd/Figure 3/E/WCL-DDX5.tif]

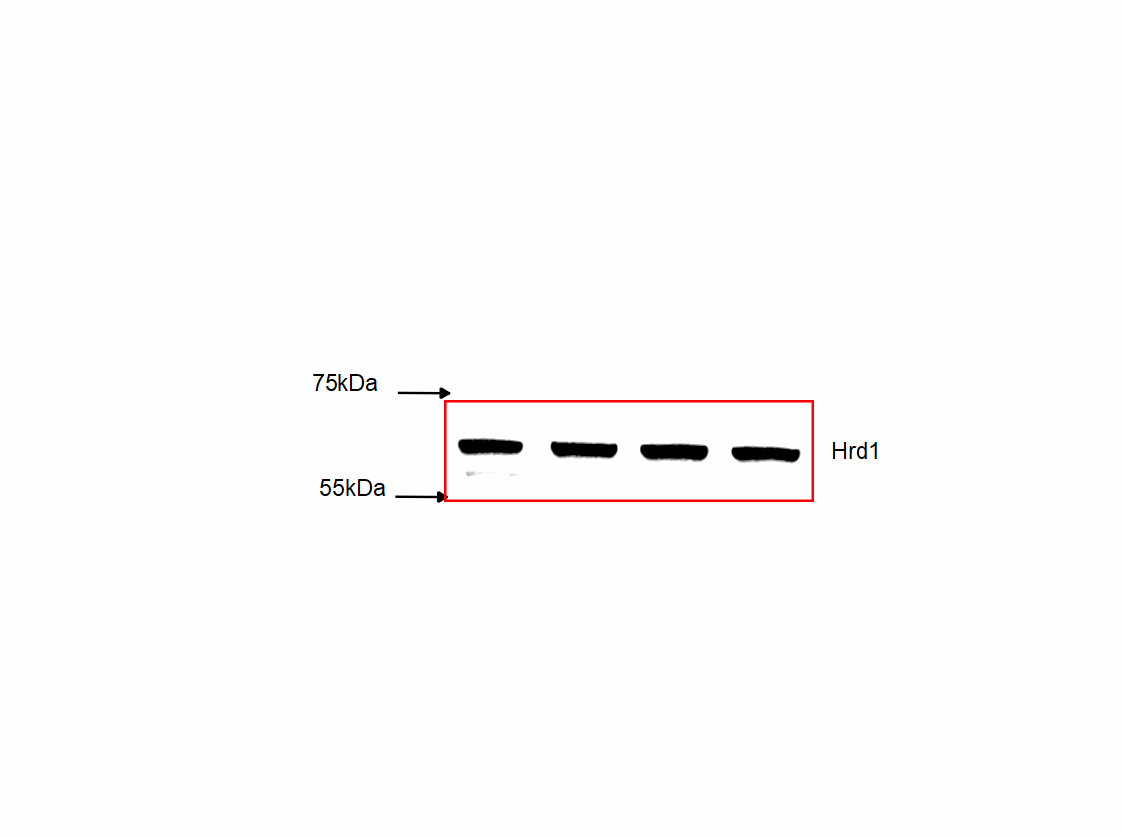

Supplement: Supplementary file 4 — Source Data Fig. 3 [file 44319_2023_47_MOESM4_ESM.zip › EMBOR-2023-57416V3-Figure_3_Source_Data-sd/Figure 3/E/WCL-Hrd1.tif]

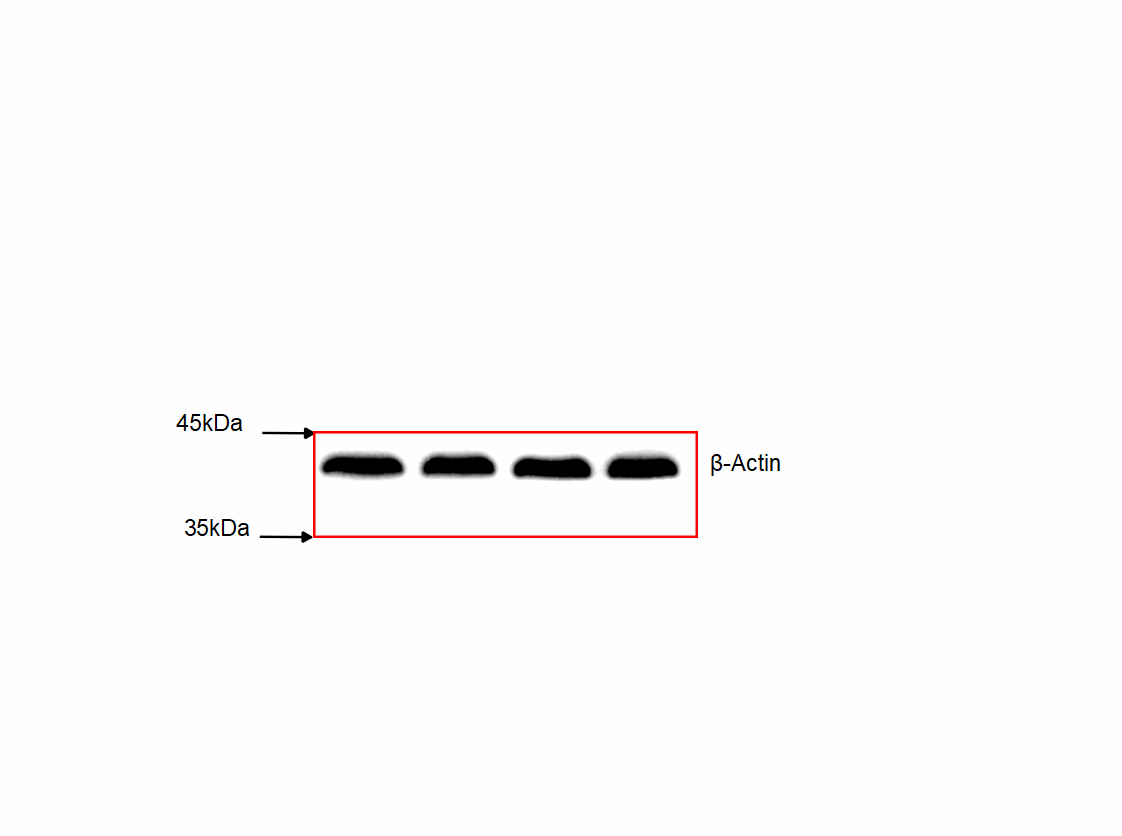

Supplement: Supplementary file 4 — Source Data Fig. 3 [file 44319_2023_47_MOESM4_ESM.zip › EMBOR-2023-57416V3-Figure_3_Source_Data-sd/Figure 3/E/WCL-β-Actin.tif]

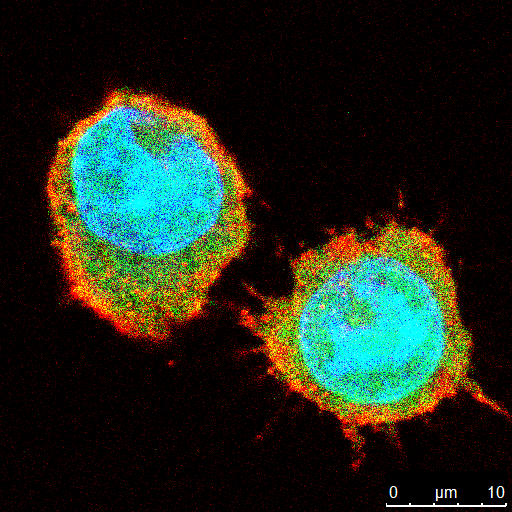

Supplement: Supplementary file 4 — Source Data Fig. 3 [file 44319_2023_47_MOESM4_ESM.zip › EMBOR-2023-57416V3-Figure_3_Source_Data-sd/Figure 3/F/Control/Experiment.lif_Series087_z0.tif]

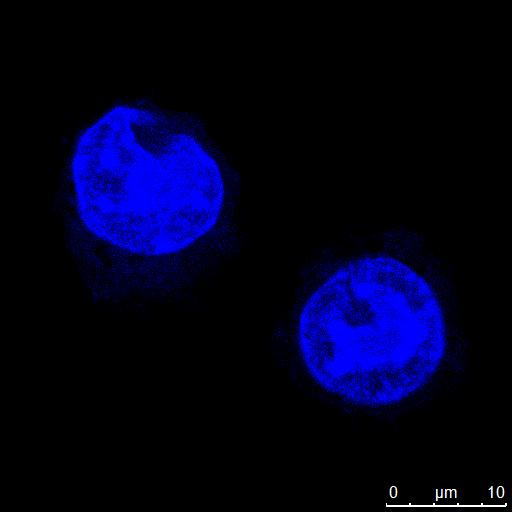

Supplement: Supplementary file 4 — Source Data Fig. 3 [file 44319_2023_47_MOESM4_ESM.zip › EMBOR-2023-57416V3-Figure_3_Source_Data-sd/Figure 3/F/Control/Experiment.lif_Series087_z0_ch00.tif]

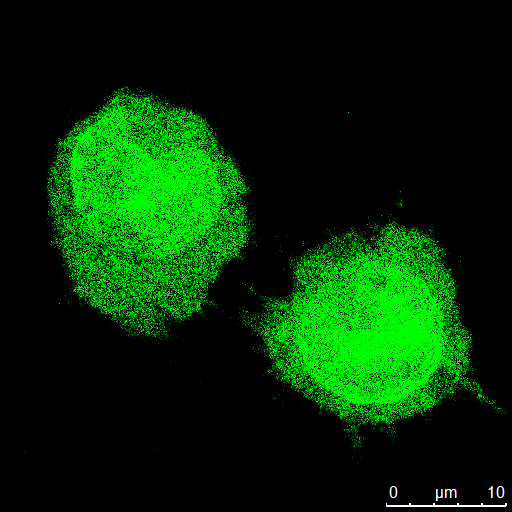

Supplement: Supplementary file 4 — Source Data Fig. 3 [file 44319_2023_47_MOESM4_ESM.zip › EMBOR-2023-57416V3-Figure_3_Source_Data-sd/Figure 3/F/Control/Experiment.lif_Series087_z0_ch01.tif]

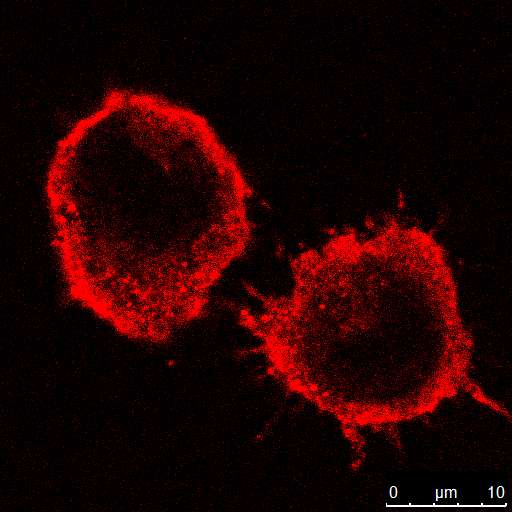

Supplement: Supplementary file 4 — Source Data Fig. 3 [file 44319_2023_47_MOESM4_ESM.zip › EMBOR-2023-57416V3-Figure_3_Source_Data-sd/Figure 3/F/Control/Experiment.lif_Series087_z0_ch02.tif]

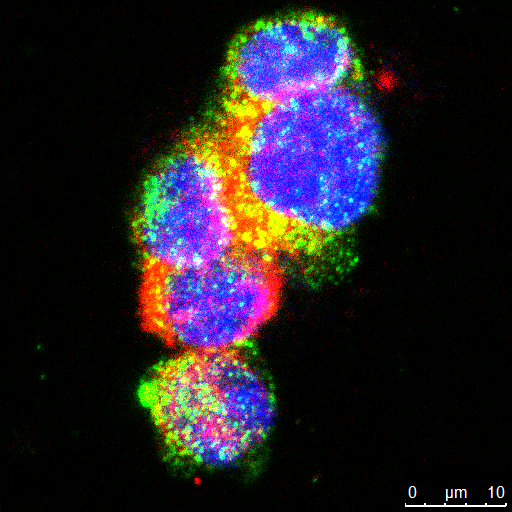

Supplement: Supplementary file 4 — Source Data Fig. 3 [file 44319_2023_47_MOESM4_ESM.zip › EMBOR-2023-57416V3-Figure_3_Source_Data-sd/Figure 3/F/FSL-1/Experiment.lif_Series085_z00.tif]

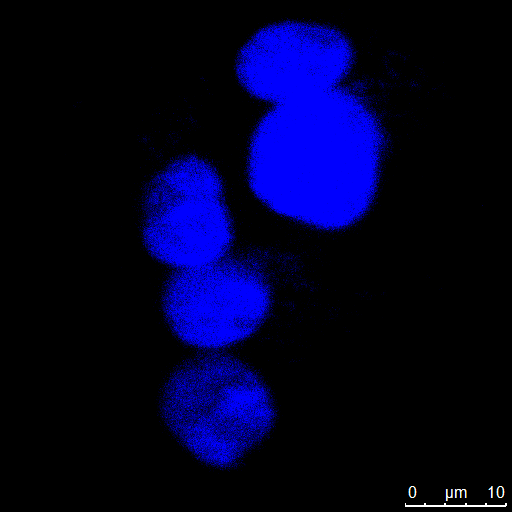

Supplement: Supplementary file 4 — Source Data Fig. 3 [file 44319_2023_47_MOESM4_ESM.zip › EMBOR-2023-57416V3-Figure_3_Source_Data-sd/Figure 3/F/FSL-1/Experiment.lif_Series085_z00_ch00.tif]

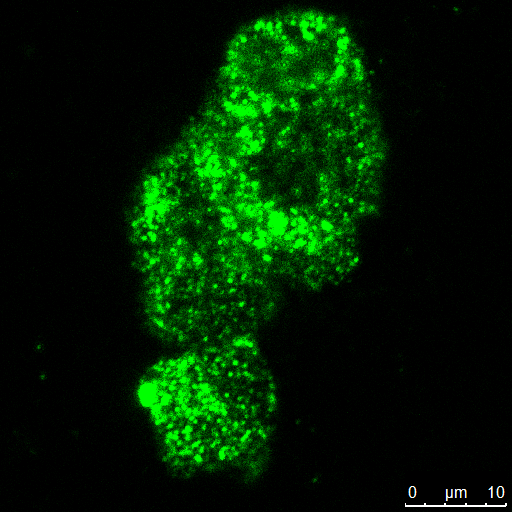

Supplement: Supplementary file 4 — Source Data Fig. 3 [file 44319_2023_47_MOESM4_ESM.zip › EMBOR-2023-57416V3-Figure_3_Source_Data-sd/Figure 3/F/FSL-1/Experiment.lif_Series085_z00_ch01.tif]

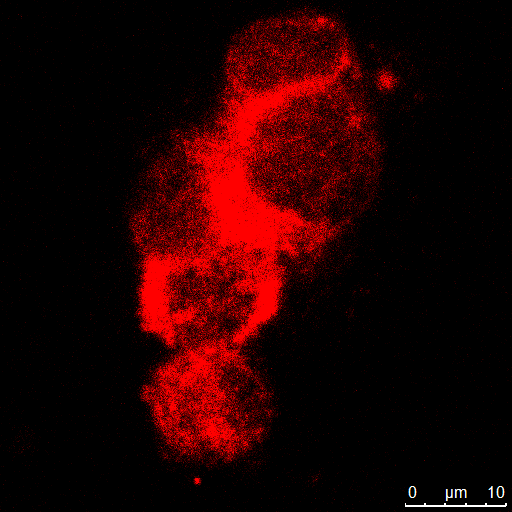

Supplement: Supplementary file 4 — Source Data Fig. 3 [file 44319_2023_47_MOESM4_ESM.zip › EMBOR-2023-57416V3-Figure_3_Source_Data-sd/Figure 3/F/FSL-1/Experiment.lif_Series085_z00_ch02.tif]

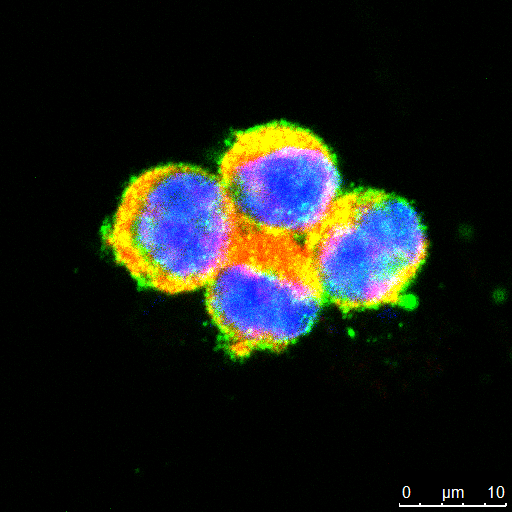

Supplement: Supplementary file 4 — Source Data Fig. 3 [file 44319_2023_47_MOESM4_ESM.zip › EMBOR-2023-57416V3-Figure_3_Source_Data-sd/Figure 3/F/LPS/Experiment.lif_Series071_z0.tif]

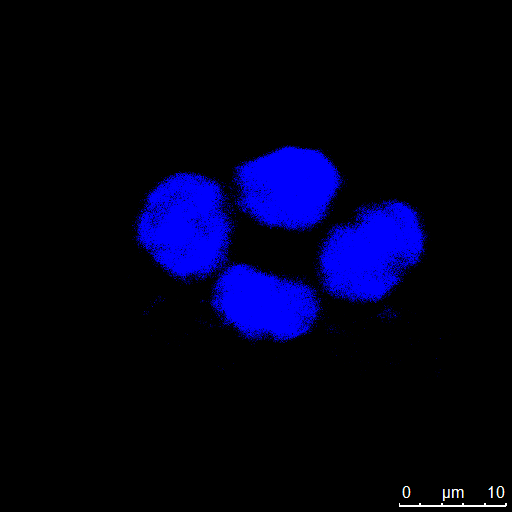

Supplement: Supplementary file 4 — Source Data Fig. 3 [file 44319_2023_47_MOESM4_ESM.zip › EMBOR-2023-57416V3-Figure_3_Source_Data-sd/Figure 3/F/LPS/Experiment.lif_Series071_z0_ch00.tif]

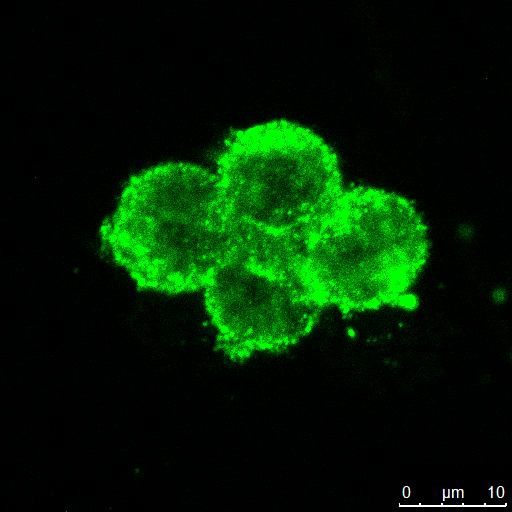

Supplement: Supplementary file 4 — Source Data Fig. 3 [file 44319_2023_47_MOESM4_ESM.zip › EMBOR-2023-57416V3-Figure_3_Source_Data-sd/Figure 3/F/LPS/Experiment.lif_Series071_z0_ch01.tif]

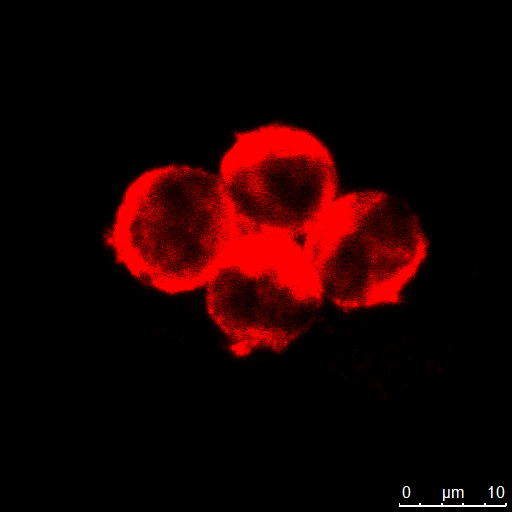

Supplement: Supplementary file 4 — Source Data Fig. 3 [file 44319_2023_47_MOESM4_ESM.zip › EMBOR-2023-57416V3-Figure_3_Source_Data-sd/Figure 3/F/LPS/Experiment.lif_Series071_z0_ch02.tif]

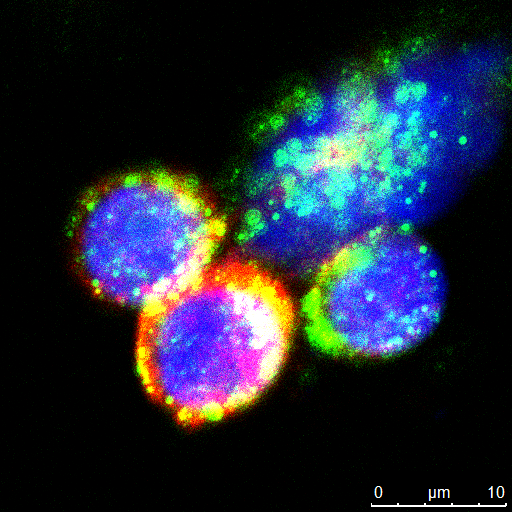

Supplement: Supplementary file 4 — Source Data Fig. 3 [file 44319_2023_47_MOESM4_ESM.zip › EMBOR-2023-57416V3-Figure_3_Source_Data-sd/Figure 3/F/Pam3CSK4/Experiment.lif_Series051_z0.tif]

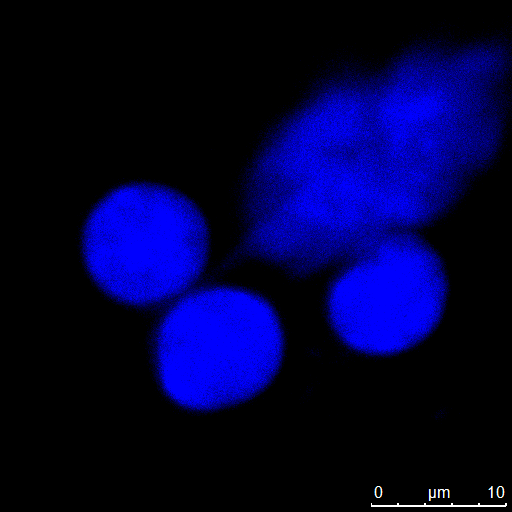

Supplement: Supplementary file 4 — Source Data Fig. 3 [file 44319_2023_47_MOESM4_ESM.zip › EMBOR-2023-57416V3-Figure_3_Source_Data-sd/Figure 3/F/Pam3CSK4/Experiment.lif_Series051_z0_ch00.tif]

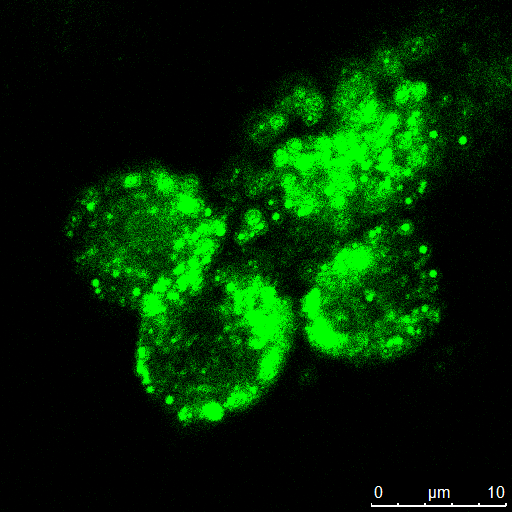

Supplement: Supplementary file 4 — Source Data Fig. 3 [file 44319_2023_47_MOESM4_ESM.zip › EMBOR-2023-57416V3-Figure_3_Source_Data-sd/Figure 3/F/Pam3CSK4/Experiment.lif_Series051_z0_ch01.tif]

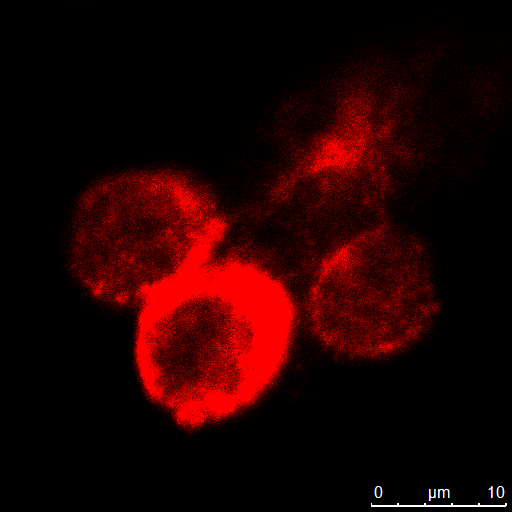

Supplement: Supplementary file 4 — Source Data Fig. 3 [file 44319_2023_47_MOESM4_ESM.zip › EMBOR-2023-57416V3-Figure_3_Source_Data-sd/Figure 3/F/Pam3CSK4/Experiment.lif_Series051_z0_ch02.tif]

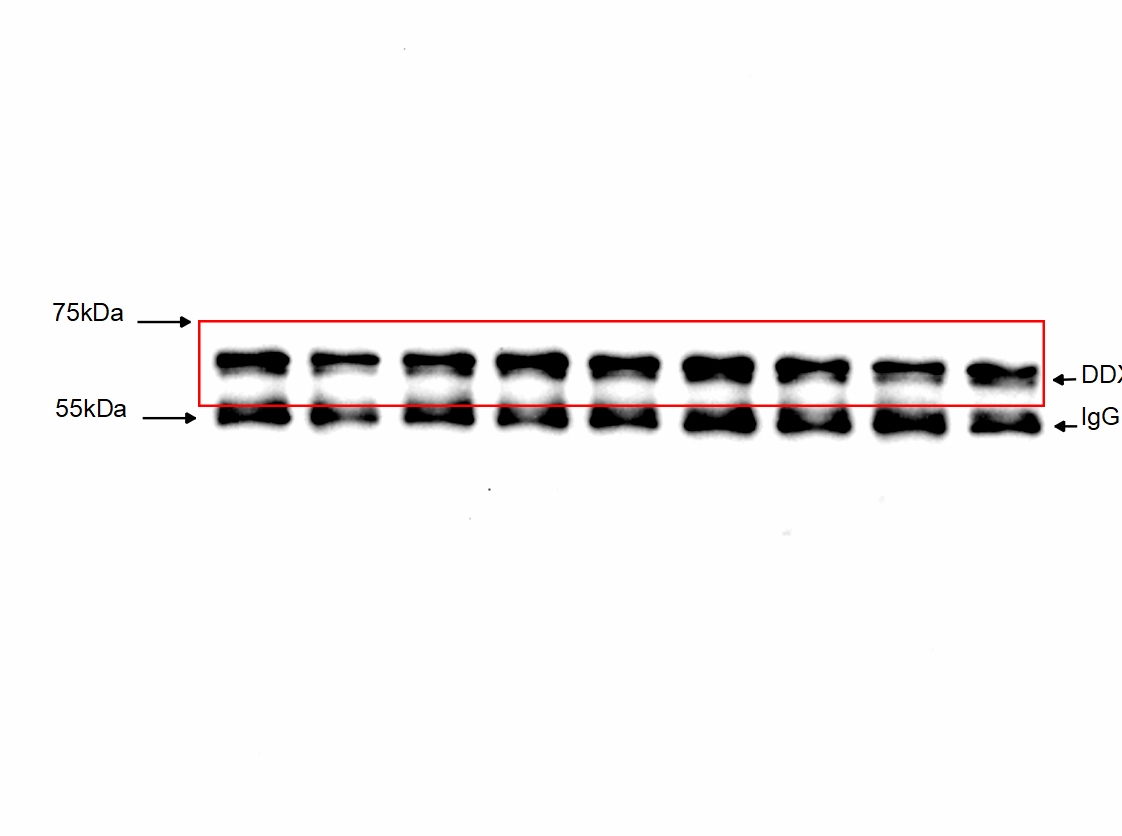

Supplement: Supplementary file 4 — Source Data Fig. 3 [file 44319_2023_47_MOESM4_ESM.zip › EMBOR-2023-57416V3-Figure_3_Source_Data-sd/Figure 3/G/IP-DDX5.tif]

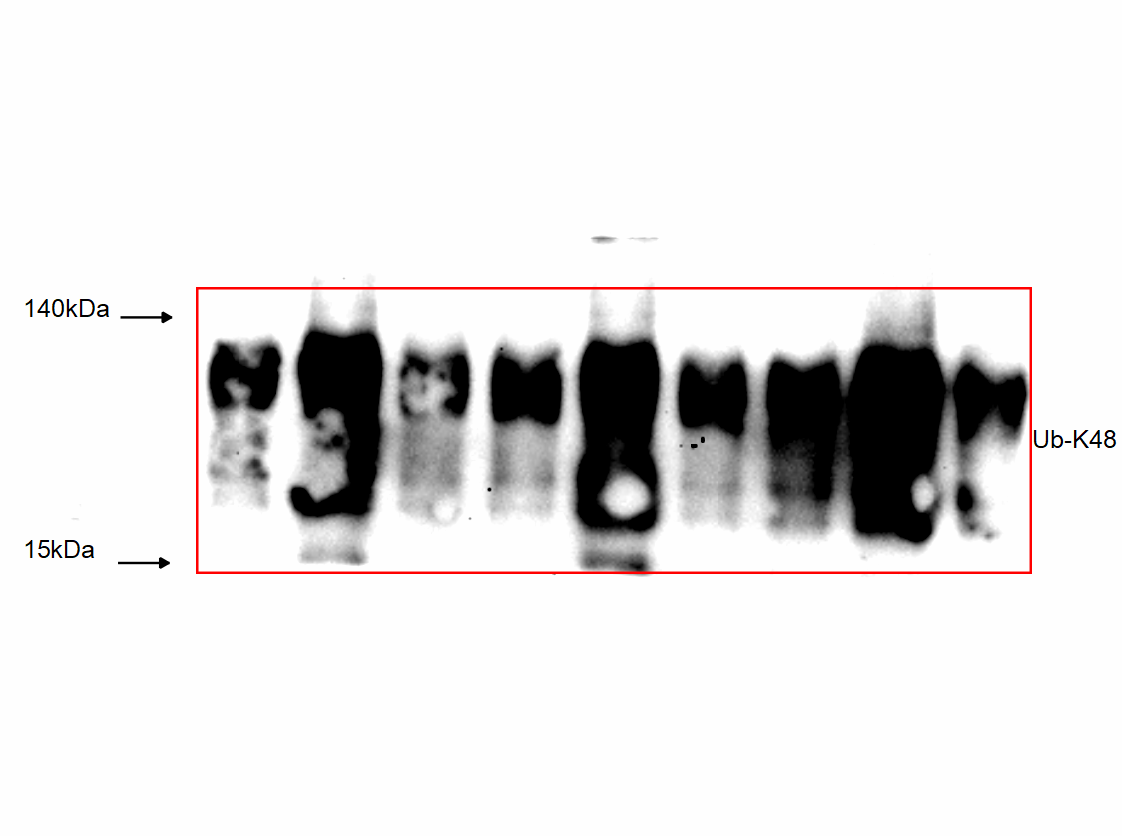

Supplement: Supplementary file 4 — Source Data Fig. 3 [file 44319_2023_47_MOESM4_ESM.zip › EMBOR-2023-57416V3-Figure_3_Source_Data-sd/Figure 3/G/IP-Ub-k48.tif]

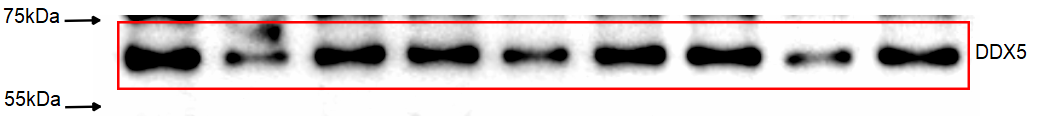

Supplement: Supplementary file 4 — Source Data Fig. 3 [file 44319_2023_47_MOESM4_ESM.zip › EMBOR-2023-57416V3-Figure_3_Source_Data-sd/Figure 3/G/WCL-DDX5.tif]

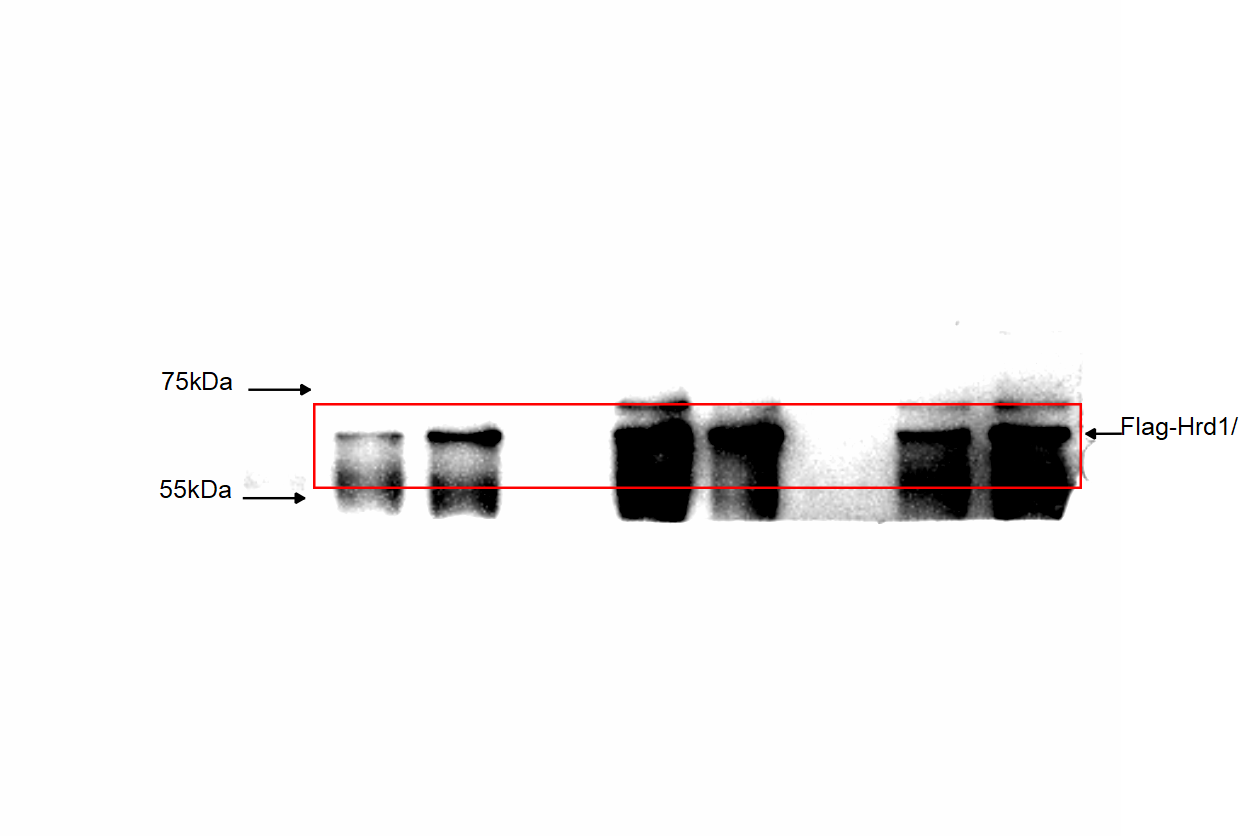

Supplement: Supplementary file 4 — Source Data Fig. 3 [file 44319_2023_47_MOESM4_ESM.zip › EMBOR-2023-57416V3-Figure_3_Source_Data-sd/Figure 3/G/WCL-Flag.tif]

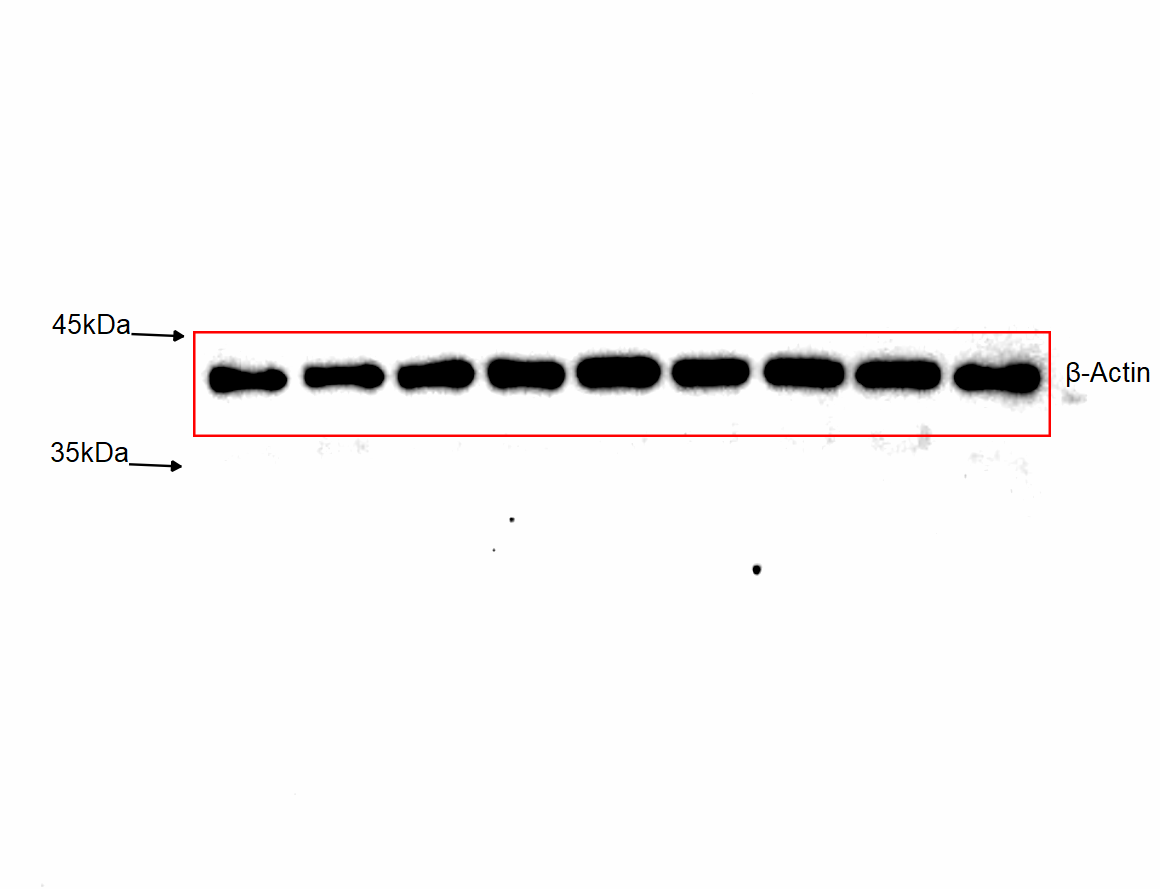

Supplement: Supplementary file 4 — Source Data Fig. 3 [file 44319_2023_47_MOESM4_ESM.zip › EMBOR-2023-57416V3-Figure_3_Source_Data-sd/Figure 3/G/WCL-β-Actin.tif]

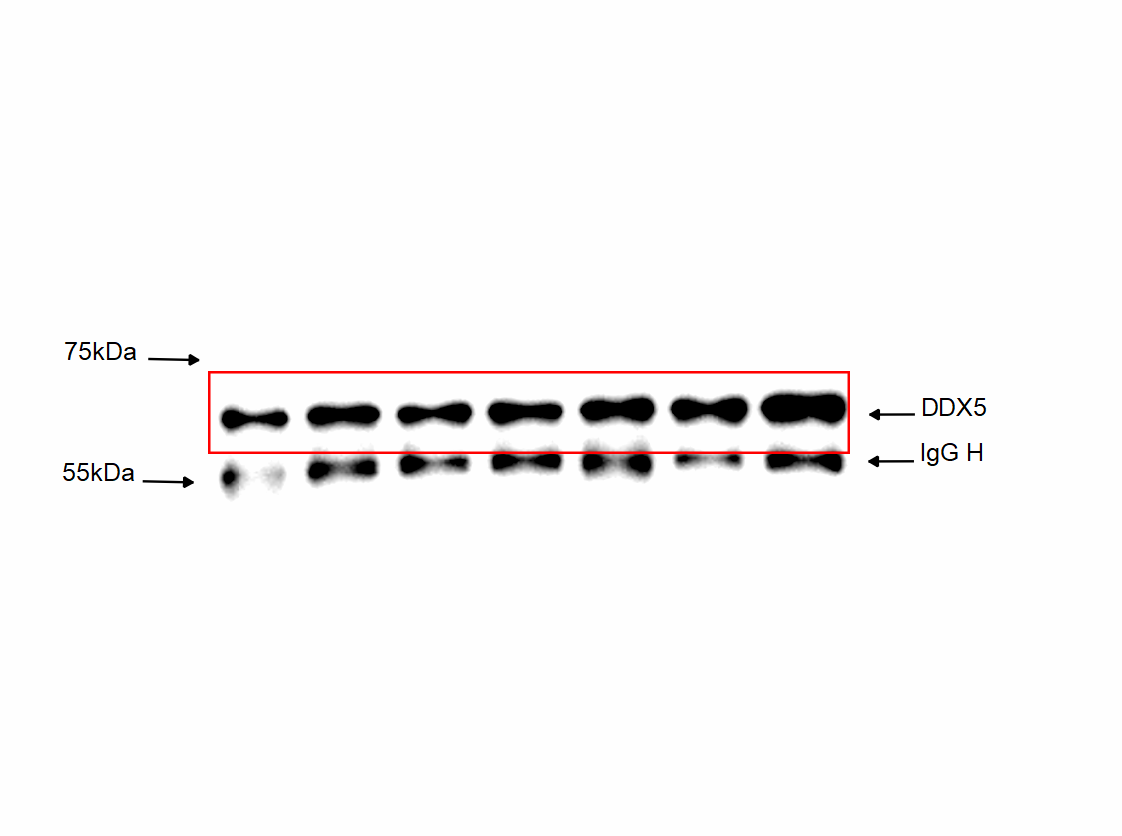

Supplement: Supplementary file 4 — Source Data Fig. 3 [file 44319_2023_47_MOESM4_ESM.zip › EMBOR-2023-57416V3-Figure_3_Source_Data-sd/Figure 3/H/IP-DDX5.tif]

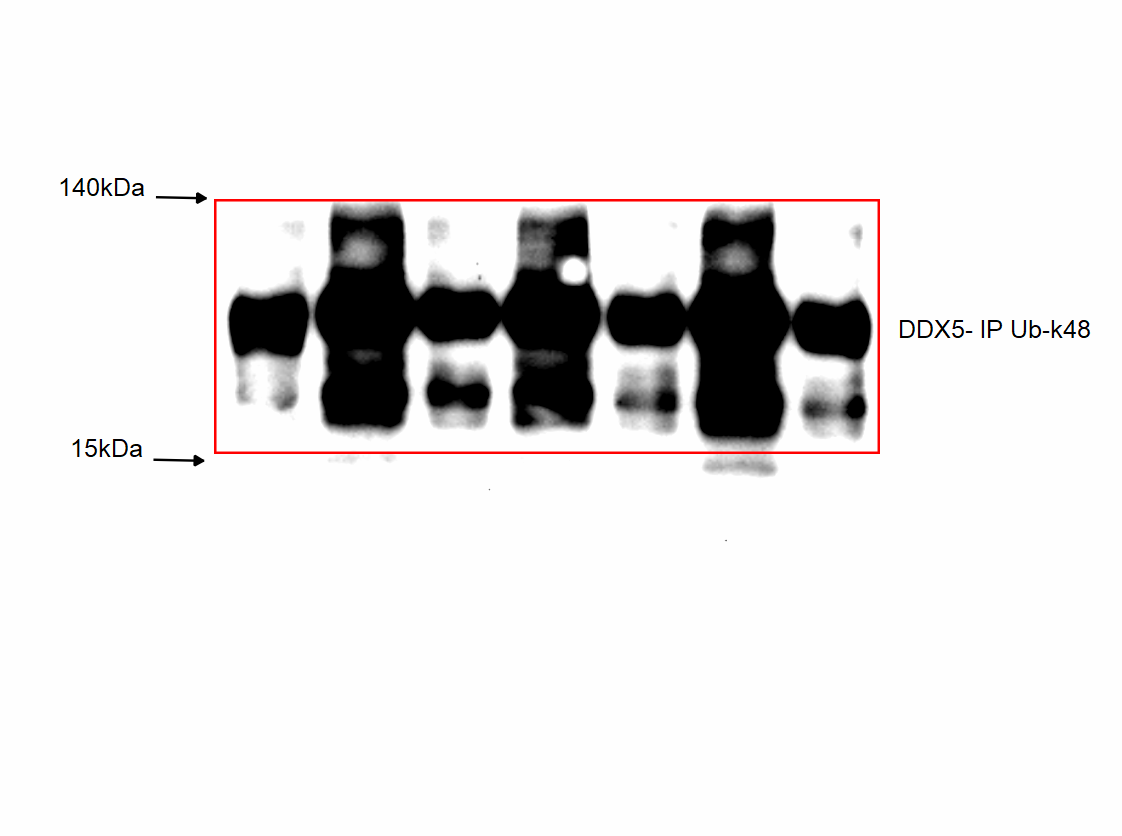

Supplement: Supplementary file 4 — Source Data Fig. 3 [file 44319_2023_47_MOESM4_ESM.zip › EMBOR-2023-57416V3-Figure_3_Source_Data-sd/Figure 3/H/IP-Ub-K48.tif]

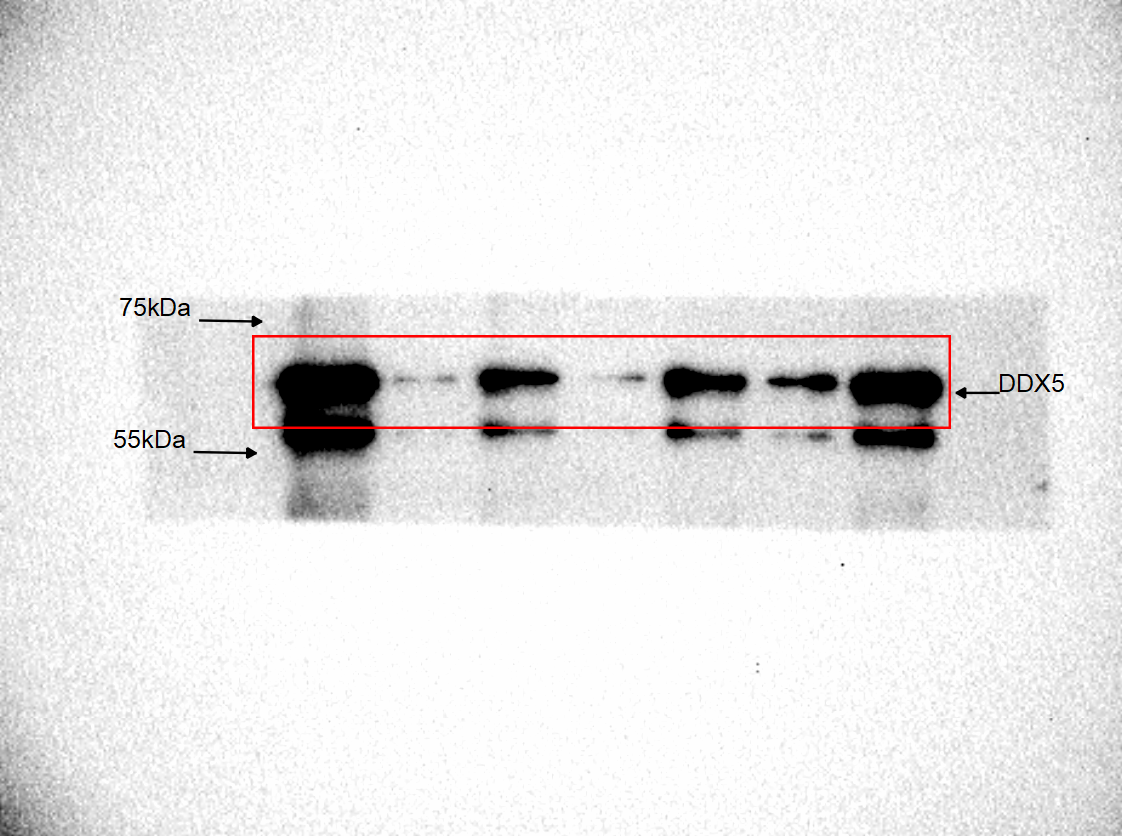

Supplement: Supplementary file 4 — Source Data Fig. 3 [file 44319_2023_47_MOESM4_ESM.zip › EMBOR-2023-57416V3-Figure_3_Source_Data-sd/Figure 3/H/WCL-DDX5.tif]

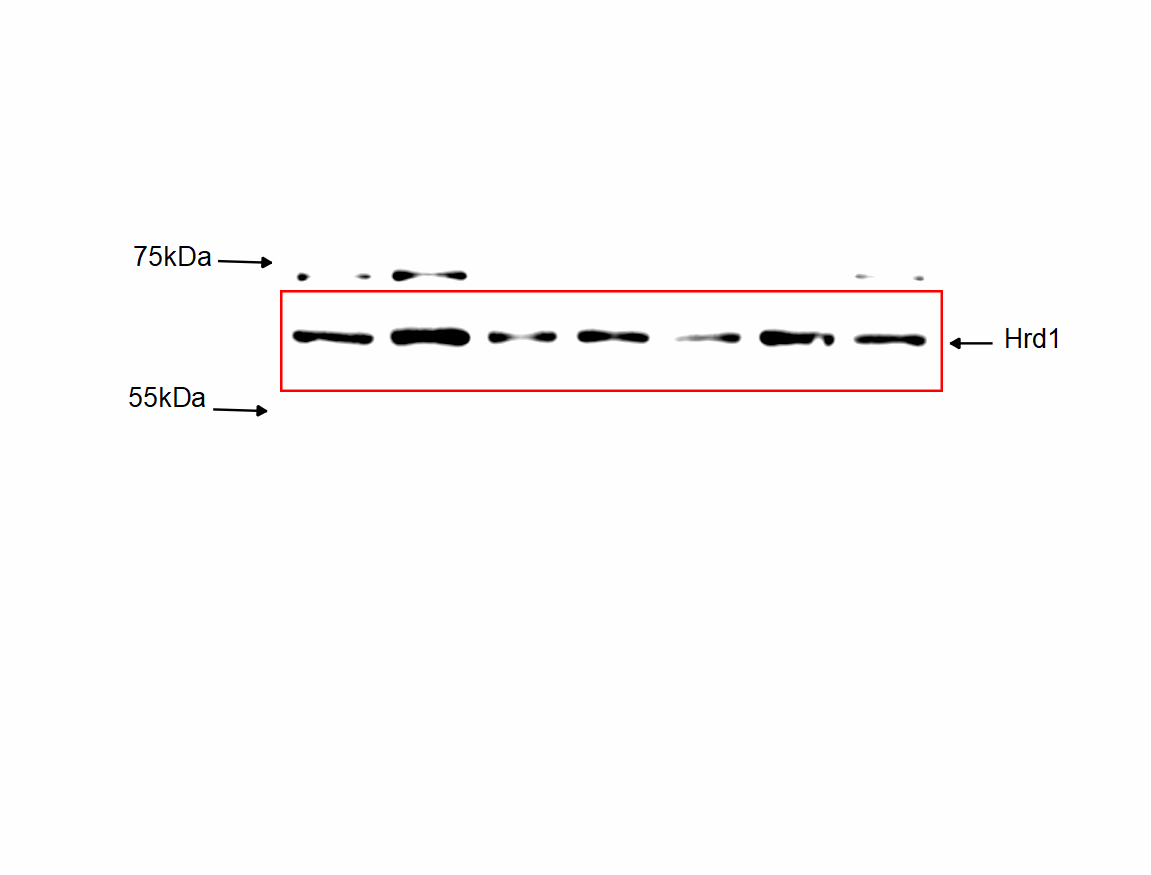

Supplement: Supplementary file 4 — Source Data Fig. 3 [file 44319_2023_47_MOESM4_ESM.zip › EMBOR-2023-57416V3-Figure_3_Source_Data-sd/Figure 3/H/WCL-Hrd1.tif]

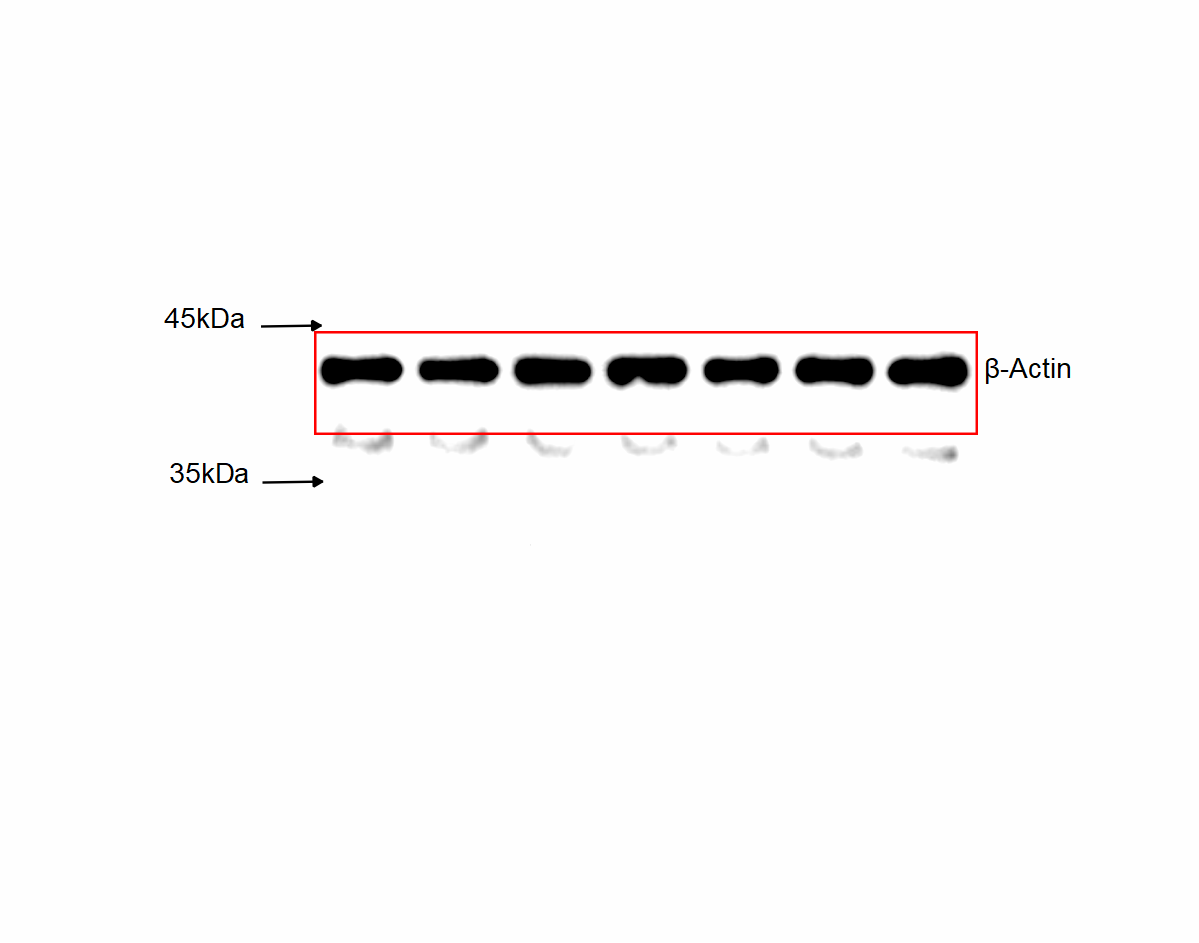

Supplement: Supplementary file 4 — Source Data Fig. 3 [file 44319_2023_47_MOESM4_ESM.zip › EMBOR-2023-57416V3-Figure_3_Source_Data-sd/Figure 3/H/WCL-β-Actin.tif]

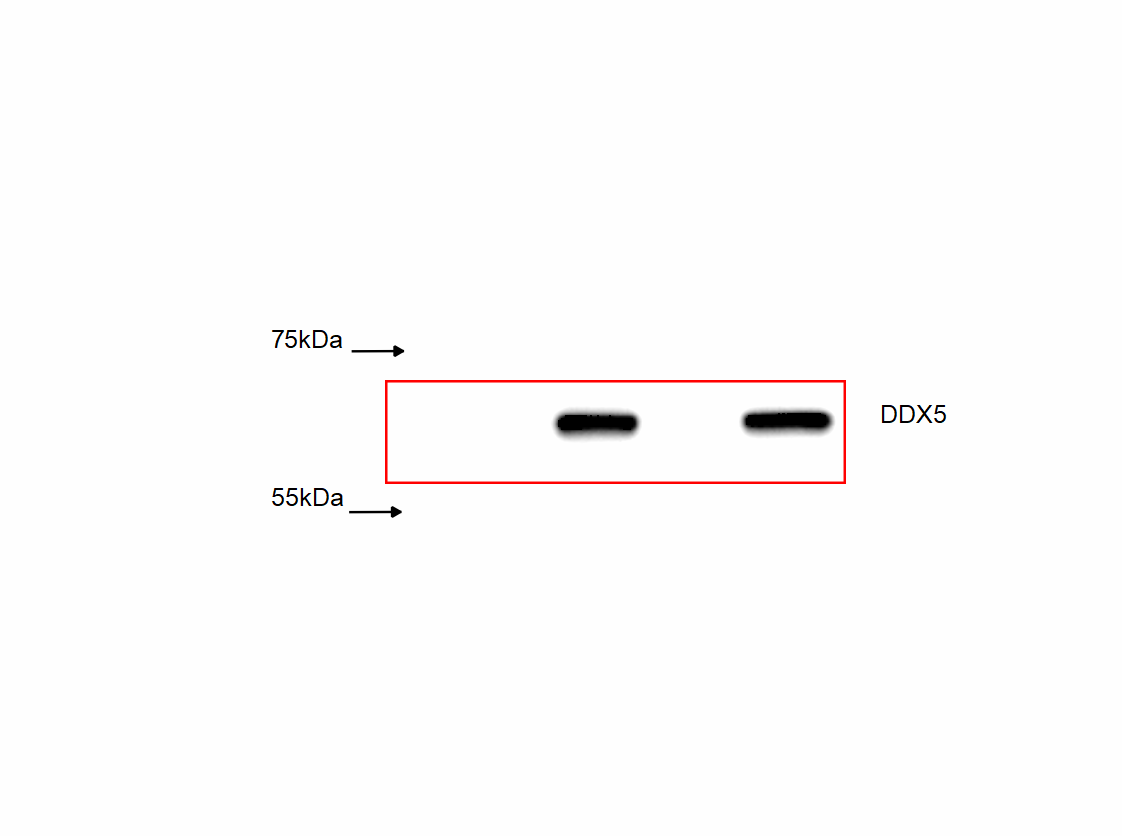

Supplement: Supplementary file 5 — Source Data Fig. 4 [file 44319_2023_47_MOESM5_ESM.zip › EMBOR-2023-57416V3-Figure_4_Source_Data-sd/Figure 4/A/IP-DDX5.tif]

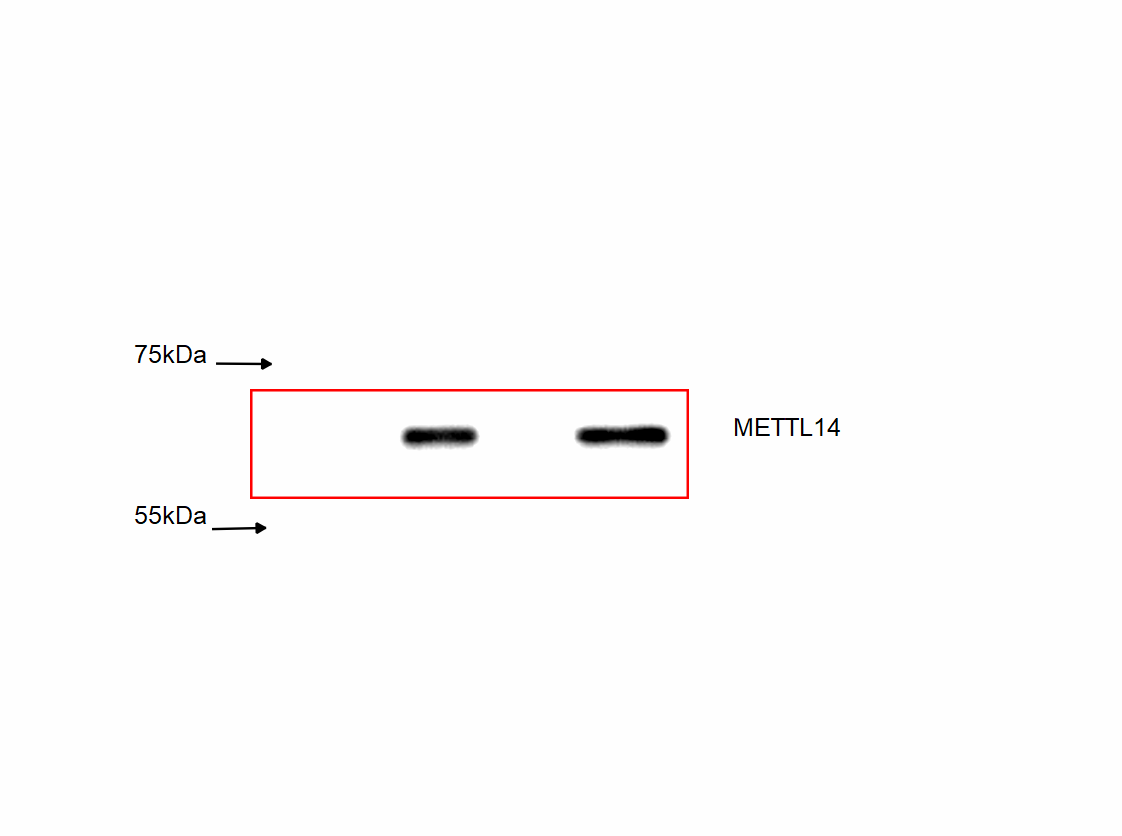

Supplement: Supplementary file 5 — Source Data Fig. 4 [file 44319_2023_47_MOESM5_ESM.zip › EMBOR-2023-57416V3-Figure_4_Source_Data-sd/Figure 4/A/IP-METTL14.tif]

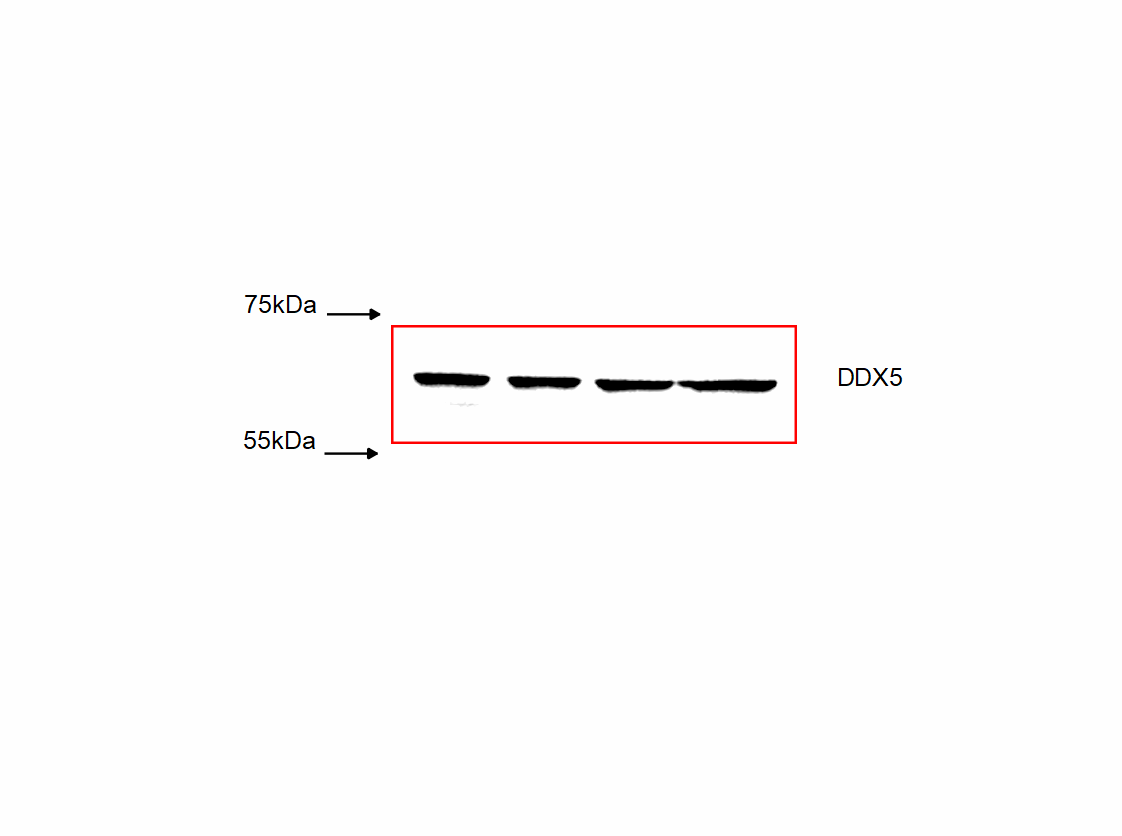

Supplement: Supplementary file 5 — Source Data Fig. 4 [file 44319_2023_47_MOESM5_ESM.zip › EMBOR-2023-57416V3-Figure_4_Source_Data-sd/Figure 4/A/WCL-DDX5.tif]

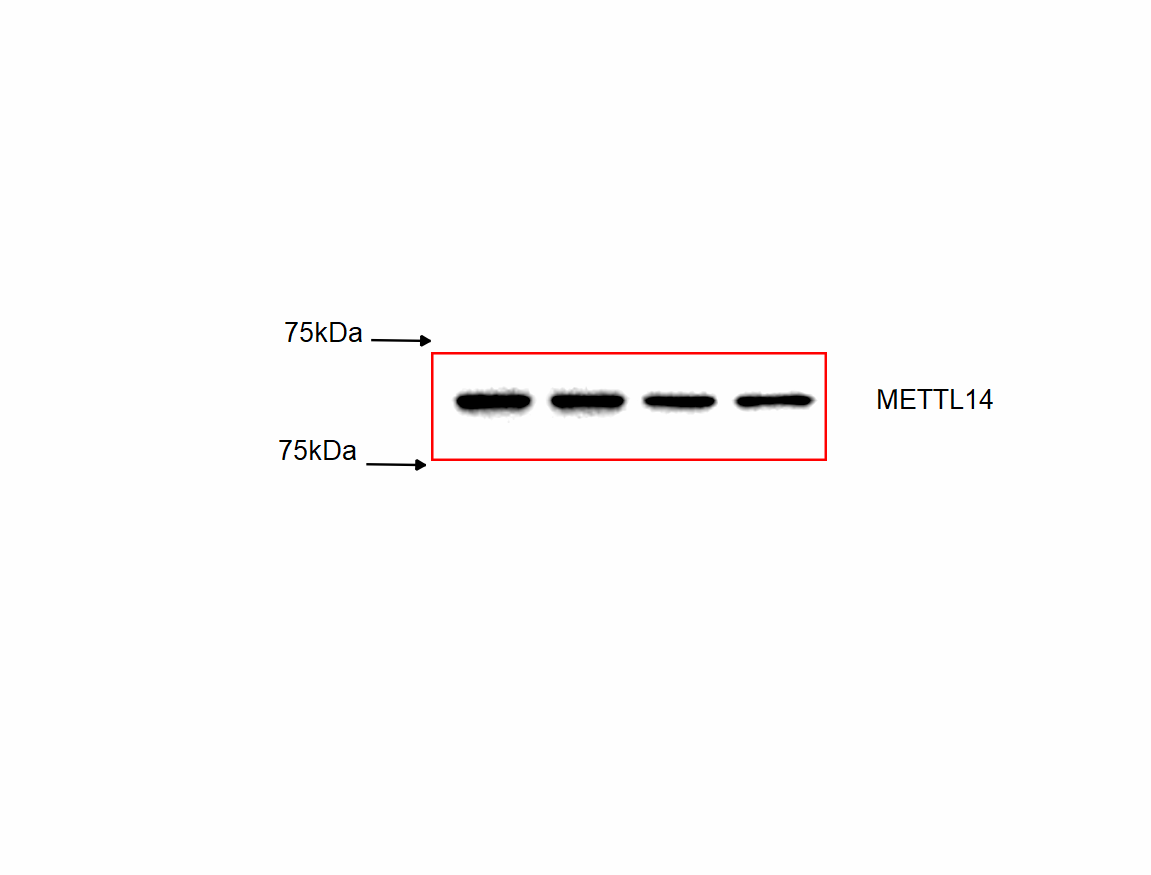

Supplement: Supplementary file 5 — Source Data Fig. 4 [file 44319_2023_47_MOESM5_ESM.zip › EMBOR-2023-57416V3-Figure_4_Source_Data-sd/Figure 4/A/WCL-METTL14.tif]

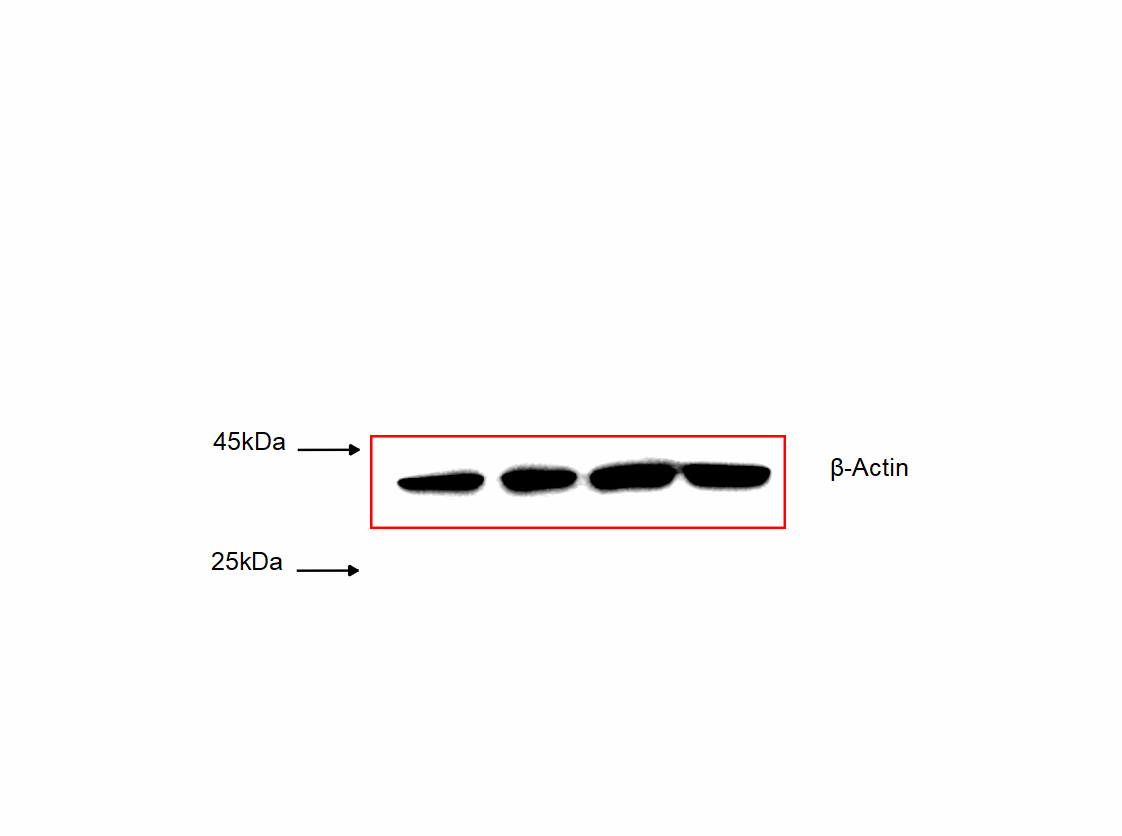

Supplement: Supplementary file 5 — Source Data Fig. 4 [file 44319_2023_47_MOESM5_ESM.zip › EMBOR-2023-57416V3-Figure_4_Source_Data-sd/Figure 4/A/WCL-β-Actin.tif]

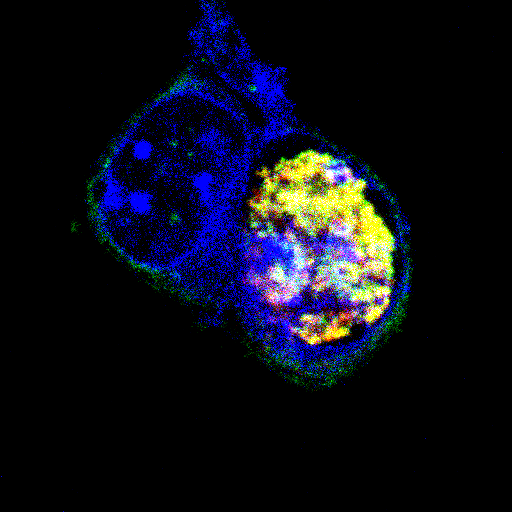

Supplement: Supplementary file 5 — Source Data Fig. 4 [file 44319_2023_47_MOESM5_ESM.zip › EMBOR-2023-57416V3-Figure_4_Source_Data-sd/Figure 4/B/MEFs/Experiment1.11.lif_3_z0.tif]

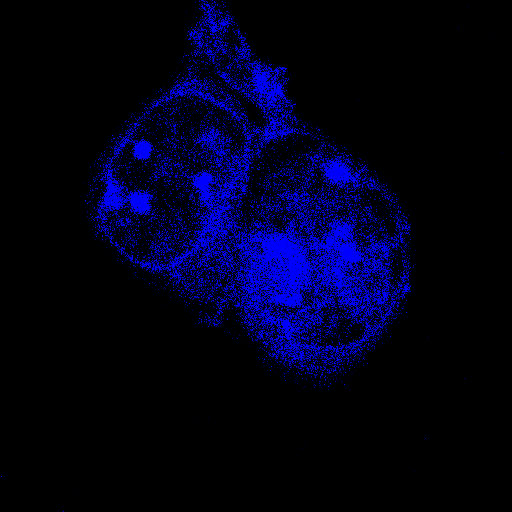

Supplement: Supplementary file 5 — Source Data Fig. 4 [file 44319_2023_47_MOESM5_ESM.zip › EMBOR-2023-57416V3-Figure_4_Source_Data-sd/Figure 4/B/MEFs/Experiment1.11.lif_3_z0_ch00.tif]

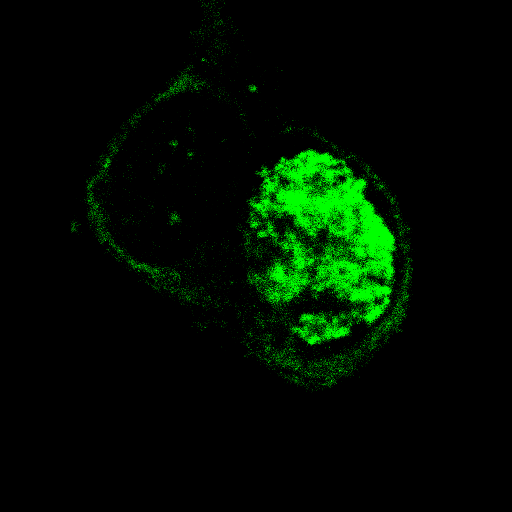

Supplement: Supplementary file 5 — Source Data Fig. 4 [file 44319_2023_47_MOESM5_ESM.zip › EMBOR-2023-57416V3-Figure_4_Source_Data-sd/Figure 4/B/MEFs/Experiment1.11.lif_3_z0_ch01.tif]

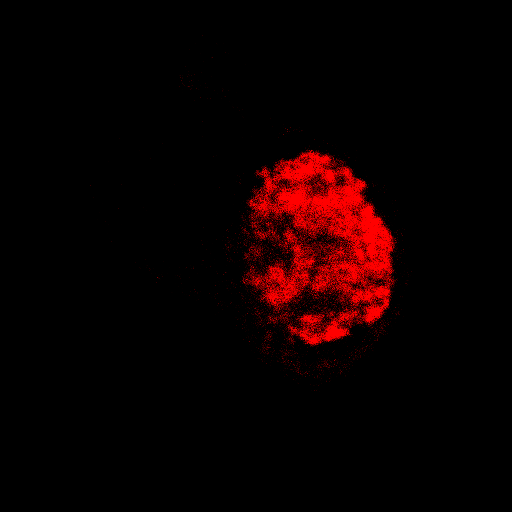

Supplement: Supplementary file 5 — Source Data Fig. 4 [file 44319_2023_47_MOESM5_ESM.zip › EMBOR-2023-57416V3-Figure_4_Source_Data-sd/Figure 4/B/MEFs/Experiment1.11.lif_3_z0_ch02.tif]

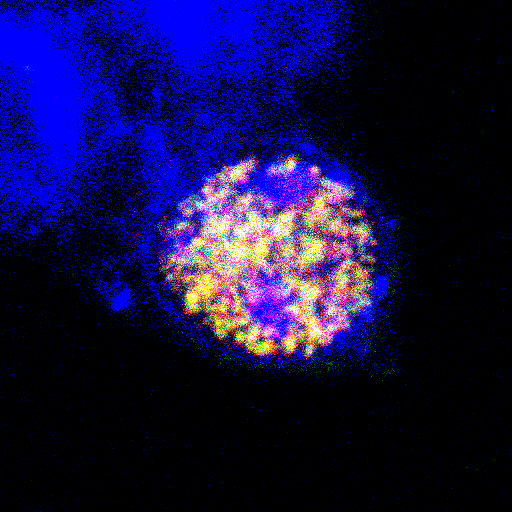

Supplement: Supplementary file 5 — Source Data Fig. 4 [file 44319_2023_47_MOESM5_ESM.zip › EMBOR-2023-57416V3-Figure_4_Source_Data-sd/Figure 4/B/Mø/Experiment1.11.lif_3-2_z0.tif]

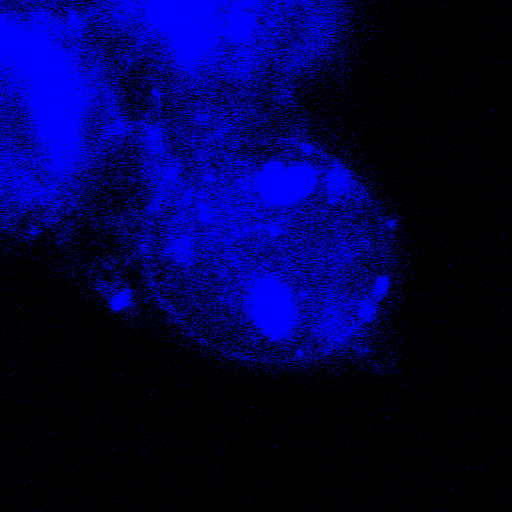

Supplement: Supplementary file 5 — Source Data Fig. 4 [file 44319_2023_47_MOESM5_ESM.zip › EMBOR-2023-57416V3-Figure_4_Source_Data-sd/Figure 4/B/Mø/Experiment1.11.lif_3-2_z0_ch00.tif]

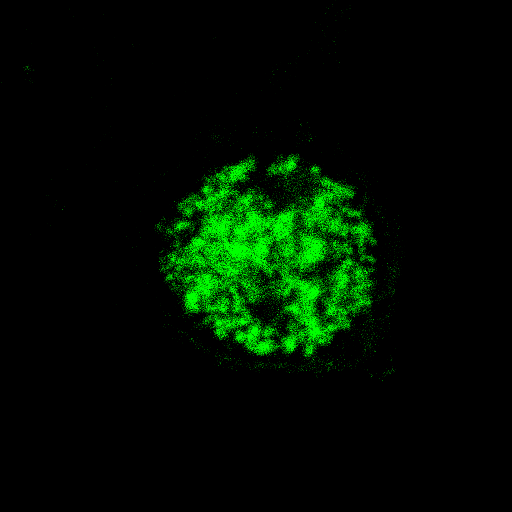

Supplement: Supplementary file 5 — Source Data Fig. 4 [file 44319_2023_47_MOESM5_ESM.zip › EMBOR-2023-57416V3-Figure_4_Source_Data-sd/Figure 4/B/Mø/Experiment1.11.lif_3-2_z0_ch01.tif]

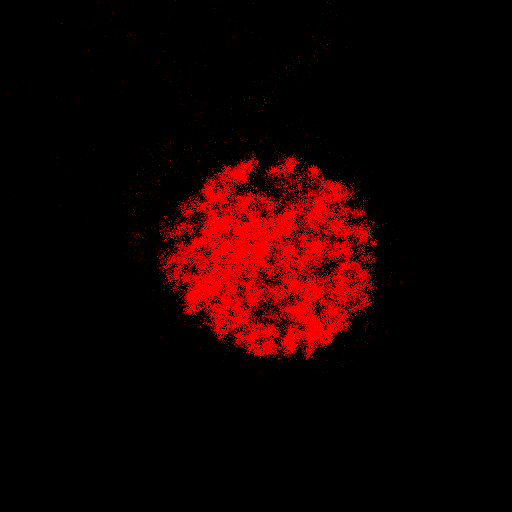

Supplement: Supplementary file 5 — Source Data Fig. 4 [file 44319_2023_47_MOESM5_ESM.zip › EMBOR-2023-57416V3-Figure_4_Source_Data-sd/Figure 4/B/Mø/Experiment1.11.lif_3-2_z0_ch02.tif]

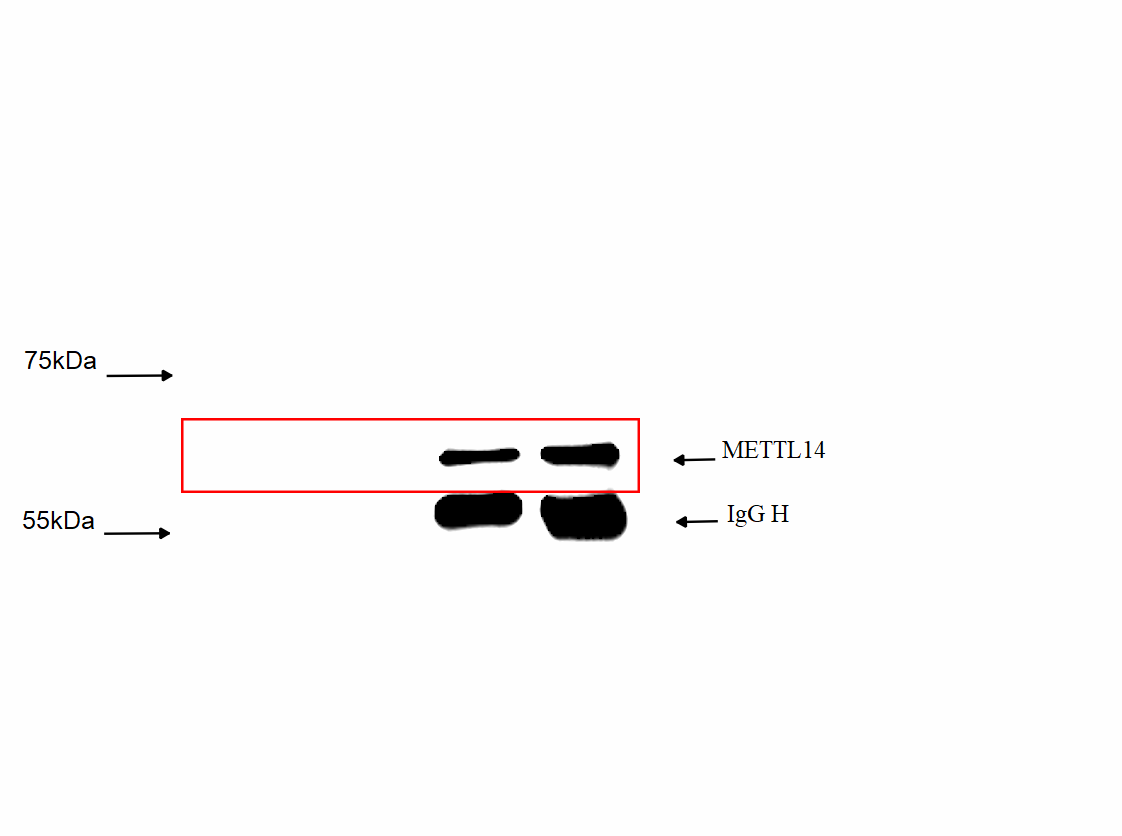

Supplement: Supplementary file 5 — Source Data Fig. 4 [file 44319_2023_47_MOESM5_ESM.zip › EMBOR-2023-57416V3-Figure_4_Source_Data-sd/Figure 4/C/IP-METTL14.tif]

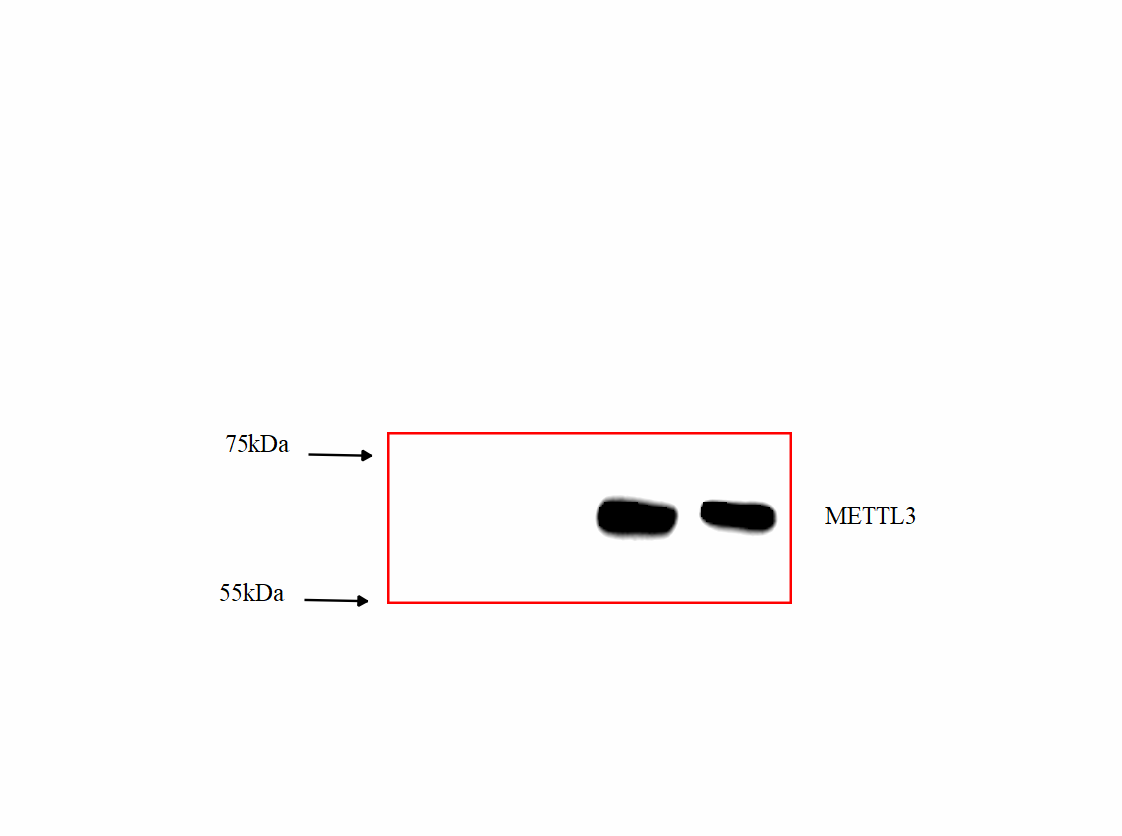

Supplement: Supplementary file 5 — Source Data Fig. 4 [file 44319_2023_47_MOESM5_ESM.zip › EMBOR-2023-57416V3-Figure_4_Source_Data-sd/Figure 4/C/IP-METTL3.tif]

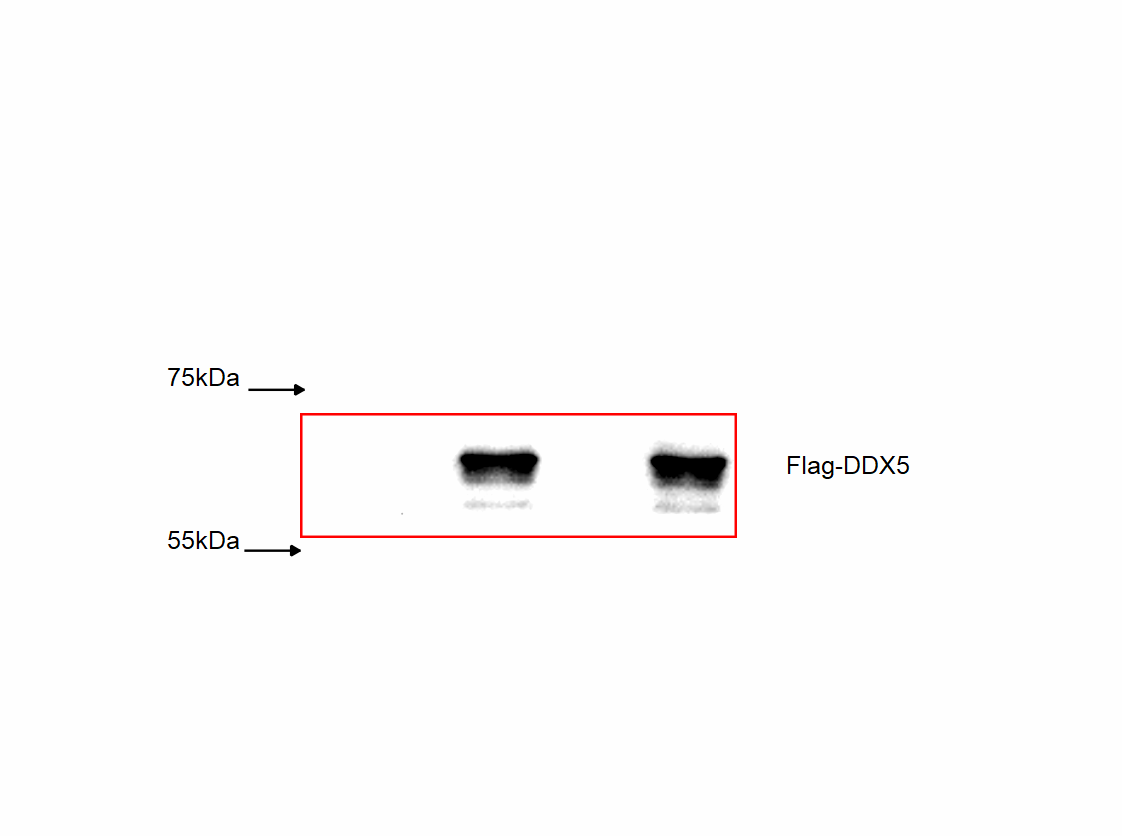

Supplement: Supplementary file 5 — Source Data Fig. 4 [file 44319_2023_47_MOESM5_ESM.zip › EMBOR-2023-57416V3-Figure_4_Source_Data-sd/Figure 4/C/WCL-Flag-DDX5.tif]

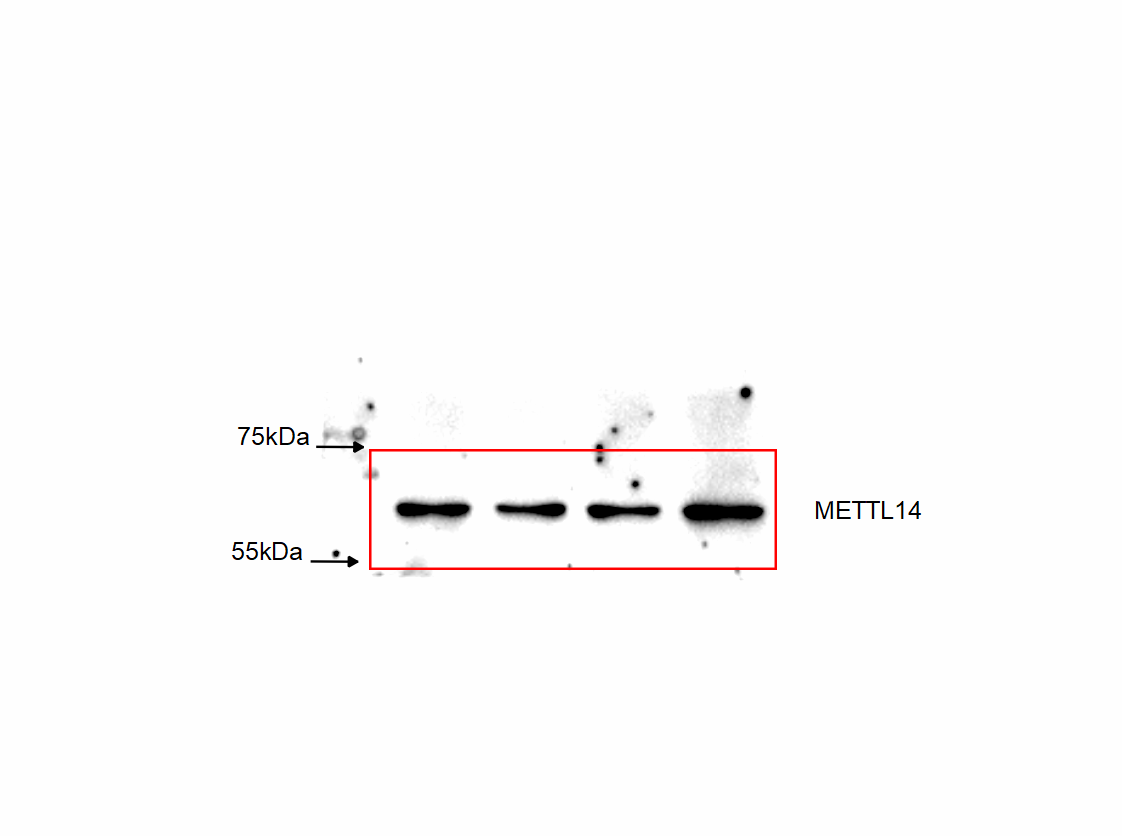

Supplement: Supplementary file 5 — Source Data Fig. 4 [file 44319_2023_47_MOESM5_ESM.zip › EMBOR-2023-57416V3-Figure_4_Source_Data-sd/Figure 4/C/WCL-METTL14.tif]

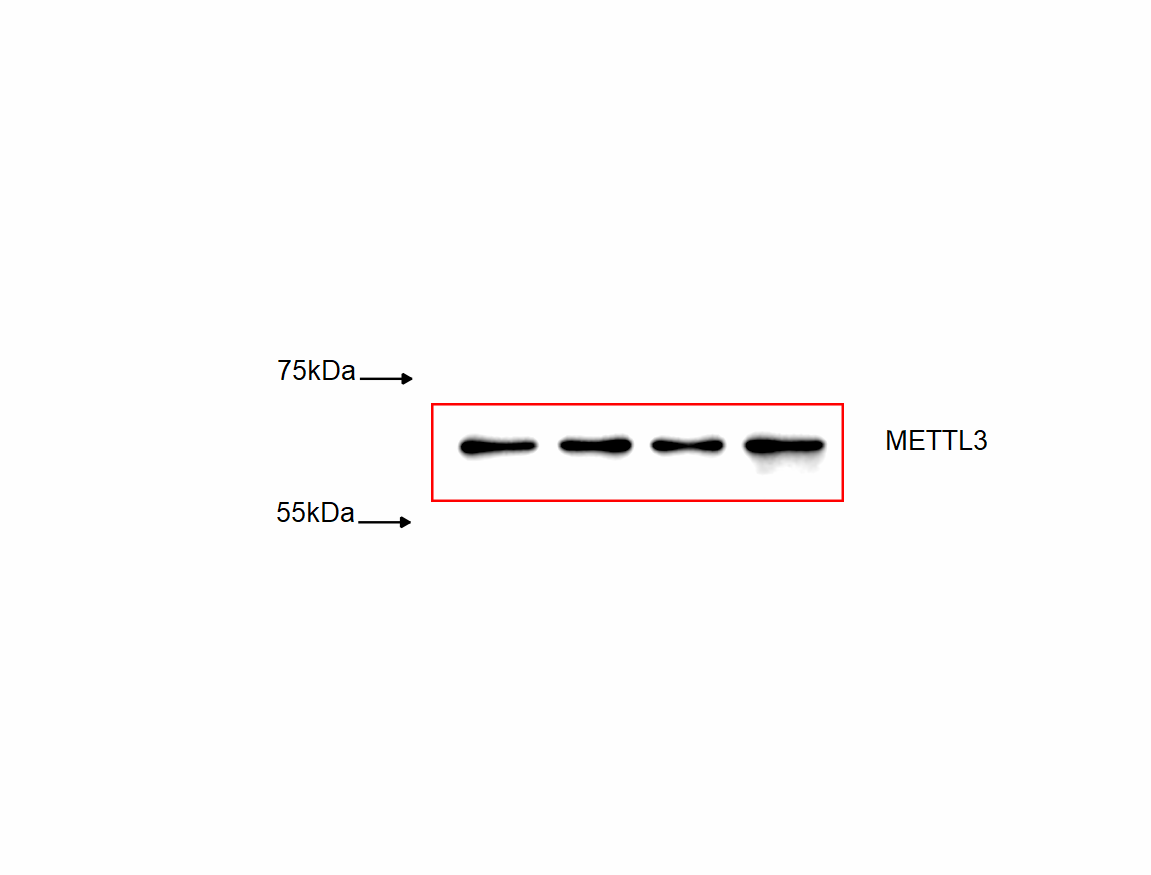

Supplement: Supplementary file 5 — Source Data Fig. 4 [file 44319_2023_47_MOESM5_ESM.zip › EMBOR-2023-57416V3-Figure_4_Source_Data-sd/Figure 4/C/WCL-METTL3.tif]

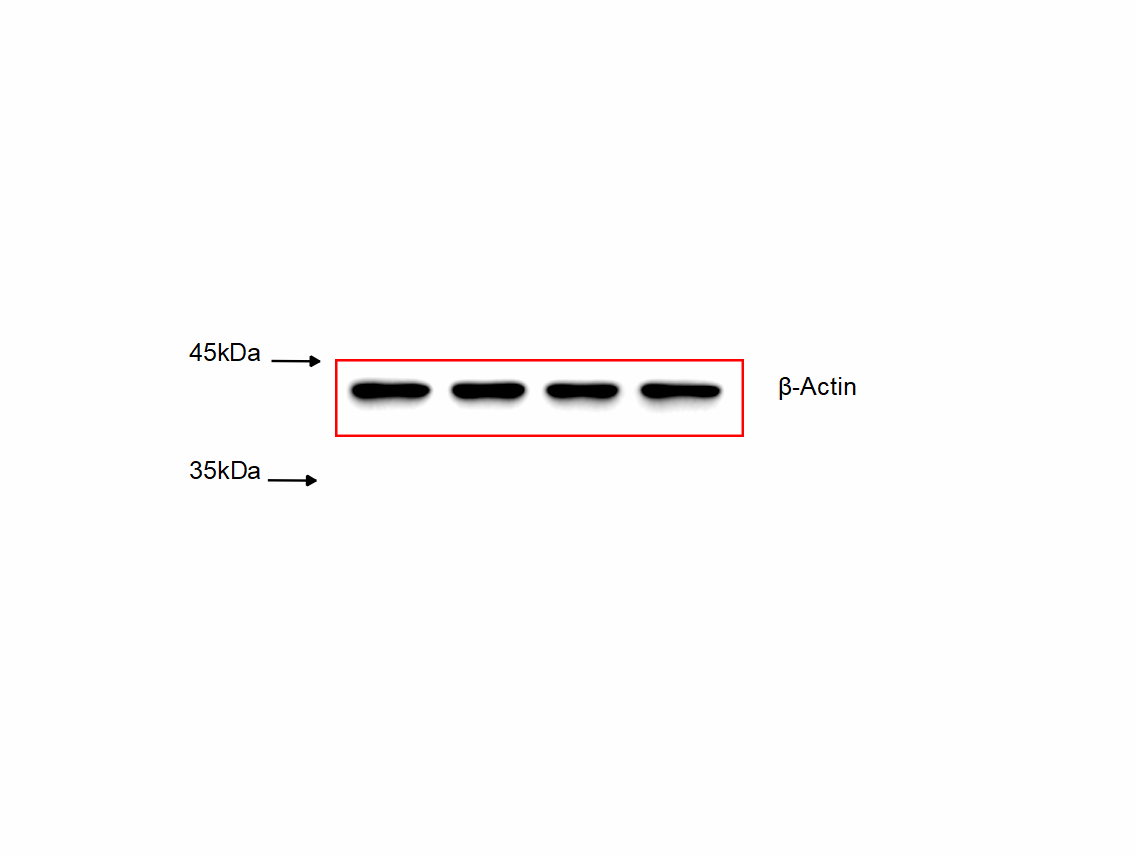

Supplement: Supplementary file 5 — Source Data Fig. 4 [file 44319_2023_47_MOESM5_ESM.zip › EMBOR-2023-57416V3-Figure_4_Source_Data-sd/Figure 4/C/WCL-β-Actin.tif]

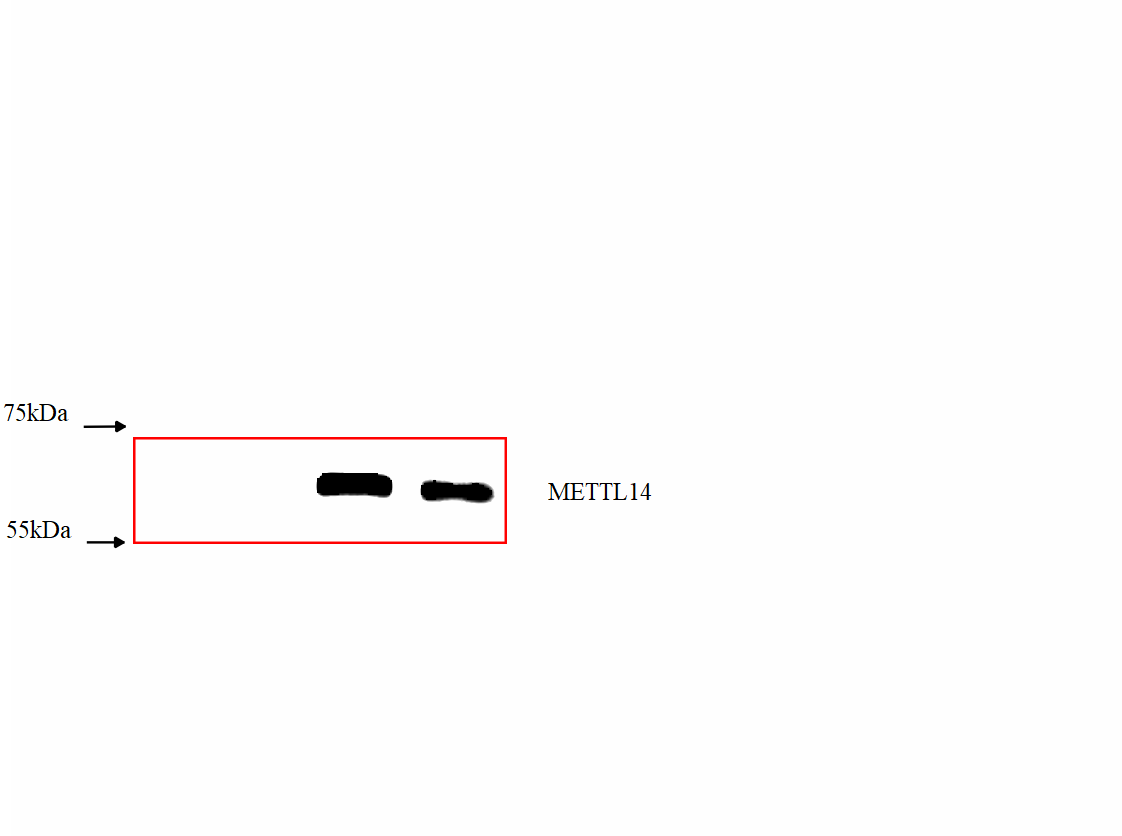

Supplement: Supplementary file 5 — Source Data Fig. 4 [file 44319_2023_47_MOESM5_ESM.zip › EMBOR-2023-57416V3-Figure_4_Source_Data-sd/Figure 4/D/IP-METTL14.tif]

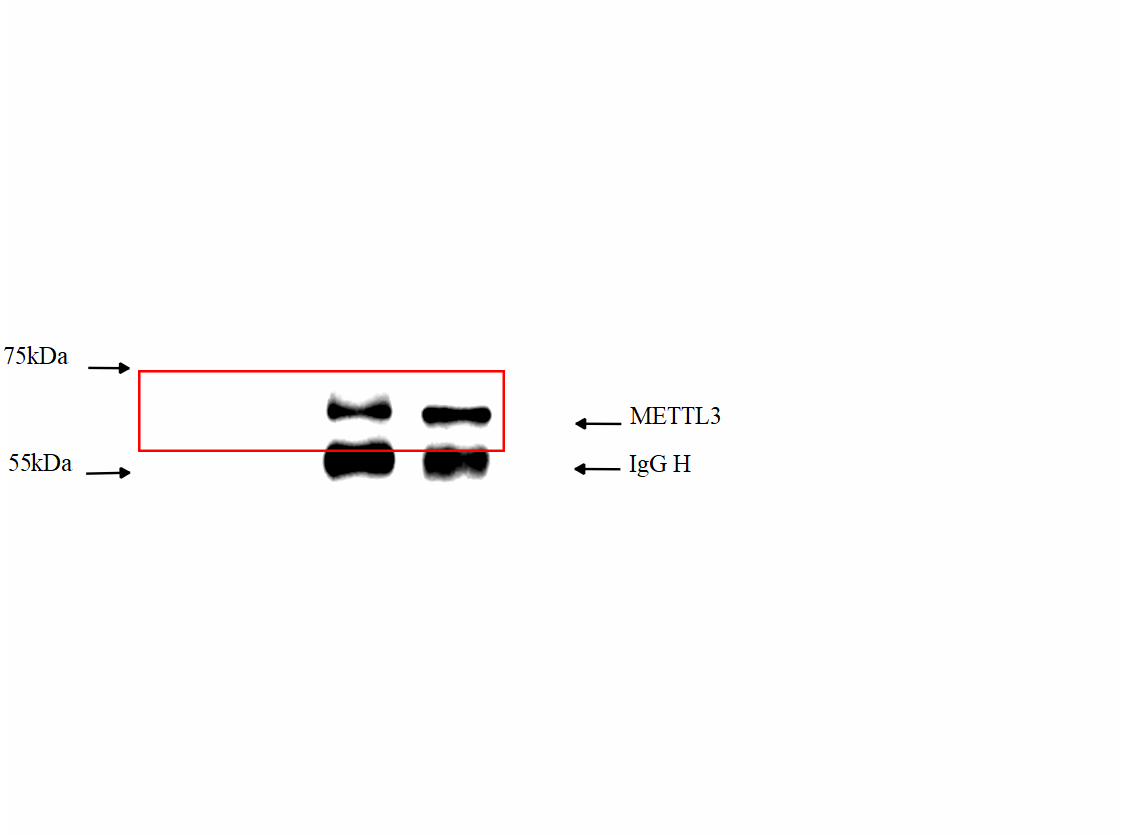

Supplement: Supplementary file 5 — Source Data Fig. 4 [file 44319_2023_47_MOESM5_ESM.zip › EMBOR-2023-57416V3-Figure_4_Source_Data-sd/Figure 4/D/IP-METTL3.tif]

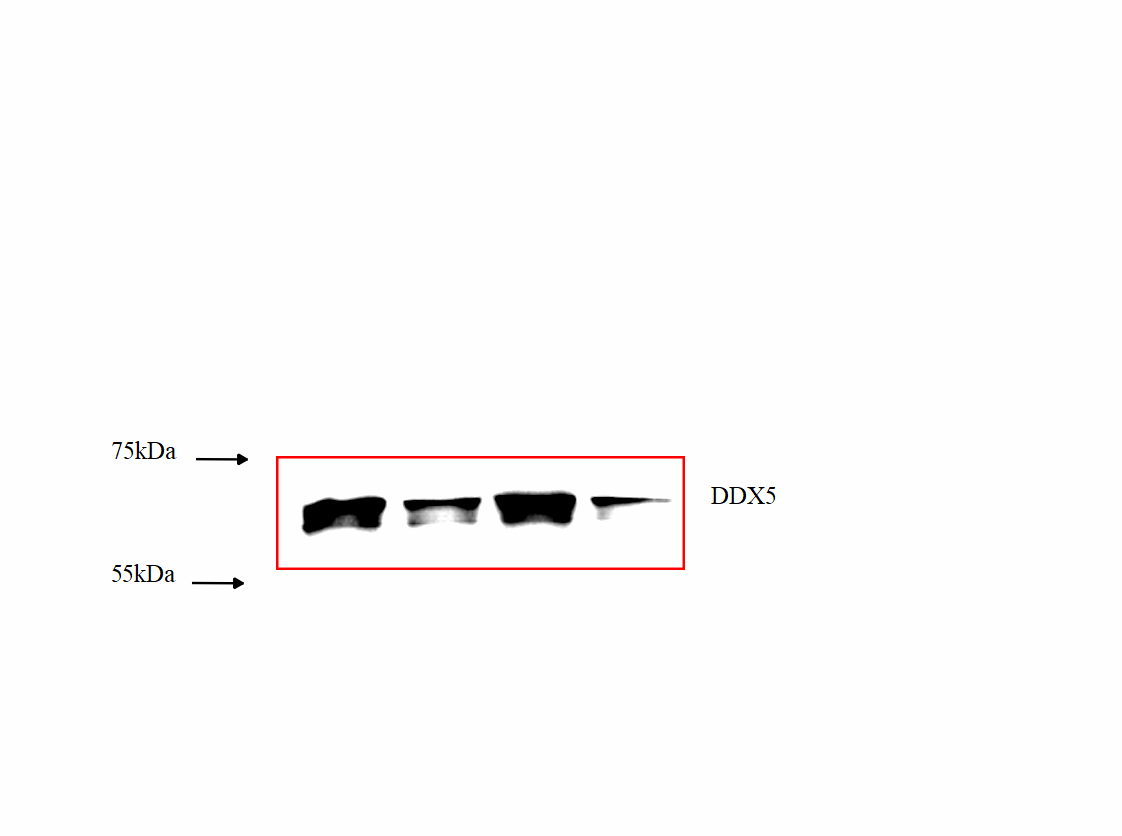

Supplement: Supplementary file 5 — Source Data Fig. 4 [file 44319_2023_47_MOESM5_ESM.zip › EMBOR-2023-57416V3-Figure_4_Source_Data-sd/Figure 4/D/WCL-DDX5.tif]

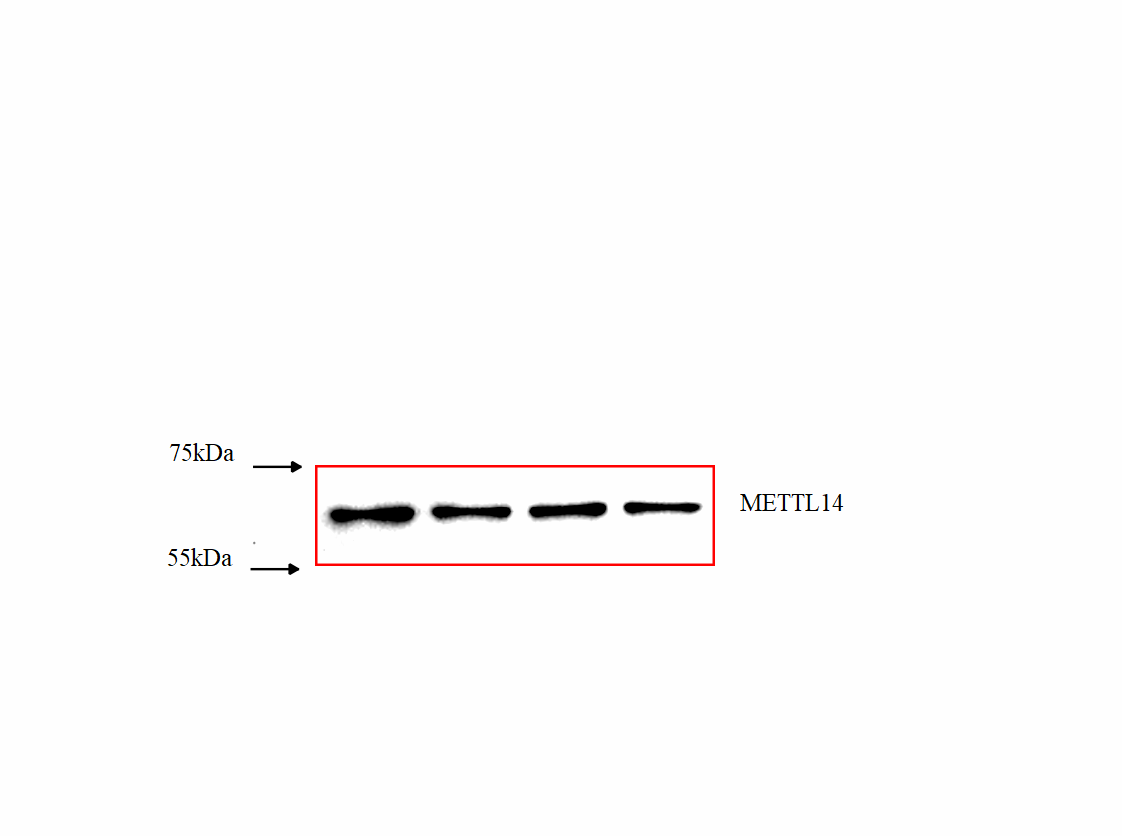

Supplement: Supplementary file 5 — Source Data Fig. 4 [file 44319_2023_47_MOESM5_ESM.zip › EMBOR-2023-57416V3-Figure_4_Source_Data-sd/Figure 4/D/WCL-METTL14.tif]

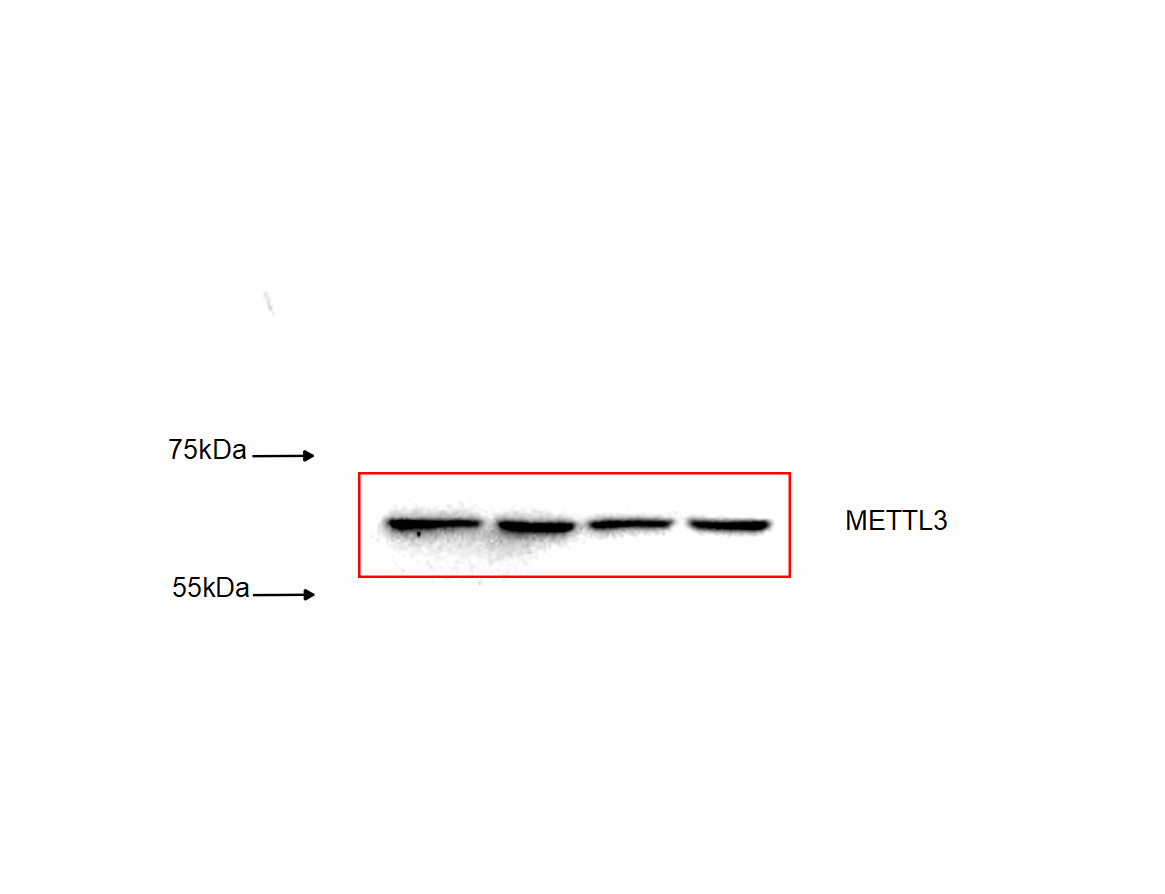

Supplement: Supplementary file 5 — Source Data Fig. 4 [file 44319_2023_47_MOESM5_ESM.zip › EMBOR-2023-57416V3-Figure_4_Source_Data-sd/Figure 4/D/WCL-METTL3.tif]
