# Supplementary figures and images for: DDX5 inhibits inflammation by modulating m6A levels of TLR2/4 transcripts during bacterial infection (part 2 of 2)
Source: EMBO Rep. 2024 Jan 5;25(2):19. doi: 10.1038/s44319-023-00047-9 (PMC10897170; doi:10.1038/s44319-023-00047-9)

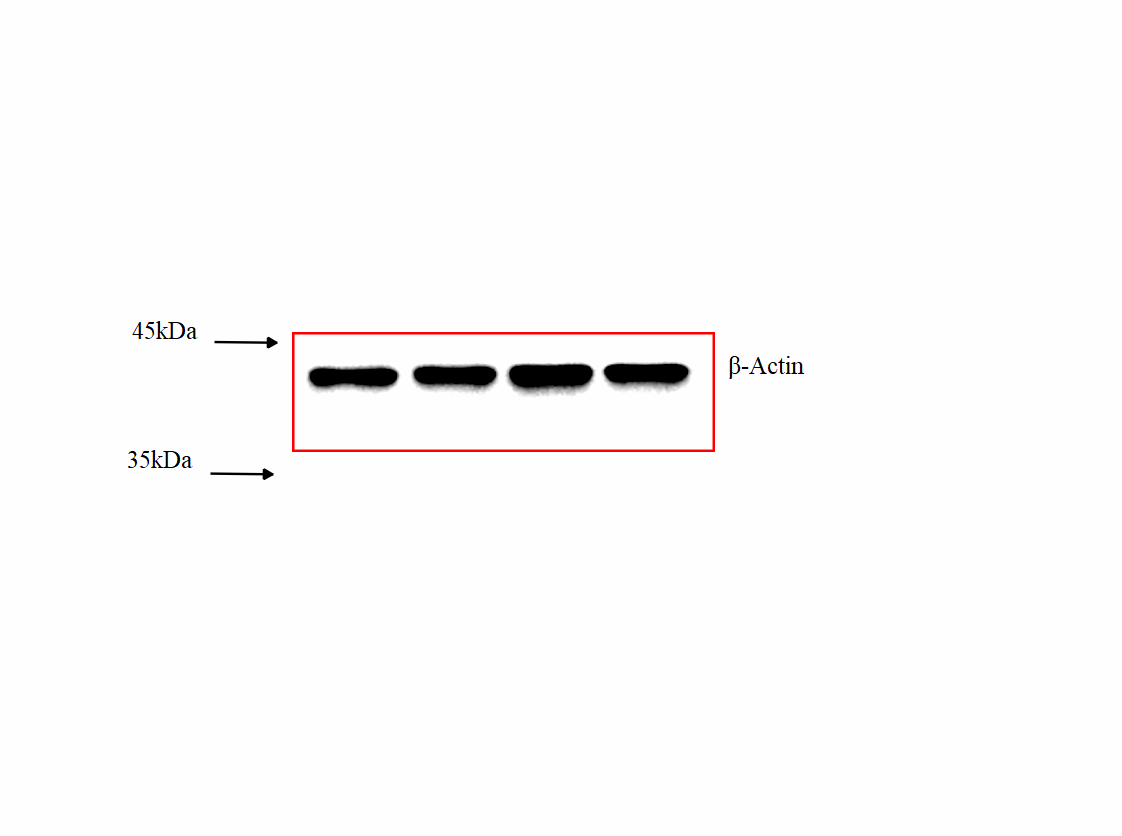

Supplement: Supplementary file 5 — Source Data Fig. 4 [file 44319_2023_47_MOESM5_ESM.zip › EMBOR-2023-57416V3-Figure_4_Source_Data-sd/Figure 4/D/WCL-β-Actin.tif]

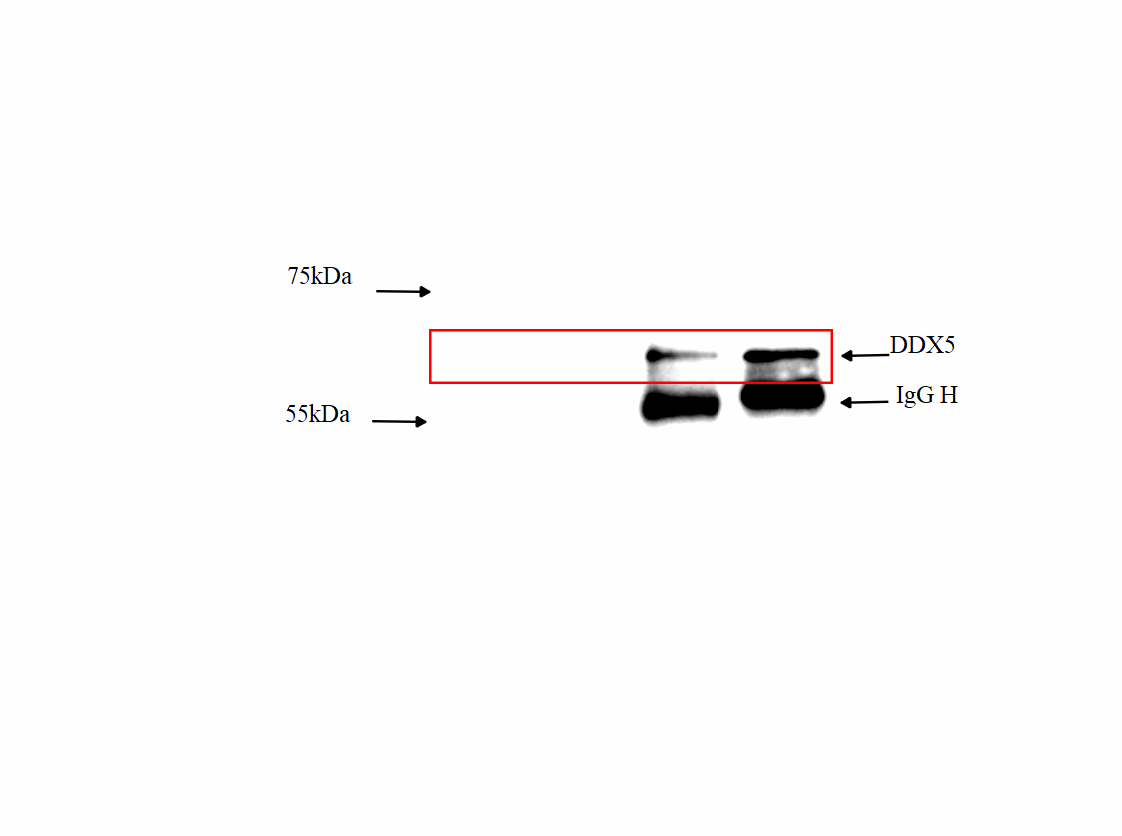

Supplement: Supplementary file 5 — Source Data Fig. 4 [file 44319_2023_47_MOESM5_ESM.zip › EMBOR-2023-57416V3-Figure_4_Source_Data-sd/Figure 4/E/IP-DDX5.tif]

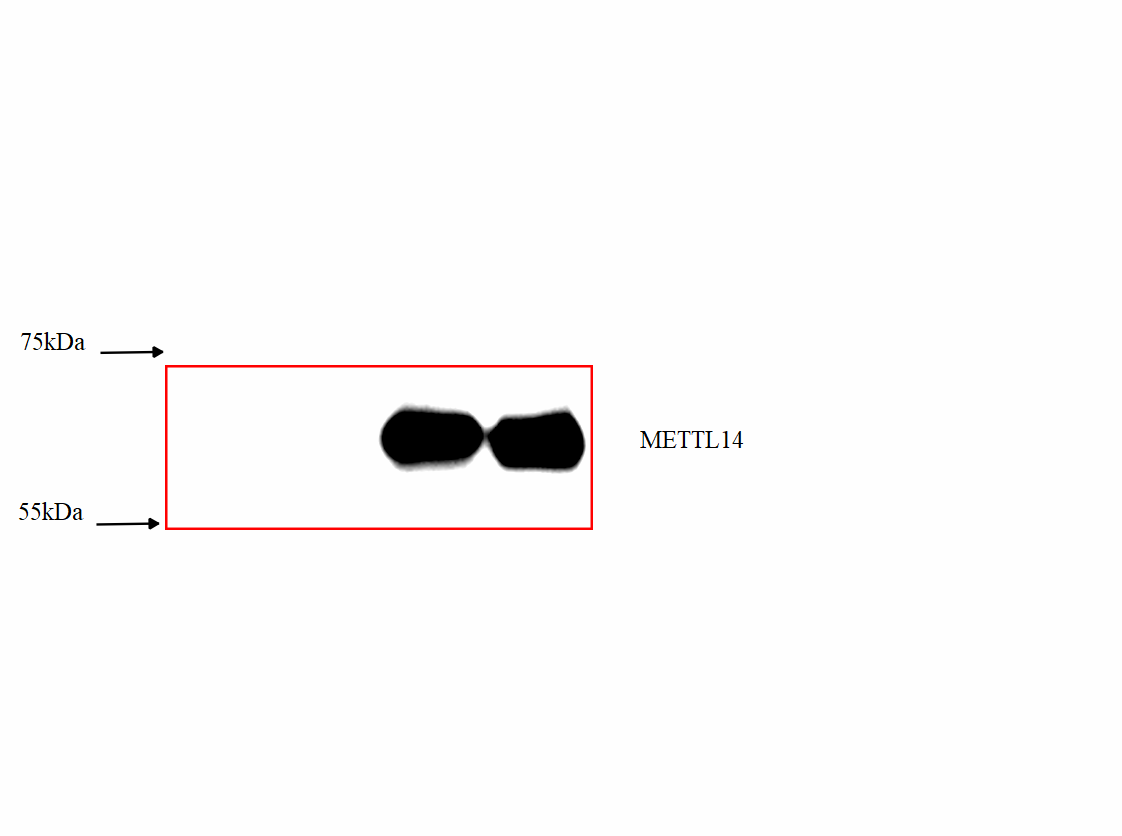

Supplement: Supplementary file 5 — Source Data Fig. 4 [file 44319_2023_47_MOESM5_ESM.zip › EMBOR-2023-57416V3-Figure_4_Source_Data-sd/Figure 4/E/IP-METTL14.tif]

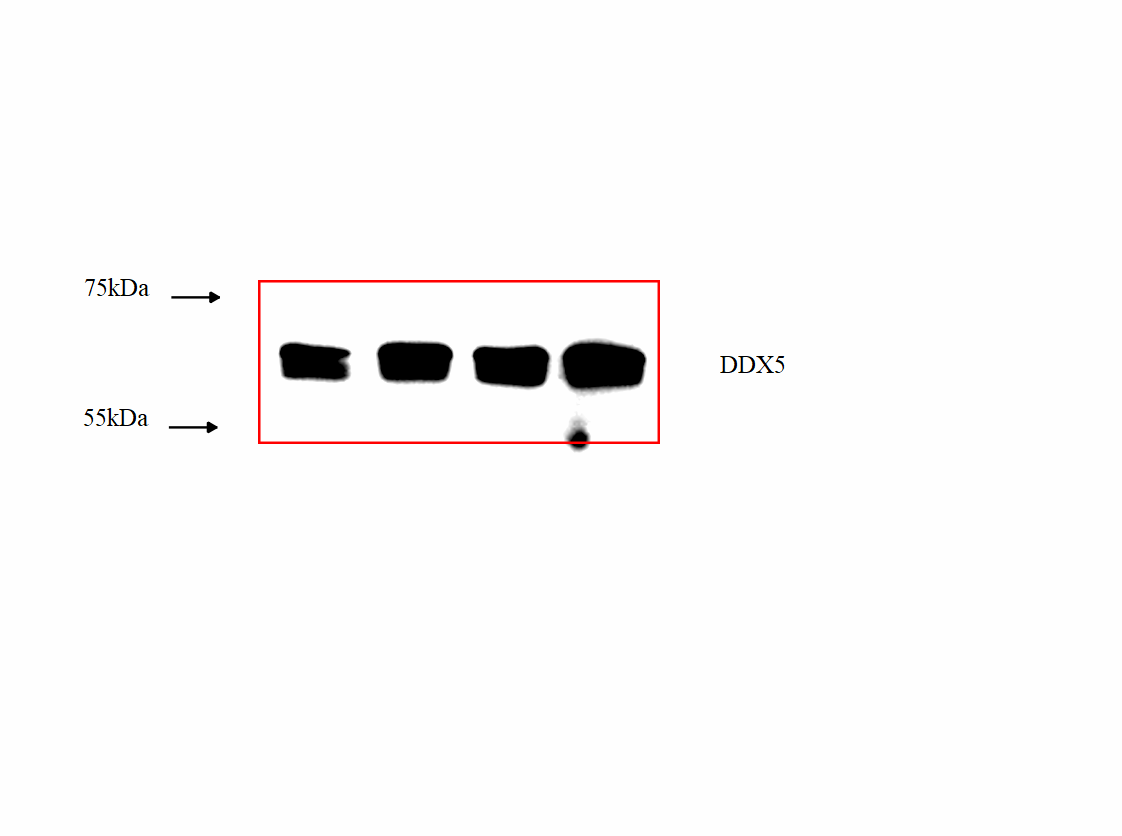

Supplement: Supplementary file 5 — Source Data Fig. 4 [file 44319_2023_47_MOESM5_ESM.zip › EMBOR-2023-57416V3-Figure_4_Source_Data-sd/Figure 4/E/WCL-DDX5.tif]

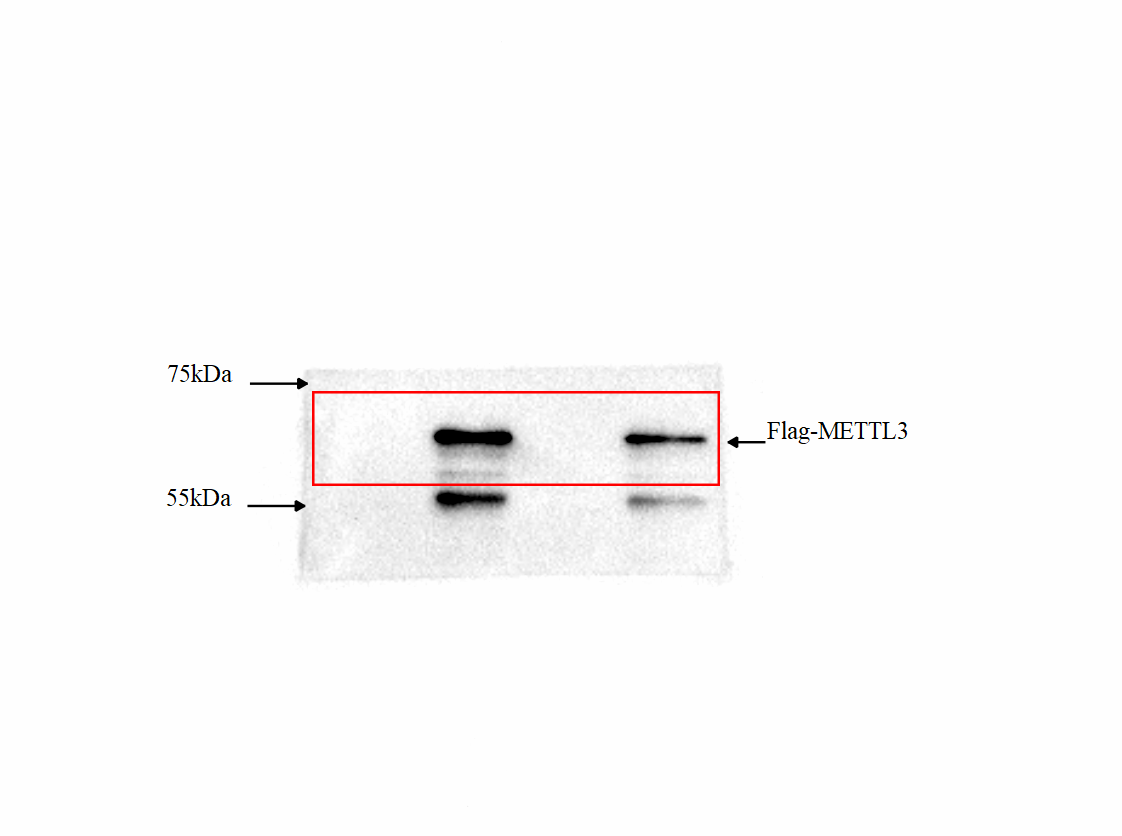

Supplement: Supplementary file 5 — Source Data Fig. 4 [file 44319_2023_47_MOESM5_ESM.zip › EMBOR-2023-57416V3-Figure_4_Source_Data-sd/Figure 4/E/WCL-Flag-METTL3.tif]

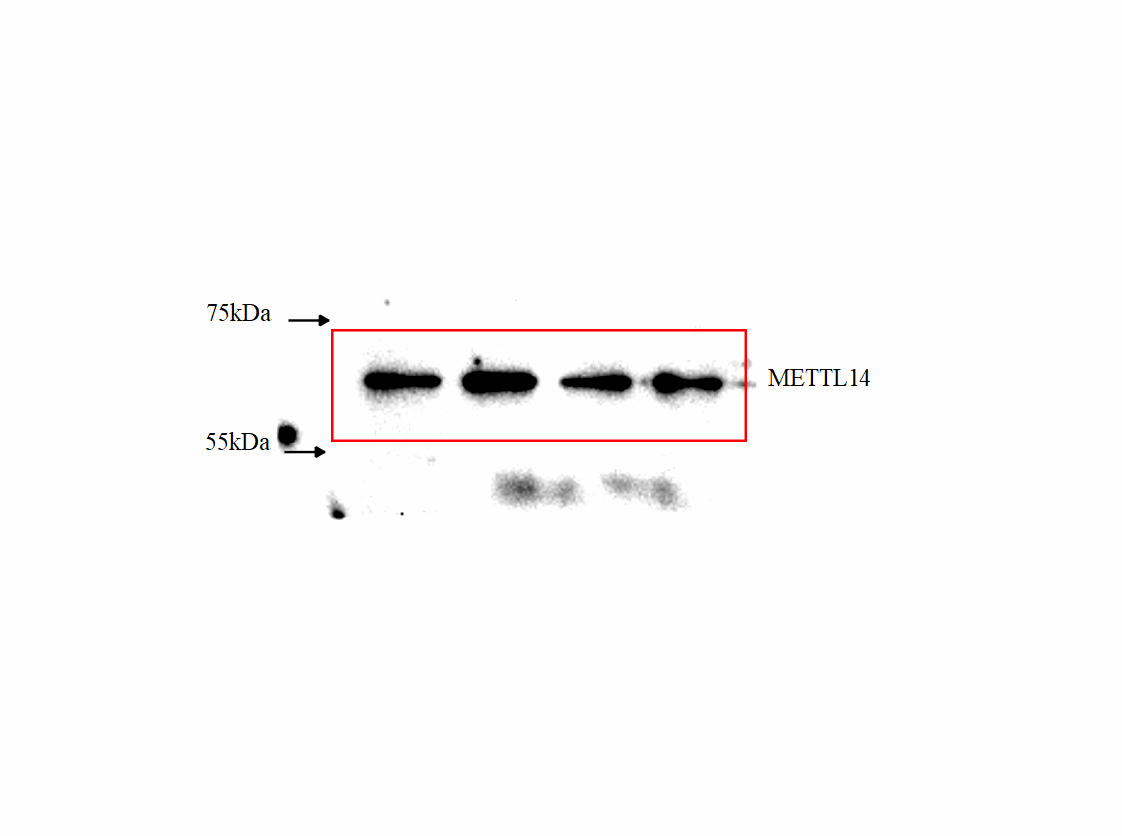

Supplement: Supplementary file 5 — Source Data Fig. 4 [file 44319_2023_47_MOESM5_ESM.zip › EMBOR-2023-57416V3-Figure_4_Source_Data-sd/Figure 4/E/WCL-METTL14.tif]

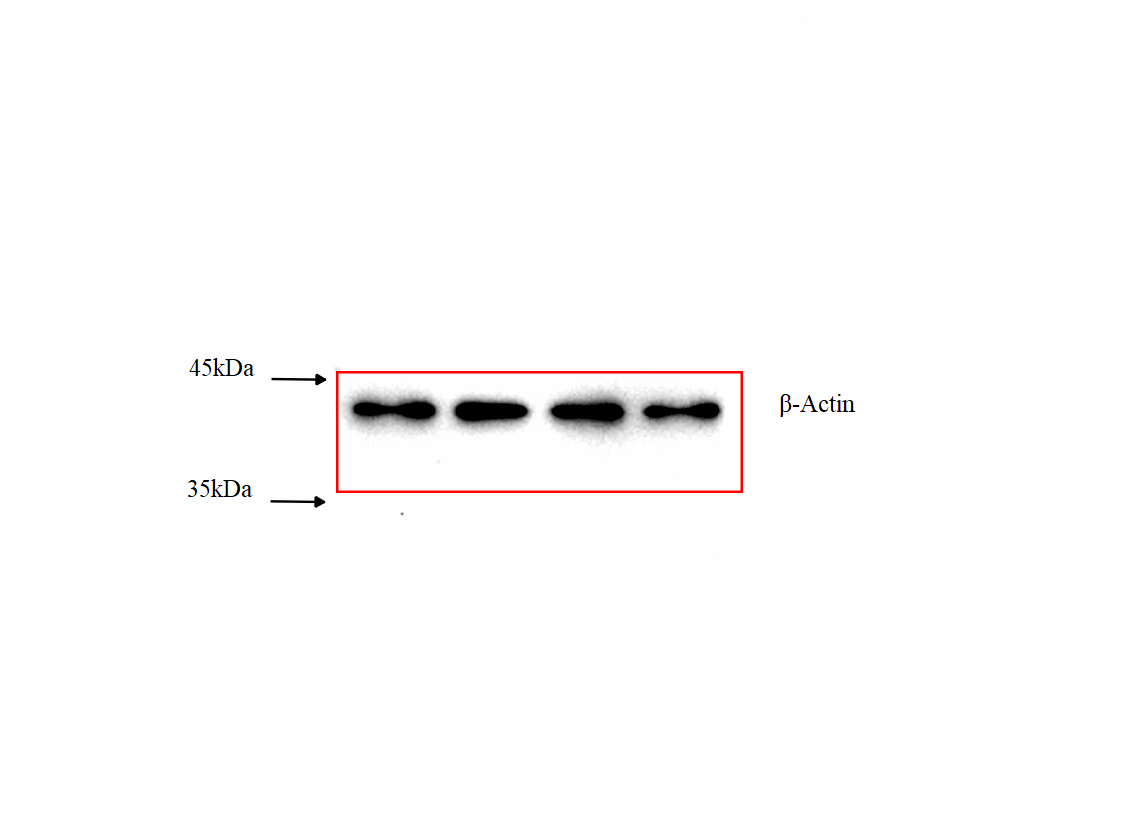

Supplement: Supplementary file 5 — Source Data Fig. 4 [file 44319_2023_47_MOESM5_ESM.zip › EMBOR-2023-57416V3-Figure_4_Source_Data-sd/Figure 4/E/WCL-β-Actin.tif]

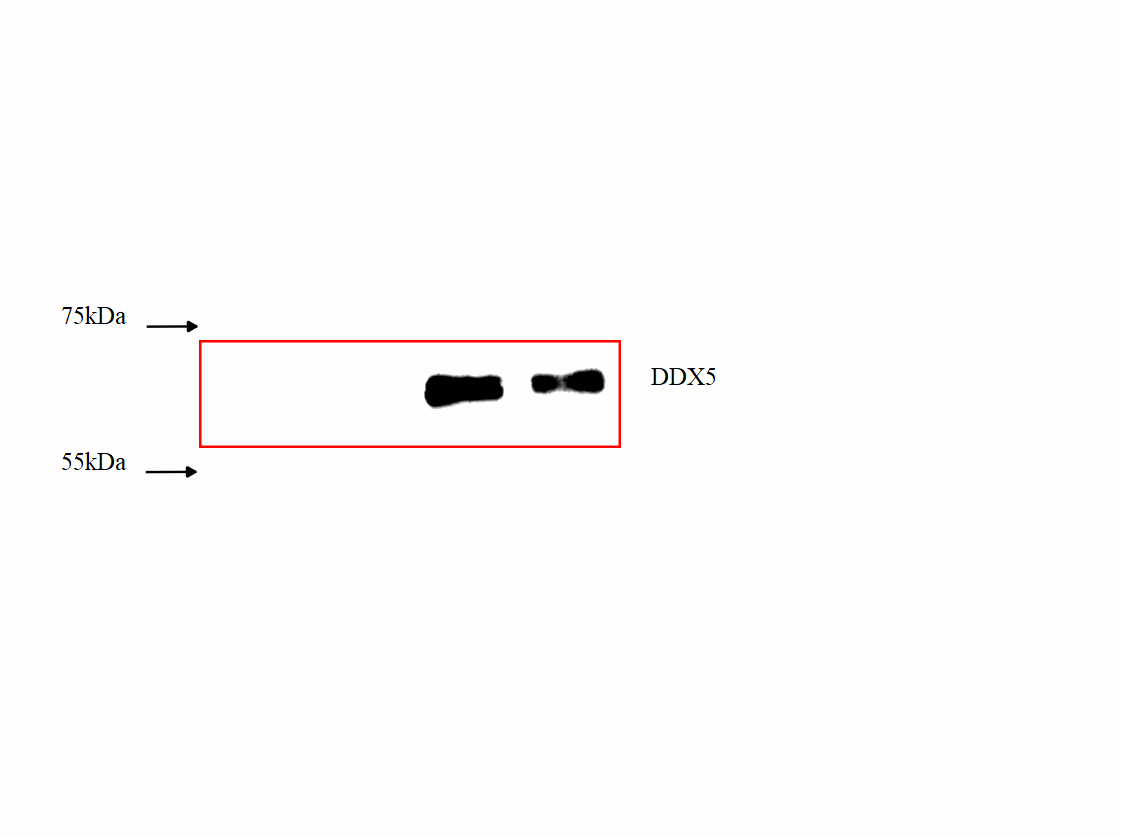

Supplement: Supplementary file 5 — Source Data Fig. 4 [file 44319_2023_47_MOESM5_ESM.zip › EMBOR-2023-57416V3-Figure_4_Source_Data-sd/Figure 4/F/IP-DDX5.tif]

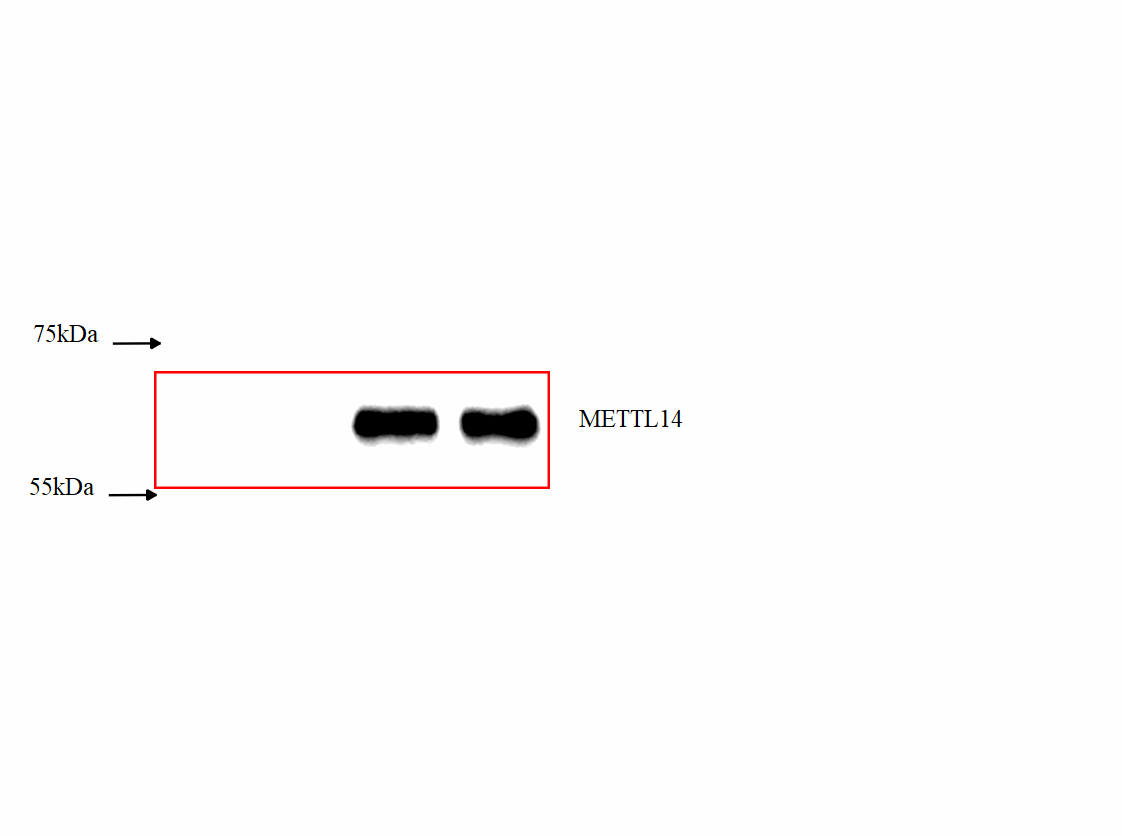

Supplement: Supplementary file 5 — Source Data Fig. 4 [file 44319_2023_47_MOESM5_ESM.zip › EMBOR-2023-57416V3-Figure_4_Source_Data-sd/Figure 4/F/IP-METTL14.tif]

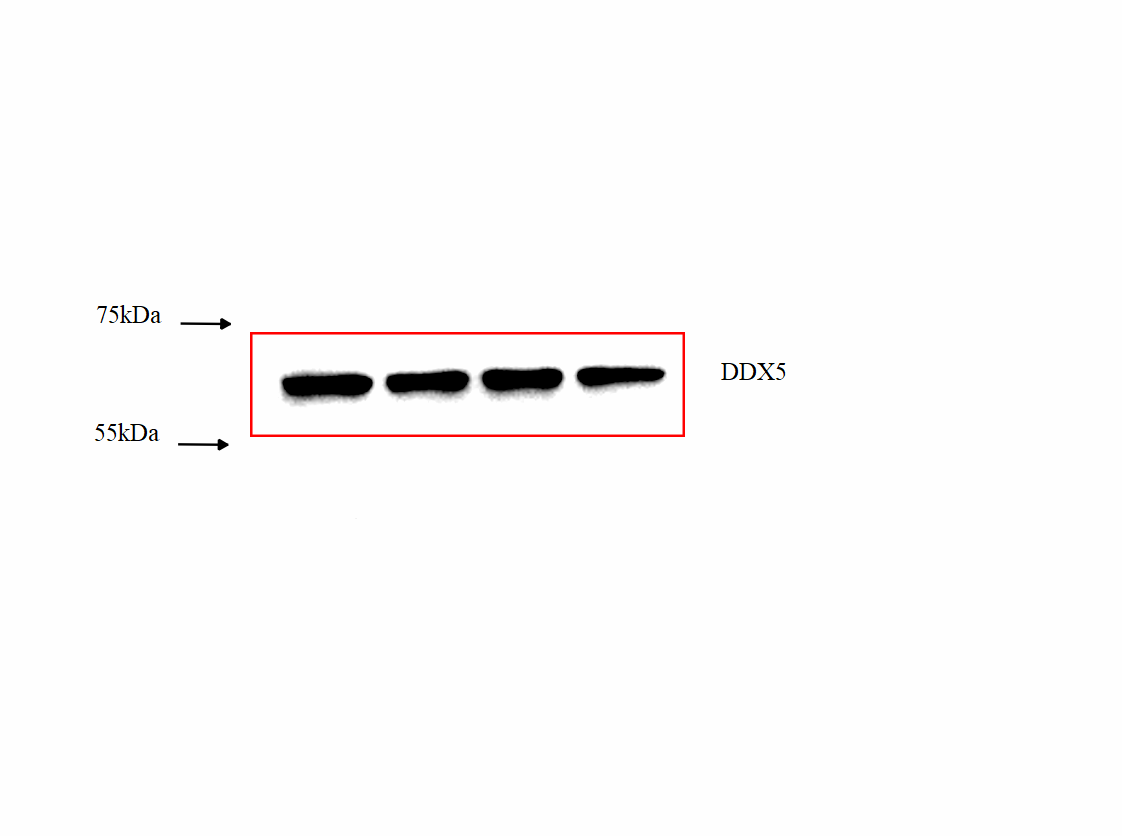

Supplement: Supplementary file 5 — Source Data Fig. 4 [file 44319_2023_47_MOESM5_ESM.zip › EMBOR-2023-57416V3-Figure_4_Source_Data-sd/Figure 4/F/WCL-DDX5.tif]

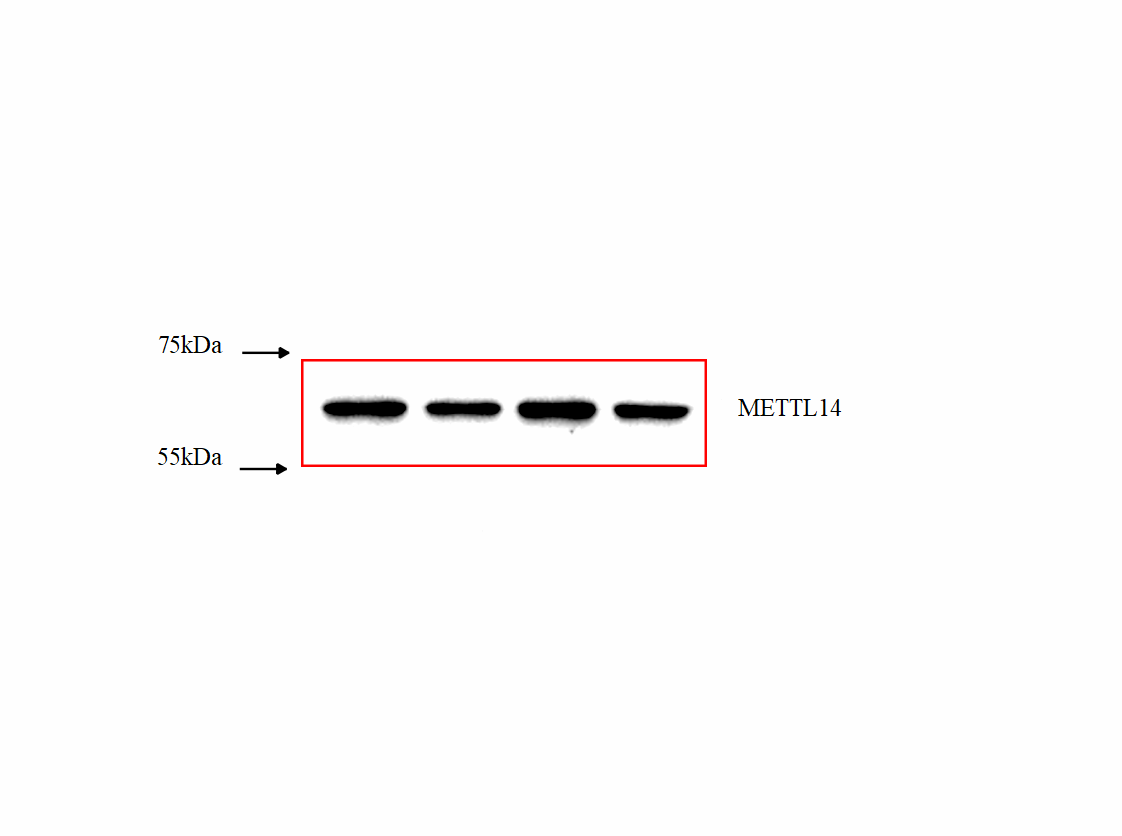

Supplement: Supplementary file 5 — Source Data Fig. 4 [file 44319_2023_47_MOESM5_ESM.zip › EMBOR-2023-57416V3-Figure_4_Source_Data-sd/Figure 4/F/WCL-METTL14.tif]

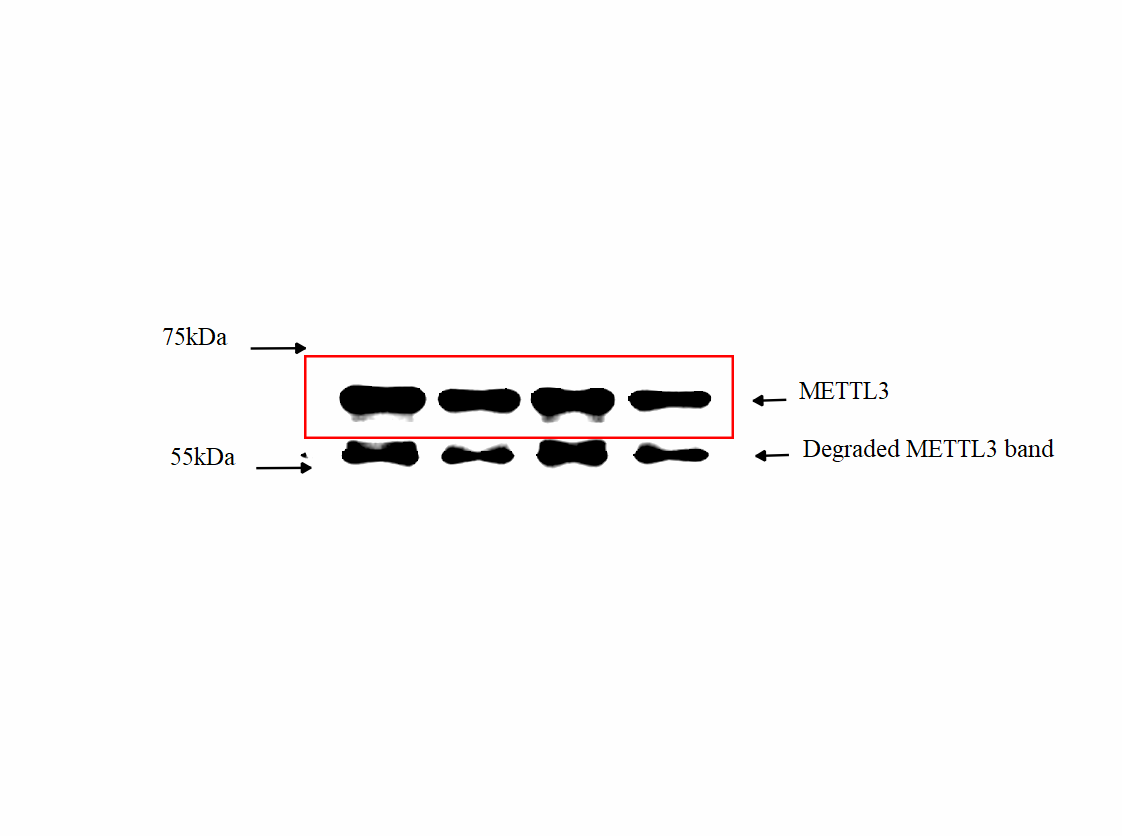

Supplement: Supplementary file 5 — Source Data Fig. 4 [file 44319_2023_47_MOESM5_ESM.zip › EMBOR-2023-57416V3-Figure_4_Source_Data-sd/Figure 4/F/WCL-METTL3.tif]

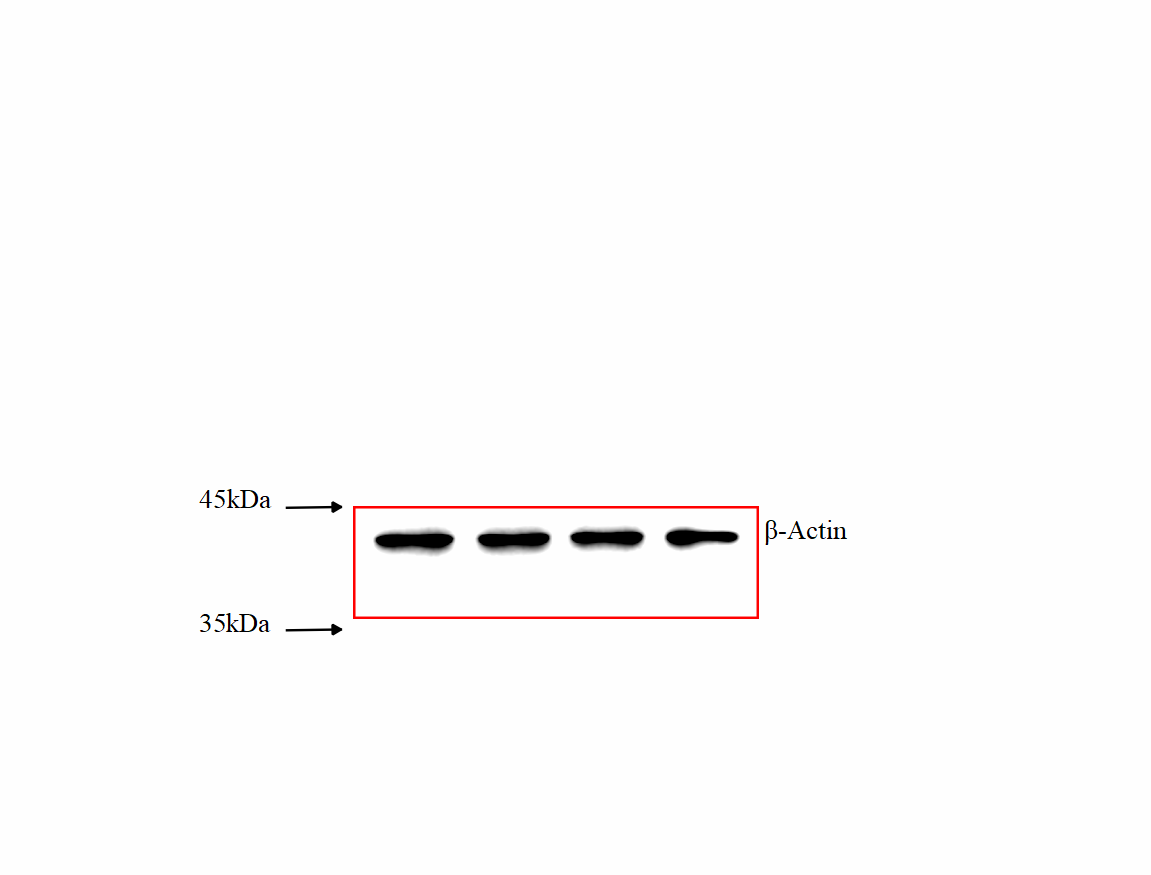

Supplement: Supplementary file 5 — Source Data Fig. 4 [file 44319_2023_47_MOESM5_ESM.zip › EMBOR-2023-57416V3-Figure_4_Source_Data-sd/Figure 4/F/WCL-β-Actin.tif]

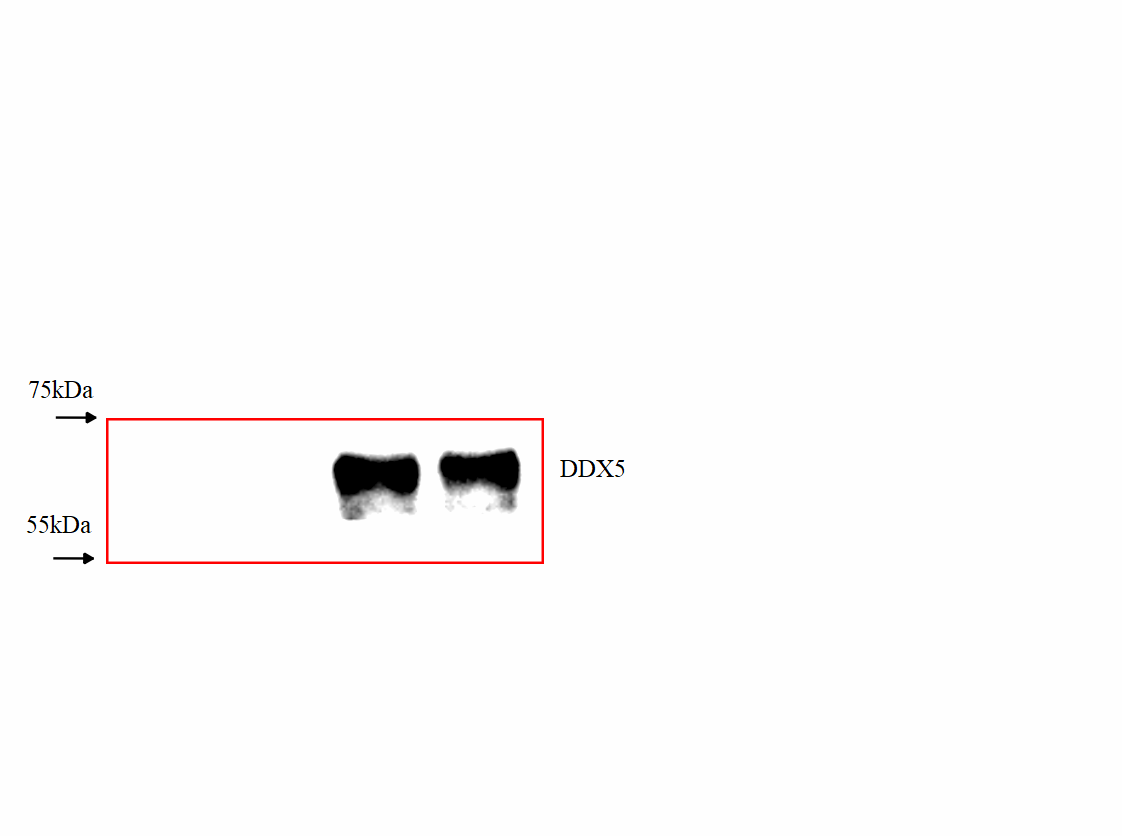

Supplement: Supplementary file 5 — Source Data Fig. 4 [file 44319_2023_47_MOESM5_ESM.zip › EMBOR-2023-57416V3-Figure_4_Source_Data-sd/Figure 4/G/IP-DDX5.tif]

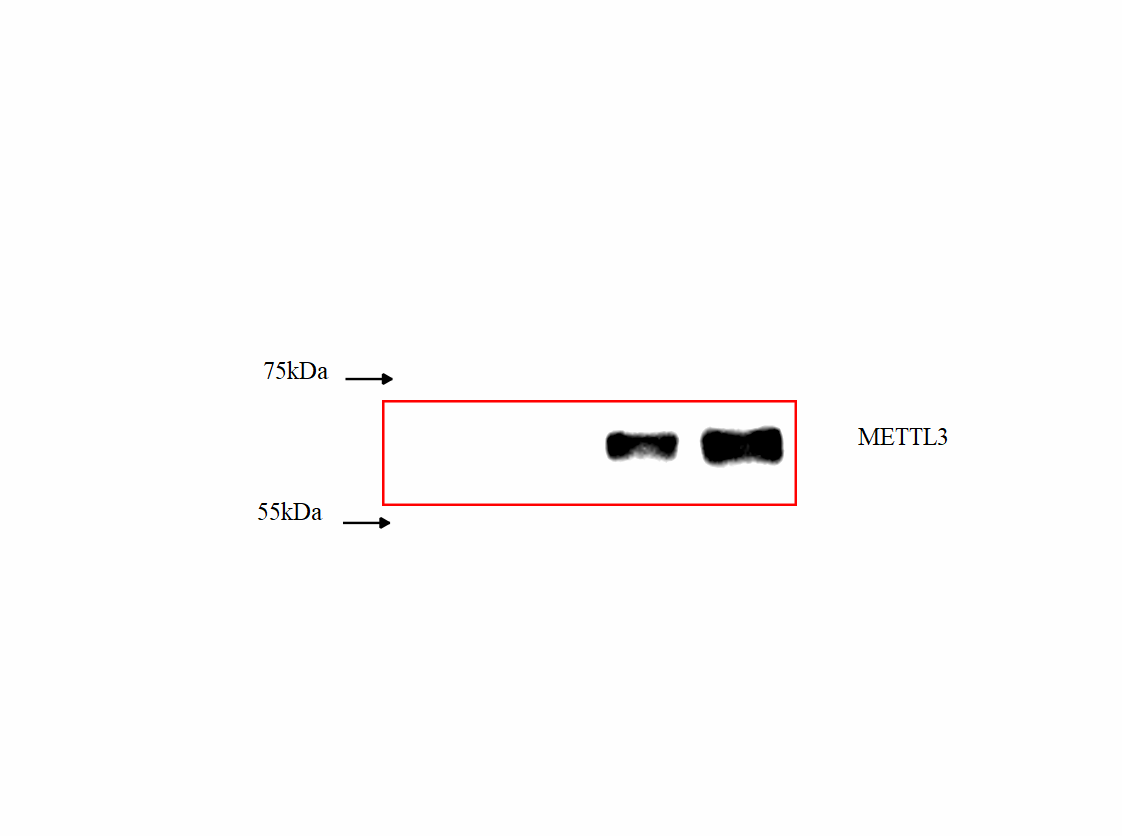

Supplement: Supplementary file 5 — Source Data Fig. 4 [file 44319_2023_47_MOESM5_ESM.zip › EMBOR-2023-57416V3-Figure_4_Source_Data-sd/Figure 4/G/IP-METTL3.tif]

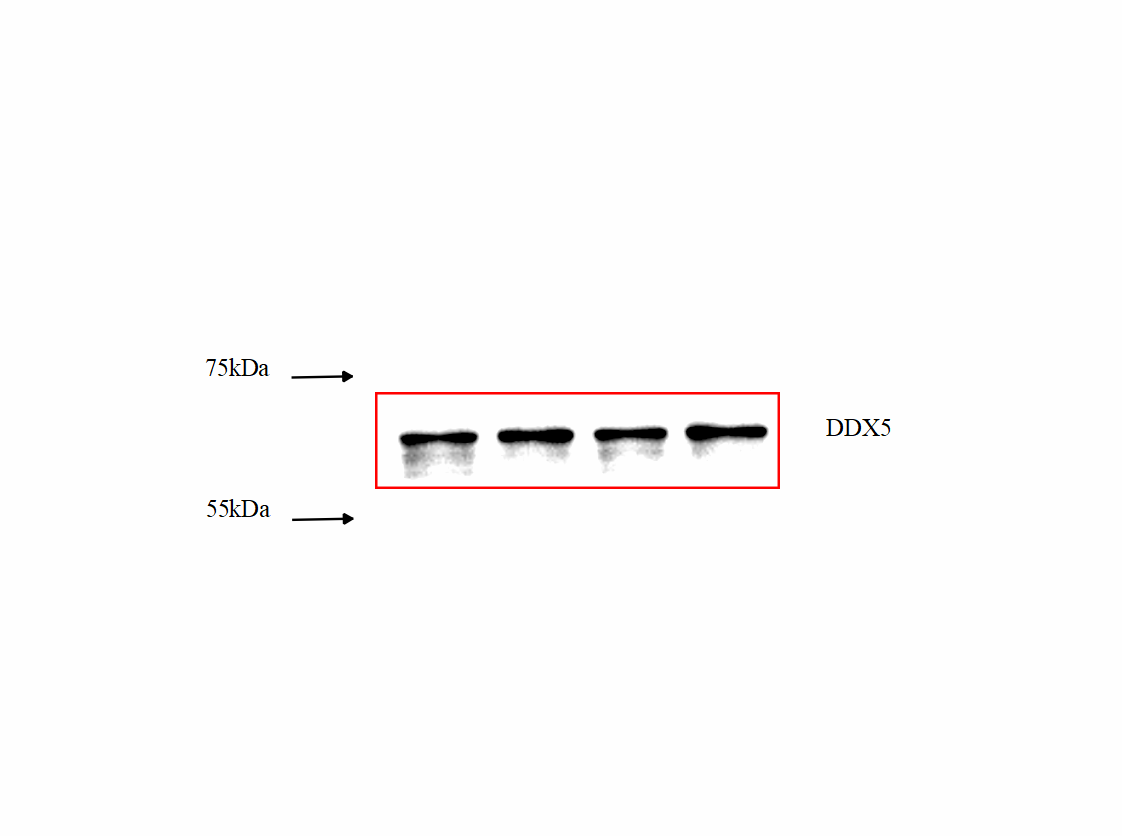

Supplement: Supplementary file 5 — Source Data Fig. 4 [file 44319_2023_47_MOESM5_ESM.zip › EMBOR-2023-57416V3-Figure_4_Source_Data-sd/Figure 4/G/WCL-DDX5.tif]

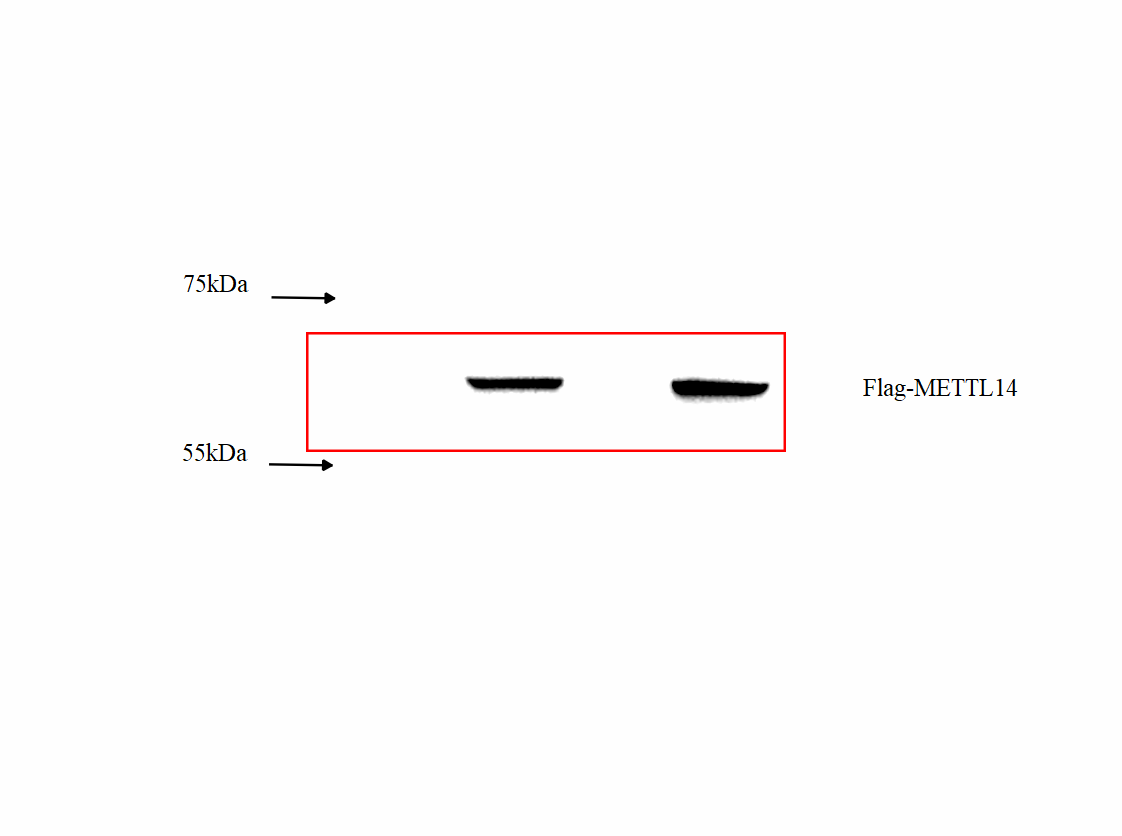

Supplement: Supplementary file 5 — Source Data Fig. 4 [file 44319_2023_47_MOESM5_ESM.zip › EMBOR-2023-57416V3-Figure_4_Source_Data-sd/Figure 4/G/WCL-Flag METTL14.tif]

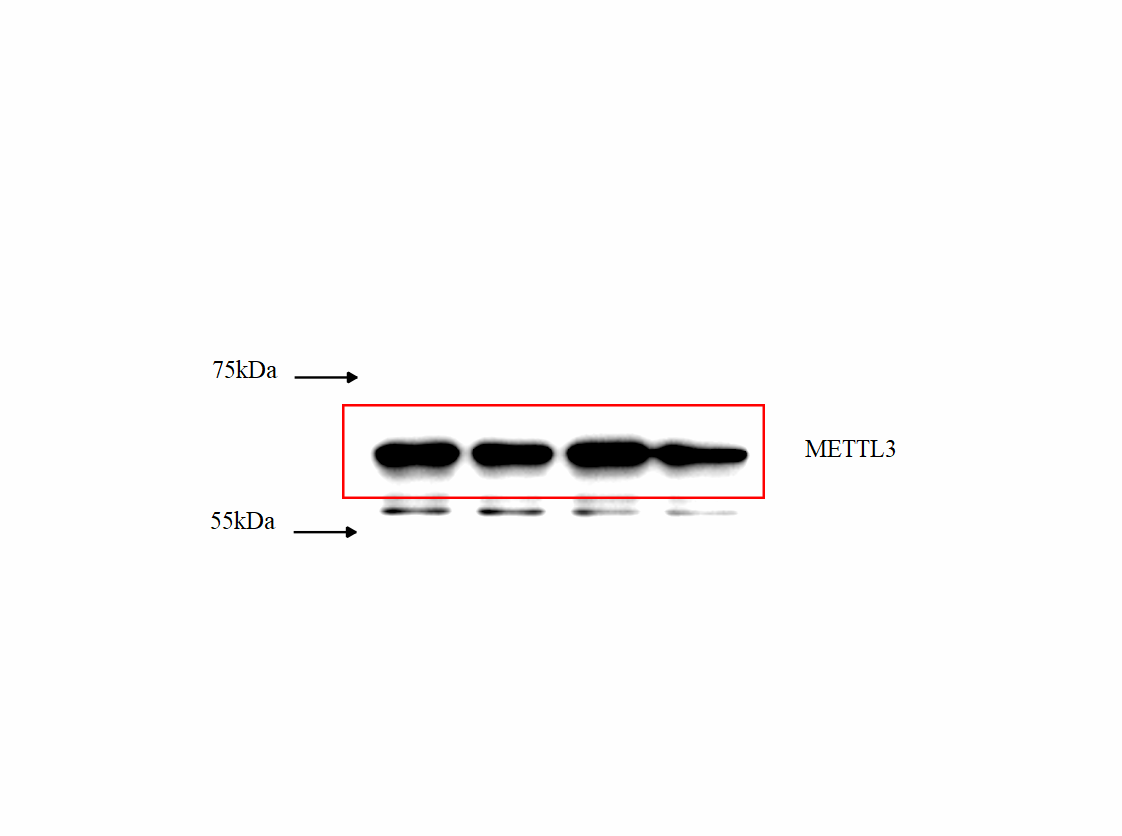

Supplement: Supplementary file 5 — Source Data Fig. 4 [file 44319_2023_47_MOESM5_ESM.zip › EMBOR-2023-57416V3-Figure_4_Source_Data-sd/Figure 4/G/WCL-METTL3.tif]

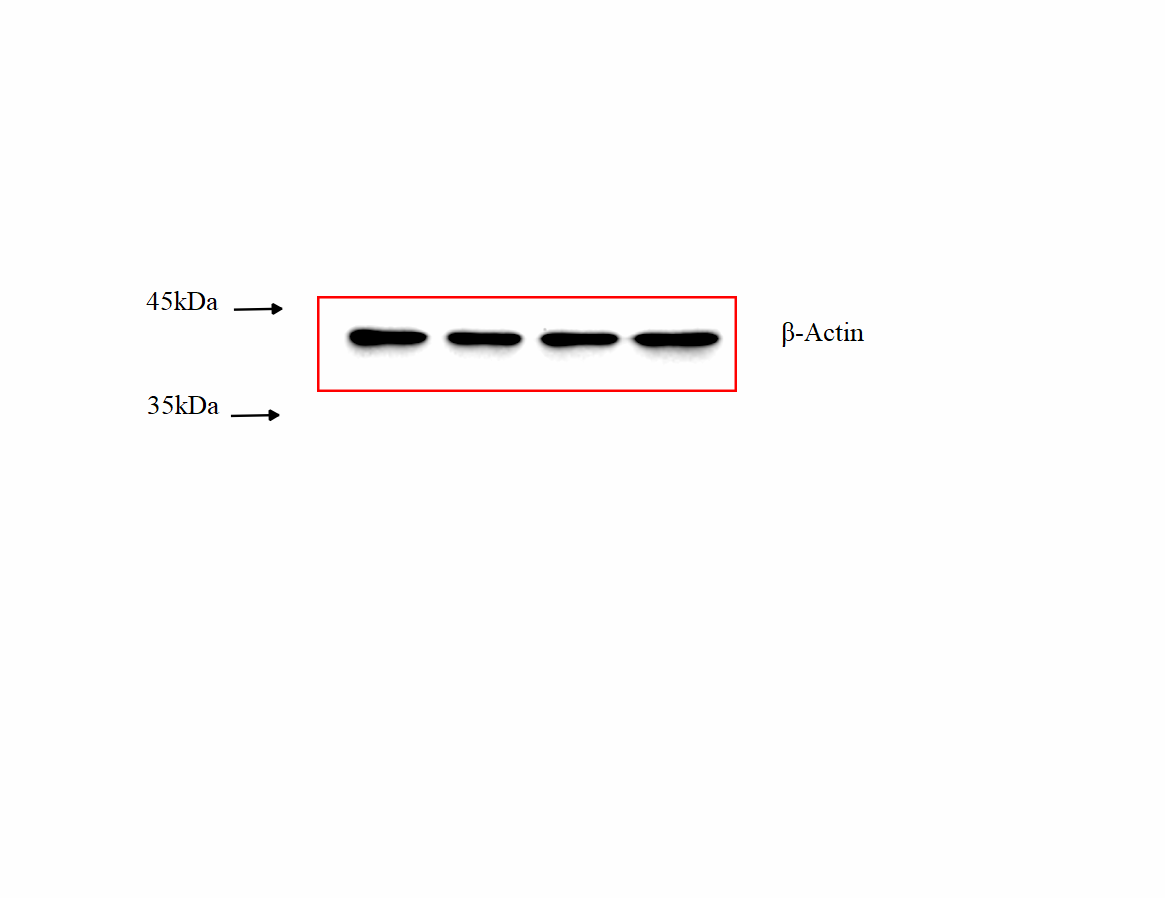

Supplement: Supplementary file 5 — Source Data Fig. 4 [file 44319_2023_47_MOESM5_ESM.zip › EMBOR-2023-57416V3-Figure_4_Source_Data-sd/Figure 4/G/WCL-β-Actin.tif]

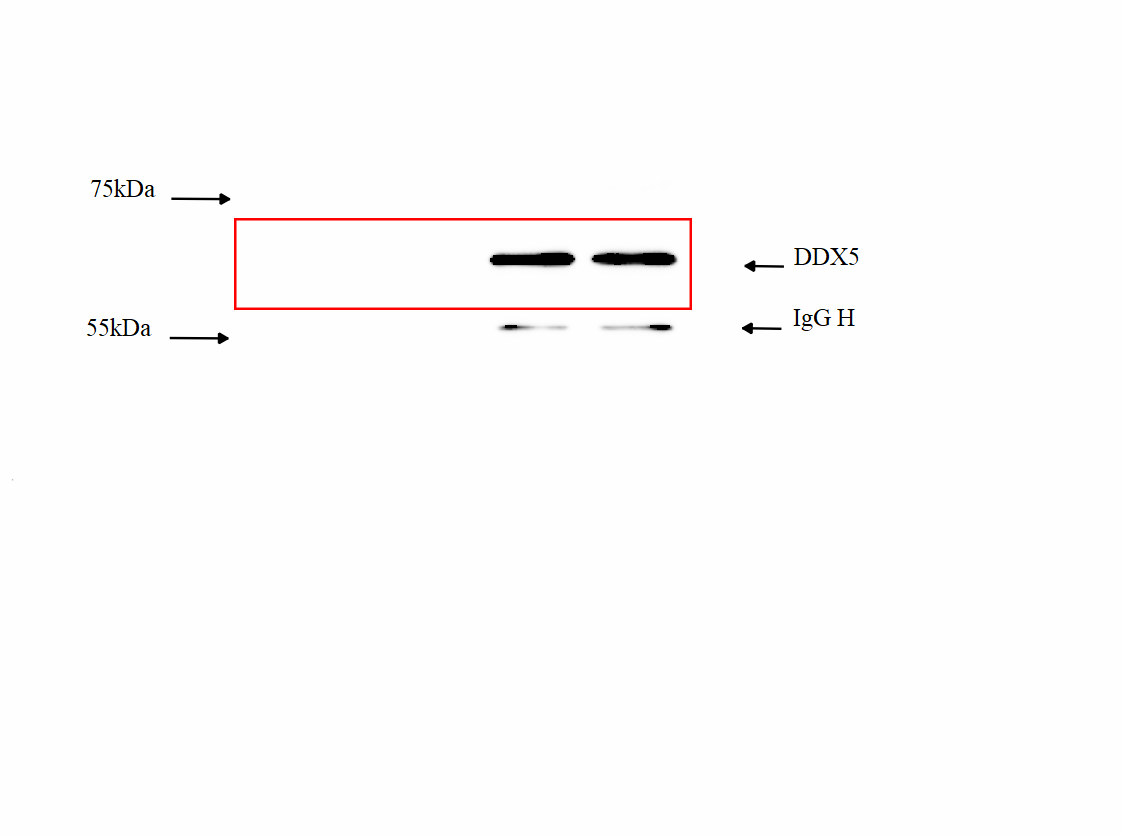

Supplement: Supplementary file 5 — Source Data Fig. 4 [file 44319_2023_47_MOESM5_ESM.zip › EMBOR-2023-57416V3-Figure_4_Source_Data-sd/Figure 4/H/IP-DDX5.tif]

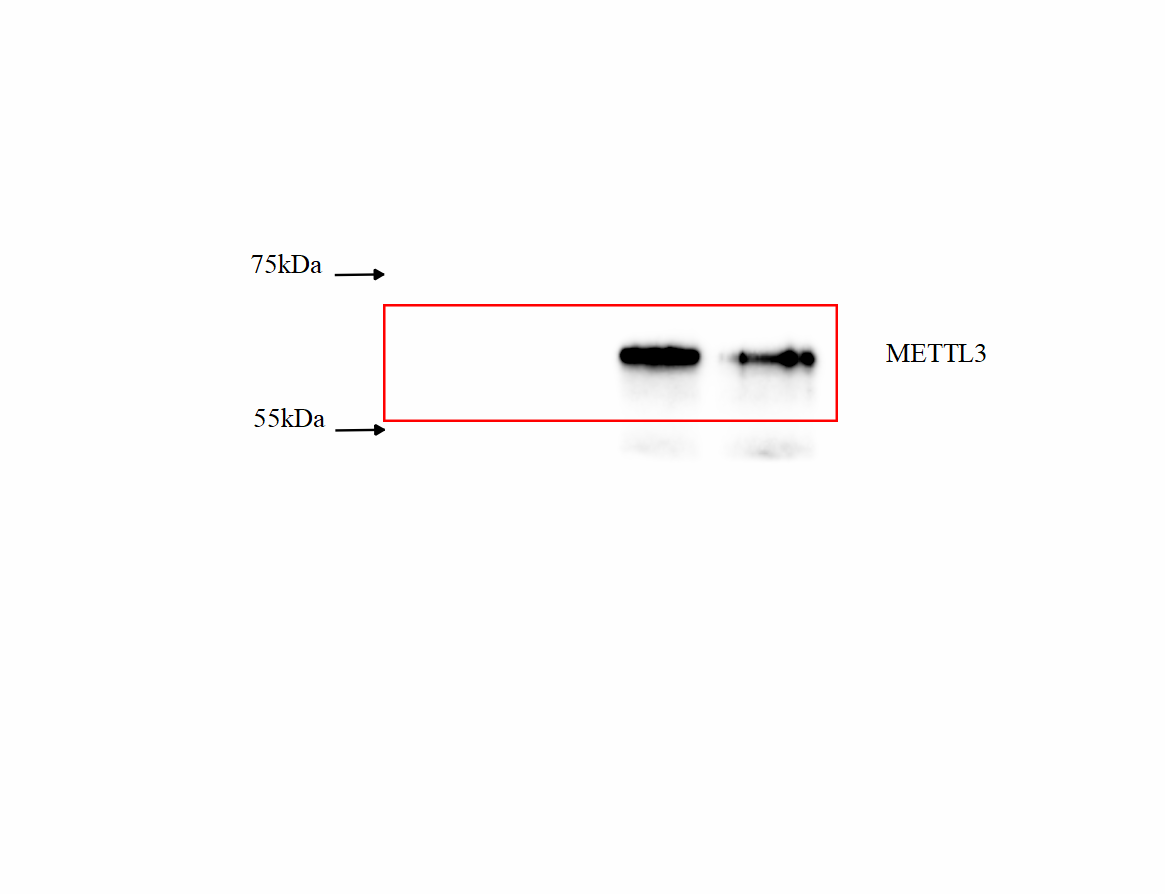

Supplement: Supplementary file 5 — Source Data Fig. 4 [file 44319_2023_47_MOESM5_ESM.zip › EMBOR-2023-57416V3-Figure_4_Source_Data-sd/Figure 4/H/IP-METTL3.tif]

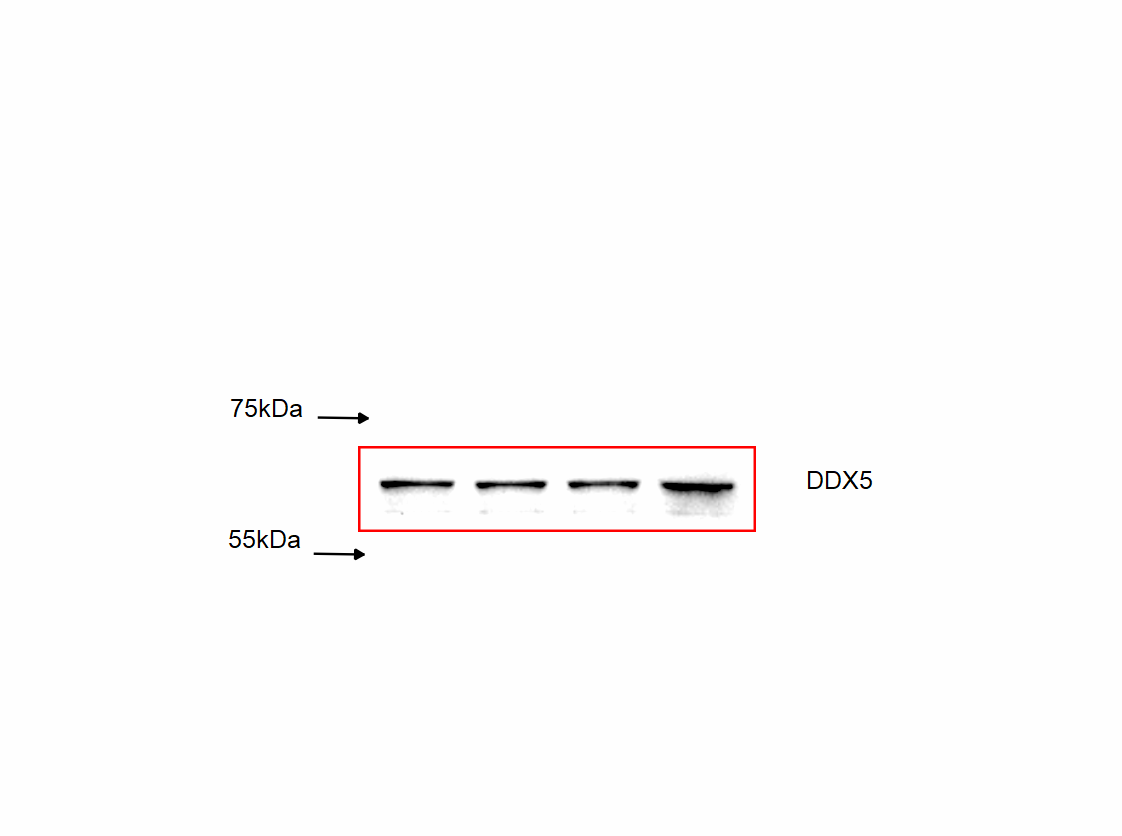

Supplement: Supplementary file 5 — Source Data Fig. 4 [file 44319_2023_47_MOESM5_ESM.zip › EMBOR-2023-57416V3-Figure_4_Source_Data-sd/Figure 4/H/WCL-DDX5.tif]

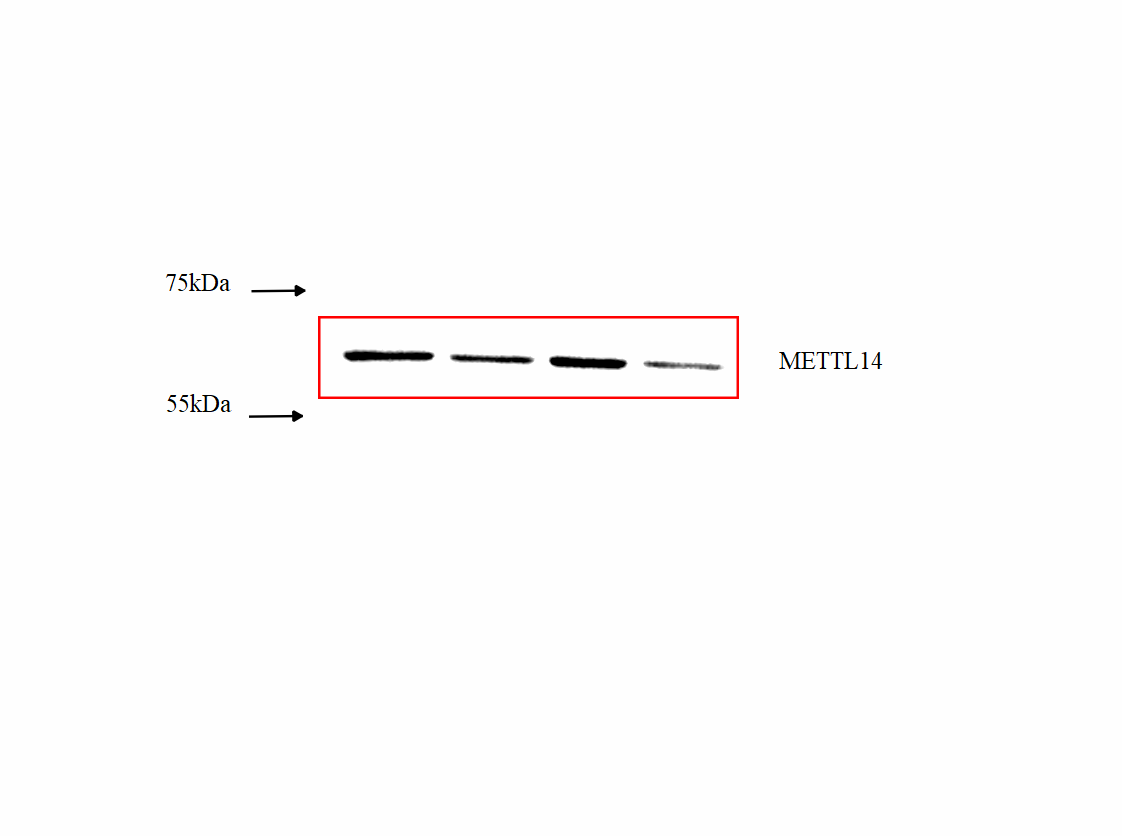

Supplement: Supplementary file 5 — Source Data Fig. 4 [file 44319_2023_47_MOESM5_ESM.zip › EMBOR-2023-57416V3-Figure_4_Source_Data-sd/Figure 4/H/WCL-METTL14.tif]

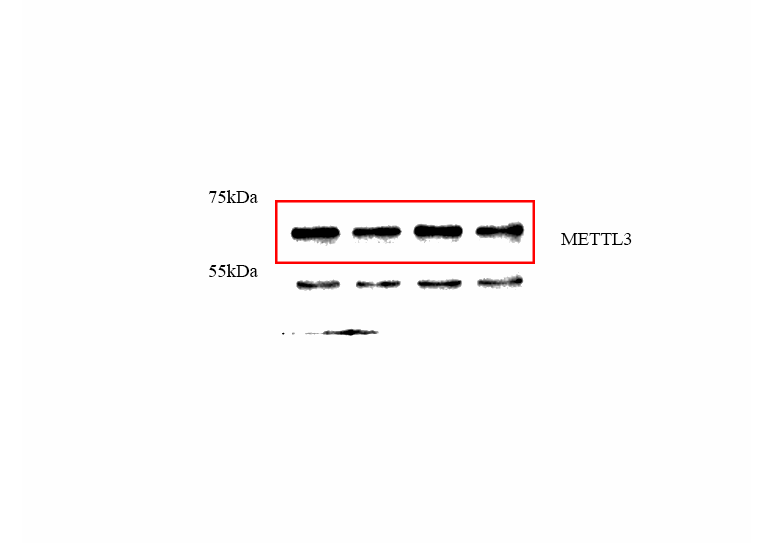

Supplement: Supplementary file 5 — Source Data Fig. 4 [file 44319_2023_47_MOESM5_ESM.zip › EMBOR-2023-57416V3-Figure_4_Source_Data-sd/Figure 4/H/WCL-METTL3.tif]

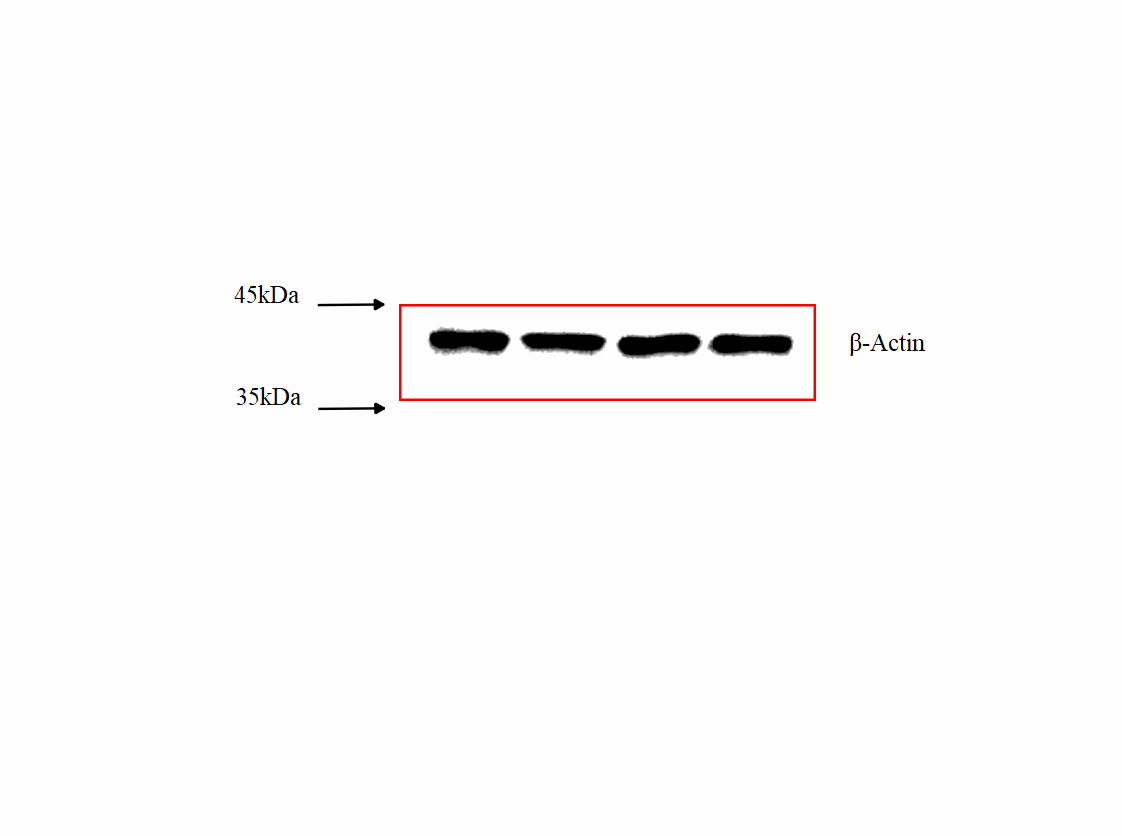

Supplement: Supplementary file 5 — Source Data Fig. 4 [file 44319_2023_47_MOESM5_ESM.zip › EMBOR-2023-57416V3-Figure_4_Source_Data-sd/Figure 4/H/WCL-β-Actin.tif]

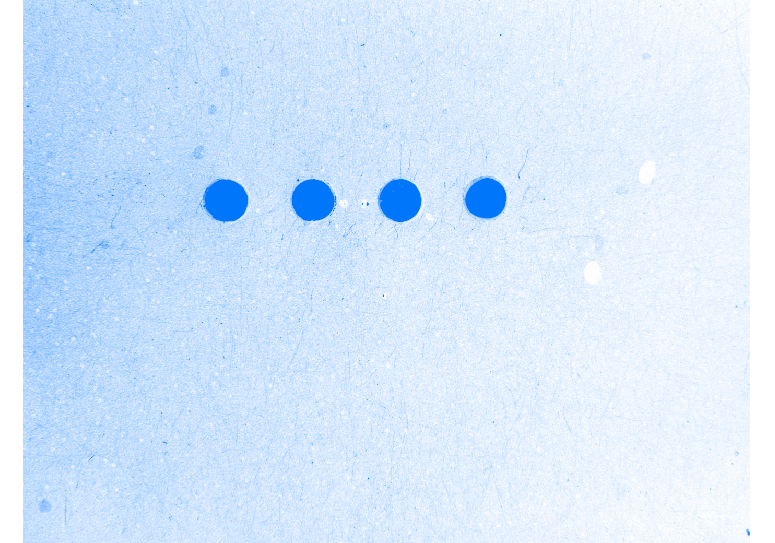

Supplement: Supplementary file 6 — Source Data Fig. 5 [file 44319_2023_47_MOESM6_ESM.zip › EMBOR-2023-57416V3-Figure_5_Source_Data-sd/Figure 5/B/FSL-1 MB-DDX5.tif]

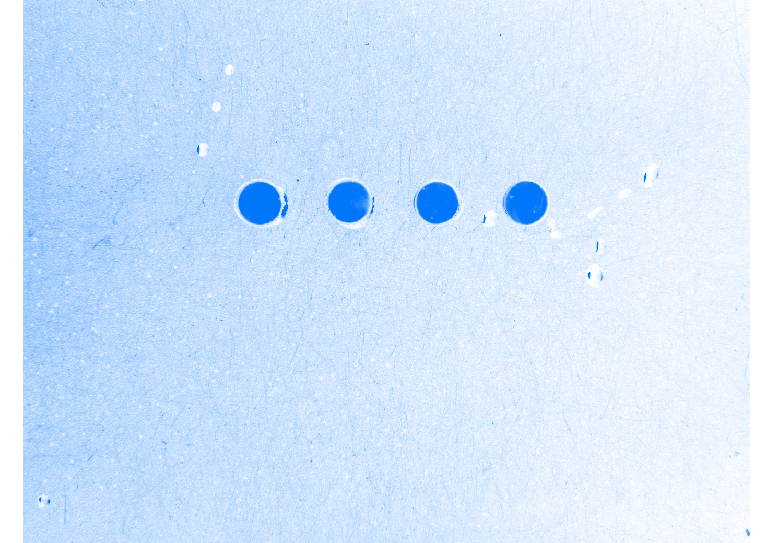

Supplement: Supplementary file 6 — Source Data Fig. 5 [file 44319_2023_47_MOESM6_ESM.zip › EMBOR-2023-57416V3-Figure_5_Source_Data-sd/Figure 5/B/FSL-1 MB-siDDX5.tif]

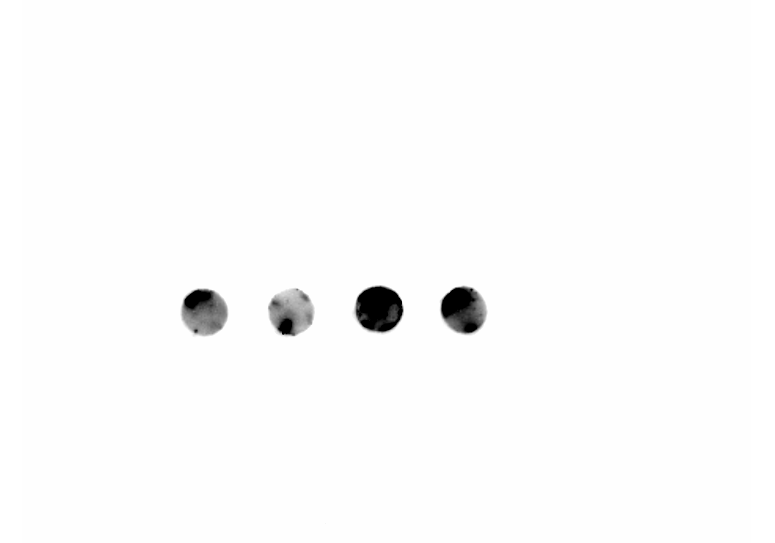

Supplement: Supplementary file 6 — Source Data Fig. 5 [file 44319_2023_47_MOESM6_ESM.zip › EMBOR-2023-57416V3-Figure_5_Source_Data-sd/Figure 5/B/FSL-1 dot blot-DDX5.tif]

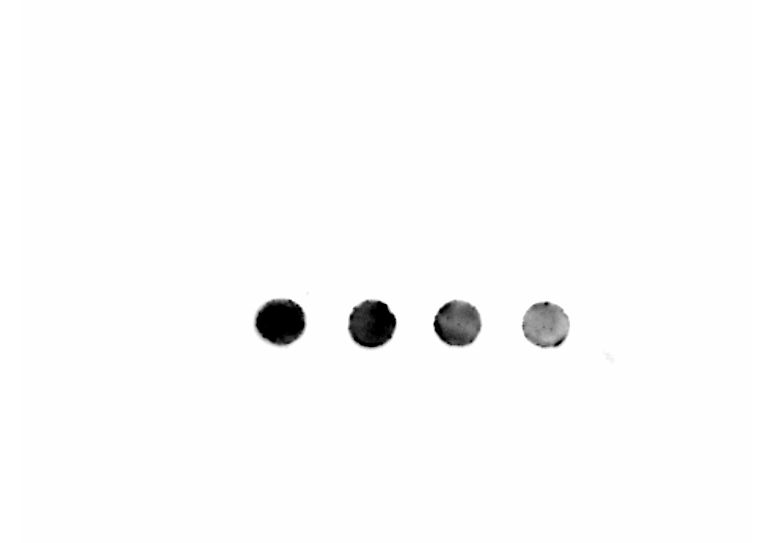

Supplement: Supplementary file 6 — Source Data Fig. 5 [file 44319_2023_47_MOESM6_ESM.zip › EMBOR-2023-57416V3-Figure_5_Source_Data-sd/Figure 5/B/FSL-1 dot blot-siDDX5.tif]

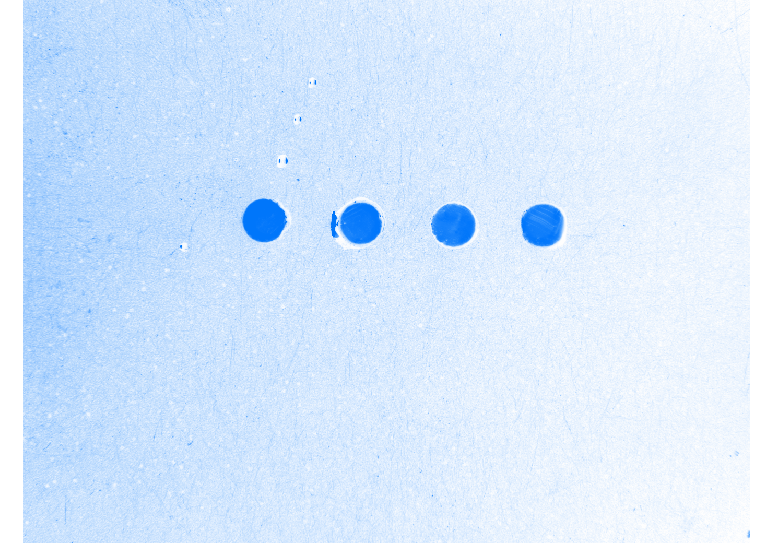

Supplement: Supplementary file 6 — Source Data Fig. 5 [file 44319_2023_47_MOESM6_ESM.zip › EMBOR-2023-57416V3-Figure_5_Source_Data-sd/Figure 5/B/LPS MB-DDX5.tif]

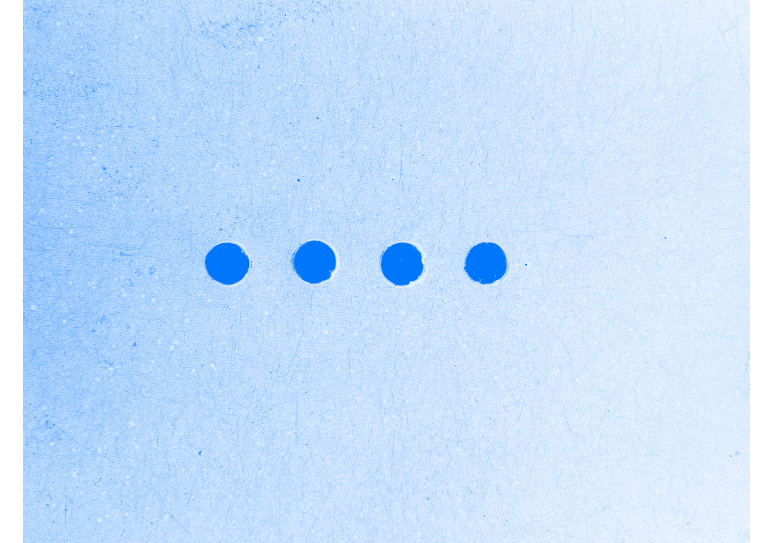

Supplement: Supplementary file 6 — Source Data Fig. 5 [file 44319_2023_47_MOESM6_ESM.zip › EMBOR-2023-57416V3-Figure_5_Source_Data-sd/Figure 5/B/LPS MB-siDDX5.tif]

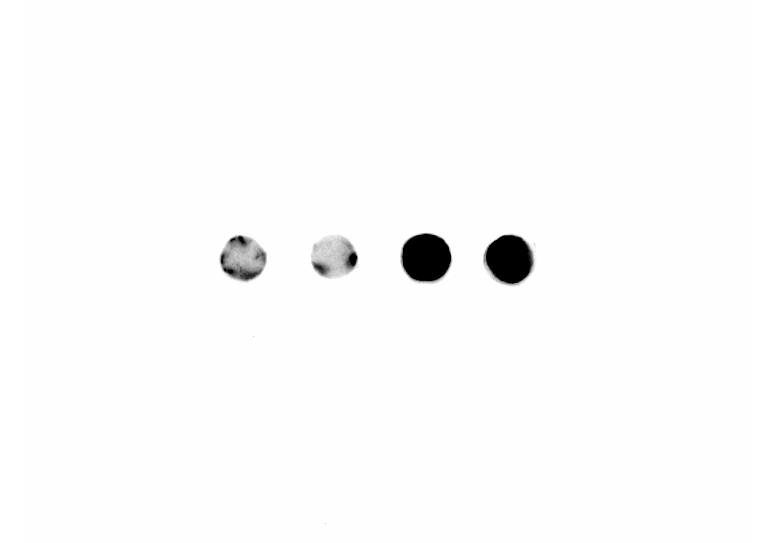

Supplement: Supplementary file 6 — Source Data Fig. 5 [file 44319_2023_47_MOESM6_ESM.zip › EMBOR-2023-57416V3-Figure_5_Source_Data-sd/Figure 5/B/LPS dot blot-DDX5.tif]

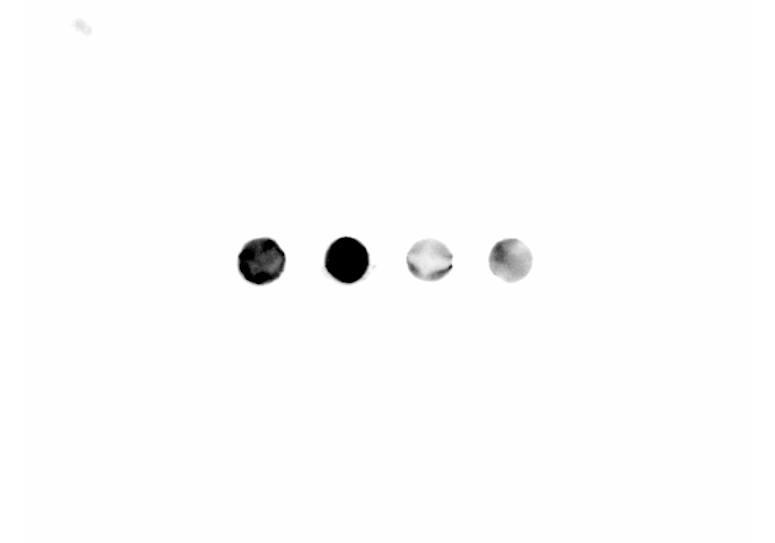

Supplement: Supplementary file 6 — Source Data Fig. 5 [file 44319_2023_47_MOESM6_ESM.zip › EMBOR-2023-57416V3-Figure_5_Source_Data-sd/Figure 5/B/LPS dot blot-siDDX5.tif]

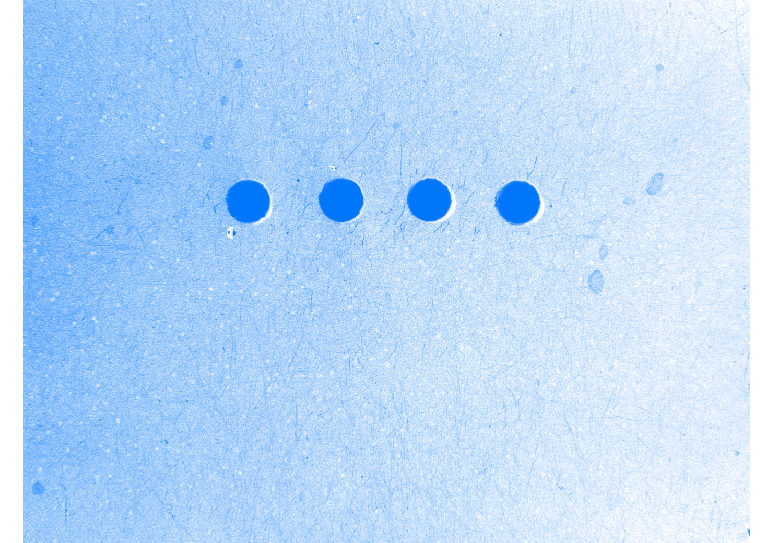

Supplement: Supplementary file 6 — Source Data Fig. 5 [file 44319_2023_47_MOESM6_ESM.zip › EMBOR-2023-57416V3-Figure_5_Source_Data-sd/Figure 5/B/Pam3CSK4 MB-DDX5.tif]

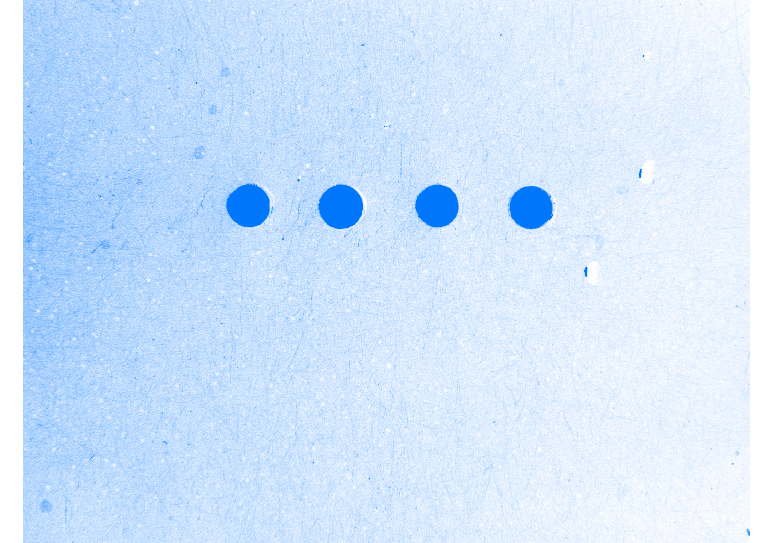

Supplement: Supplementary file 6 — Source Data Fig. 5 [file 44319_2023_47_MOESM6_ESM.zip › EMBOR-2023-57416V3-Figure_5_Source_Data-sd/Figure 5/B/Pam3CSK4 MB-siDDX5.tif]

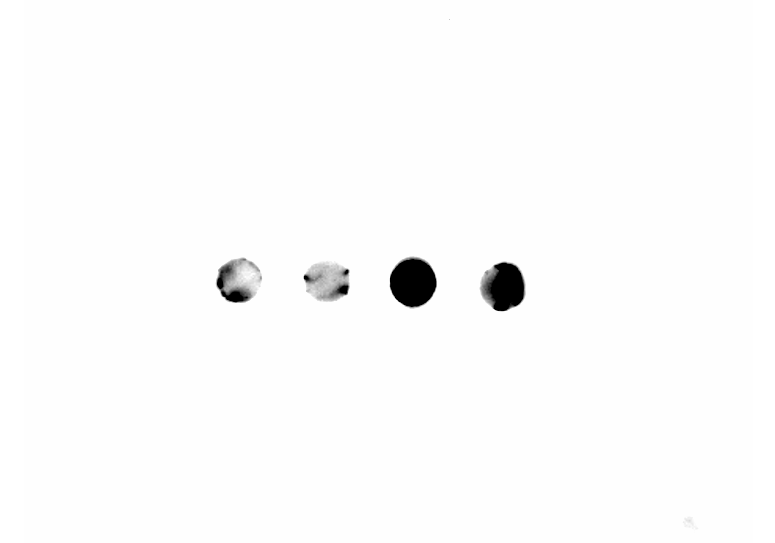

Supplement: Supplementary file 6 — Source Data Fig. 5 [file 44319_2023_47_MOESM6_ESM.zip › EMBOR-2023-57416V3-Figure_5_Source_Data-sd/Figure 5/B/Pam3CSK4 dot blot-DDX5.tif]

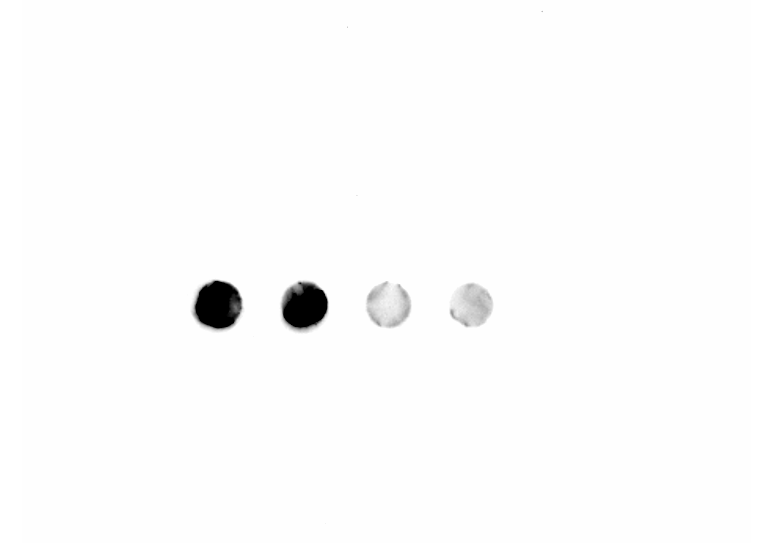

Supplement: Supplementary file 6 — Source Data Fig. 5 [file 44319_2023_47_MOESM6_ESM.zip › EMBOR-2023-57416V3-Figure_5_Source_Data-sd/Figure 5/B/Pam3CSK4 dot blot-siDDX5.tif]

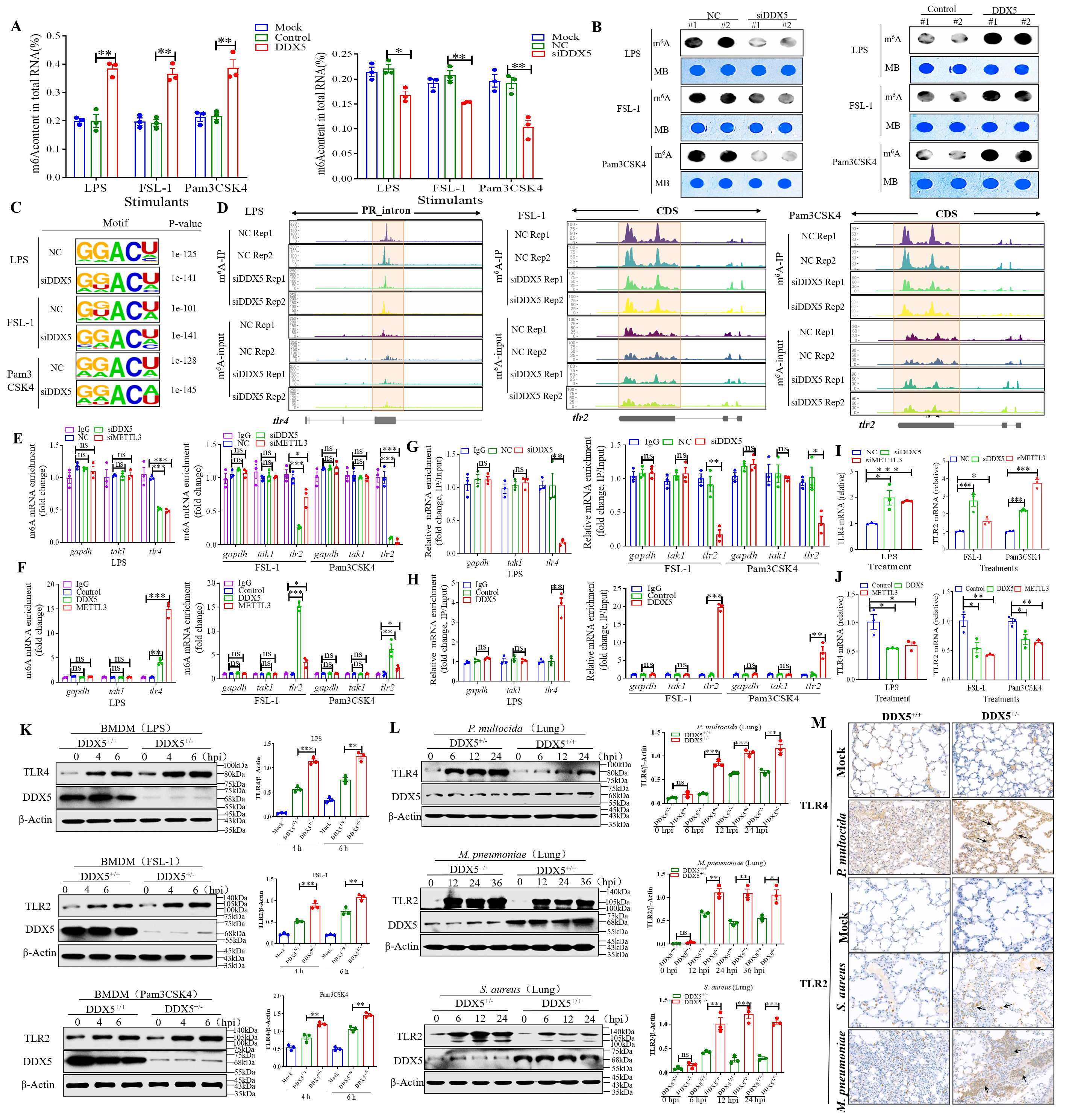

Supplement: Supplementary file 6 — Source Data Fig. 5 [file 44319_2023_47_MOESM6_ESM.zip › EMBOR-2023-57416V3-Figure_5_Source_Data-sd/Figure 5/C/m6A motif-FSL-1-NC.tif]

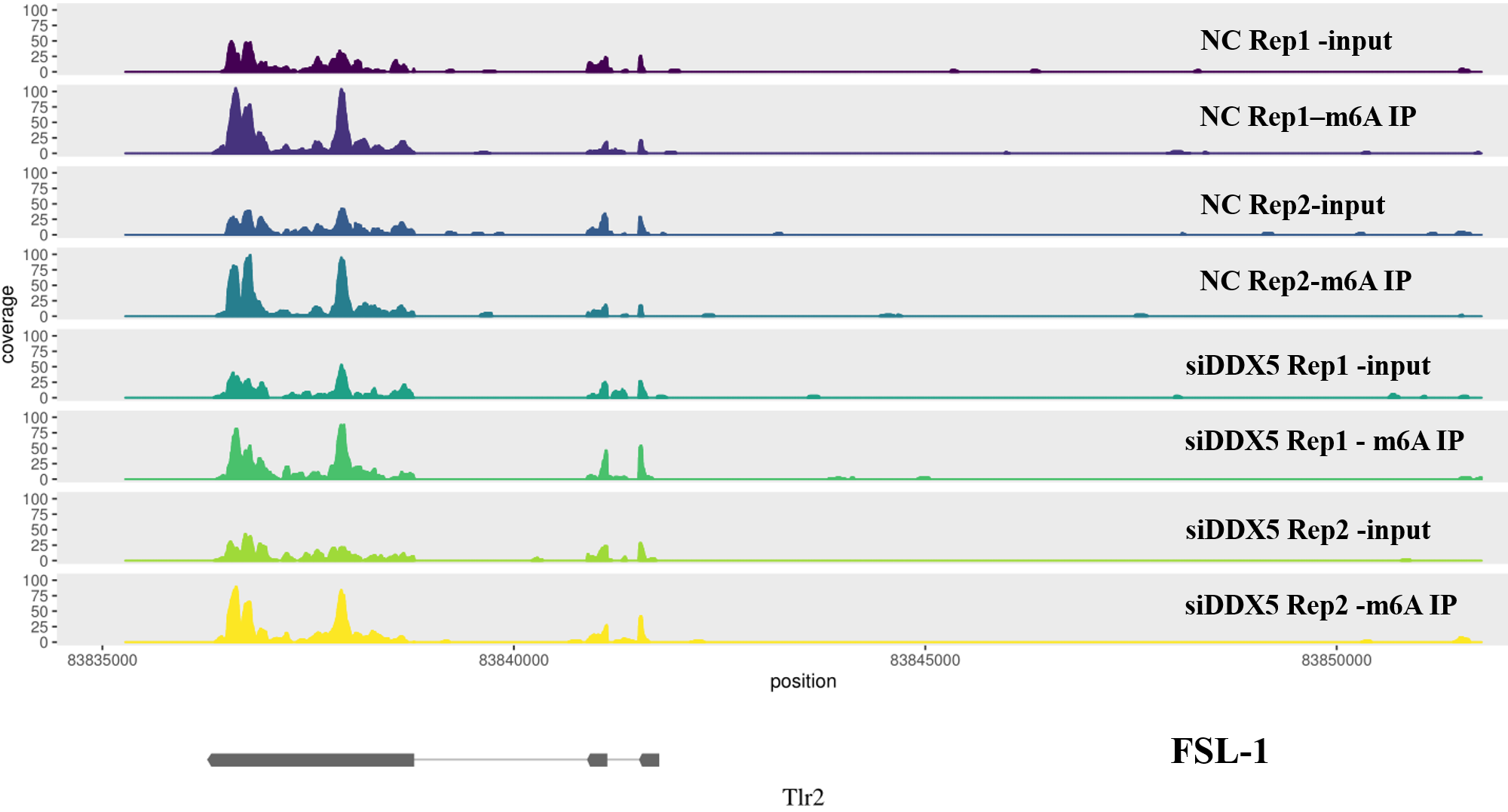

Supplement: Supplementary file 6 — Source Data Fig. 5 [file 44319_2023_47_MOESM6_ESM.zip › EMBOR-2023-57416V3-Figure_5_Source_Data-sd/Figure 5/D/IGV-FSL-1.tif]

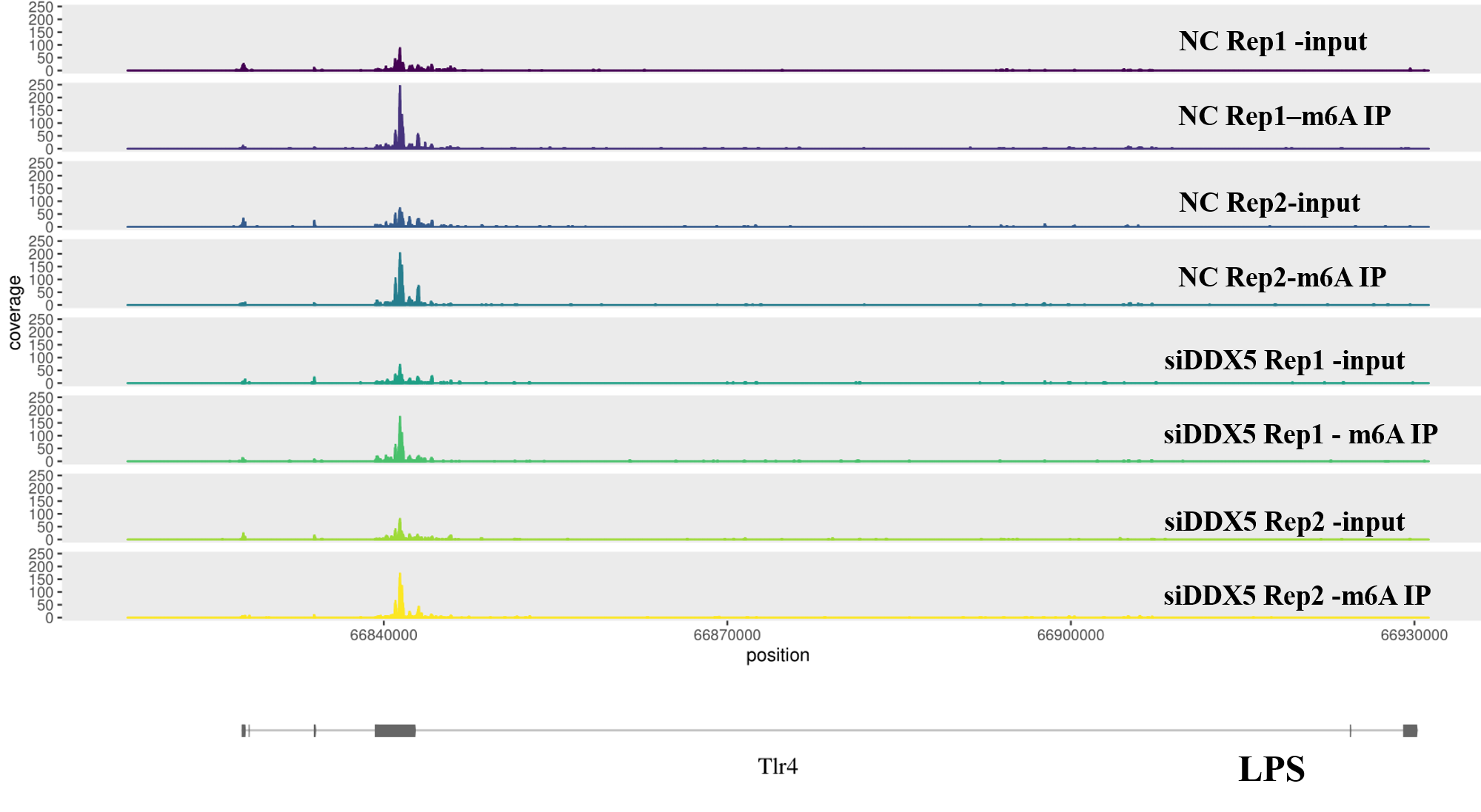

Supplement: Supplementary file 6 — Source Data Fig. 5 [file 44319_2023_47_MOESM6_ESM.zip › EMBOR-2023-57416V3-Figure_5_Source_Data-sd/Figure 5/D/IGV-LPS.tif]

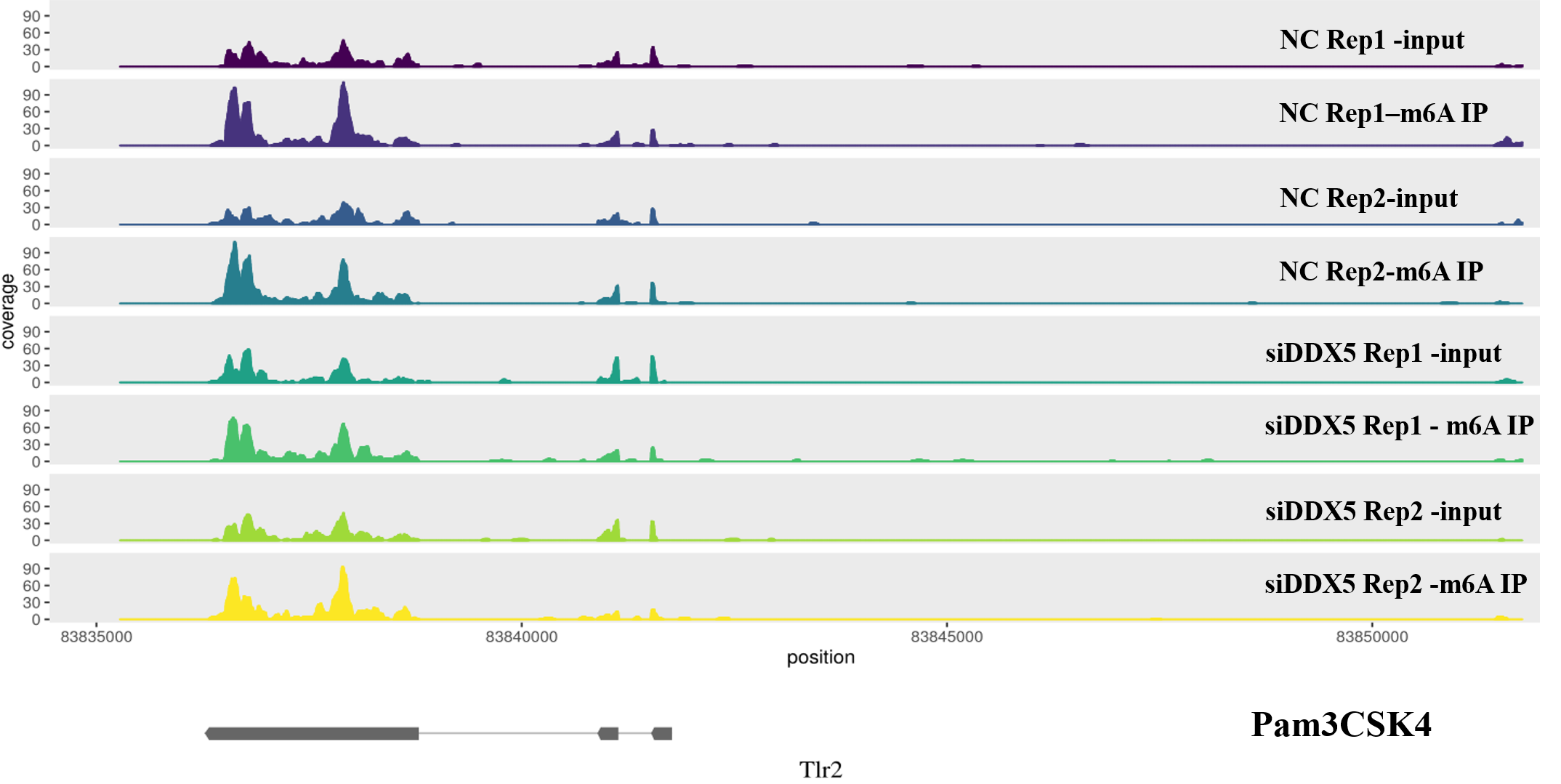

Supplement: Supplementary file 6 — Source Data Fig. 5 [file 44319_2023_47_MOESM6_ESM.zip › EMBOR-2023-57416V3-Figure_5_Source_Data-sd/Figure 5/D/IGV-Pam3CSK4.tif]

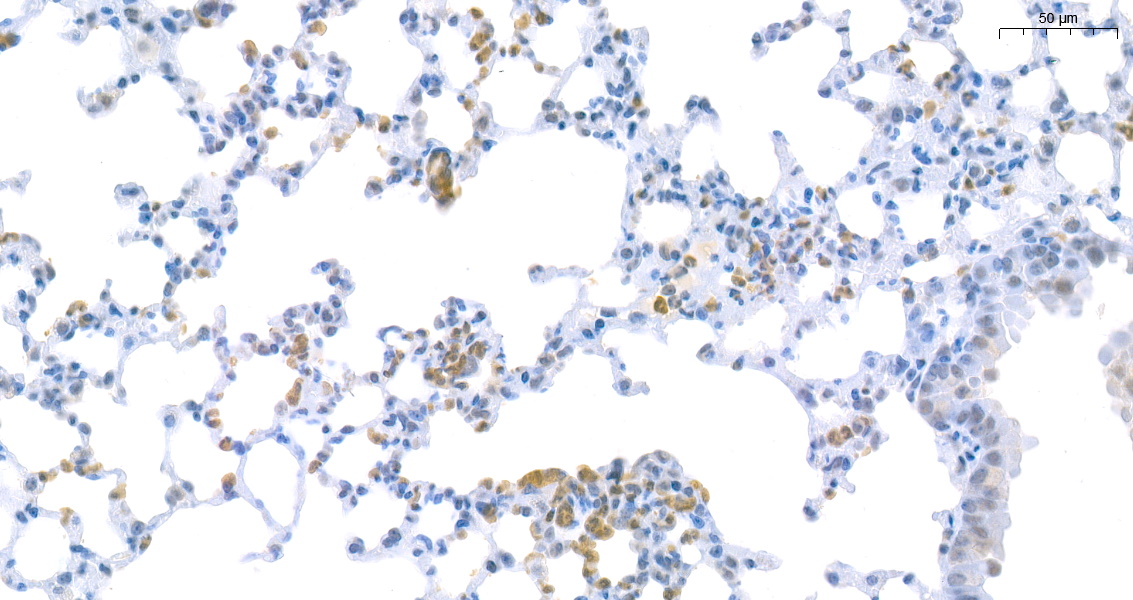

Supplement: Supplementary file 8 — Source Data Fig. 7 [file 44319_2023_47_MOESM8_ESM.zip › EMBOR-2023-57416V3-Figure_7_Source_Data-sd/Figure 7/A/TLR2 METTL3 WT-M.pneumoniae.jpg]

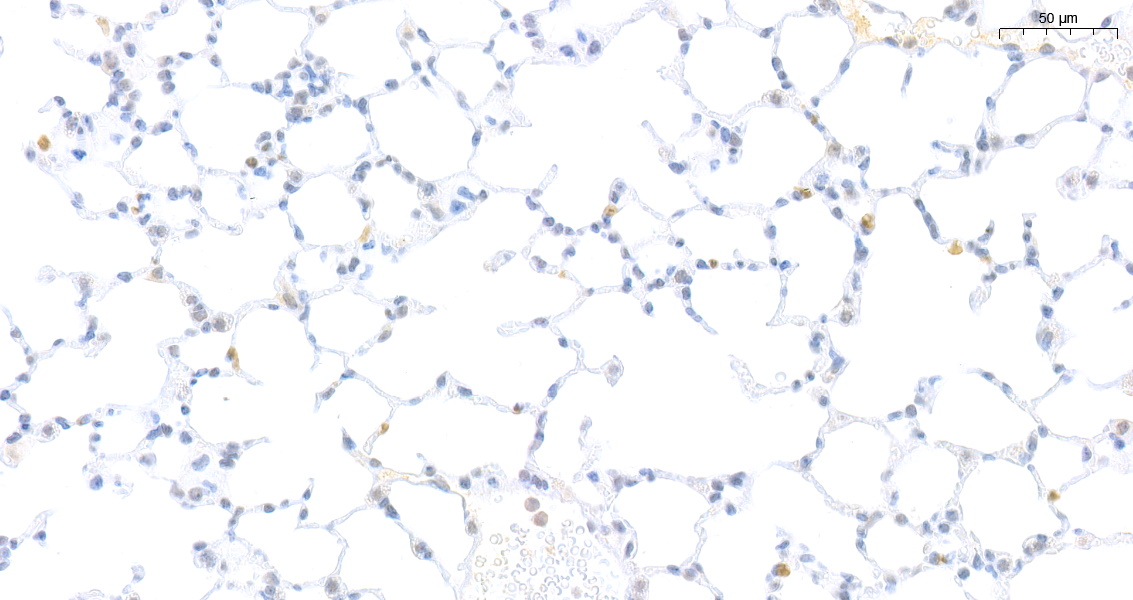

Supplement: Supplementary file 8 — Source Data Fig. 7 [file 44319_2023_47_MOESM8_ESM.zip › EMBOR-2023-57416V3-Figure_7_Source_Data-sd/Figure 7/A/TLR2 METTL3 WT-Mock.jpg]

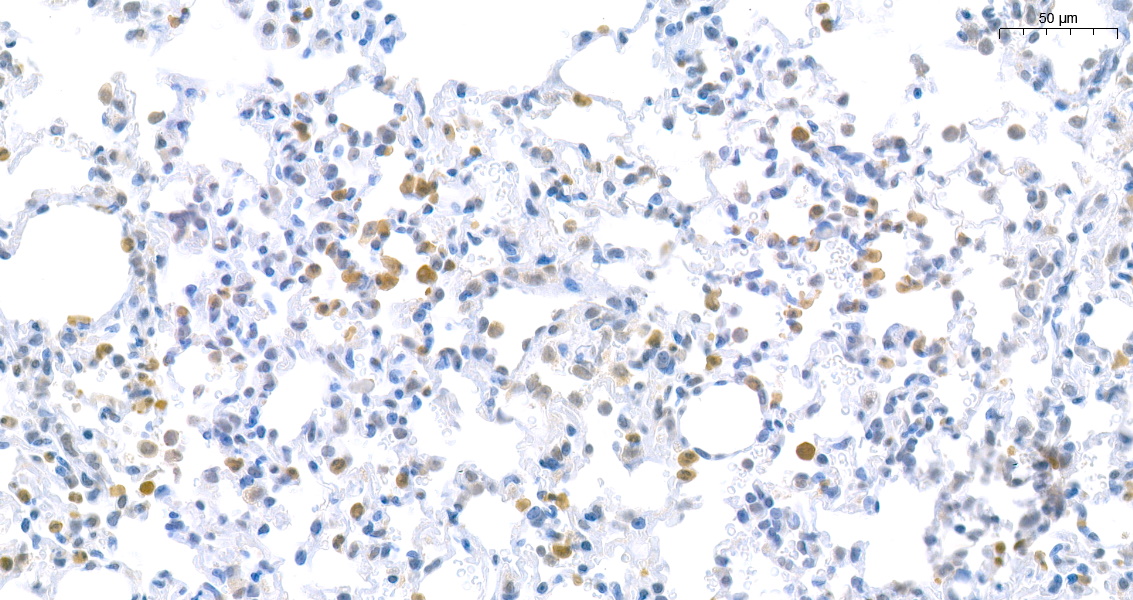

Supplement: Supplementary file 8 — Source Data Fig. 7 [file 44319_2023_47_MOESM8_ESM.zip › EMBOR-2023-57416V3-Figure_7_Source_Data-sd/Figure 7/A/TLR2 METTL3 WT-S.aureus.jpg]

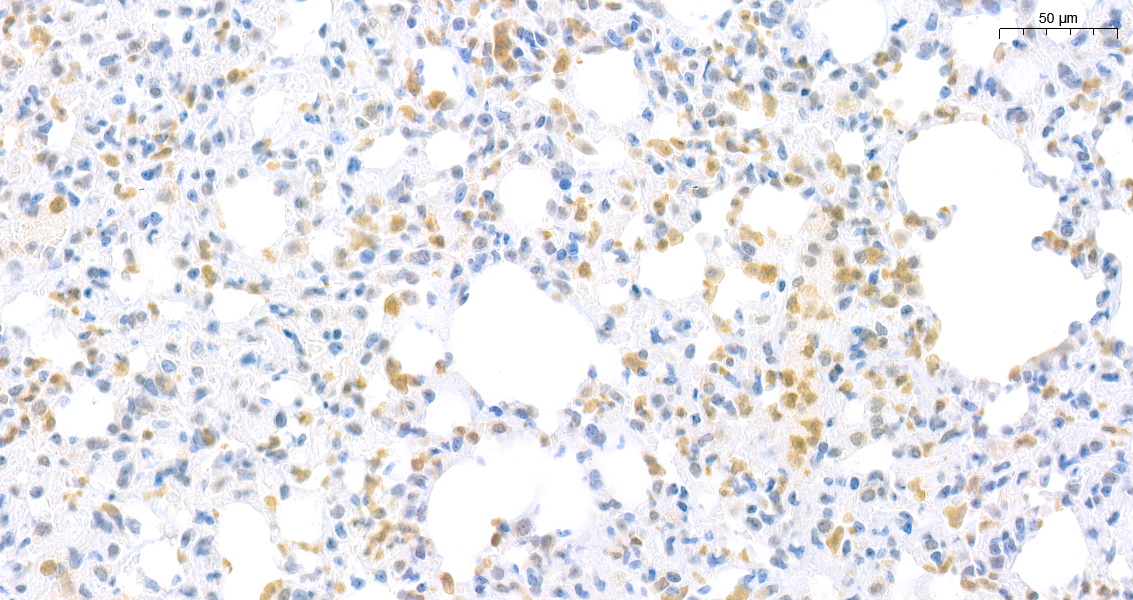

Supplement: Supplementary file 8 — Source Data Fig. 7 [file 44319_2023_47_MOESM8_ESM.zip › EMBOR-2023-57416V3-Figure_7_Source_Data-sd/Figure 7/A/TLR2 METTL3 cKO-M.pneumoniae.jpg]

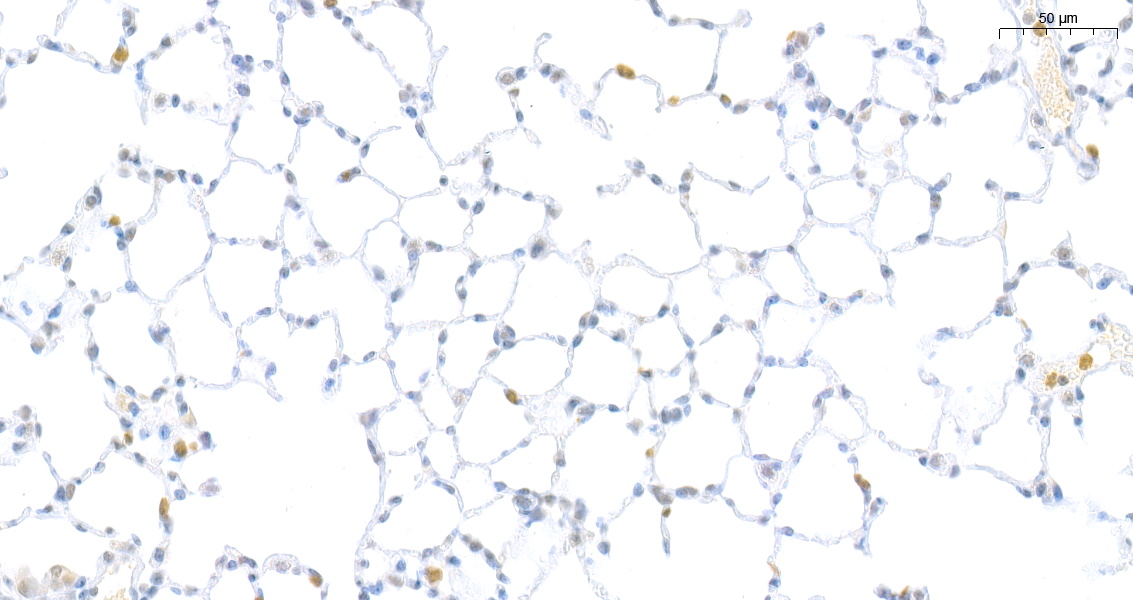

Supplement: Supplementary file 8 — Source Data Fig. 7 [file 44319_2023_47_MOESM8_ESM.zip › EMBOR-2023-57416V3-Figure_7_Source_Data-sd/Figure 7/A/TLR2 METTL3 cKO-Mock.jpg]

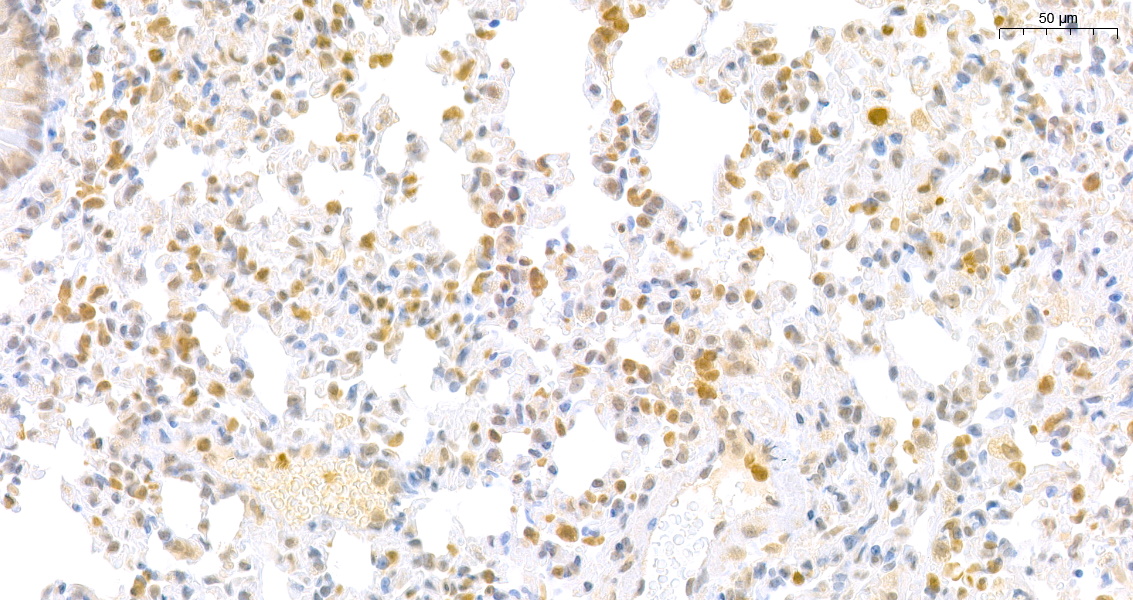

Supplement: Supplementary file 8 — Source Data Fig. 7 [file 44319_2023_47_MOESM8_ESM.zip › EMBOR-2023-57416V3-Figure_7_Source_Data-sd/Figure 7/A/TLR2 METTL3 cKO-S.aureus.jpg]

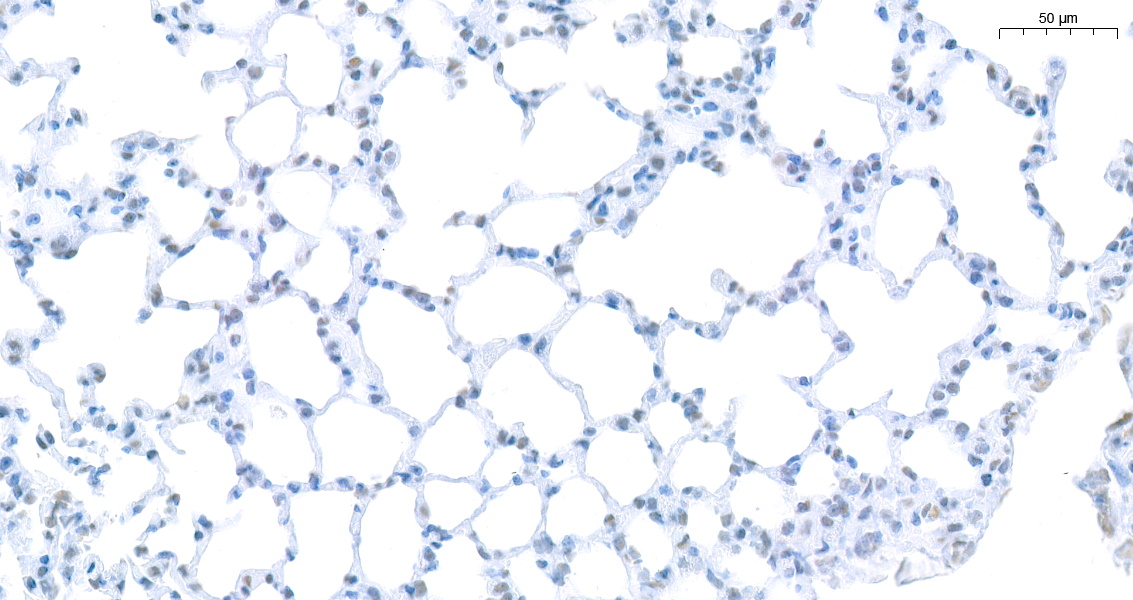

Supplement: Supplementary file 8 — Source Data Fig. 7 [file 44319_2023_47_MOESM8_ESM.zip › EMBOR-2023-57416V3-Figure_7_Source_Data-sd/Figure 7/A/TLR4 METTL3 WT-Mock.jpg]

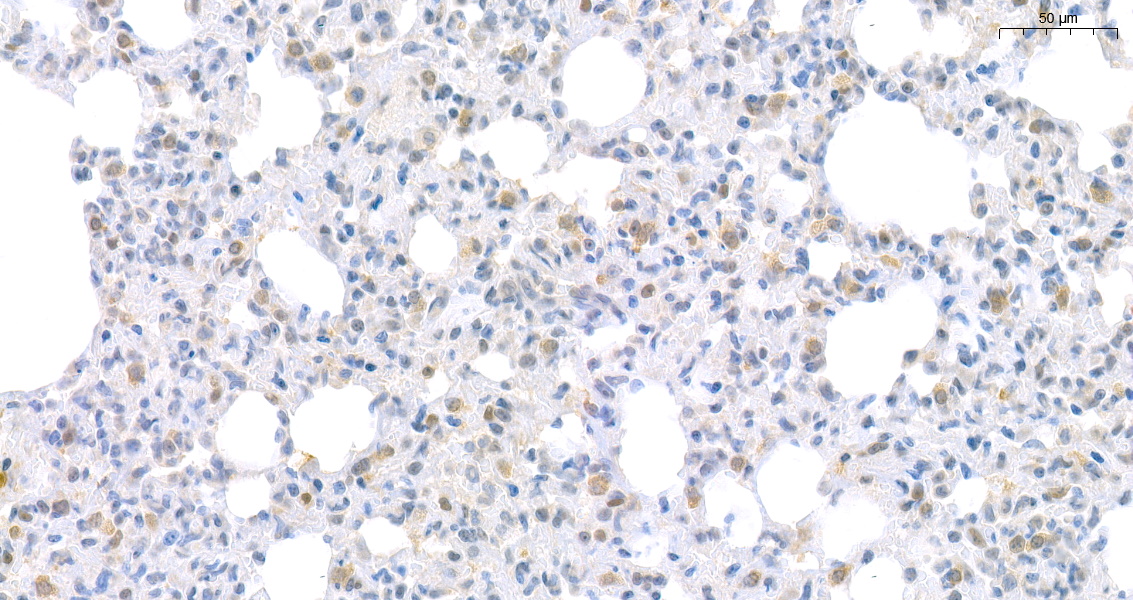

Supplement: Supplementary file 8 — Source Data Fig. 7 [file 44319_2023_47_MOESM8_ESM.zip › EMBOR-2023-57416V3-Figure_7_Source_Data-sd/Figure 7/A/TLR4 METTL3 WT-P.multocida.jpg]

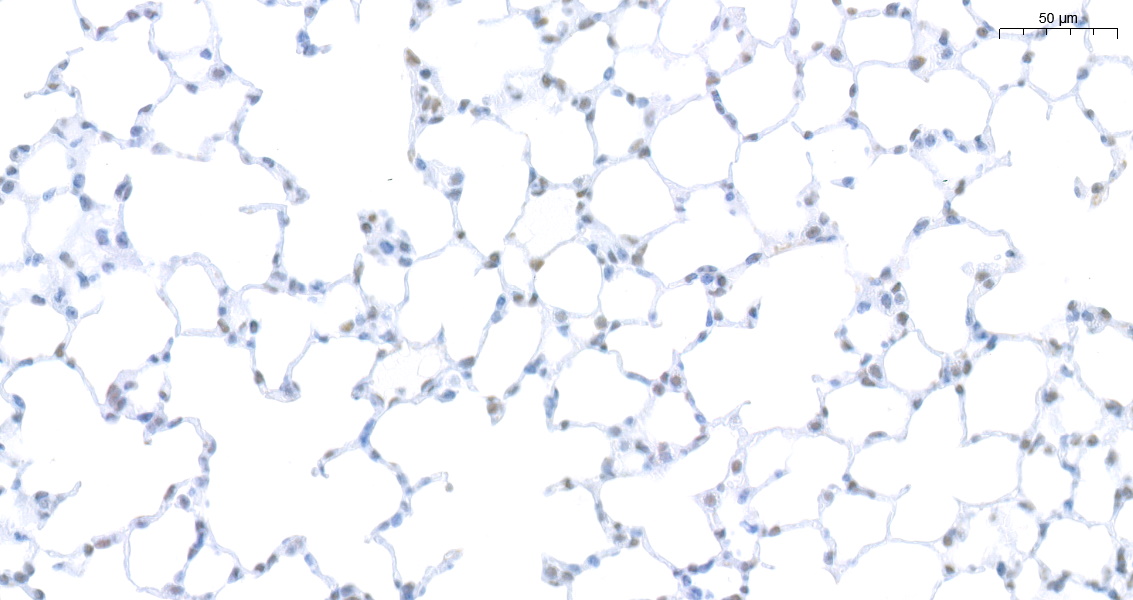

Supplement: Supplementary file 8 — Source Data Fig. 7 [file 44319_2023_47_MOESM8_ESM.zip › EMBOR-2023-57416V3-Figure_7_Source_Data-sd/Figure 7/A/TLR4 METTL3 cKO-Mock.jpg]

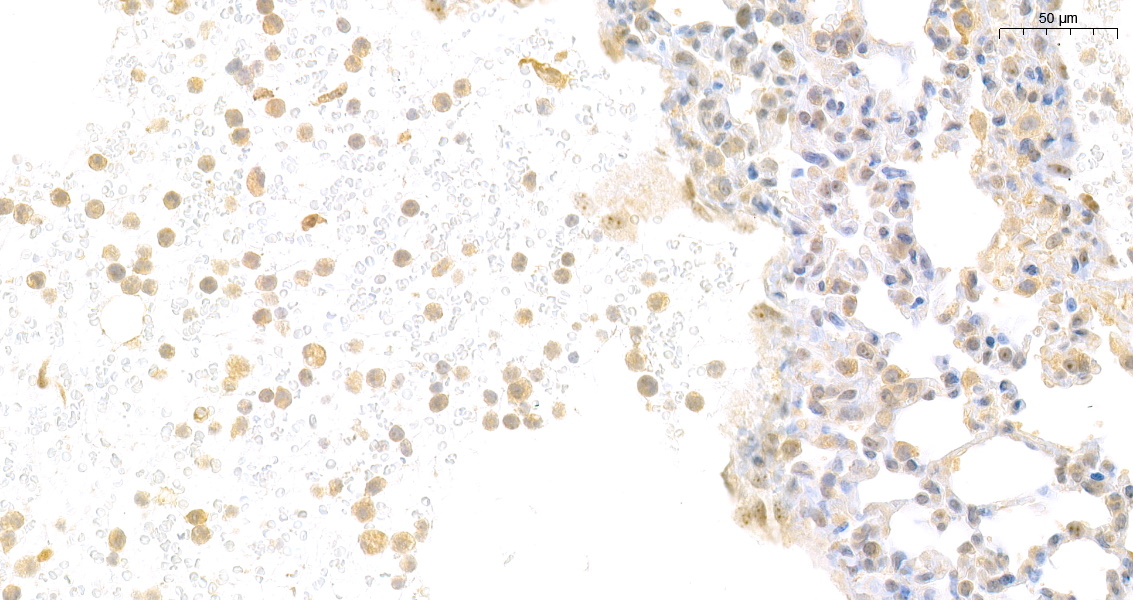

Supplement: Supplementary file 8 — Source Data Fig. 7 [file 44319_2023_47_MOESM8_ESM.zip › EMBOR-2023-57416V3-Figure_7_Source_Data-sd/Figure 7/A/TLR4 METTL3 cKO-P.multocida.jpg]

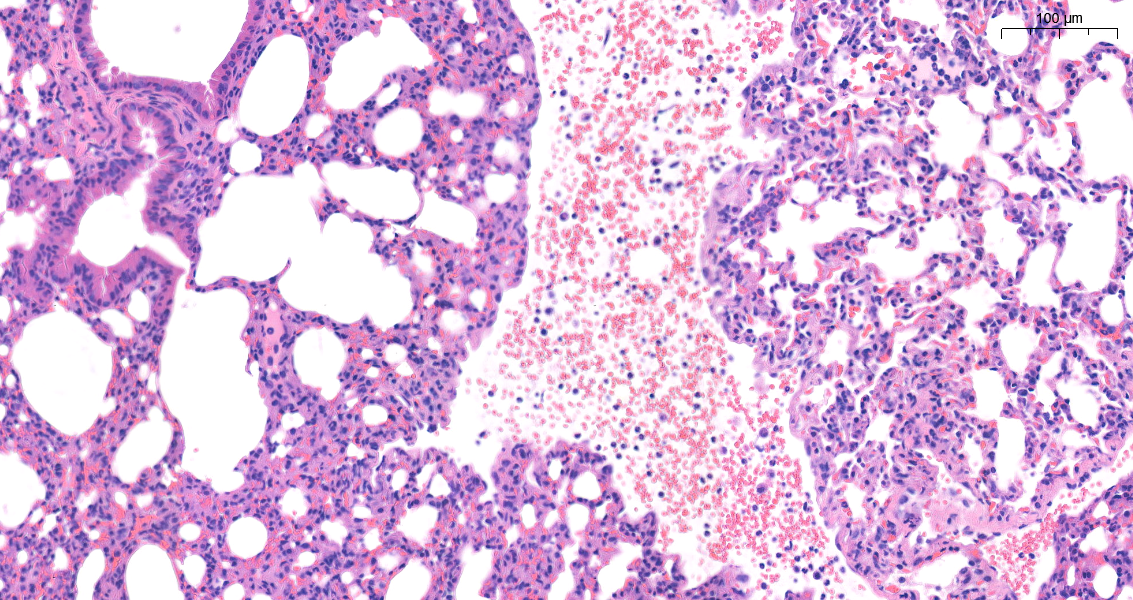

Supplement: Supplementary file 8 — Source Data Fig. 7 [file 44319_2023_47_MOESM8_ESM.zip › EMBOR-2023-57416V3-Figure_7_Source_Data-sd/Figure 7/F/METTL3 WT-M.pneumoniae.tif]

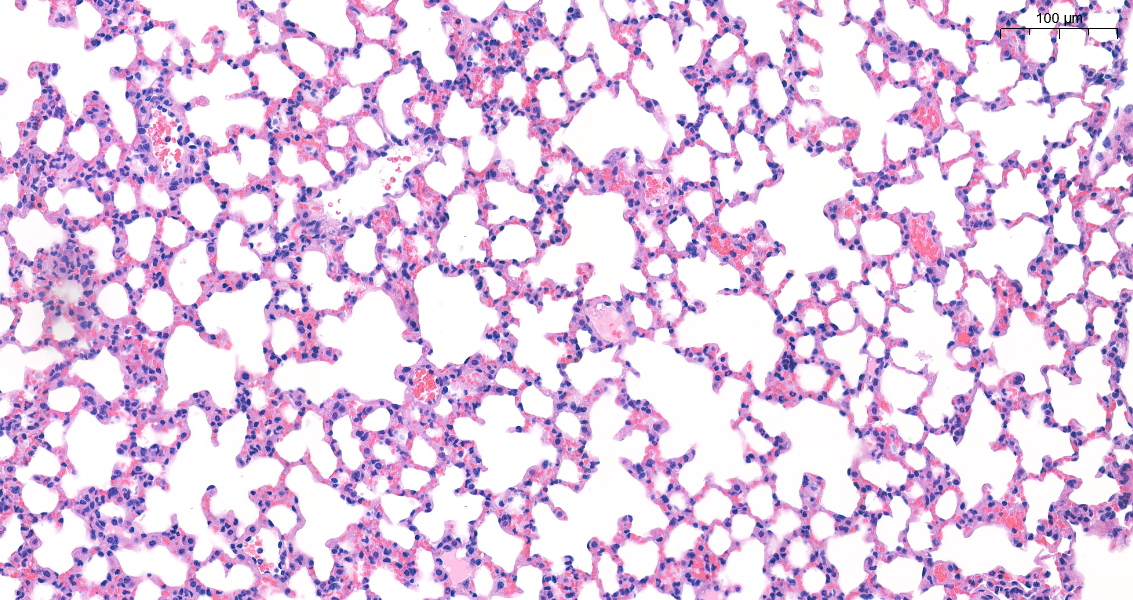

Supplement: Supplementary file 8 — Source Data Fig. 7 [file 44319_2023_47_MOESM8_ESM.zip › EMBOR-2023-57416V3-Figure_7_Source_Data-sd/Figure 7/F/METTL3 WT-Mock.tif]

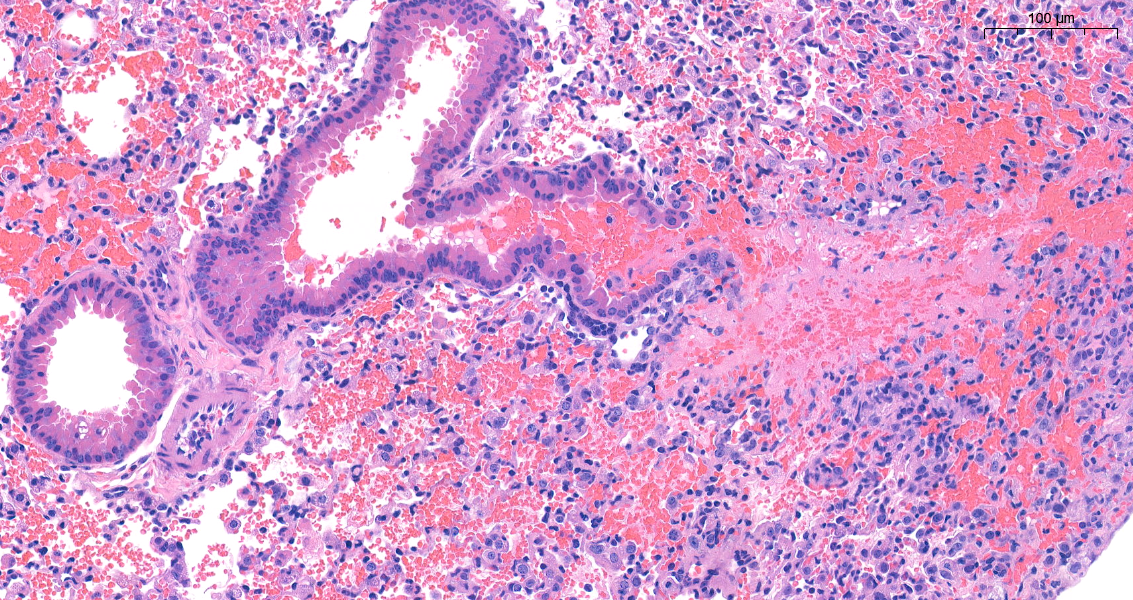

Supplement: Supplementary file 8 — Source Data Fig. 7 [file 44319_2023_47_MOESM8_ESM.zip › EMBOR-2023-57416V3-Figure_7_Source_Data-sd/Figure 7/F/METTL3 WT-P.multocida.tif]

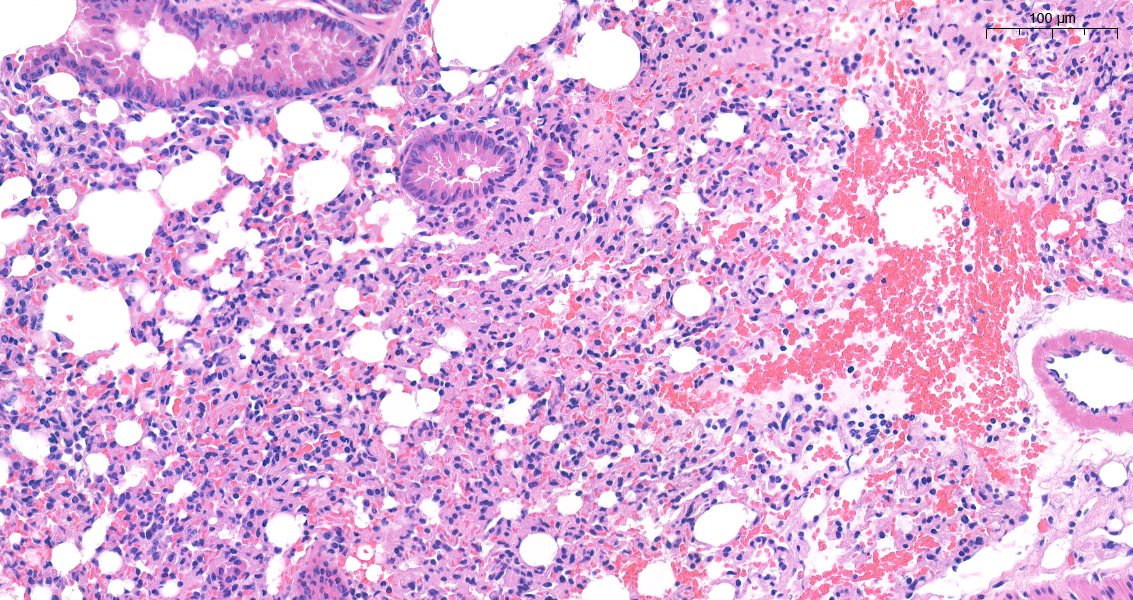

Supplement: Supplementary file 8 — Source Data Fig. 7 [file 44319_2023_47_MOESM8_ESM.zip › EMBOR-2023-57416V3-Figure_7_Source_Data-sd/Figure 7/F/METTL3 WT-S.aureus.tif]

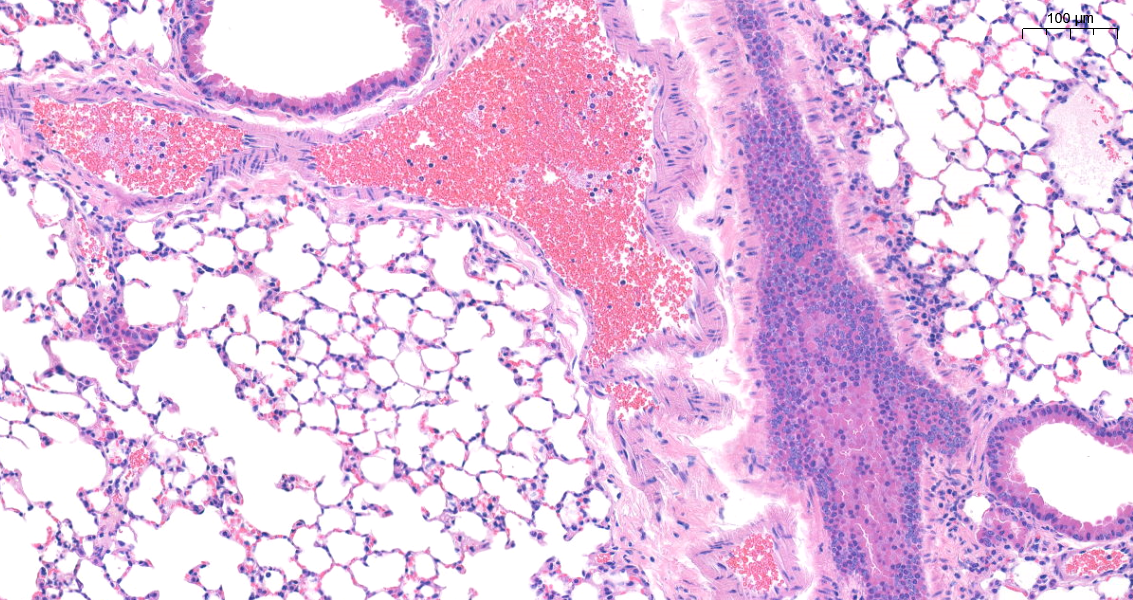

Supplement: Supplementary file 8 — Source Data Fig. 7 [file 44319_2023_47_MOESM8_ESM.zip › EMBOR-2023-57416V3-Figure_7_Source_Data-sd/Figure 7/F/METTL3 cKO-M.pneumoniae.tif]

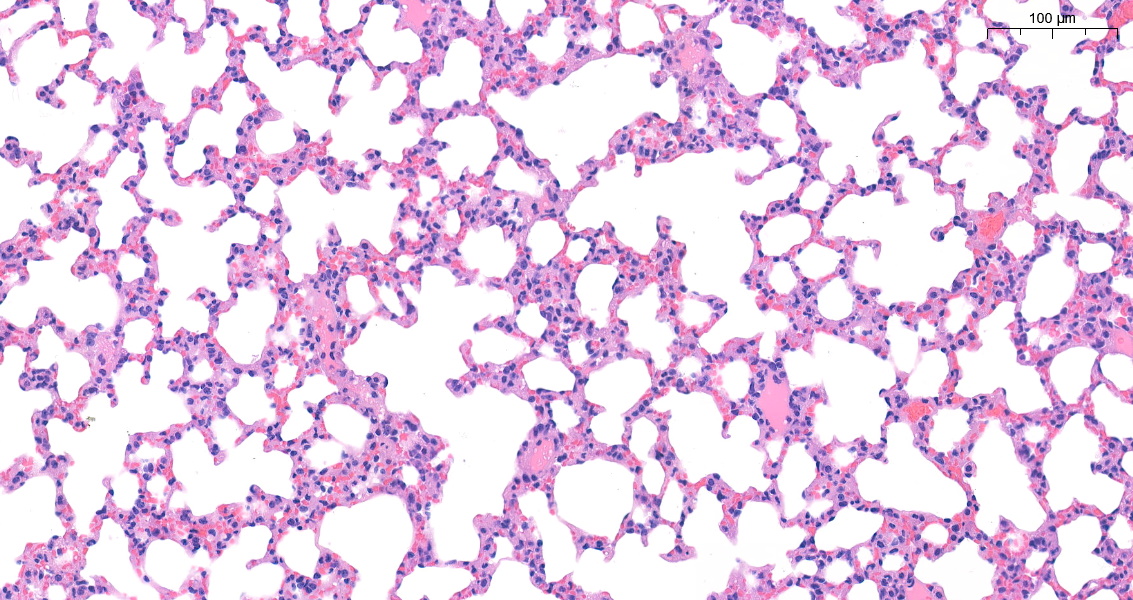

Supplement: Supplementary file 8 — Source Data Fig. 7 [file 44319_2023_47_MOESM8_ESM.zip › EMBOR-2023-57416V3-Figure_7_Source_Data-sd/Figure 7/F/METTL3 cKO-Mock.jpg]

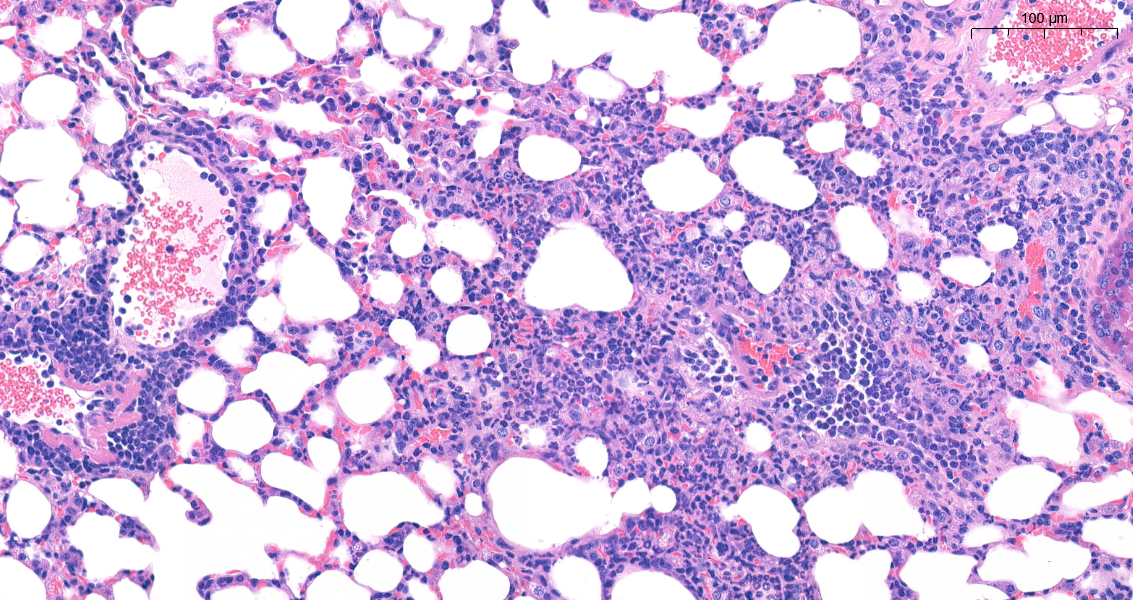

Supplement: Supplementary file 8 — Source Data Fig. 7 [file 44319_2023_47_MOESM8_ESM.zip › EMBOR-2023-57416V3-Figure_7_Source_Data-sd/Figure 7/F/METTL3 cKO-P.multocida.tif]

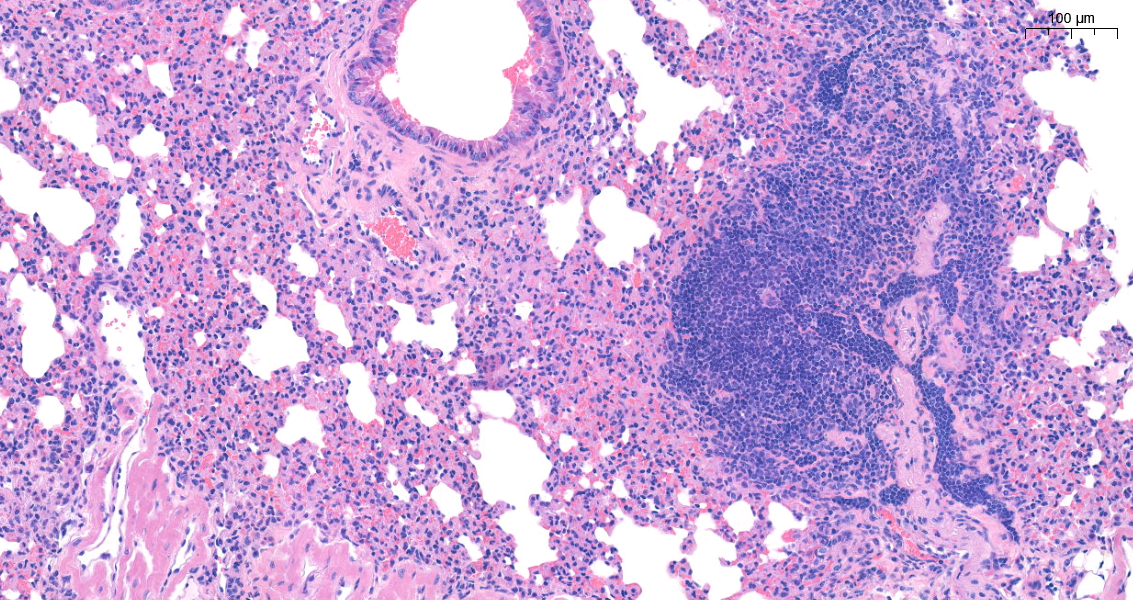

Supplement: Supplementary file 8 — Source Data Fig. 7 [file 44319_2023_47_MOESM8_ESM.zip › EMBOR-2023-57416V3-Figure_7_Source_Data-sd/Figure 7/F/METTL3 cKO-S.aureus.tif]
