# Supplementary material for: DDX5 inhibits inflammation by modulating m6A levels of TLR2/4 transcripts during bacterial infection
Source: EMBO Rep. 2024 Jan 5;25(2):19. doi: 10.1038/s44319-023-00047-9 (PMC10897170; doi:10.1038/s44319-023-00047-9)
Supplement: Supplementary file 10 — Expanded View Figures [file 44319_2023_47_MOESM10_ESM.pdf]

Expanded View Figures

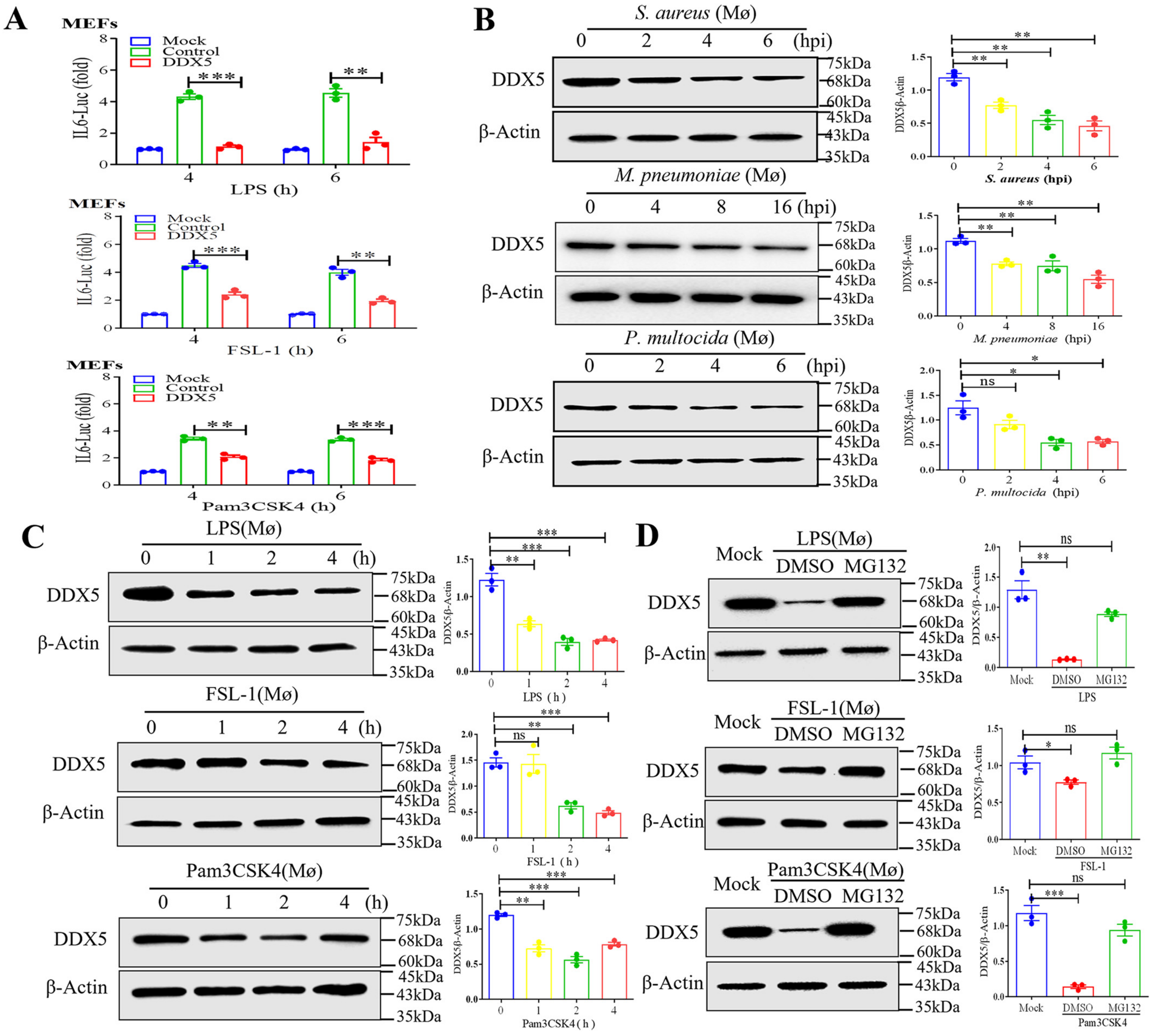

Figure EV1. Luciferase activity of the IL-6 promoter in DDX5-overexpressing MEFs and the degradation of DDX5 via K48 linked ubiquitination in Mø treated with pathogenic bacteria or TLR2/4 agonists.

(**A**) Luciferase activity of the IL-6 promoter in MEFs treated with LPS, FSL-1, and Pam3CSK4 for 4 and 6 h. (**B**) DDX5 expression in Mø infected with *S. aureus* for 0, 2, 4, and 6 h; *M. pneumoniae* for 0, 4, 8, and 16 h; *P. multocida* for 0, 2, 4, and 6 h. β-Actin as a reference control. (**C**) DDX5 expression in Mø treated with LPS, FSL-1, and Pam3CSK4 for 0, 1, 2, and 4 h. (**D**) DDX5 level in Mø treated with the MG132 proteasome inhibitor, as well as LPS, FSL-1, and Pam3CSK4 for 8 h; DMSO served as the control. The expression of DDX5 was quantified by the band intensity of DDX5/β-Actin in the western blot; the band intensity was measured by Image J software. Data information: In (**A–D**), all data are represented as the mean ± SEM of three biologically independent samples. “ns” indicates no significant difference, *$p < 0.05$, **$p < 0.01$, and ***$p < 0.001$ (Student’s t test).

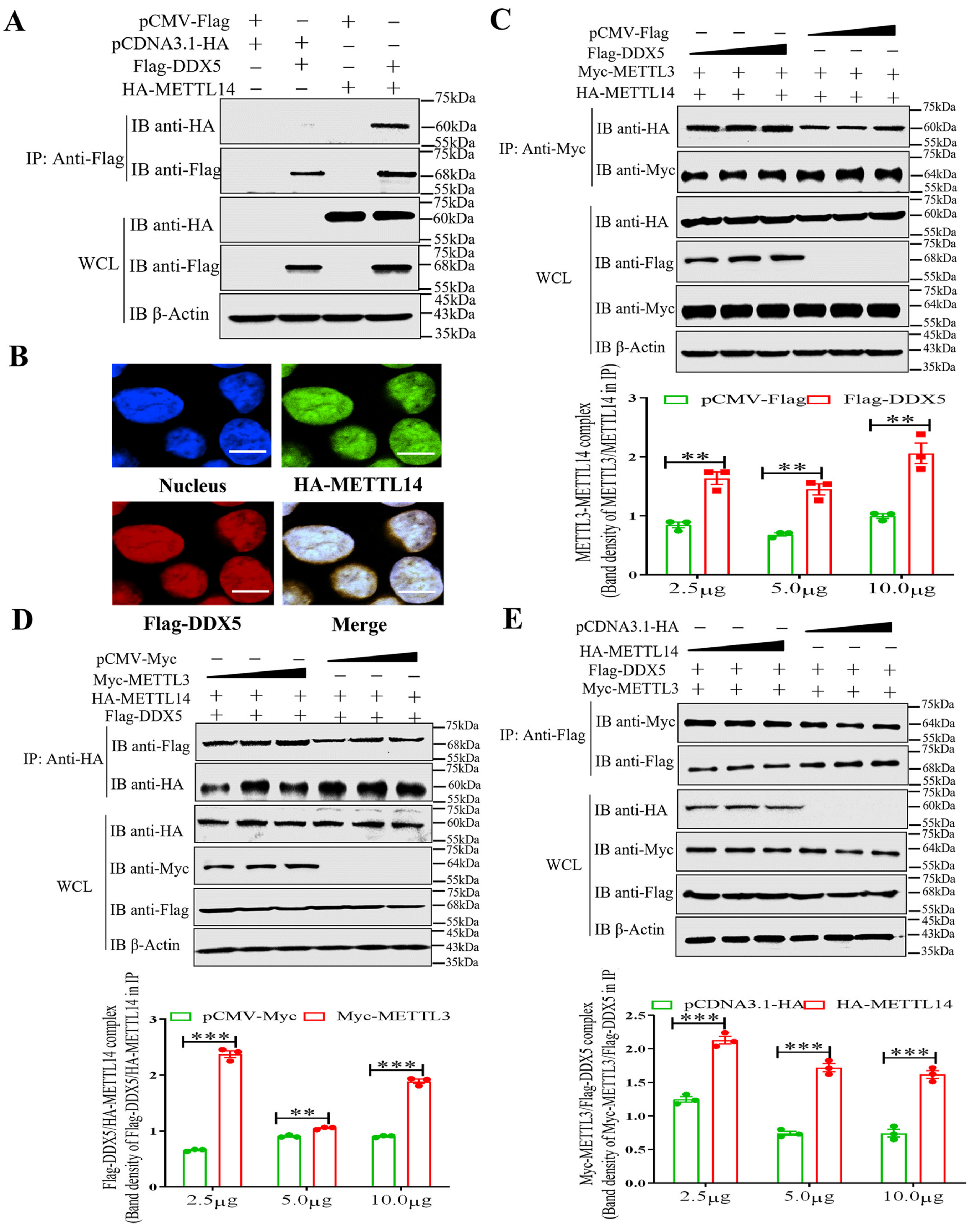
A
pCMV-Flag
pCDNA3.1-HA
Flag-DDX5
HA-METTL14
IP: Anti-Flag
IB anti-HA
IB anti-Flag
WCL
IB β-Actin
75kDa
60kDa
55kDa
68kDa
45kDa
43kDa
35kDa
B
Nucleus
HA-METTL14
Flag-DDX5
Merge
C
Myc-METTL3
IP: Anti-Myc
IB anti-Myc
64kDa
pCMV-Flag Flag-DDX5
METTL3-METTL14 complex
(Band density of METTL3/METTL14 in IP)
2.5μg
5.0μg
10.0μg
D
pCMV-Myc
IP: Anti-HA
pCMV-Myc Myc-METTL3
Flag-DDX5/HA-METTL14 complex
(Band density of Flag-DDX5/HA-METTL14 in IP
E
pCDNA3.1-HA HA-METTL14
Myc-METTL3/Flag-DDX5 complex
(Band density of Myc-METTL3/Flag-DDX5 in IP

 © The Author(s)

Figure EV2. The interplay and stability of the DDX5-METTL3-METTL14 complex in 293T cells.

(**A**) Interaction between Flag-DDX5 and HA-METTL14 in 293T cells detected by Co-IP. (**B**) Co-localization of HA-METTL14 and Flag-DDX5 in 293T cells. Scale bars: 10 μm. (**C–E**) The interplay of METTL3, METTL14, and DDX5 in 293T cells. Interaction between Myc-METTL3 and HA-METTL14 in 293T cells transfected with different doses of Flag-DDX5, Myc-METTL3, HA-METTL14 or control vector (2.5, 5.0, and 10.0 μg). The interaction of Myc-METTL3 and HA-METTL14 (**C**), HA-METTL14 and Flag-DDX5 (**D**), or Myc-METTL3 and Flag-DDX5 (**E**) was quantified by the band intensity of Myc-METTL3/HA-METTL14, Flag-DDX5/HA-METTL14, Myc-METTL3/ Flag-DDX5 in the IP system; the band intensity was measured by Image J software. Data information: In (**C–E**), all data are represented as the mean ± SEM of three biologically independent samples. $^{**}p < 0.01$ and $^{***}p < 0.001$ (Student's t test).

© The Author(s)

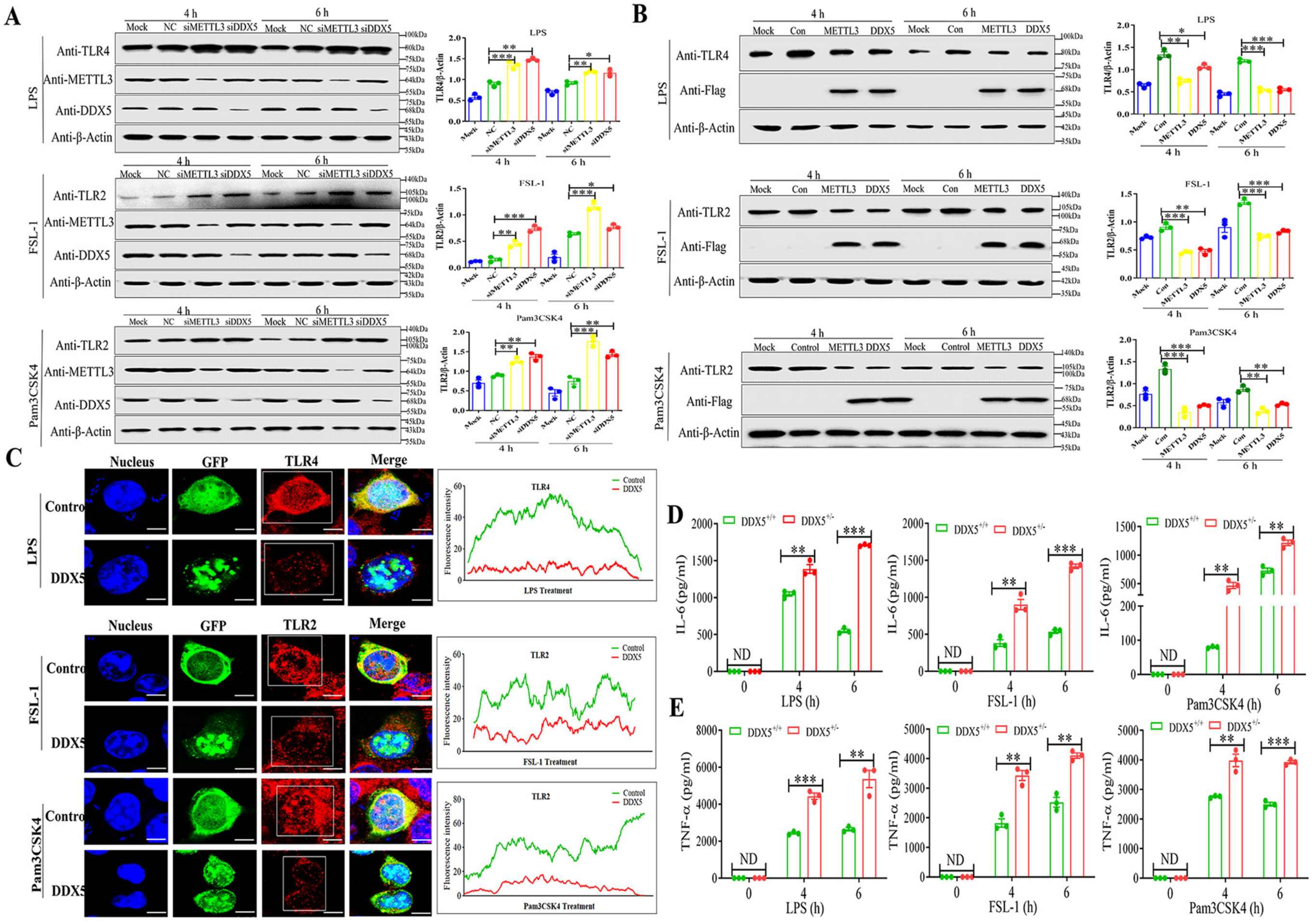

Figure EV3. mRNA m6A modification reduces the expression of TLR4/TLR2 in MEFs.

(**A**, **B**) TLR4/TLR2 protein levels in NC, siDDX5/siMETTL3 MEFs (**A**), or in DDX5/METTL3- overexpressing MEFs (**B**) treated with LPS, FSL-1 and Pam3CSK4 for 4 and 6 h, respectively, and determined by western blot. The expression of TLR4 and TLR2 were quantified by the band intensity of DDX5/β-Actin in the western blot; the band intensity was measured by Image J software. (**C**) TLR2/4 localization and expression in GFP control or GFP-DDX5 expressing MEFs. MEFs were transfected with GFP control or GFP-DDX5 expressing plasmid for 24 h, then MEFs were treated with LPS, FSL-1, or Pam3CSK4, respectively; TLR4/TLR2 were detected in MEFs by IFA and CLSM. The green fluorescence indicated GFP (Control)/GFP-DDX5 (DDX5), and the red fluorescence indicated TLR4/TLR2. The right panels show the green line (Control) and red (DDX5) pixel intensity of the white markings in the pictures. Scale bars: 10 μm. (**D**, **E**) The production of IL-6 and TNF-α in primary BMDM following treatment with TLR2/4 agonists. IL-6 (I) or TNF-α (J) production in primary DDX5$^{+/+}$ BMDM or DDX5$^{+/-}$ BMDM treated with LPS, FSL-1, or Pam3CSK4 for 4 and 6 h ($n = 3$). Data information: In (**A**, **B**, **D**, **E**), all data are represented as the mean ± SEM of three biologically independent samples. ND not detected. *$p < 0.05$, **$p < 0.01$, and ***$p < 0.001$ (Student's t test).

 © The Author(s)

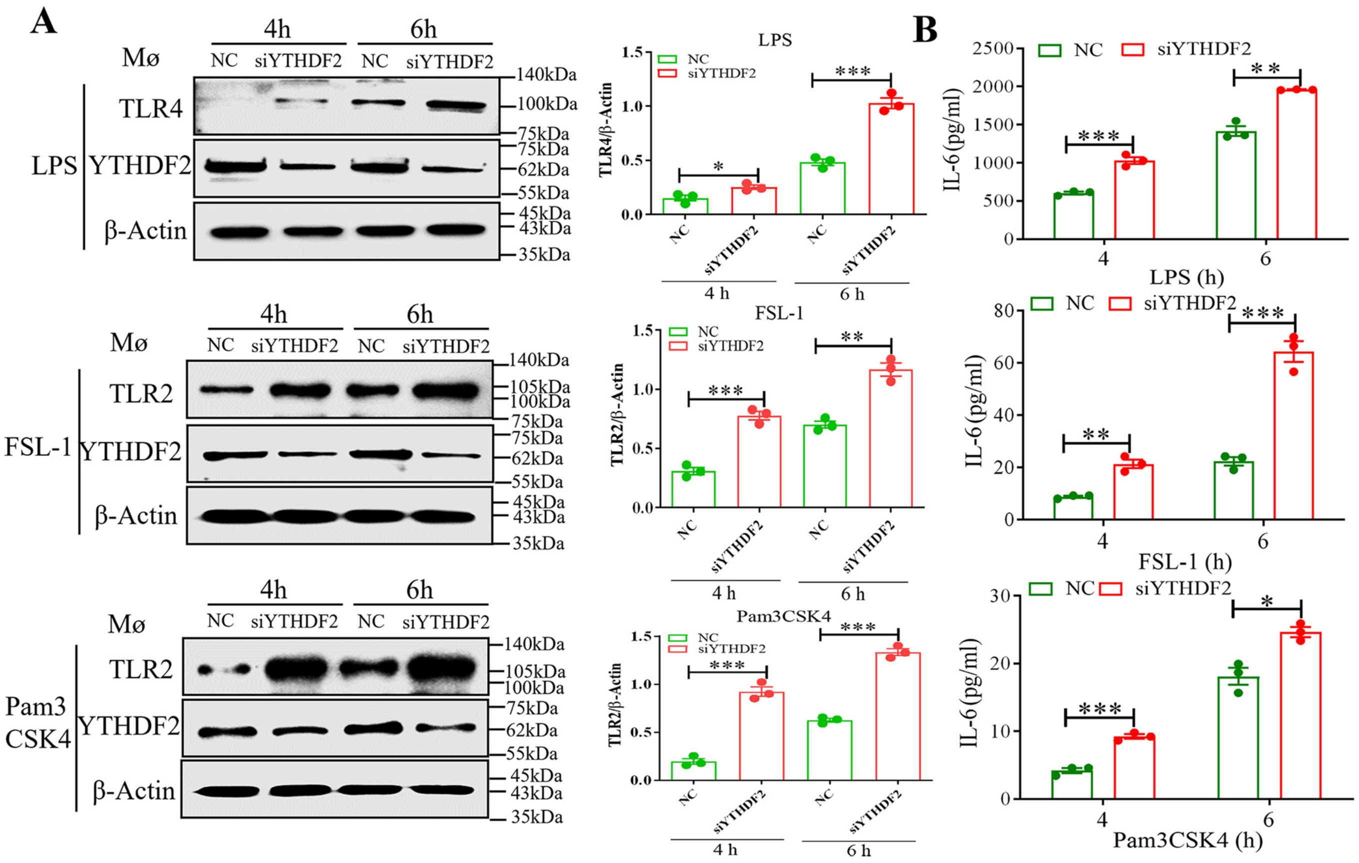

Figure EV4. Expression of TLR2/TLR4 and IL-6 production in YTHDF2 knockdown mouse macrophages (Mø) treated with LPS, FSL-1, and Pam3CSK4.

(**A**) TLR4/TLR2 protein level in NC and siYTHDF2 mouse macrophages (Mø) treated with LPS, FSL-1, or Pam3CSK4 for 4 or 6 h. (**B**) IL-6 production in NC and siYTHDF2 mouse macrophages (Mø) treated with LPS, FSL-1, and Pam3CSK4. The expression of TLR4 and TLR2 were quantified by the band intensity of TLR4/β-Actin or TLR2/β-Actin in the western blot; the band intensity was measured by Image J software. Data information: In (**A**, **B**), all data are represented as the mean ± SEM of three biologically independent samples. *$p < 0.05$, **$p < 0.01$, and ***$p < 0.001$ (Student's *t* test).

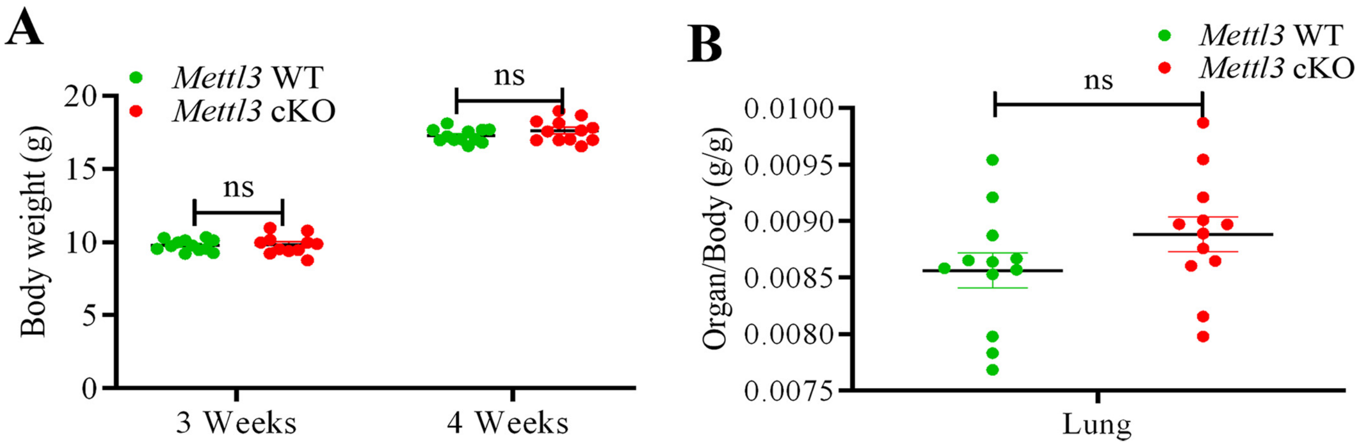

Figure EV5. Quantification of the organ-body rate of *Mettl3* cKO and *Mettl3* WT mice.

(**A**) Quantification of *Mettl3* WT or *Mettl3* cKO mice at postnatal 3 and 4 weeks ($n = 12$). (**B**) Organ-body rate was quantified between *Mettl3* WT and *Mettl3* cKO littermates ($n = 12$). Data information: In (**A**, **B**), all data are represented as the mean ± SEM of 12 biologically independent samples. "ns" indicates no significant difference (Student's *t* test).

 © The Author(s)
